# The 5:2 diet does not increase adult hippocampal neurogenesis or enhance spatial memory in mice

**DOI:** 10.15252/embr.202357269

**Published:** 2023-11-21

**Authors:** Luke D Roberts, Amanda KE Hornsby, Alanna Thomas, Martina Sassi, Aimee Kinzett, Nathan Hsiao, Bethan R David, Mark Good, Timothy Wells, Jeffrey S Davies

**Affiliations:** ^1^ Molecular Neurobiology, Institute of Life Sciences, School of Medicine Swansea University Swansea UK; ^2^ School of Biosciences Cardiff University Cardiff UK; ^3^ School of Psychology Cardiff University Cardiff UK

**Keywords:** 5:2 diet, dietary restriction, ghrelin receptor, intermittent fasting, neurogenesis, Metabolism, Neuroscience, Stem Cells & Regenerative Medicine

## Abstract

New neurones are generated throughout life in the mammalian brain in a process known as adult hippocampal neurogenesis (AHN). Since this phenomenon grants a high degree of neuroplasticity influencing learning and memory, identifying factors that regulate AHN may be important for ameliorating age‐related cognitive decline. Calorie restriction (CR) has been shown to enhance AHN and improve memory, mediated by the stomach hormone, ghrelin. Intermittent fasting (IF), a dietary strategy offering more flexibility than conventional CR, has also been shown to promote aspects of AHN. The 5:2 diet is a popular form of IF; however, its effects on AHN are not well characterised. To address this, we quantified AHN in adolescent and adult wild‐type and ghrelin‐receptor‐deficient mice following 6 weeks on a 5:2 diet. We report an age‐related decline in neurogenic processes. However, the 5:2 diet does not increase AHN nor enhance memory performance, suggesting that this specific form of IF is ineffective in promoting brain plasticity to support learning.

## Graphical abstract

Intermittent fasting, in the form of a 5:2 diet, does not induce hippocampal neurogenesis and learning in adolescent or adult mice of either sex.

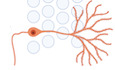

## Introduction

In order to effectively combat age‐related morbidities, both in the brain and at the systemic level, appropriate interventions that target the underlying molecular and cellular hallmarks are required. A promising candidate in this regard is dietary restriction (DR), a group of interventions that reduce either daily caloric intake, macronutrient intake, feeding duration or a mixture of each, without inducing malnutrition (Fontana & Partridge, 2015; Di Francesco *et al*, 2018). These paradigms have emerged as robust interventions for delaying and preventing the incidence of age‐related disease and metabolic dysfunction in rodents and non‐human primates (Fontana & Partridge, 2015; Mattison *et al*, 2017; Di Francesco *et al*, 2018).

While DR exists in numerous forms, notable examples include calorie restriction (CR), where daily calorie intake is reduced (typically by 20–40%) but meal frequency and timing is not constrained; and intermittent fasting (IF) diets, where individuals undergo extended periods (16–48 h) with very limited or no food intake, with the resumption of *ad libitum* eating habits in between (Mattson *et al*, 2017; Di Francesco *et al*, 2018).

There are likely to be myriad cellular and molecular mechanisms underlying the beneficial effects of DR (Mattson *et al*, 2017; Di Francesco *et al*, 2018). However, a notable paradigm implicated in DR‐regulated brain plasticity is the birth of new neurones in the adult hippocampus (Morgan *et al*, 2017; Buntwal *et al*, 2019). This phenomenon, known as adult hippocampal neurogenesis (AHN), provides a high degree of neuroplasticity and regulates spatial memory function and mood‐like behaviours. AHN declines markedly with age, therefore, identifying factors that regulate AHN are potentially significant for attenuating age‐related cognitive decline (Mu & Gage, 2011).

IF and periodic fasting mimicking diets (FMD) (Brandhorst *et al*, 2015) have been shown to promote aspects of neurogenesis including cell proliferation (Brandhorst *et al*, 2015; Dias *et al*, 2021), new cell survival (Lee *et al*, 2000, 2002a,b; Dias *et al*, 2021) and neurone differentiation (Dias *et al*, 2021). Moreover, CR increases neurone differentiation, the number of new adult born hippocampal neurones and improves hippocampal‐dependent cognition by signalling via the stomach hormone, ghrelin (Hornsby *et al*, 2016).

Ghrelin, which exists as acylated and unacylated forms in the circulation, is secreted from the stomach during periods of fasting (Tschop *et al*, 2000) to stimulate feeding (Nakazato *et al*, 2001) and glucose homeostasis (Mani & Zigman, 2017). Exogenous administration of acyl‐ghrelin stimulated the proliferation and neuronal differentiation of hippocampal progenitors in adult mice (Moon *et al*, 2009). Subsequently, cell proliferation in the dentate gyrus (DG), as well as the proportion of new BrdU^+^ cells that express markers of immature neurones (BrdU^+^/DCX^+^), neurone differentiation (% of BrdU^+^/NeuN^+^) and mature neurones (BrdU^+^/NeuN^+^), were reported to be decreased in ghrelin‐knockout (GKO) mice (Li *et al*, 2013). Ghrelin administration restored levels of neuronal differentiation to those of C57BL/6J controls, supporting its pro‐neurogenic role (Li *et al*, 2013). In addition, the stress‐induced reduction in AHN in a mouse model of chronic psychosocial stress is more severe in mice lacking the ghrelin‐receptor, growth hormone secretagogue receptor (GHSR) (Walker *et al*, 2015).

Notably, we showed that two weeks of daily acyl‐ghrelin injections, at physiological levels, significantly increased the number of new adult‐born neurones in the dentate gyrus of the hippocampus. The acyl‐ghrelin‐treated rats also displayed enhanced pattern separation performance in a Spontaneous Location Recognition task conducted 8–10 days following the end of treatment, indicating that acyl‐ghrelin had a long‐lasting effect on spatial memory (Kent *et al*, 2015). Similarly, our data suggested that the beneficial effects of CR on AHN are also mediated by acyl‐ghrelin as a short period of 30% CR increased the number of new adult‐born hippocampal neurones in a ghrelin‐receptor‐dependent manner (Hornsby *et al*, 2016). Wild‐type and GKO mice maintained for 3 months on alternate day fasting (ADF) or *ad libitum* diet reported that DR increased the number of new adult‐born cells in wild‐type mice, but not in GKO mice, suggesting that acyl‐ghrelin signalling is required for the beneficial effects of DR on aspects of neurogenesis (Kim *et al*, 2015). Daily systemic injections of acyl‐ghrelin for 8 days enhanced AHN (Zhao *et al*, 2014) and acyl‐ghrelin treatment rescued abnormal neurogenesis in a mouse model of AD (5xFAD) (Moon *et al*, 2014). These studies provide strong evidence in support of the pro‐neurogenic role of acyl‐ghrelin signalling (Kent *et al*, 2015).

The precise mechanisms by which acyl‐ghrelin signalling mediates AHN remain unclear; however, this may be via non‐cell autonomous pathways involving the release of pro‐neurogenic factors (Buntwal *et al*, 2019; Sassi *et al*, 2022). More recently, we showed that acyl‐ghrelin increased BDNF mRNA—a neurotrophic factor—within the granule cell layer of the hippocampus (Hornsby *et al*, 2020). Consistent with an increase in BDNF, acyl‐ghrelin binding to GHSR on hippocampal neurones promoted dendritic spine formation, increased synaptic plasticity and LTP (Diano *et al*, 2006).

The 5:2 diet is a popular form of IF in which the usual calorie intake is restricted on 2 non‐consecutive days within the week, while a normal diet is consumed on the remaining 5 days (Patterson *et al*, 2015). Reducing the normal calorie intake to 25% for 2 days per week improves several health biomarkers (Harvie *et al*, 2011). However, unlike for conventional CR and ADF diets, studies exploring the impact of the 5:2 diet on brain health and AHN are limited. One study reported that a four‐week 5:2 diet improved pattern separation performance but impaired memory retention in an adult population with central obesity (Kim *et al*, 2020). As relatively little is known about the impact of the 5:2 diet on AHN and pattern separation memory, we address this gap in knowledge by testing the hypothesis that the 5:2 diet promotes AHN and cognition via ghrelin‐receptor signalling.

## Results

### 6‐weeks of 5:2 diet feeding did not reduce body weight

**Figure 1:**
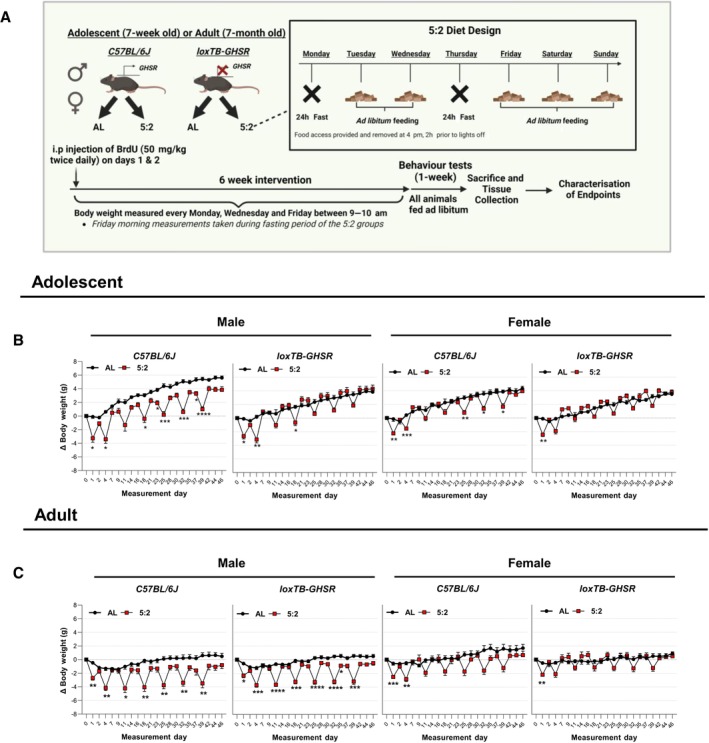
Body weight changes over time following 5:2 diet or *ad libitum* feeding Schematic overview of the experimental design used to investigate the effects of the 5:2 diet in mice. Mice in 5:2 diet groups underwent two non‐consecutive 24 h fasting days (food removed at 4 pm and access provided at 4 pm the following day) per week for 6‐weeks before re‐feeding on an *ad libitum* diet for a further week. The body weight of each individual mouse was recorded every Monday, Wednesday and Friday, with the Friday measurements taken during the fasting period of the 5:2 diet groups.Body weight changes over time for adolescent mice. Body weight changes (Δ Body Weight) across the experimental time‐course were calculated by subtracting the starting weight of each individual mouse from all subsequent recorded body weights. The effect of 5:2 diet compared to *ad libitum* (AL) controls was assessed separately for both male and female *C57BL/6J* and *loxTB‐GHSR* mice.Body weight changes over time for adult mice (same methodology as B). Data Information: Symbols represent mean values with SEM error bars. Statistical comparisons made using repeated measures (RM) 2‐Way ANOVA followed by Šídák's multiple comparisons of *ad libitum* vs. 5:2 diet at each time point. *P ≤ 0.05; **P ≤ 0.01; ***P ≤ 0.001; ****P ≤ 0.0001. For time points lacking a symbol of statistical significance *P* ≥ 0.05. *n* = 6–9 mice per group. Source data are available online for this figure.

To assess the effect of the 5:2 diet on AHN and underlying involvement of ghrelin signalling, adolescent (7‐week‐old) and adult (7‐month‐old) homozygous loxTB‐GHSR mice and their wild type (C57BL/6J) littermate equivalents were subjected to two non‐consecutive fasting days (Monday and Thursday) per week for a period of 6 weeks (Fig 1A). To avoid the potential metabolic consequences of disrupting the circadian regulation of food intake (Glad *et al*, 2011), chow was returned to IF mice at 16:00 h, 2 h before lights off, on re‐feeding days (Tuesday and Friday). This was followed by a week of *ad libitum* feeding, in which hippocampal learning and memory was assessed via the object in place (OIP) task while the open field task was used to assess exploratory and anxiety‐like behaviour. Control animals were fed *ad libitum* for the entire duration of the study.

Endpoint body weight analyses (Fig 1B and C; Appendix Table S1) revealed that 6 weeks of feeding on the 5:2 diet paradigm did not lead to significant weight loss compared to *ad libitum* controls in any of the genotypes, sexes or age groups under study. However, repeated body weight change measurements (relative to baseline) over the course of the experimental time course indicated considerable fluctuations in body weight for mice fed on the 5:2 diet, with animals losing weight in response to fasting and subsequently regaining this weight upon re‐feeding (Fig 1B and C). While the temporal pattern of body weight on the 5:2 diet was observed irrespective of genotype, sex or age (Time × Diet: *P* ≤ 0.0001 for all groups in Fig 1B and C), the extent of the body weight fluctuations appeared to be more prominent in adolescent male C57BL/6J mice (Diet: *P* ≤ 0.0001) as well as adult males of both genotypes (Diet: C57BL/6J: *P* = 0.0051; loxTB‐GHSR: *P* = 0.0003). No significant main diet effects were found for adolescent male loxTB‐GHSR mice or any female mice, while these groups also had fewer significant post hoc differences between *ad libitum* and 5:2 diets (Fig 1B and C). These findings suggest that there is sexual dimorphism in the metabolic response to the 5:2 diet, despite an overall lack of weight loss at the end of the dietary regimen.

Additional endpoint characterisations of nose‐anus length (mm) and tibial length (mm) did not reveal any significant effects for the 5:2 diet (Appendix Table S1). However, endpoint growth and body weight characterisations did reveal several significant findings for loss of GHSR expression. Indeed, adolescent male loxTB‐GHSR mice (*P* = 0.0110) as well as both male (*P* = 0.0080) and female (*P* = 0.0021) adult loxTB‐GHSR mice weighed significantly less than sex and age matched C57BL/6J mice (Appendix Table S1). Moreover, adolescent female loxTB‐GHSR mice had significantly shorter nose‐anus lengths (*P* = 0.0342), while both male (*P* = 0.0259) and female (*P* = 0.0015) adult loxTB‐GHS‐R mice had significantly shorter tibial lengths compared to C57BL/6J counterparts.

The temporal fluctuations in body weight on the 5:2 diet, as well as the lack of overall weight loss, suggest that there is elevated food consumption following the recommencement of *ad libitum* feeding on non‐fasting days. To further investigate the association between 5:2 mediated weight loss and food intake, a separate 3‐week investigation was conducted in adolescent male and female C57BL6/J mice, with animals being fed *ad libitum* or with the same 5:2 dietary paradigm used previously. For the first 11 days of the investigation, mice were group housed and daily body weight measurements were recorded.

**Figure EV1:**
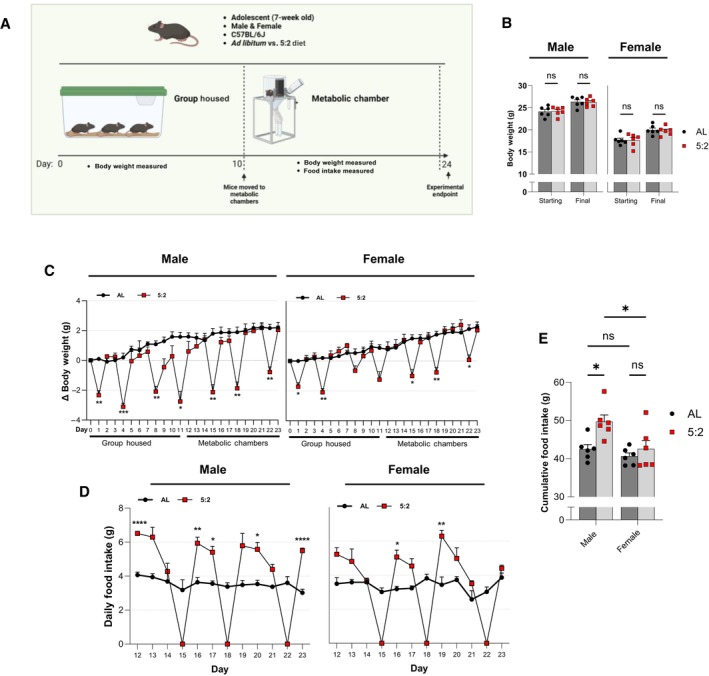
Association between body weight change and food intake during 5:2 dietary regimen (related to Fig 1) A
Schematic overview of the experimental design. 7‐week‐old male and female C57BL/6J mice were fed either *ad libitum* or on the 5:2 diet for 24 days. Mice were group housed for the first 10 days of the intervention before being moved to individual metabolic chambers to measure daily food intake in addition to body weight.B
Starting (day 0) and final (day 23) body weights in male (left) and female (right) mice.C
Daily body weight changes (Δ Body Weight) over the experimental time course.D
Daily food intake between days 12 and 23.E
Cumulative food intake at the end of the recording period. Data Information: For (B) and (E), symbols represent individual mice, bars represent mean values, error bars represent SEM. For (C) and (D), symbols represent mean values, error bars represent SEM. Statistical comparisons made using RM 2‐way ANOVA for (B–D) and ordinary 2‐way ANOVA for (E). Šídák's multiple comparisons used for post hoc assessments. ns *P* ≥ 0.05; *P ≤ 0.05; **P ≤ 0.01; ***P ≤ 0.001; ****P ≤ 0.0001. *n* = 6 mice per group. Source data are available online for this figure.

Subsequently, mice were moved to individual metabolic chambers for recording food intake in addition to body weight. As with our previous findings, the 5:2 dietary regimen did not lead to weight loss at the end of the investigation (Fig EV1B) and resulted in considerable body weight fluctuations over time (Fig EV1C). Notably, food intake data confirmed that mice fed on the 5:2 diet generally consumed more food on non‐fasting days compared to mice fed on an *ad libitum* diet. The effect of the 5:2 diet on rebound feeding was clear for male mice (Main effect of diet: *P* = 0.0073), with significantly higher food consumption compared to *ad libitum* controls on days 12 (*P* ≤ 0.0001), 16 (*P* = 0.0062), 17 (*P* = 0.0226), 20 (*P* = 0.0310) and 23 (*P* ≤ 0.0001) (Fig EV1D). While analysis of cumulative food intake over one 7‐day cycle of the dietary regimen (days 17–23) reported no statistically significant differences, analysis over the 12‐day measurement period reported that male mice fed on the 5:2 diet had significantly higher cumulative food intake (*P* = 0.0196) than *ad libitum* controls (Fig EV1E). While female 5:2 fed mice displayed a similar trend to male mice, significant rebound feeding compared to *ad libitum* mice was only observed on days 16 (*P* = 0.0464) and 19 (*P* = 0.0086) (Fig EV1D) and there was no significant increase in cumulative food intake over the 12‐day period (*P* = 0.8750) (Fig EV1E). These data indicate that a 5:2 diet with two non‐consecutive fasting days per week does not reduce cumulative food intake in females, but increases it in males.

### Cell proliferation in the hippocampus was not altered by the 5:2 diet

**Figure 2:**
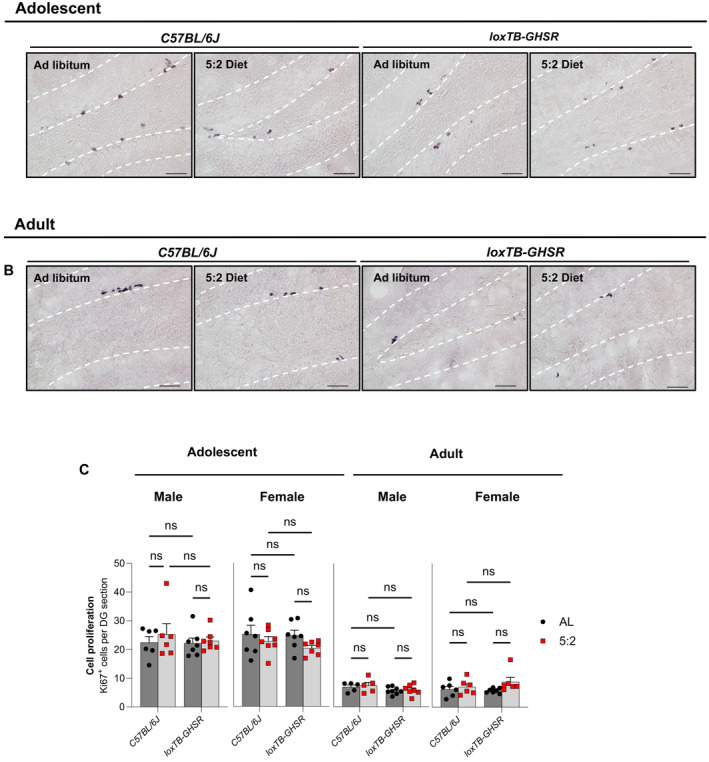
5:2 diet does not significantly affect cell proliferation in the dentate gyrus (DG) Representative light microscopy images of positive Ki67 immunoreactivity, indicative of proliferating cells, in the dentate gyrus (DG) of adolescent male mice. Scale bars represent 50 μm.Representative images of Ki67^+^ cells in adult male mice. Scale bars represent 50 μm.Average number of Ki67^+^ cells per DG section. Ki67 cell counts for each DG section were quantified using Image J. The effect of diet (*ad libitum* vs. 5:2 diet) was assessed in the presence and absence of functional ghrelin receptor (GHSR) expression (*C57BL/6J* vs. *loxTB‐GHSR*) for both male and female mice of both adolescent and adult age groups. Data Information: Symbols represent individual mice, bars represent mean values, error bars represent SEM. Statistical comparisons made using ordinary 2‐way ANOVA followed by Šídák's multiple comparisons. ns ≥ 0.05. *n* = 5–8 mice per group. Source data are available online for this figure.

To assess the effect of the 5:2 diet on cell proliferation (including Neural Stem Cells [NSCs]) in the DG of the hippocampus, we quantified Ki67 positive cells using DAB immunohistochemistry (Fig 2A and B; Appendix Table S2). Adolescent mice generally had lower Ki67^+^ cell counts compared to adult animals (Fig 2C). However, across both age groups and both sexes, we found no statistical evidence that the 5:2 alters the abundance of proliferating cells (Adolescent, Male: *P* = 0.5960; Adolescent, Female: *P* = 0.1070; Adult, Male: *P* = 0.5064; Adult, Female: *P* = 0.1543) (Fig 2C). This is consistent with our previous CR study (Hornsby *et al*, 2016) and ADF studies from other research groups (Lee *et al*, 2000, 2002a,b; Bondolfi *et al*, 2004; Kim *et al*, 2015).

Interestingly, our analyses did reveal a significant main genotype effect on cell proliferation in adult males (*P* = 0.0072) but not in adult females (*P* = 0.2979) or adolescent mice (Males: *P* = 0.6685; Females: *P* = 0.5030).

### The 5:2 diet does not increase the number of immature neurones in the hippocampus

**Figure 3:**
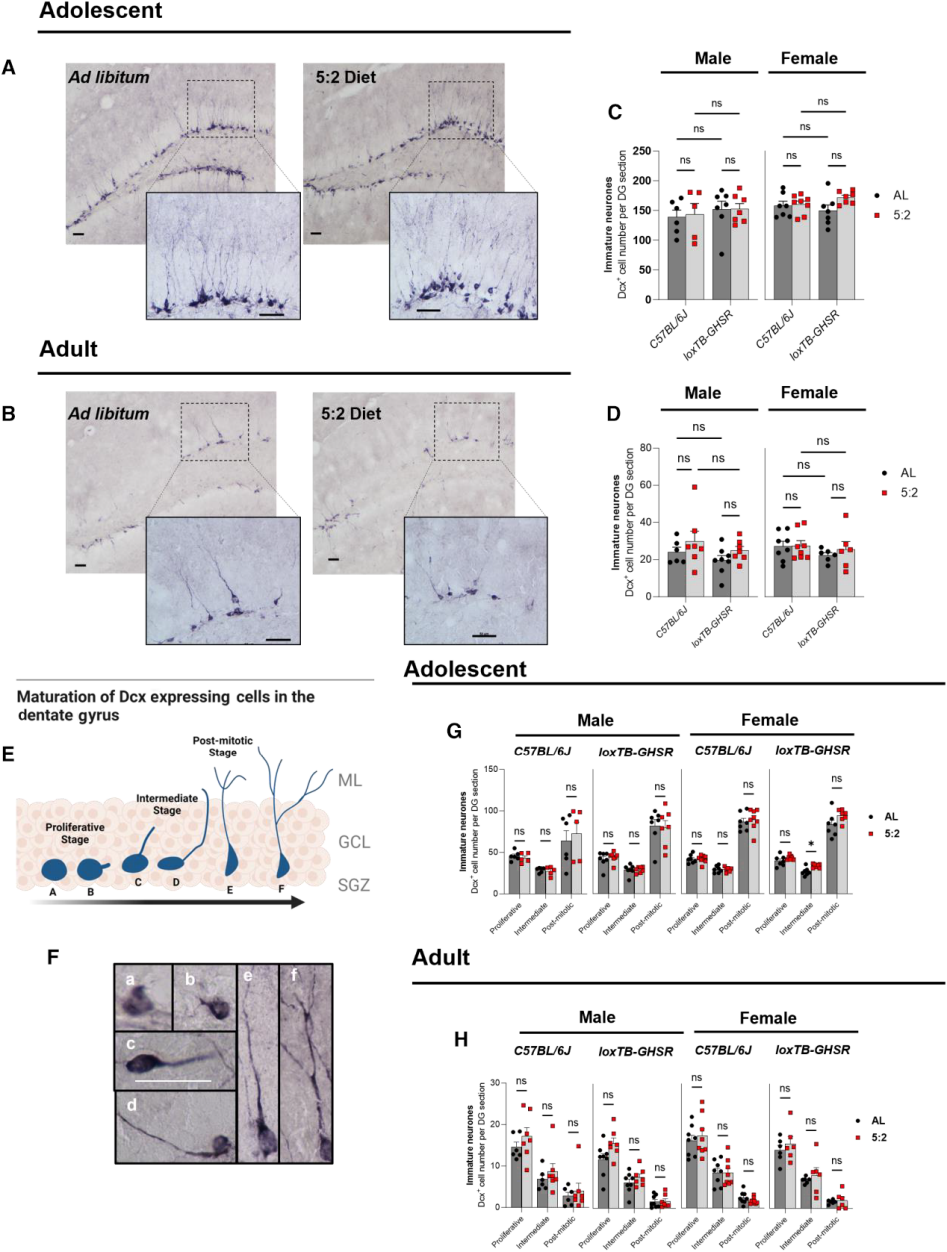
Effect of 5:2 diet on immature neurone abundance and maturation Representative light microscopy images of DCX^+^ immunoreactivity, indicative of immature neurones, in the dentate gyrus (DG) of adolescent male mice. Scale bars represent 50 μm.Representative images of DCX^+^ immunoreactivity in adult male mice. Scale bars represent 50 μm.Average number of DCX^+^ cells per DG section in adolescent mice. DCX cell counts for each DG section were quantified using image J. The effect of diet (*ad libitum* vs. 5:2 diet) was assessed in the presence and absence of functional ghrelin receptor (GHSR) expression (*C57BL/6J* vs. *loxTB‐GHSR*) for both males and females.Average number of DCX^+^ cells per DG section in adult mice (same methodology as C).Schematic illustration of the maturation process that DCX expressing cells undergo in the neurogenic niche of the DG. Morphological categorisations based on the criteria established by Plümpe *et al* (2006).Representative microscopy images of DCX expressing cell morphologies a to f. Scale bar (shown in image of morphology c cell) represents 50 μm.Average number of proliferative, intermediate and post‐mitotic DCX expressing cells in adolescent mice. The effect of diet (*ad libitum* vs. 5:2 diet) was assessed independently for both sexes and both genotypes.Average number of proliferative, intermediate and post‐mitotic DCX expressing cells in adult mice (same methodology as G). Data Information: Symbols represent individual mice, bars represent mean values, error bars represent SEM. Statistical comparisons made using ordinary 2‐way ANOVA for (C) and (D) and RM 2‐way ANOVA for (G) and (H). Šídák's multiple comparisons used for *post hoc* assessments. *P ≤ 0.05; ns *P ≥* 0.05. *n* = 5–8 mice per group. Source data are available online for this figure.

Next, we quantified immature neurones in the DG using DAB‐based IHC against doublecortin (DCX; Fig 3A and B; Appendix Table S2). We observed a striking age‐related decline in the number of DCX^+^ cells, with adult animals (Fig 3D) having considerably fewer immature neurones than adolescent animals (Fig 3C), regardless of sex, diet or genotype. As was the case for proliferating cells, we found no evidence that the 5:2 diet significantly effects the total number of immature neurones in the DG of adolescent (Fig 3C) (male: *P* = 0.8465; female: *P* = 0.0896) or Adult (Fig 3D) (male: *P* = 0.1254; female: *P* = 0.5800) mice.

Moreover, loss of GHSR expression did not alter DCX^+^ cell number either (Fig 3C and D) (Adolescent male: *P* = 0.4074; Adolescent female: *P* = 0.8334; Adult male: *P* = 0.1254; Adult female: *P* = 0.2427).

While DCX is generally used as a marker of immature neurones, the period of DCX expression during AHN (~ 3 weeks) covers both proliferative (type 2b NSCs & neuroblasts) and post‐mitotic stages (Plümpe *et al*, 2006). DCX immune‐positive cells therefore display a range of morphologies that are representative of distinct phases of neurogenic regulation and developing electrophysiological properties (Plümpe *et al*, 2006) (Fig 3E and F). Indeed, Plümpe *et al* (2006) categorised DCX expressing cells into 6 categories (*a–f*) according to the morphology of apical dendrites. As shown in Fig 3E and F, categories *a* and *b* represent proliferating cells, with the cells of category *a* displaying no processes and category *b* cells displaying short, plump processes. Cells in categories *c* and *d*, which represent an intermediate stage of maturation, display single processes without any discernible dendritic arborisation. The process of category *d* cells is longer than that of category *c* cells, typically reaching into the molecular layer. The final two categories, *e–f*, consist of post‐mitotic cells with cells of category *e* displaying a single prominent dendrite branching into the molecular layer, while category *f* cells display a more complex dendritic arbour.

Thus, to delineate whether the 5:2 diet effects the maturation of DCX expressing cells, we assessed the number (per DG section) (Fig 3G and H; Appendix Table S3) of proliferative (*a–b*), intermediate (*c–d*), and post‐mitotic (*e–f*) DCX expressing cell morphologies. In the adolescent age group (Fig 3G), there was no significant effect of the 5:2 diet on DCX^+^ cell maturation for male C57BL/6J (Diet: *P* = 0.8395; Diet × Maturation Stage: *P* = 0.7093) and loxTB‐GHSR mice (Diet: *P* = 0.9641; Diet × Maturation Stage: *P* = 0.8588) or female C57BL/6J mice (Diet: *P* = 0.7991; Diet × Maturation Stage: *P* = 0.7446). Interestingly, 5:2 diet fed female loxTB‐GHSR mice did have a significantly higher number of intermediate stage DCX^+^ cells compared to *ad libitum* fed mice (*P* = 0.0228) (Fig 3G), but not proliferating (*P* = 0.4377) or post‐mitotic (0.2721) morphological stages.

Compared to adolescent mice, in which post‐mitotic cells were most abundant (Fig 3G), adult mice (Fig 3H) displayed a notable shift towards a predominantly proliferative phenotype. There was no evidence for an effect of the 5:2 diet on DCX maturation in male C57BL/6J (Diet: *P* = 0.3890; Diet × Maturation Stage: *P* = 0.7158) and both female C57BL/6J (Diet: *P* = 0.9412; Diet × Maturation Stage: *P* = 0.4978) and loxTB‐GHSR (Diet: *P* = 0.5232; Diet × Maturation Stage: *P* = 0.6423) mice. A significant interaction between diet and maturation stage (*P* = 0.0254) was identified in the male loxTB‐GHSR mice; however, there were no significant post hoc differences between *ad libitum* and 5:2 diets (Proliferative: *P* = 0.1625; Intermediate: *P* = 0.6478; Post‐mitotic: *P* = 0.9985).

### The number of new adult‐born cells in the hippocampus was not altered by the 5:2 diet

**Figure 4:**
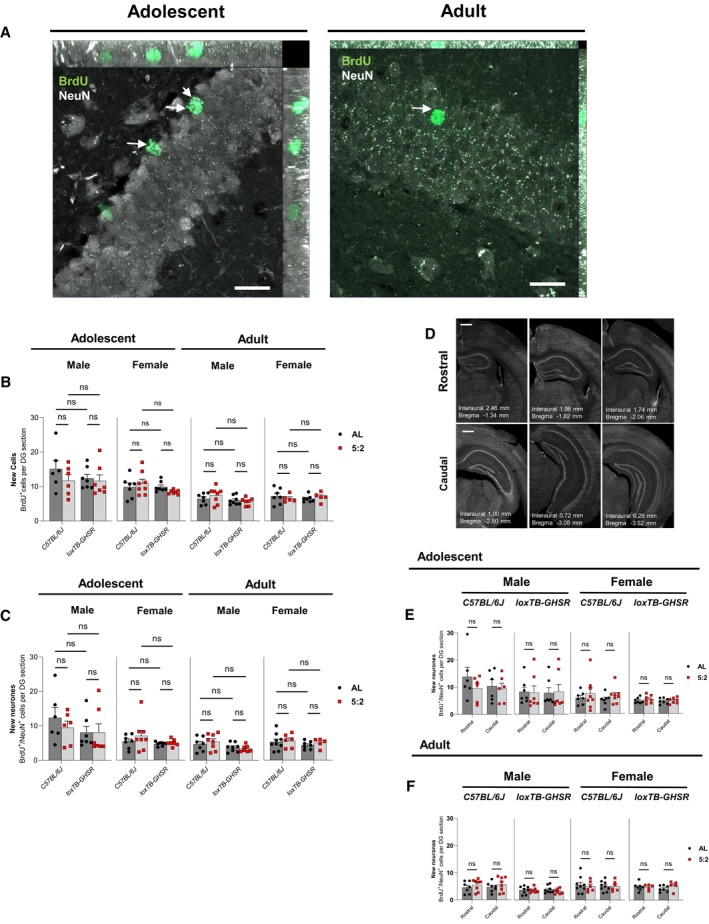
5:2 diet does not affect the generation of new cells or new neurones in the DG Representative maximum intensity projections (MIPs) of BrdU (488 nm) and NeuN (568 nm) immunofluorescence in the DG of adolescent and adult male C57BL/6J *ad libitum* fed mice. Images were acquired with an LSM980‐Airyscan2 confocal system (Zeiss), using the SR‐4Y airyscan mode. Scale bars represent 20 μm. Arrows in the adolescent rep image indicate BrdU^+^/NeuN^+^ cells, while the arrow in the adult rep image shows a BrdU^+^/NeuN^−^ cell.Quantification of total BrdU^+^ cells per DG section in adolescent and adult mice. Cells were manually counted. The effect of diet (*ad libitum* vs. 5:2 diet) was assessed in the presence and absence of functional ghrelin receptor (GHSR) expression (*C57BL/6J* vs. *loxTB‐GHSR*) for both males and females of both age groups.Quantification of BrdU^+^/NeuN^+^ cells, indicative of new neurones, per DG section in adolescent and adult mice (methodology same as B).Representative NeuN immunoreactivity across the hippocampal rostral‐caudal axis. Scale bars represent 500 μm.Quantification of BrdU^+^/NeuN^+^ cells in the rostral and caudal DG of adolescent mice. The effect of diet (*ad libitum* vs. 5:2 diet) was assessed independently for both sexes and both genotypes.Quantification of BrdU^+^/NeuN^+^ cells in the rostral and caudal DG of adult mice (same methodology as E). Data Information: Symbols represent individual mice, bars represent mean values, error bars represent SEM. Statistical comparisons made using ordinary 2‐way ANOVA for (B) and (C) and RM 2‐way ANOVA for (E) and (F). Šídák's multiple comparisons used for *post hoc* assessments. ns *P* ≥ 0.05. *n* = 5–9 mice per group. Source data are available online for this figure.

Next, we explored the effect of the 5:2 diet on the generation of new adult‐born cells in the hippocampal DG in both C57BL/6J and loxTB‐GHSR mice, from adolescent and adult populations. To do this, a BrdU‐pulse chase method was used with all mice receiving twice‐daily injections of BrdU on the first 2 days of the study and BrdU^+^ immunoreactive cells in the sub‐granular zone (SGZ) and granule cell layer (GCL) were counted 7 weeks later (Fig 4A and B; Appendix Table S2).

In contrast to reported findings for ADF and CR regimens (Dias *et al*, 2021), we found no evidence that the 5:2 diet significantly increases the number of BrdU^+^ cells in DG (Fig 4B). This was evident for both adolescent (Male: *P* = 0.2682; Female: *P* = 0.7978) and adult (Male: *P* = 0.6027; Female: *P* = 0.8237) mice. Additionally, we did not find any significant genotype effects on BrdU^+^ cell number in either adolescent (Male: *P* = 0.4394; Female: *P* = 0.1944) or adult (Male: *P* = 0.0685; Female: *P* = 0.5713) age groups, suggesting that GHSR signalling does not enhance new cell generation in the DG.

### The 5:2 diet did not alter the number of new adult‐born neurones (BrdU ^+^/NeuN ^+^) in the hippocampal DG

To determine the fate of the new BrdU^+^ cells, we explored the effect of the 5:2 diet compared to AL feeding on the generation of adult‐born hippocampal neurones (BrdU^+^/NeuN^+^) (Fig 4A and C; Appendix Table S2) across the entire rostro‐caudal extent of the hippocampus. As with the total BrdU^+^ cell counts, we found no evidence for an effect of the 5:2 diet on the number of BrdU^+^/NeuN^+^ cells in either adolescent (Male: *P* = 0.5482; Female: *P* = 0.3272) or adult mice (Male: *P* = 0.6383; *P* = 0.6072).

Interestingly, adult male loxTB‐GHSR mice had significantly fewer BrdU^+^/NeuN^+^ cells compared with adult male C57BL/6J mice (main genotype effect: *P* = 0.0257), Such genotype effects were not observed for adult females (*P* = 0.3370) or adolescent mice of either sex (Male: *P* = 0.2368; Female: *P* = 0.1690).

Given the distinct roles of new adult‐born neurones in the rostral and caudal poles of the DG (Anacker *et al*, 2018), BrdU^+^/NeuN^+^ cells were quantified according to their location along the hippocampal rostro‐caudal axis (Fig 4D). As with our findings for the entire DG, there were no significant effects for the 5:2 diet on the number of BrdU^+^/NeuN^+^ cells at either the rostral or caudal poles (Fig 4E and F; Appendix Table S4). This was evident for each genotype, sex and age group.

**Figure EV2:**
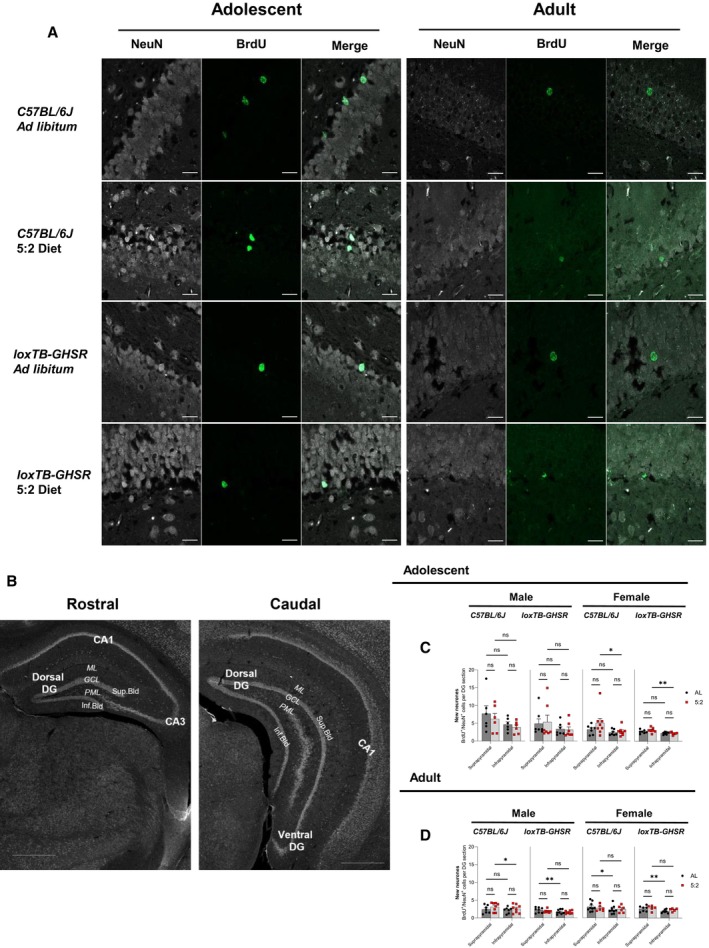
Further characterisation of BrdU^+^/NeuN^+^ cell counts (Related to Fig 4) Representative multichannel images of BrdU (488 nm) and NeuN (568 nm) immunofluorescence in the DG of adolescent and adult male C57BL/6J mice. Images were acquired with an LSM980‐Airyscan2 confocal system (Zeiss), using the SR‐4Y airyscan mode. Scale bars represent 20 μm. It should be noted that the top row images (*ad libitum* fed animals) depict the same cells shown in the maximum intensity projections of Fig 4A.Annotated microscopy images of selected rostral and caudal hippocampal sections from Fig 4D. Images have been re‐used to clarify the anatomical labelling used for the rostral and caudal dentate gyrus (DG) in this study. The three layers of the DG, the molecular layer (ML), granule cell layer (GCL) and polymorphic layer (PML), as well as the suprapyramidal (Sup.bld) and infrapyramidal (Inf.bld) blades of the GCL are highlighted in both images. The DG is entirely dorsal in the rostral image, whereas both dorsal and ventral portions of the DG are evident in the caudal image. In addition to the DG, both CA1 and CA3 hippocampal subfields are present in the rostral image, but only the CA1 is present in the representative caudal image. Scale bars represent 500 μm.Quantification of BrdU^+^/NeuN^+^ cells in the suprapyramidal and infrapyramidal blades of the DG in adolescent mice.Quantification of BrdU^+^/NeuN^+^ cells in the suprapyramidal and infrapyramidal blades of the DG in adult mice. Data Information: Symbols represent individual mice, bars represent mean values, error bars represent SEM. Statistical comparisons made using RM 2‐way ANOVA with Šídák's multiple comparisons *post hoc* assessments. ns *P* ≥ 0.05; *P ≤ 0.05; **P ≤ 0.01. *n* = 5–9 mice per group. Source data are available online for this figure.

To provide further insight into the spatial organisation of new adult‐born neurones, BrdU^+^/NeuN^+^ cells were quantified in the suprapyramidal and infrapyramidal blades of the GCL (Fig EV2B). Across the entire rostral‐caudal axis, BrdU^+^/NeuN^+^ cells were generally more abundant in the suprapyramidal blade of the GCL than the infrapyramidal blade, with significant main effects for GCL blade differences being identified in each RM two‐way ANOVA performed on combined rostral‐caudal data (Fig EV2C and D; Appendix Table S5). Such differences were also evident when data from rostral and caudal poles were analysed separately, although there was an interesting lack of GCL blade effect in the rostral pole of adult male C57BL/6J mice (*P* = 0.0816) (Appendix Table S5). Nevertheless, we found no significant effects for dietary regimen across our analyses of the supra‐ and infrapyramidal blades (Appendix Table S5).

### The 5:2 diet did not increase the number of new neural stem cells (NSCs) in the hippocampal DG

**Figure 5:**
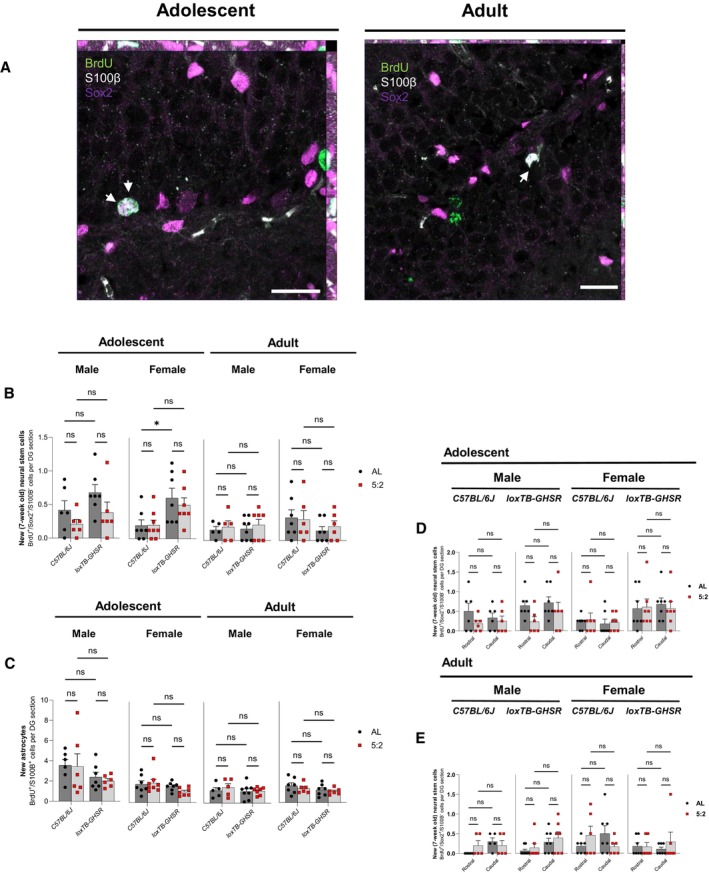
5:2 diet does not significantly affect the number of new (7‐week‐old) neural stem cells (NSCs) or astrocytes in the DG Representative maximum intensity projections (MIPs) of BrdU (488 nm), S100β (568 nm) and Sox2 (647 nm) immunofluorescence in the DG of adolescent and adult male C57BL/6J *ad libitum* fed mice. Images were acquired with an LSM980‐Airyscan2 confocal system (Zeiss), using the SR‐4Y airyscan mode. Scale bars represent 20 μm. Arrows indicate BrdU^+^/S100β^+^/Sox2^+^ cells.Quantification of BrdU^+^/ Sox2^+^/S100β^−^ cells, indicative of new (7‐week‐old) neural stem cells, per DG section in adolescent and adult mice. Cells were manually counted. The effect of diet (*ad libitum* vs. 5:2 diet) was assessed in the presence and absence of functional ghrelin receptor (GHSR) expression (*C57BL/6J* vs. *loxTB‐GHSR*) for both males and females of both age groups.Quantification of BrdU^+^/S100β^+^ cells, indicative of new astrocytes, per DG section in adolescent and adult mice (methodology same as B).Quantification of BrdU^+^/ Sox2^+^/S100β^−^ cells in the rostral and caudal DG of adolescent mice. The effect of diet (*ad libitum* vs. 5:2 diet) was assessed independently for both sexes and both genotypes.Quantification of BrdU^+^/ Sox2^+^/S100β^−^ cells in the rostral and caudal DG of adult mice (same methodology as D). Data Information: Symbols represent individual mice, bars represent mean values, error bars represent SEM. Statistical comparisons made using ordinary 2‐way ANOVA for (B) and (C) and RM 2‐way ANOVA for (E) and (F). Šídák's multiple comparisons used for *post hoc* assessments. ns *P* ≥ 0.05. *n* = 5–8 mice per group. Source data are available online for this figure.

Next, to determine whether the 5:2 diet regulates new 7‐week‐old NSC number in the hippocampal DG, BrdU^+^ immunoreactive cells in the SGZ were assessed for co‐expression with Sox2—a marker of type II NSCs. To allow new NSCs to be distinguished from a subset of new astrocytes that also express Sox2, immunoreactive cells were assessed for co‐expression with the astrocytic marker, S100ß (Fig 5A). New 7‐week‐old NSCs (BrdU^+^/Sox2^+^/S100ß^−^) were counted across the entire rostro‐caudal hippocampal SGZ (Fig 5B; Appendix Table S2).

Adolescent male mice of both genotypes that were fed on the 5:2 dietary paradigm generally had fewer BrdU^+^/Sox2^+^/S100ß^−^ cell counts per DG section compared to age and sex matched *ad libitum* fed mice (Fig 5B). However, these differences did not result in a significant main effect for dietary regimen (*P* = 0.0566), or any significant *post hoc* differences between diets in either C57BL6/J (AL vs. 5:2 *P* = 0.7053) and loxTB‐GHSR (*P* = 0.3473). No notable or significant effects of dietary regimen on NSC number were observed for any of the other groups analysed (Adolescent, Female: *P* = 0.6379; Adult, Male: *P* = 0.4992; Adult, Female: *P* = 0.8517). Thus, the 5:2 dietary regimen does not impact NSC number in our experimental paradigm.

Interestingly, adolescent female loxTB‐GHSR mice had significantly more 7‐week‐old NSCs compared to adolescent female C57BL6/J mice (main genotype effect: *P* = 0.0025), although *post hoc* comparisons were only significant for *ad libitum* fed mice (*P* = 0.0469) and not 5:2 diet fed mice (*P* = 0.1982). In contrast, we found no statistical evidence for an effect of GHSR expression on 7‐week‐old NSCs in adolescent males (*P* = 0.1306), or in adult mice of either sex (Male: *P* = 0.7577; Female: *P* = 0.1557).

To further investigate whether dietary regimen differentially affected NSC renewal in the rostral and caudal poles of the DG, paired rostral and caudal BrdU^+^/Sox2^+^/S100ß^−^ cell counts were analysed via RM Two‐way ANOVAs (Fig 5D and E; Appendix Table S6). Consistent with the findings for the entire rostro‐caudal axis (Fig 5B), there were no main effects of dietary regimen in either adolescent (Male, C57BL/6J: *P* = 0.2446; Male, loxTB‐GHSR: *P* = 0.1383; Female, C57BL/6J: *P* = 0.7113; Female, loxTB‐GHSR: *P* = 0.8637) or adult (Male, C57BL/6J: *P* = 0.6454; Male, loxTB‐GHSR: *P* = 0.3197; Female, C57BL/6J: *P* = 0.8822; Female, loxTB‐GHSR: *P* = 0.5051) mice. Notable differences in the rostral‐caudal NSC counts were observed in adult female C57BL/6J mice, with 5:2 fed mice having higher rostral counts (0.46 ± 0.23 vs. 0.18 ± 0.07) but lower caudal counts (0.17 ± 0.08 vs. 0.50 ± 0.20) on average compared to those fed *ad libitum*. Nevertheless, the interaction between dietary regimen and rostral‐caudal position was not significant (*P* = 0.0591) at the 0.05 significance threshold.

### The 5:2 diet did not increase the number of new adult‐born astrocytes in the hippocampal DG

In addition to new NSCs, we also characterised the generation of new astrocytes (BrdU^+^/S100ß^+^) in the hippocampal DG (Fig 5A and C; Appendix Table S2). As with the other new‐born cell types, adult animals generally had fewer new astrocytes compared to adolescent animals. However, no significant differences were reported in the number of new astrocytes between *ad libitum* and 5:2‐fed mice (Adolescent, Male: *P* = 0.7250; Adolescent, Female: *P* = 0.4853; Adult, Male: *P* = 0.5295; Adult, Female: *P* = 0.3716), or between C57BL/6J and loxTB‐GHSR mice (Adolescent, Male: *P* = 0.0870; Adolescent, Female: *P* = 0.0661; Adult, Male: *P* = 0.4434; Adult, Female: *P* = 0.1330) (Fig 5C). Thus, while the generation of new astrocytes in the hippocampal DG appears to be susceptible to age related decline, it does not appear to be affected by 5:2 diet or loss of GHSR expression.

### Spatial memory performance was not enhanced by 5:2 diet

**Figure 6:**
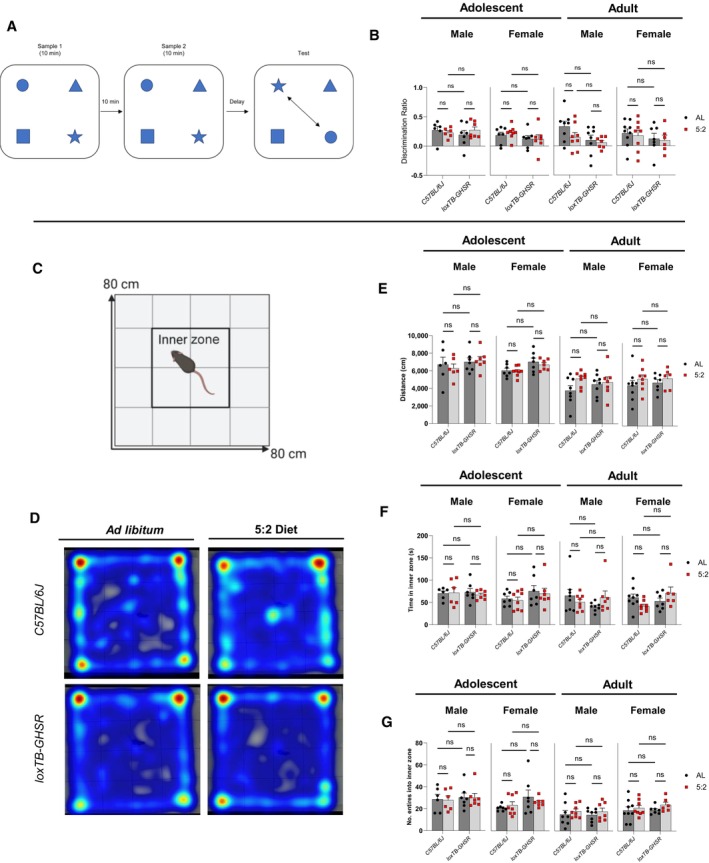
5:2 diet does not significantly affect object in place (OIP) or open field (OF) test performance Schematic illustration of the OIP test.Comparison of OIP discrimination ratios, indicative of spatial discrimination performance. The effect of diet (*ad libitum* vs. 5:2 diet) was assessed in the presence and absence of functional ghrelin receptor (GHSR) expression (*C57BL/6J* vs. *loxTB‐GHSR*) for both males and females of both age groups.Schematic illustration of the OF test arena. Following a 30‐min habituation period, mice were placed into the centre of the 80 × 80 cm test arena and allowed to freely explore for 10 min.Representative heat maps of movement in the OF test arena for adolescent male mice. Heat maps generated from videos of the 10‐min exploratory task using Ethovision XT13 software.Comparison of overall distance (cm) travelled in the OF test arena during the 10‐min exploration (group analysis methodology same as B).Comparison of time (s) spent in the 40 × 40 cm inner zone of the OF test arena during the 10‐min exploration (group analysis methodology same as B).Comparison of inner zone entry frequency during the 10‐min exploration (group analysis methodology same as B). Data Information: Symbols represent individual mice, bars represent mean values, error bars represent SEM. Statistical comparisons made using ordinary 2‐way ANOVA with Šídák's multiple comparisons used for *post hoc* assessments. ns *P* ≥ 0.05. *n* = 6–9 mice per group. Source data are available online for this figure.

To assess whether the 5:2 diet affects hippocampal‐dependent spatial memory performance, we utilised the object in place (OIP) task (Fig 6A), as previously described (Evans *et al*, 2020). Consistent with our BrdU/NeuN data, the 5:2 diet did not improve spatial memory performance (Fig 6B) in either adolescent (Male: *P* = 0.7495; Female: *P* = 0.7349) or adult animals (Male: *P* = 0.1606; Female: *P* = 0.7089), while loss of GHSR expression impaired spatial memory performance in adult males (main genotype effect: *P* = 0.0450) but not in adult females (*P* = 0.3288) or adolescent mice of either sex (Male: *P* = 0.8022; Female: *P* = 0.1479).

Similarly, analysis of locomotor and anxiety‐associated behaviour using the open field test (Fig 6C and D) revealed no significant effects for the 5:2 diet on the overall distance travelled (Fig 6E) (Adolescent, Male: *P* = 0.8272; Adolescent, Female: *P* = 0.4749; Adult, Male: *P* = 0.1176; Adult, Female: *P* = 0.2338), time spent in the inner zone (Fig 6F) (Adolescent, Male: *P* = 0.7805; Adolescent, Female: *P* = 0.6351; Adult, Male: *P* = 0.7588; Adult, Female: *P* = 0.9467) or inner zone entry frequency (Fig 6G) (Adolescent, Male: *P* = 0.9188; Adolescent, Female: *P* = 0.7651; Adult, Male: *P* = 0.2945; Adult, Female: *P* = 0.2119). Interestingly, a significant diet × genotype interaction was observed for time spent in the inner zone with adult female mice (*P* = 0.0352) (Fig 6F). However *post hoc* comparisons did not yield any significant differences between diets (C57BL/6J: *ad libitum* vs. 5:2 diet: *P* = 0.3252; loxTB‐GHSR: *ad libitum* vs. 5:2 diet: *P* = 0.5128) or genotypes (*ad libitum*: C57BL/6J vs. loxTB‐GHSR: *P* = 0.9283; 5:2 diet: C57BL/6J vs. loxTB‐GHSR: *P* = 0.0972). With regards to GHSR expression, we found a significant main genotype effect (*P* = 0.0067) in adolescent females, with loxTB‐GHSR mice travelling further on average in the open field test area compared to C57BL/6J mice. However, neither the duration of time spent in the inner zone (*P* = 0.1116), or inner zone entry frequency (*P* = 0.0933) were significantly affected by loss of GHSR expression in these mice. No significant genotype effects were found in adolescent males or adult mice of either sex.

### Endpoint plasma corticosterone levels were significantly elevated in adolescent female loxTB‐GHSR mice following feeding on 5:2 diet

To further characterise the stress response of our 5:2 dietary paradigm, plasma samples collected at the experimental endpoint were assayed for corticosterone levels. Given that rodent corticosterone levels display circadian and ultradian rhythms (Walker *et al*, 2012; den Boon *et al*, 2019) and that plasma samples were collected at different times throughout the day, we used non‐parametric tests to compare the mean ranks of the data distributions. Interestingly, a significant increase in corticosterone concentration was observed in adolescent female loxTB‐GHSR mice (*P* = 0.009) following 5:2 dietary feeding, but not in age and sex matched C57BL/6J mice (*P* = 0.6389). No other significant effects of 5:2 diet were found in any of the other groups analysed (Fig EV4).

## Discussion

Consistent with previous studies (Kuhn *et al*, 1996; Bondolfi *et al*, 2004) we report the detrimental effect of ageing on the generation of new adult‐born cells in the hippocampal DG. Immature neurone (DCX^+^) and new adult‐born neurone (BrdU^+^/NeuN^+^) production was also significantly decreased in the adult mice in comparison to the adolescent mice (Appendix Fig S1), supporting the notion that age is a key modulator of AHN (Kempermann *et al*, 1998; Kuhn *et al*, 2018). The age‐associated decrease in new neurone production may be attributable to alterations in NSC homeostasis (Ibrayeva *et al*, 2021). Our findings demonstrate an age‐related decline in the number of new 7‐week‐old NSCs in the SGZ across the entire rostro‐caudal axis. This is consistent with a significant decline in the number of new NSCs in the SGZ of the aged rat brain (Hattiangady & Shetty, 2008). We also sought to determine the effect of GHSR signalling on new NSC number. Our previous study reported that exogenous acyl‐ghrelin, administered intraperitoneally at a physiological dose, did not enhance new hippocampal NSC number in 3‐month‐old female rats (Kent *et al*, 2015). Here, we show that new NSC number was increased in adolescent female loxTB‐GHSR mice in comparison to their wild‐type counterparts, with the loss of this effect in adult mice. This increase is unlikely to be a direct effect as GHSR is not expressed in type II (Sox2^+^) NSCs in the SGZ (Hornsby *et al*, 2016). Instead, it may be due to an indirect mechanism yet to be elucidated, whereby the absence of GHSR signalling promotes NSC renewal and/or possibly hinders NSC differentiation. Indeed, we have shown that the absence of GHSR signalling reduces the rate of neuronal differentiation (Hornsby *et al*, 2016). However, despite this increase in new NSCs during adolescence there was no corresponding increase in the number of new adult‐born neurones at this age. These observations collectively suggest that the loss of GHSR signalling leads to a shift in AHN dynamics, whereby the number of cells displaying early‐maturation stage phenotypes is increased and mature neuronal phenotypes are decreased.

Our findings relating to new adult‐born hippocampal cell formation, differentiation and maturation in response to DR, demonstrate that a 7‐week 5:2 diet regime did not promote neurogenesis in the adult mouse hippocampus, nor enhance hippocampal‐dependent spatial memory performance. Similarly, the 5:2 diet did not influence locomotor activity or anxiety‐like behaviour in mice of any age, sex or genotype.

This contrasts with our previous study demonstrating that 2‐weeks of daily 30% CR, followed by 2‐weeks of *ad libitum* feeding, increased AHN (Hornsby *et al*, 2016), suggesting that this approach may be more efficient at enhancing AHN in the short term due to the regime of daily restriction. However, compared to ADF and continuous mild CR, the 5:2‐fed mice in the present study underwent less frequent DR; being subjected to 2 non‐consecutive fasting days every week (Mondays and Thursdays), with 2–3 days of *ad libitum* feeding between each fasting period. Thus, it is possible that the less frequent periods of CR, with lengthened *ad libitum* feeding intervals in between, were insufficient to achieve pro‐neurogenic effects, or that the 6‐week period was an insufficient length of time for the 5:2 dietary regimen to elicit pro‐neurogenic effects. It is also possible that the 5:2 diet led to increased food intake, as observed in male mice in our pilot study, which may counter beneficial effects on neurogenesis.

Previous rodent studies did not use a 5:2 feeding pattern and thus are not directly comparable to the present study. Those studies used more frequent DR periods, with rodents subjected to daily CR (Park *et al*, 2013; Hornsby *et al*, 2016; Apple *et al*, 2019) or variable IF regimens with a maximum of 24 h between fasts (Lee *et al*, 2000, 2002a,b; Kim *et al*, 2015; Baik *et al*, 2020; Dias *et al*, 2021; Cao *et al*, 2022). Indeed, ADF was implicated as a pro‐neurogenic intervention two decades ago, whereby 3 months of ADF increased the survival of BrdU^+^ cells in the adult rodent DG 3–4 weeks after BrdU administration (Lee *et al*, 2000, 2002a,b). Subsequently, several studies have reported that ADF (usually as a 3‐month intervention) stimulates aspects of neurogenesis (Kitamura *et al*, 2005; Kumar *et al*, 2009; Baik *et al*, 2020; Dias *et al*, 2021; Cao *et al*, 2022). However, several of these studies limit their analysis to the proliferative stage (NSPCs and neuroblasts) without assessment of neuronal maturation, survival and functional integration into circuits regulating spatial memory (Kumar *et al*, 2009; Cao *et al*, 2022). Likewise, several studies report data for total BrdU^+^ cells in the DG, but not BrdU^+^/NeuN^+^ cells that are indicative of adult born neurones (Lee *et al*, 2000, 2002a,b; Kitamura *et al*, 2005).

Nevertheless, the recent study of Dias *et al* (2021), which utilised comprehensive IHC characterisations to evaluate the effects of three‐months ADF or 10% daily CR on AHN, supported the earlier conclusion that IF, as well as CR, stimulates AHN. Indeed, both dietary regimens significantly increased total BrdU^+^ cells (24‐h and 4‐weeks post injection), DCX^+^ cells, and the percentage of BrdU^+^/NeuN^+^ cells, compared to *ad libitum* controls. Moreover, the number of BrdU^+^ cells at the 24‐h time point, as well as the number of DCX^+^ cells and the percentage of BrdU^+^/NeuN^+^ cells, was also significantly higher for the ADF group compared to the CR group; leading the authors to conclude that IF is more effective at stimulating AHN than daily 10% CR.

Interestingly, in this issue of EMBO Reports, Gabarro‐Solanas *et al* (2023) questions the suitability of ADF as a pro‐neurogenic intervention, with neither 1‐month nor 3‐months of ADF stimulating AHN. Utilising a tamoxifen inducible transgenic model to trace NSC lineage, ADF did not enhance NSC proliferation, nor the abundance of immature neurones. Moreover, 3 months of ADF failed to increase the number of new adult‐born neurones, while 1 month of ADF significantly reduced the number of new adult‐born neurones compared to *ad libitum* control mice. To account for potentially confounding factors such as genetic strain, they characterised the effect of their ADF regimen in C57BL/6 mice, as well as potential anti‐neurogenic effects arising from tamoxifen administration to report no data supporting ADF as a pro‐neurogenic intervention. Collectively, these findings do not align with other studies on DR and neurogenesis. However, variations in the age and sex of animals used in each of these studies limit the value of direct comparisons. Thus, further comprehensive studies are required to determine the optimal level and duration of DR restriction, as well as optimal frequency for restrictive periods, for the enhancement of AHN and spatial memory.

While the effect of GHSR signalling on NSC renewal remains incompletely understood, our findings, suggest that GHSR signalling enhances AHN by supporting the maturation of new adult‐born neurones. Previous studies showed that AHN is decreased during ageing and dysregulated in neurodegenerative diseases (Kuhn *et al*, 1996; Moreno‐Jimenez *et al*, 2019; Terreros‐Roncal *et al*, 2021). Thus, future work into the mechanisms underlying the pro‐neurogenic effects of GHSR signalling may reveal new therapeutic targets for age‐related decline and neurodegenerative diseases.

In summary, we confirm that GHSR is needed for intact AHN and spatial pattern separation performance in adult male mice. We show for the first time that GHSR regulates new 7‐week‐old NSC formation in the hippocampal neurogenic niche of adolescent female mice. However, our data demonstrate that the 5:2 diet does not increase the formation of new adult born neurones or enhance hippocampal spatial memory performance. These findings suggest that distinct DR regimens differentially regulate neurogenesis in the adult hippocampus and that further studies are required to identify optimal protocols to support cognition during ageing.

## Materials and Methods

All animal work was performed at Cardiff University under the appropriate Home Office Animal Act 1986 approval. Figures 1, 3, 6 and EV1 were created with BioRender.

### Mice

Male and female homozygous loxTB‐GHSR mice and age/sex‐matched wild‐type (C57BL/6J) mice from the same colony (a gift from Professor Jeffrey Zigman, UT Southwestern; Zigman *et al*, 2005) were bred from mice heterozygous for the recombinant lox‐P flanked transcriptional blockade of the *Ghsr* allele. Mice belonged to one of two age groups: adolescent (7 weeks old at the start of the study; *N* = 55), or adult (7‐month‐old; *N* = 61). Mice were housed, using a randomised procedure, at Cardiff University under standard laboratory conditions on 12 h light: 12 h dark cycles (lights on at 06:00 h). For the labelling of proliferating cells, all mice were given twice daily intraperitoneal injection of the thymidine analogue, BrdU (50 mg/kg) on days 1 and 2 of the study.

Mice were fed standard laboratory chow (Harlan Laboratories) and maintained on either *ad libitum* (unrestricted) or 5:2 dietary regimens for 6 weeks. The 5:2 regimen consisted of two non‐consecutive days without food access (Monday and Thursday), and *ad libitum* feeding on the remaining 5 days, with access to food provided at 4 pm, 2 h prior to lights off. Body weight was measured between 9 and 10 am on Mondays, Wednesdays, and Fridays. After this 6‐week period, all mice were subjected to 1 week of *ad libitum* feeding whilst behavioural tests were conducted.

Mice were sacrificed by intracardial perfusion with 4% paraformaldehyde (PFA). Brains were removed and post‐fixed in 4% PFA overnight at 4°C, prior to cryoprotection in 30% sucrose solution. Brains were transported to Swansea University and stored at −80°C. Brains were cut into 30 μm‐thick coronal sections along the rostro‐caudal extent of the hippocampus using a freezing‐stage Microtome (MicroM HM450, ThermoScientific). Sections were collected 1:12 in 0.1% sodium azide in PBS and stored at 4°C until required for immunohistochemistry.

### The effect of the 5:2 diet on food consumption

7‐week‐old male (*m*) and female (*f*) C57BL/6J mice (Charles River) underwent 3 weeks of *ad libitum* (*m*, *n* = 6; *f*, *n* = 6) or 5:2 diet pattern of feeding (*m*, *n* = 6; *f*, *n* = 6). The 5:2 diet pattern of feeding consisted of 2 non‐consecutive 24‐h periods of fasting, where diet was removed at 16:00 h Monday and replaced at 16:00 h Tuesday, and again at 16:00 h Thursday and replaced 16:00 h Friday. The diet used in this study was RM3 (E) (SDS Diets).

Mice were standard group housed (*n* = 3 per box) for 10 days, to acclimatise to their respective feeding patterns. Following this, mice were singly housed, in their respective feeding patterns, in Techniplast mouse metabolic cages with environmental enrichment, where food intake was monitored daily for 14 days.

### Immunohistochemistry

For DAB‐immunohistochemical analysis of Ki67 and DCX labelling, sections were washed in 0.1 M PBS (2 × 10 min) and 0.1 M PBS‐T (1 × 10 min). Subsequently, endogenous peroxidases were quenched by washing in a PBS plus 1.5% H_2_O_2_ solution for 20 min. Sections were washed again (as above) and incubated in 5% Normal Goat Serum (NGS) in PBS‐T for 1 h. Sections were incubated overnight at 4°C with rabbit anti‐Ki67 (1:500, ab16667, Abcam) or guinea pig anti‐DCX (1:15,000 Sigma AB2253), in PBS‐T and 2% NGS solution. Another wash step followed prior to incubation with biotinylated goat anti‐rabbit (1:500 Vectorlabs, USA) for Ki67 or biotinylated goat anti‐guinea pig (1:500 Vectorlabs, USA) for DCX, in PBS‐T for 70 min. The sections were washed and incubated in ABC (Vectorlabs, USA) solution for 90 min in the dark prior to another two washes in PBS, and incubation with 0.1 M sodium acetate pH6 for 10 min. Immunoreactivity was developed in nickel‐enhanced DAB solution followed by two washes in PBS. Sections were mounted onto superfrost^+^ slides (VWR, France) and allowed to dry overnight before being de‐hydrated and de‐lipified in increasing concentrations of ethanol. Finally, sections were incubated in Histoclear (2 × 3 min; National Diagnostics, USA) and coverslipped using Entellan mounting medium (Merck, USA). Slides were allowed to dry overnight prior to imaging.

### Immunofluorescence

All immunofluorescence experiments were carried out on free‐floating sections at room temperature, unless stated otherwise.

#### Double sequential immunofluorescence (BrdU/NeuN)

Four rostral and four caudal brain sections were used for each mouse. Sections were washed three times in 1× Phosphate Buffered Saline (PBS) on a rocker for 5 min, followed by permeabilisation in methanol at −20°C for 2 min, and subsequent washing in PBS as before. For antigen retrieval, sections were treated with 2 M HCl at 37°C for 30 min. This was followed by washing in 0.1 M borate buffer (pH 8.5) for 20 min. Sections were washed in PBS as before, prior to blocking with 5% NGS in PBS plus 0.1% Triton‐X100 (PBS‐T) for 1 h. Excess block was removed, and sections were incubated overnight at 4°C in Rat anti‐BrdU (MCA2060, AbD Serotec) diluted 1:400 in PBS‐T. Sections were washed in PBS as before, and incubated for 1 h in the dark in goat anti‐rat AF488 (A‐11006, Life Technologies, USA) diluted 1:500 in PBS‐T. All washes and incubations following this step were carried out in the dark. Following another series of washes in PBS, sections were treated with mouse anti‐NeuN (MAB377, Millipore, USA) diluted 1:1,000 in PBS‐T and incubated for 1 h. Three further washes in PBS were carried out, followed by incubation for 30 min in Goat anti‐mouse AF568 (A‐11004, Life Technologies, USA) diluted 1:500 in PBS‐T. Sections were washed once in Hoescht nuclear stain diluted 1:5,000 in PBS for 5 min, followed by two additional washes in PBS as before. Sections were mounted onto superfrost^+^ slides with Prolong Gold anti‐fade solution (Life Technologies, USA) equilibrated to room temperature. Coverslips were applied and slides were left covered on the bench for 24 h to allow to dry, prior to storing at 4°C in the dark.

#### Triple immunofluorescence (BrdU/Sox2/S100b)

For BrdU/Sox2/S100β immunofluorescence, two rostral and two caudal brain sections were used from each mouse. Initially, sections were treated as previously described for BrdU/NeuN, with the exception that non‐specific binding sites were first blocked in 5% Normal Donkey Serum (NDS) in PBS‐T for 30 min, and then in 5% NGS for 30 min. In addition, the primary antibodies were applied together in a cocktail that consisted of rat anti‐BrdU (1:400), rabbit anti‐Sox2 (1:500; ab97959, Abcam) and mouse anti‐S100β (1:500; S2532, Sigma) in PBS‐T, and incubated overnight at 4°C. Sections were then washed in PBS as before. This was followed by incubation for 30 min in the dark with the secondary antibody cocktail that consisted of goat anti‐rat AF488, donkey anti‐rabbit AF647 (A‐31573, Life Technologies, USA) and goat anti‐ mouse AF568, all diluted 1:500 in PBS‐T. Sections were washed once in Hoescht nuclear stain in PBS and twice more in PBS and then mounted as previously described.

### Quantification of immunoreactive cells

DAB Immuno‐stained brain tissue was imaged by light microscopy (Nikon 50i) (for DAB), with immunolabelled cells (Ki67, DCX) being quantified using Image J software. For analysis of DCX maturation stage, DCX morphologies (a–f) were determined by manual counting. Tissue sections that did not generate any detectable immunoreactivity were not quantified. This was performed in a blinded manner. BrdU^+^/NeuN^+^ immuno‐stained sections were quantified using a fluorescent microscope (Axioscope, Zeiss). A ×40 objective lens was used to manually count BrdU^+^ immunoreactive cells through the *z*‐axis across the rostro‐caudal extent and both blades of the GCL and SGZ. The SGZ was defined as the area occupied by approximately two cell bodies, above and below, the inferior edge of the GCL.

BrdU^+^ immunolabelled cells were then assessed for co‐expression with the NeuN. For the quantification of new stem cells, BrdU^+^ immunolabelled cells were assessed for co‐expression with Sox2—a marker of type II NSCs. Since Sox2 is also expressed by a subset of hippocampal astrocytes (Komitova & Eriksson, 2004), the astrocytic marker, S100β, was used to differentiate new stem cells (BrdU^+^/Sox2^+^/S100β^−^) from new astrocytes (BrdU^+^/Sox2^−^/S100β^+^/ & BrdU^+^/Sox2^+^/S100β^+^).

Cell counts were divided by the number of DGs analysed to determine the mean number of immunoreactive cells per DG section, in each of the IHC analyses performed. All experimental and quantification steps were performed blinded to the feeding group and genotype of the mice to prevent bias.

**Figure EV3:**
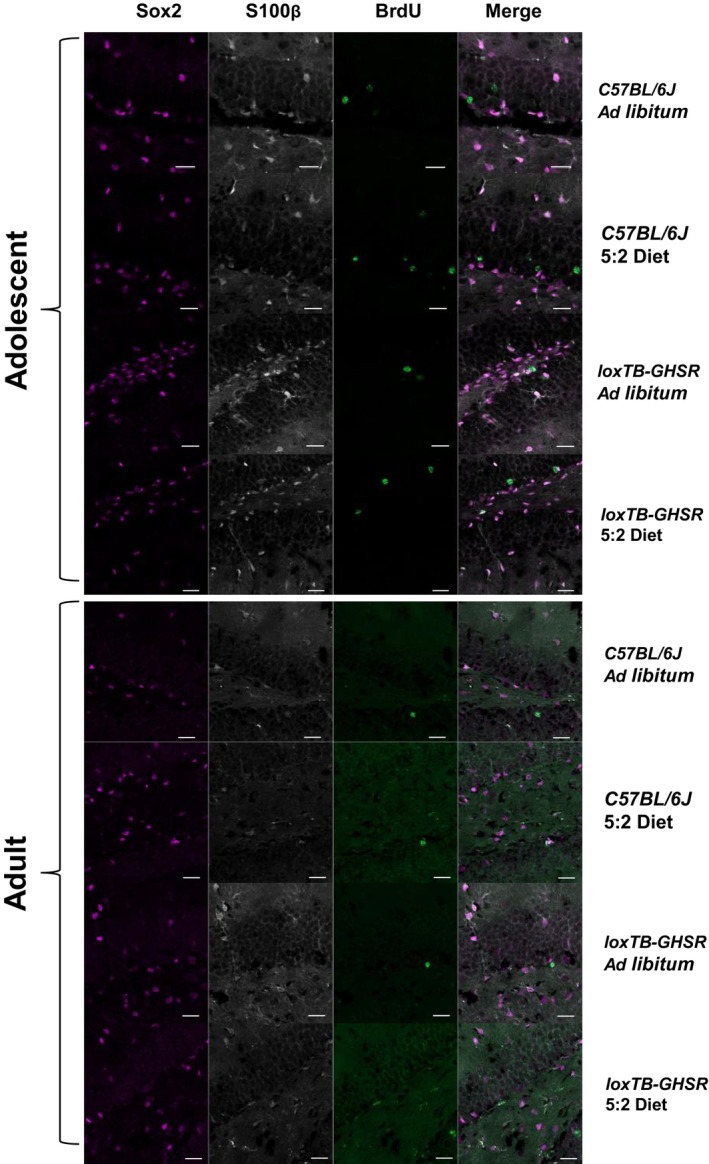
Multichannel microscopy images of BrdU‐S100β‐Sox2 immunofluorescence (related to Fig 5) Images were acquired with an LSM980‐Airyscan2 confocal system (Zeiss), using the SR‐4Y airyscan mode. Scale bars represent 20 μm. BrdU = 488 nm; S100β = 568 nm; Sox2 = 647 nm. Source data are available online for this figure.

Representative fluorescent microscopy images were acquired using an LSM980‐Airyscan 2 confocal microscope (Zeiss) with subsequent processing in Zen Blue (Version 3.8) software. The orthogonal projections presented in Figs 4 and 5, were generated from Z‐stacks acquired in the SR‐4Y Airyscan mode at a slice interval of 0.17 μm, using a 63× 1.40 N/A Plan‐Apochromat oil‐immersion objective. Multichannel images of BrdU^+^/NeuN^+^ immunoreactivity (Fig EV2A) were generated using the same methodology as the orthogonal projections. Multichannel images of BrdU^+^/Sox2^+^/S100β^+^ immunoreactivity (Fig EV3) were acquired in the SR Airyscan acquisition mode using a 20× 0.8 N/A Plan‐Apochromat objective.

**Figure EV4:**
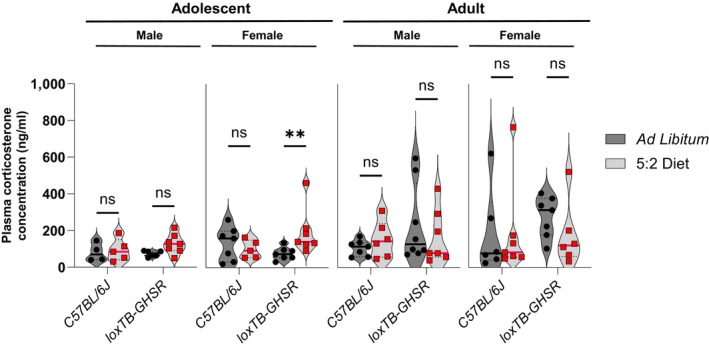
Assessment of plasma corticosterone levels at experimental endpoint Plasma corticosterone concentration was quantified using a colorimetric competitive ELISA (Enzo Lifesciences ADI‐900‐097). Plasma samples were pre‐incubated 1:1 with steroid displacement reagent and diluted to a final dilution of 1:40 with assay buffer, in line with the manufacturers protocol. Data presented as violin plots, with a solid line drawn at the median, dotted lines drawn at the quartiles, and overlaying symbols of individual mice. Non‐parametric comparison of mean ranks was performed using multiple Mann–Whitney tests, with Holm–Šídák correction method to account for multiple comparisons. ns *P* ≤ 0.05; **P ≤ 0.01. Source data are available online for this figure.

### Object in place task

The apparatus used for all experiments was a large Perspex arena, 80 × 80 × 45 cm. The box was placed on a square table at waist height. The apparatus was set up in a quiet and brightly lit (38 cd/m^2^ at the arena surface) behavioural testing room. Exploration was recorded with an overhead camera. The camera input was used to monitor activity in the arena on a laptop and each session was recorded using the USB camera, laptop software and external hard drive.

The duration of object exploration throughout the trials was recorded manually with a stopwatch. All objects used were everyday objects made of non‐porous materials. All objects were at least 10 cm high to avoid the mice climbing and sitting on the objects and were all weighted so that they could not be displaced by the animals. Both the arena and the objects were cleaned thoroughly with 70% ethanol in between each trial to prevent the use of odour cues, urine and excrement were also removed from the arena after each trial.

The main aim of this experiment was to assess whether experimental conditions influenced memory for specific object‐location associations.

The week prior to testing, mice were handled for 5 min a day, three times a week. For 3 days prior to testing, mice were placed in the behavioural test room in their home cages, for 30 min a day. On days 2 and 3, mice were also given habituation session in which to freely explore the arena with no objects present for 10 min. A further habituation session was given on day 3 where mice explored the arena with a single novel object in the centre, for 10 min. Training commenced the following day.

Mice were placed in the centre of the arena and presented with four different objects, each in a different corner of the arena (30 cm diagonally from each corner) during the sample and test trials. Mice were allowed to explore the arena and the objects for 10 min before being removed for a 10‐min interval spent in their home cage (located in the testing room). Mice were then given a second 10‐min sample phase. Following the second sample phase, the mice were returned to their home cage for a 10‐min retention interval (located in the testing room). In all experiments, mice received a 10‐min test phase following this interval.

In the test phase, two of the objects swapped their spatial locations. This resulted in two familiar objects located in different positions, and two familiar objects that remained in their original location. The objects that exchanged their spatial locations were counterbalanced, and the location of the objects in the arena was also counterbalanced to avoid spatial biases.

For each experiment, the dependent variable was the amount of time spent by the animals exploring objects. Object exploration was defined as the time spent attending to (actively sniffing or interacting with) the object at a distance no greater than 1 cm. Object exploration was not scored if the animal was in contact with but not facing the object, or if it attempted to climb on the objects to look around the rest of the arena. In order to ensure that procedures were sensitive to differences between the groups independent of variation in individual contact times, a discrimination ratio was calculated for each experimental test phase. Discrimination ratios were calculated as follows; DR = average time exploring the two objects in new locations/(average time exploring the two objects in the familiar location + average time exploring the two objects in different locations).

### Open field test

Mice were habituated to the dim light test room 30 min prior to the task. The test arena consisted of an 80 × 80 cm arena with 45 cm clear Perspex walls which were sprayed with 70% EtOH and wiped clean prior to each mouse being tested. Mice were placed into the centre of the arena and allowed to freely explore for 10 min.

Videos of this task were recorded with a USB camera and laptop, and these videos were analysed post hoc with Ethovision XT 13 software. The test arena was divided into 2 zones, the “inner zone” comprising of the central 40 × 40 cm portion of the arena and the “outer zone” comprising of the 20 cm area circling the “inner zone”.

### Corticosterone ELISA

Plasma corticosterone levels were measured using a colorimetric competitive ELISA (Enzolifesciences ADI‐900‐097) in accordance with the manufacturers protocol. 10 μl of each plasma sample was diluted 1:1 with 10 μl of steroid displacement reagent (prepared at dilution of 1:100 in PBS), before further dilution with 380 μl of 1x assay buffer to give final dilutions of 1:40. 100 μl of samples and standards were added in duplicate to donkey anti‐sheep IgG coated microtiter plates before the addition of 50 μl alkaline phosphatase‐conjugated corticosterone and then 50 μl of sheep anti‐corticosterone antibody. Plates were then incubated on a plate shaker set to 500 rpm for 2 h at room temperature. Wells were then emptied and washed three times in 1× wash buffer, before the addition of 200 μl of p‐nitrophenyl phosphate (pNPP) substrate. Following a further 1 h incubation (without shaking), 50 μl of stop solution (trisodium phosphate in water) was added to each well, and absorbance was measured at 405 nm (with correction at 570 nm) using a FLUOstar Omega plate reader (BMG Labtech). Corticosterone concentration of diluted plasma samples was interpolated from standard curves using a sigmoidal 4 parameter logistic curve fitting method in GraphPad Prism 10.0.2. Samples with concentrations above the highest standard (20,000 pg/ml) were not interpolated. Following dilution factor correction, concentrations were converted from pg/ml to ng/ml and plotted for statistical analyses.

### Statistical analysis

Statistical analyses were performed using GraphPad Prism 10.0.2. For unpaired data, statistical significance was assessed using two‐way analysis of variance (ANOVA), with Šídák's multiple comparisons test. Statistical significance of paired, matched or repeated measures (RM) data was assessed using RM two‐way ANOVA and Šídák's multiple comparisons test. Residuals were assessed for deviation from a normal distribution using a Shapiro–Wilk test. Statistical significance for plasma corticosterone levels was assessed using multiple non‐parametric Mann–Whitney tests, with a Holm–Šídák correction method to account for multiple comparisons. For all statistical tests, *P < 0.05; **P < 0.01; ***P < 0.001; ****P < 0.0001 were considered significant.

## Author contributions

**Luke D Roberts:** Conceptualization; data curation; formal analysis; funding acquisition; investigation; methodology; writing – original draft; writing – review and editing. **Amanda KE Hornsby:** Data curation; formal analysis; investigation; methodology; project administration; writing – review and editing. **Alanna Thomas:** Formal analysis; investigation; methodology; writing – review and editing. **Martina Sassi:** Formal analysis; investigation; methodology; writing – review and editing. **Aimee Kinzett:** Formal analysis; investigation; writing – review and editing. **Nathan Hsiao:** Formal analysis; investigation; writing – review and editing. **Bethan R David:** Formal analysis; investigation; writing – review and editing. **Mark Good:** Conceptualization; resources; software; supervision; methodology; writing – review and editing. **Timothy Wells:** Conceptualization; data curation; formal analysis; supervision; funding acquisition; investigation; methodology; project administration; writing – review and editing. **Jeffrey S Davies:** Conceptualization; data curation; software; supervision; funding acquisition; investigation; methodology; writing – original draft; project administration; writing – review and editing.

## Disclosure and competing interests statement

The authors declare that they have no conflict of interest.

## Supporting information



Appendix S1

Expanded View Figures PDF

PDF+

Source Data for Expanded View and Appendix

Source Data for Figure 1

Source Data for Figure 2

Source Data for Figure 3

Source Data for Figure 4

Source Data for Figure 5

Source Data for Figure 6

## Data Availability

Our study includes data deposited in public repositories. BioStudies accession number S‐BSST1218 are source data video files of behavioural assays presented in Fig 6A–G.
