# Supplementary material for: The 5:2 diet does not increase adult hippocampal neurogenesis or enhance spatial memory in mice
Source: EMBO Rep. 2023 Nov 21;24(12):e57269. doi: 10.15252/embr.202357269 (PMC10702912; doi:10.15252/embr.202357269)
Supplement: Supplementary file 1 — Appendix S1 [file EMBR-24-e57269-s009.pdf]

Appendix

Table of Contents

Page 1: Table of contents.
Page 2: Appendix Table S1. Summary of endpoint body weight and growth data.
Page 3: Appendix Table S2. Summary of data for cellular markers of adult hippocampal neurogenesis.
Page 4: Appendix Table S3. Summary of data for DCX^+ cell maturation.
Page 5: Appendix Table S4. Summary of data for $BrdU^+/NeuN^+$ cell counts in the rostral and caudal dentate gyrus (DG).
Page 6: Appendix Table S5. Summary of data for $BrdU^+/NeuN^+$ cell counts in the suprapyramidal and infrapyramidal blades of the granule cell layer (GCL) of the DG.
Page 7: Appendix Table S6. Summary of data for $BrdU^+/Sox2^+$ cell counts in the rostral and caudal DG.
Page 8: Appendix Figure S1. Comparison of age effects of cellular markers of AHN.

Appendix Table S1.

Group Information																
Age	Adolescent								Adult							
Sex	Male				Female				Male				Female			
Genotype	*C57BL/6J*		*loxTB-GHSR*		*C57BL/6J*		*loxTB-GHSR*		*C57BL/6J*		*loxTB-GHSR*		*C57BL/6J*		*loxTB-GHSR*	
Diet	Ad libitum	5:2 Diet	Ad libitum	5:2 Diet	Ad libitum	5:2 Diet	Ad libitum	5:2 Diet	Ad libitum	5:2 Diet	Ad libitum	5:2 Diet	Ad libitum	5:2 Diet	Ad libitum	5:2 Diet
n	6	6	7	7	7	8	7	7	8	8	8	7	8	8	7	6
**Body Weight (g)**																
Mean ± SEM	27.47 ± 0.74	26.39 ± 0.63	24.88 ± 0.74	25.22 ± 0.58	22.05 ± 0.58	21.55 ± 0.40	21.30 ± 0.62	20.89 ± 0.50	35.63 ± 1.96	34.67 ± 1.46	31.79 ± 0.79	30.79 ± 0.47	28.18 ± 1.47	28.23 ± 1.25	24.49 ± 0.73	23.76 ± 0.81
Two Way ANOVA																
Genotype				P = 0.0110 (*)				P = 0.1902 (ns)				P = 0.0080 (**)				P = 0.0021 (**)
Diet				P = 0.5895 (ns)				P = 0.4007 (ns)				P = 0.4748 (ns)				P = 0.7742 (ns)
Genotype x Diet				P = 0.3045 (ns)				P = 0.9330 (ns)				P = 0.9876 (ns)				P = 0.7426 (ns)
**Nose-Anus Length (mm)**																
Mean ± SEM	90.00 ± 1.86	88.00 ± 1.44	89.86 ± 1.32	90.00 ± 1.00	87.43 ± 0.84	86.63 ± 1.08	84.57 ± 0.87	84.57 ± 1.46	97.00 ± 1.32	96.63 ± 1.05	94.88 ± 0.83	94.86 ± 0.99	90.38 ± 1.60	92.63 ± 1.21	89.71 ± 1.60	88.17 ± 0.83
Two Way ANOVA																
Genotype				P = 0.5157 (ns)				P = 0.0342 (*)				P = 0.0805 (ns)				P = 0.0810 (ns)
Diet				P = 0.5157 (ns)				P = 0.7170 (ns)				P = 0.8560 (ns)				P = 0.8050 (ns)
Genotype x Diet				P = 0.4540 (ns)				P = 0.7170 (ns)				P = 0.8689 (ns)				P = 0.1894 (ns)
**Tibial Length (mm)**																
Mean ± SEM	17.77 ± 0.13	17.91 ± 0.09	17.62 ± 0.08	17.80 ± 0.15	17.59 ± 0.11	17.65 ± 0.19	17.45 ± 0.11	17.51 ± 0.10	18.46 ± 0.10	18.57 ± 0.13	18.23 ± 0.07	18.24 ± 0.17	18.58 ± 0.11	18.53 ± 0.08	18.16 ± 0.14	18.11 ± 0.14
Two Way ANOVA																
Genotype				P = 0.2920 (ns)				P = 0.3279 (ns)				P = 0.0259 (*)				P = 0.0015 (**)
Diet				P = 0.2023 (ns)				P = 0.6588 (ns)				P = 0.6263 (ns)				P = 0.6593 (ns)
Genotype x Diet				P = 0.8488 (ns)				P = 0.9616 (ns)				P = 0.6526 (ns)				P = 0.9892 (ns)

Appendix Table S1. Summary of endpoint body weight and growth data.

Appendix Table S2.

Group Information								
Age	Adolescent							
Sex	Male				Female			
Genotype	*C57BL/6J*		*loxTB-GHSR*		*C57BL/6J*		*loxTB-GHSR*	
Diet	Ad libitum	5:2 Diet	Ad libitum	5:2 Diet	Ad libitum	5:2 Diet	Ad libitum	5:2 Diet
**Ki67$^+$ cells per DG section**								
Mean ± SEM	22.43 ± 2.07	25.26 ± 3.71	22.19 ± 1.77	23.02 ± 1.35	25.19 ± 3.14	22.73 ± 1.64	24.76 ± 1.85	20.36 ± 0.89
n (mice per group)	6	6	7	7	7	7	7	7
Two Way ANOVA								
Genotype				P = 0.5960 (ns)				P = 0.5030 (ns)
Diet				P = 0.4342 (ns)				P = 0.1070 (ns)
Genotype x Diet				P = 0.6685 (ns)				P = 0.6407 (ns)
**DCX$^+$ cells per DG section**								
Mean ± SEM	138.85 ± 11.62	143.24 ± 18.51	151.77 ± 13.67	152.52 ± 9.04	157.94 ± 7.49	160.32 ± 5.58	149.60 ± 9.59	171.60 ± 3.93
n (mice per group)	6	5	7	7	7	8	7	7
Two Way ANOVA								
Genotype				P = 0.4074 (ns)				P = 0.8334 (ns)
Diet				P = 0.8465 (ns)				P = 0.0896 (ns)
Genotype x Diet				P = 0.8910 (ns)				P = 0.1678 (ns)
**BrdU$^+$ cells per DG section**								
Mean ± SEM	15.11 ± 2.47	11.74 ± 1.76	12.37 ± 1.20	11.64 ± 1.74	9.81 ± 1.16	10.88 ± 1.88	9.89 ± 0.57	8.36 ± 0.23
n (mice per group)	6	6	7	7	7	8	7	7
Two Way ANOVA								
Genotype				P = 0.4394 (ns)				P = 0.1676 (ns)
Diet				P = 0.2682 (ns)				P = 0.1944 (ns)
Genotype x Diet				P = 0.4721 (ns)				P = 0.7978 (ns)
**BrdU$^+$/NeuN$^+$ cells per DG section**								
Mean ± SEM	12.34 ± 2.98	9.47 ± 1.88	8.06 ± 1.79	8.08 ± 2.52	5.46 ± 0.76	7.09 ± 1.60	4.71 ± 0.23	5.04 ± 0.37
n (mice per group)	6	6	7	7	7	8	7	7
Two Way ANOVA								
Genotype				P = 0.2368 (ns)				P = 0.1690 (ns)
Diet				P = 0.5482 (ns)				P = 0.3272 (ns)
Genotype x Diet				P = 0.5434 (ns)				P = 0.5128 (ns)
**BrdU$^+$/Sox2$^+$ cells per DG section**								
Mean ± SEM	0.42 ± 0.14	0.21 ± 0.07	0.68 ± 0.12	0.38 ± 0.16	0.20 ± 0.09	0.20 ± 0.07	0.61 ± 0.15	0.50 ± 0.11
n (mice per group)	6	6	7	6	7	8	7	7
Two Way ANOVA								
Genotype				P = 0.1002 (ns)				P = 0.0025 (**)
Diet				P = 0.0566 (ns)				P = 0.6379 (ns)
Genotype x Diet				P = 0.7211 (ns)				P = 0.5940 (ns)
**BrdU$^+$/S100β$^+$ cells per DG section**								
Mean ± SEM	3.56 ± 0.58	3.44 ± 1.26	2.39 ± 0.48	2.00 ± 0.23	1.82 ± 0.34	1.95 ± 0.37	1.61 ± 0.17	1.07 ± 0.15
n (mice per group)	6	6	7	6	7	8	7	7
Two Way ANOVA								
Genotype				P = 0.0870 (ns)				P = 0.0661 (ns)
Diet				P = 0.7250 (ns)				P = 0.4853 (ns)
Genotype x Diet				P = 0.8554 (ns)				P = 0.2529 (ns)

Group Information								
Age	Adult							
Sex	Male				Female			
Genotype	*C57BL/6J*		*loxTB-GHSR*		*C57BL/6J*		*loxTB-GHSR*	
Diet	Ad libitum	5:2 Diet	Ad libitum	5:2 Diet	Ad libitum	5:2 Diet	Ad libitum	5:2 Diet
**Ki67$^+$ cells per DG section**								
Mean ± SEM	8.95 ± 1.51	10.50 ± 2.04	5.62 ± 0.45	5.90 ± 0.68	8.09 ± 2.04	10.42 ± 2.34	5.77 ± 0.31	8.83 ± 1.56
n (mice per group)	7	8	8	7	8	8	7	6
Two Way ANOVA								
Genotype				P = 0.0072 (**)				P = 0.2979 (ns)
Diet				P = 0.5064 (ns)				P = 0.1543 (ns)
Genotype x Diet				P = 0.6452 (ns)				P = 0.8452 (ns)
**DCX$^+$ cells per DG section**								
Mean ± SEM	24.11 ± 2.58	29.87 ± 5.49	19.57 ± 2.57	24.92 ± 2.17	27.31 ± 2.65	27.60 ± 2.71	22.40 ± 1.43	25.43 ± 4.35
n (mice per group)	6	7	8	8	8	8	6	6
Two Way ANOVA								
Genotype				P = 0.1869 (ns)				P = 0.2427 (ns)
Diet				P = 0.1254 (ns)				P = 0.5800 (ns)
Genotype x Diet				P = 0.9542 (ns)				P = 0.6464 (ns)
**BrdU$^+$ cells per DG section**								
Mean ± SEM	6.45 ± 0.59	7.41 ± 0.82	5.94 ± 0.45	5.62 ± 0.42	7.42 ± 0.67	6.65 ± 0.37	6.45 ± 0.38	6.96 ± 0.61
n (mice per group)	7	8	8	7	9	7	7	5
Two Way ANOVA								
Genotype				P = 0.0685 (ns)				P = 0.5713 (ns)
Diet				P = 0.6027 (ns)				P = 0.8237 (ns)
Genotype x Diet				P = 0.2975 (ns)				P = 0.2709 (ns)
**BrdU$^+$/NeuN$^+$ cells per DG section**								
Mean ± SEM	4.58 ± 0.73	5.58 ± 0.84	3.76 ± 0.46	3.37 ± 0.36	5.35 ± 0.80	5.41 ± 0.72	4.34 ± 0.43	5.03 ± 0.59
n (mice per group)	7	8	8	7	9	7	7	5
Two Way ANOVA								
Genotype				P = 0.0257 (*)				P = 0.3370 (ns)
Diet				P = 0.6383 (ns)				P = 0.6072 (ns)
Genotype x Diet				P = 0.2867 (ns)				P = 0.6634 (ns)
**BrdU$^+$/Sox2$^+$ cells per DG section**								
Mean ± SEM	0.16 ± 0.05	0.20 ± 0.09	0.17 ± 0.06	0.23 ± 0.08	0.34 ± 0.12	0.31 ± 0.13	0.14 ±0.06	0.21 ± 0.08
n (mice per group)	5	5	8	7	7	6	7	6
Two Way ANOVA								
Genotype				P = 0.7577 (ns)				P = 0.1557 (ns)
Diet				P = 0.4992 (ns)				P = 0.8517(ns)
Genotype x Diet				P = 0.9087 (ns)				P = 0.6563 (ns)
**BrdU$^+$/S100β$^+$ cells per DG section**								
Mean ± SEM	1.24 ± 0.30	1.55 ± 0.38	1.16 ± 0.27	1.20 ± 0.14	1.66 ± 0.29	1.40 ± 0.19	1.25 ± 0.22	1.10 ± 0.13
n (mice per group)	5	5	8	7	7	6	7	6
Two Way ANOVA								
Genotype				P = 0.4434 (ns)				P = 0.1330 (ns)
Diet				P = 0.5295 (ns)				P = 0.3716 (ns)
Genotype x Diet				P = 0.6261 (ns)				P = 0.7939 (ns)

Appendix Table S2. Summary of data for cellular markers of adult hippocampal neurogenesis.

Appendix Table S3.

Group Information								
Age	Adolescent							
Sex	Male				Female			
Genotype	*C57BL/6J*		*loxTB-GHSR*		*C57BL/6J*		*loxTB-GHSR*	
Diet	Ad libitum (n = 6)	5:2 Diet (n = 5)	Ad libitum (n = 7)	5:2 Diet (n = 7)	Ad libitum (n = 7)	5:2 Diet (n = 8)	Ad libitum (n = 7)	5:2 Diet (n = 7)
**DCX⁺ cells per DG section**								
Proliferative (Mean ± SEM)	45.72 ± 2.28	43.19 ± 2.61	41.32 ± 3.51	43.95 ± 2.40	41.79 ± 1.96	41.68 ± 1.64	39.64 ± 2.41	43.54 ± 1.03
Intermediate (Mean ± SEM)	28.91 ± 1.21	27.47 ± 2.41	28.73 ± 2.38	28.82 1.36	29.37 ± 1.57	29.14 ± 0.93	26.74 ± 1.82	33.72 ± 0.92
Post-Mitotic (Mean ± SEM)	64.21 ± 12.10	72.59 ± 13.87	81.72 ± 7.91	79.96 ± 8.38	86.77 ± 4.12	89.51 ± 4.44	83.22 ± 5.43	94.34 ± 2.67
RM Two Way ANOVA								
Maturation Stage		P = 0.0027 (**)		P = <0.0001 (****)		P = <0.0001 (****)		P = <0.0001 (****)
Diet		P = 0.8395 (ns)		P = 0.9641 (ns)		P = 0.7991 (ns)		P = 0.0553 (ns)
Maturation Stage x Diet		P = 0.7093 (ns)		P = 0.8588 (ns)		P = 0.7446 (ns)		P = 0.1322 (ns)
Subject		P = 0.2445 (ns)		P = 0.0222 (ns)		P = 0.0087 (**)		P = <0.0001 (****)
**% of total DCX+ cells per DG section**								
Proliferative (Mean ± SEM)	34.47 ± 3.92	31.53 ± 2.79	27.38 ± 0.41	29.37 ± 2.45	26.47 ± 0.32	26.12 ± 1.14	26.52 ± 0.19	25.38 ± 0.20
Intermediate (Mean ± SEM)	21.46 ± 1.89	19.69 ± 1.18	19.10 ± 0.47	19.07 ± 0.86	18.58 ± 0.33	18.21 ± 0.36	17.86 ± 0.22	19.68 ± 0.53
Post-Mitotic (Mean ± SEM)	44.07 ± 5.39	48.79 ± 3.86	53.52 ± 0.77	51.56 ± 3.22	54.95 ± 0.36	55.67 ± 1.41	55.62 ± 0.29	54.95 ± 0.61

Group Information								
Age	Adult							
Sex	Male				Female			
Genotype	*C57BL/6J*		*loxTB-GHSR*		*C57BL/6J*		*loxTB-GHSR*	
Diet	Ad libitum (n = 6)	5:2 Diet (n = 7)	Ad libitum (n = 8)	5:2 Diet (n = 7)	Ad libitum (n = 8)	5:2 Diet (n = 8)	Ad libitum (n = 6)	5:2 Diet (n = 6)
**DCX⁺ cells per DG section**								
Proliferative (Mean ± SEM)	14.57 ± 1.20	17.17 ± 2.09	11.79 ± 1.38	15.71 ± 1.28	16.25 ± 1.31	17.40 ± 1.74	14.04 ± 1.20	15.67 ± 1.72
Intermediate (Mean ± SEM)	6.78 ± 1.05	8.67 ± 1.93	6.17 ± 0.88	7.48 ± 0.82	8.55 ± 1.06	8.49 ± 1.15	6.79 ± 0.32	7.97 ± 1.86
Post-Mitotic (Mean ± SEM)	2.77 ± 0.89	4.03 ± 1.83	1.60 ± 0.52	1.72 ± 0.60	2.51 ± 0.53	1.70 ± 0.28	1.58 ± 0.20	1.89 ± 0.84
RM Two Way ANOVA								
Maturation Stage		P = <0.0001 (****)		P = <0.0001 (****)		P = <0.0001 (****)		P = <0.0001 (****)
Diet		P = 0.3890 (ns)		P = 0.1423 (ns)		P = 0.9412 (ns)		P = 0.5232 (ns)
Maturation Stage x Diet		P = 0.7158 (ns)		P = 0.0254 (*)		P = 0.4978 (ns)		P = 0.6423 (ns)
Subject		P = <0.0001 (****)		P = 0.0007 (***)		P = 0.0023 (**)		P = <0.0001 (****)
**% of total DCX+ cells per DG section**								
Proliferative (Mean ± SEM)	61.87 ± 4.18	60.73 ± 3.47	61.93 ± 2.77	63.69 ± 2.89	60.57 ± 2.45	63.23 ± 2.24	62.22 ± 1.61	64.72 ± 4.67
Intermediate (Mean ± SEM)	27.65 ± 1.80	28.38 ± 0.99	31.13 ± 2.04	29.96 ± 1.42	30.80 ± 1.61	30.50 ± 2.14	30.57 ± 1.20	29.47 ± 2.84
Post-Mitotic (Mean ± SEM)	10.48 ± 3.07	10.90 ± 2.68	6.94 ± 1.78	6.35 ± 1.79	8.63 ± 1.32	6.27 ± 0.98	7.20 ± 1.02	5.81 ± 2.21

Appendix Table S3. Summary of data for DCX^+ cell maturation.

Appendix Table S4.

Group Information	Adolescent							
Sex	**Male**				**Female**			
Genotype	***C57BL/6J***		***loxTB-GHSR***		***C57BL/6J***		***loxTB-GHSR***	
Diet	Ad libitum (n = 6)	5:2 Diet (n = 6)	Ad libitum (n = 7)	5:2 Diet (n = 7)	Ad libitum (n = 7)	5:2 Diet (n = 8)	Ad libitum (n = 7)	5:2 Diet (n = 7)
**BrdU$^+$/NeuN$^+$ cells per DG section**								
Rostral DG (Mean ± SEM)	13.82 ± 3.47	9.61 ± 1.88	8.26 ± 1.74	7.96 ± 2.47	5.79 ± 0.87	7.38 ± 2.08	4.81 ± 0.40	5.31 ± 0.56
Caudal DG (Mean ± SEM)	10.40 ± 2.32	9.44 ± 1.98	7.87 ± 1.90	8.31 ± 2.66	5.13 ± 0.82	6.71 ± 1.08	4.63 ± 0.40	4.79 ± 0.41
RM Two Way ANOVA								
Rostral-Caudal axis		P = 0.1131 (ns)		P = 0.9649 (ns)		P = 0.3662 (ns)		P = 0.4597 (ns)
Diet		P = 0.4613 (ns)		P = 0.9817 (ns)		P = 0.3968 (ns)		P = 0.4605 (ns)
Rostral-Caudal axis x Diet		P = 0.1464 (ns)		P = 0.3862 (ns)		P = 0.9953 (ns)		P = 0.7160 (ns)
Subject		P = 0.0004 (***)		P = <0.0001 (****)		P = 0.0009 (***)		P = 0.5712 (ns)

Group Information	Adult							
Sex	**Male**				**Female**			
Genotype	***C57BL/6J***		***loxTB-GHSR***		***C57BL/6J***		***loxTB-GHSR***	
Diet	Ad libitum (n = 7)	5:2 Diet (n = 8)	Ad libitum (n = 8)	5:2 Diet (n = 7)	Ad libitum (n = 9)	5:2 Diet (n = 6)	Ad libitum (n = 7)	5:2 Diet (n = 5)
**BrdU$^+$/NeuN$^+$ cells per DG section**								
Rostral DG (Mean ± SEM)	4.56 ± 0.81	5.52 ± 0.77	3.63 ± 0.50	3.59 ± 0.37	5.54 ± 1.00	5.02 ± 0.81	4.86 ± 0.56	4.78 ± 0.45
Caudal DG (Mean ± SEM)	4.68 ± 0.68	5.67 ± 0.95	3.89 ± 0.48	3.14 ± 0.37	5.10 ± 0.62	5.16 ± 0.78	4.22 ± 0.42	5.28 ± 0.77
RM Two Way ANOVA								
Rostral-Caudal axis		P = 0.5903 (ns)		P = 0.6784 (ns)		P = 0.6402 (ns)		P = 0.8470 (ns)
Diet		P = 0.4043 (ns)		P = 0.5202 (ns)		P = 0.8470 (ns)		P = 0.5128 (ns)
Rostral-Caudal axis x Diet		P = 0.9442 (ns)		P = 0.1188 (ns)		P = 0.3822 (ns)		P = 0.1267 (ns)
Subject		P = <0.0001 (****)		P = 0.0004 (***)		P = <0.0001 (****)		P = 0.0140 (*)

Appendix Table S4. Summary of data for BrdU$^+$/NeuN$^+$ cell counts in the rostral and caudal dentate gyrus (DG).

Appendix Table S5.

Group Information								
Age	Adolescent							
Sex	Male				Female			
Genotype	*C57BL/6J*		*lox TB-GHSR*		*C57BL/6J*		*lox TB-GHSR*	
Diet	Ad libitum (n = 6)	5:2 Diet (n = 6)	Ad libitum (n = 7)	5:2 Diet (n = 7)	Ad libitum (n = 7)	5:2 Diet (n = 8)	Ad libitum (n = 7)	5:2 Diet (n = 7)
**Rostral DG**								
Suprapyramidal Blade (Mean ± SEM)	8.53 ± 2.74	6.35 ± 1.69	5.05 ± 1.48	5.54 ± 2.05	3.31 ± 0.54	5.67 ± 1.82	2.63 ± 0.20	3.24 ± 0.43
Infrapyramidal Blade (Mean ± SEM)	5.10 ± 0.78	4.08 ± 0.82	3.59 ± 0.69	3.11 ± 0.81	2.70 ± 0.42	2.74 ± 0.59	2.43 ± 0.32	2.25 ± 0.16
RM Two Way ANOVA								
GCL blade		P = 0.0448 (*)		P = 0.0359 (*)		P = 0.0296 (*)		P = 0.0476 (*)
Diet		P = 0.4565 (ns)		P = 0.9994 (ns)		P = 0.3859 (ns)		P = 0.5141 (ns)
GCL blade x Diet		P = 0.6514 (ns)		P = 0.5700 (ns)		P = 0.1342 (ns)		P = 0.1667 (ns)
Subject		P = 0.0633 (ns)		P = 0.0069 (**)		P = 0.0171 (*)		P = 0.2806 (ns)
**Caudal DG**								
Suprapyramidal Blade (Mean ± SEM)	6.49 ± 1.46	6.09 ± 1.47	4.74 ± 1.21	5.20 ± 1.77	3.13 ± 0.54	4.39 ± 0.80	2.76 ± 0.26	2.96 ± 0.25
Infrapyramidal Blade (Mean ± SEM)	4.14 ± 0.88	3.56 ± 0.58	3.12 ± 0.71	3.28 ± 0.85	2.09 ± 0.31	2.64 ± 0.25	2.01 ± 0.23	2.16 ± 0.23
RM Two Way ANOVA								
GCL blade		P = 0.0075 (**)		P = 0.0067 (**)		P = 0.0035 (**)		P = 0.0043 (**)
Diet		P = 0.7462 (ns)		P = 0.8526 (ns)		P = 0.1854 (ns)		P = 0.5396 (ns)
GCL blade x Diet		P = 0.8988 (ns)		P = 0.7897 (ns)		P = 0.3695 (ns)		P = 0.9346 (ns)
Subject		P = 0.0184 (*)		P = 0.0003 (***)		P = 0.0404 (*)		P = 0.2467 (ns)
**Rostral + Caudal DG**								
Suprapyramidal Blade (Mean ± SEM)	7.70 ± 2.20	6.19 ± 1.55	4.91 ± 1.33	5.37 ± 1.91	3.22 ± 0.48	5.03 ± 1.28	2.68 ± 0.18	3.09 ± 0.26
Infrapyramidal Blade (Mean ± SEM)	4.65 ± 0.76	3.84 ± 0.70	3.33 ± 0.67	3.17 ± 0.80	2.40 ± 0.31	2.71 ± 0.41	2.25 ± 0.13	2.21 ± 0.13
RM Two Way ANOVA								
GCL blade		P = 0.0193 (*)		P = 0.0163 (*)		P = 0.0095 (**)		P = 0.0003 (***)
Diet		P = 0.5322 (ns)		P = 0.9362 (ns)		P = 0.2878 (ns)		P = 0.4370 (ns)
GCL blade x Diet		P = 0.7259 (ns)		P = 0.6452 (ns)		P = 0.1708 (ns)		P = 0.1085 (ns)
Subject		P = 0.0333 (*)		P = 0.0018 (**)		P = 0.0166 (*)		P = 0.0319 (*)

Group Information								
Age	Adult							
Sex	Male				Female			
Genotype	*C57BL/6J*		*lox TB-GHSR*		*C57BL/6J*		*lox TB-GHSR*	
Diet	Ad libitum (n = 7)	5:2 Diet (n = 8)	Ad libitum (n = 8)	5:2 Diet (n = 7)	Ad libitum (n = 9)	5:2 Diet (n = 6)	Ad libitum (n = 7)	5:2 Diet (n = 5)
**Rostral DG**								
Suprapyramidal Blade (Mean ± SEM)	2.33 ± 0.48	3.10 ± 0.38	2.08 ± 0.26	1.91 ± 0.22	3.37 ± 0.54	2.71 ± 0.37	2.46 ± 0.24	2.53 ± 0.28
Infrapyramidal Blade (Mean ± SEM)	2.32 ± 0.33	2.51 ± 0.35	1.57 ± 0.25	1.68 ± 0.19	2.34 ± 0.45	2.47 ± 0.46	1.98 ± 0.26	2.25 ± 0.21
RM Two Way ANOVA								
GCL blade		P = 0.0816 (ns)		P = 0.0047 (**)		P = 0.0314 (*)		P = 0.0079 (**)
Diet		P = 0.3763 (ns)		P = 0.9166 (ns)		P = 0.6915 (ns)		P = 0.6426 (ns)
GCL blade x Diet		P = 0.0990 (ns)		P = 0.2258 (ns)		P = 0.1556 (ns)		P = 0.3864 (ns)
Subject		P = <0.0001 (****)		P = 0.0003 (***)		P = 0.0012 (**)		P = 0.0009 (***)
**Caudal DG**								
Suprapyramidal Blade (Mean ± SEM)	2.54 ± 0.40	3.10 ± 0.47	2.30 ± 0.28	1.85 ± 0.16	2.82 ± 0.35	2.78 ± 0.43	2.77 ± 0.39	3.06 ± 0.42
Infrapyramidal Blade (Mean ± SEM)	2.14 ± 0.31	2.61 ± 0.48	1.82 ± 0.21	1.50 ± 0.16	2.28 ± 0.31	2.45 ± 0.37	1.80 ± 0.15	2.38 ± 0.22
RM Two Way ANOVA								
GCL blade		P = 0.0058 (**)		P = 0.0003 (***)		P = 0.0279 (*)		P = 0.0075 (**)
Diet		P = 0.3984 (ns)		P = 0.2144 (ns)		P = 0.8954 (ns)		P = 0.2772 (ns)
GCL blade x Diet		P = 0.7293 (ns)		P = 0.4341 (ns)		P = 0.5691 (ns)		P = 0.5722 (ns)
Subject		P = <0.0001 (****)		P = <0.0001 (****)		P = 0.0004 (***)		P = 0.1046 (ns)
**Rostral + Caudal DG**								
Suprapyramidal Blade (Mean ± SEM)	2.43 ± 0.42	3.13 ± 0.43	2.19 ± 0.26	1.88 ± 0.18	3.11 ± 0.43	2.74 ± 0.35	2.62 ± 0.30	2.79 ± 0.33
Infrapyramidal Blade (Mean ± SEM)	2.20 ± 0.31	2.59 ± 0.42	1.70 ± 0.22	1.59 ± 0.17	2.32 ± 0.37	2.46 ± 0.40	1.89 ± 0.18	2.31 ± 0.20
RM Two Way ANOVA								
GCL blade		P = 0.0066 (**)		P = 0.0002 (***)		P = 0.0105 (*)		P = 0.0011 (**)
Diet		P = 0.3465 (ns)		P = 0.4919 (ns)		P = 0.8347 (ns)		P = 0.4159 (ns)
GCL blade x Diet		P = 0.2173 (ns)		P = 0.2035 (ns)		P = 0.1770 (ns)		P = 0.3737 (ns)
Subject		P = <0.0001 (****)		P = <0.0001 (****)		P = 0.0001 (***)		P = 0.0026 (**)

Appendix Table S5. Summary of data for $BrdU^+$/$NeuN^+$ cell counts in the suprapyramidal and infrapyramidal blades of the granule cell layer (GCL) of the DG.

Appendix Table S6.

Group Information																
Age	Adolescent								Adult							
Sex	Male				Female				Male				Female			
Genotype	*C57BL/6J*		*loxTB-GHSR*		*C57BL/6J*		*loxTB-GHSR*		*C57BL/6J*		*loxTB-GHSR*		*C57BL/6J*		*loxTB-GHSR*	
Diet	Ad libitum (n = 6)	5:2 Diet (n = 6)	Ad libitum (n = 7)	5:2 Diet (n = 6)	Ad libitum (n = 7)	5:2 Diet (n = 7)	Ad libitum (n = 7)	5:2 Diet (n = 7)	Ad libitum (n = 5)	5:2 Diet (n = 5)	Ad libitum (n = 8)	5:2 Diet (n = 7)	Ad libitum (n = 7)	5:2 Diet (n = 6)	Ad libitum (n = 7)	5:2 Diet (n = 6)
**BrdU$^+$ Sox2$^+$ cells per DG section**																
Rostral DG (Mean ± SEM)	0.50 ± 0.20	0.19 ± 0.08	0.64 ± 0.12	0.23 ± 0.12	0.21 ± 0.07	0.29 ± 0.17	0.57 ± 0.19	0.61 ± 0.20	0.00 ± 0.00	0.20 ± 0.12	0.06 ± 0.04	0.14 ± 0.11	0.18 ± 0.07	0.46 ± 0.23	0.18 ± 0.09	0.17 ± 0.11
Caudal DG (Mean ± SEM)	0.30 ± 0.12	0.25 ± 0.13	0.71 ± 0.15	0.50 ± 0.22	0.18 ± 0.12	0.21 ± 0.09	0.68 ± 0.16	0.57 ± 0.18	0.30 ± 0.09	0.20 ± 0.12	0.28 ± 0.10	0.39 ± 0.15	0.50 ± 0.20	0.17 ± 0.08	0.11 ± 0.05	0.29 ± 0.25
RM Two Way ANOVA																
Rostral-Caudal axis		P = 0.6710 (ns)		P = 0.1363 (ns)		P = 0.5282 (ns)		P = 0.8316 (ns)		P = 0.1411 (ns)		P = 0.0619 (ns)		P = 0.9205 (ns)		P = 0.8555 (ns)
Diet		P = 0.2446 (ns)		P = 0.1383 (ns)		P = 0.7113 (ns)		P = 0.8637 (ns)		P = 0.6454 (ns)		P = 0.3197 (ns)		P = 0.8822 (ns)		P = 0.5051 (ns)
Rostral-Caudal axis x Diet		P = 0.3585 (ns)		P = 0.3733 (ns)		P = 0.8322 (ns)		P = 0.6715 (ns)		P = 0.1411 (ns)		P = 0.8937 (ns)		P = 0.0591 (ns)		P = 0.5083 (ns)
Subject		P = 0.1821 (ns)		P = 0.0258 (*)		P = 0.0369 (*)		P = 0.2343 (ns)		P = 0.3612 (ns)		P = 0.7728 (ns)		P = 0.2669 (ns)		P = 0.6711 (ns)

Appendix Table S6. Summary of data for BrdU$^+$/Sox2$^+$ cell counts in the rostral and caudal DG.

Appendix Figure S1.

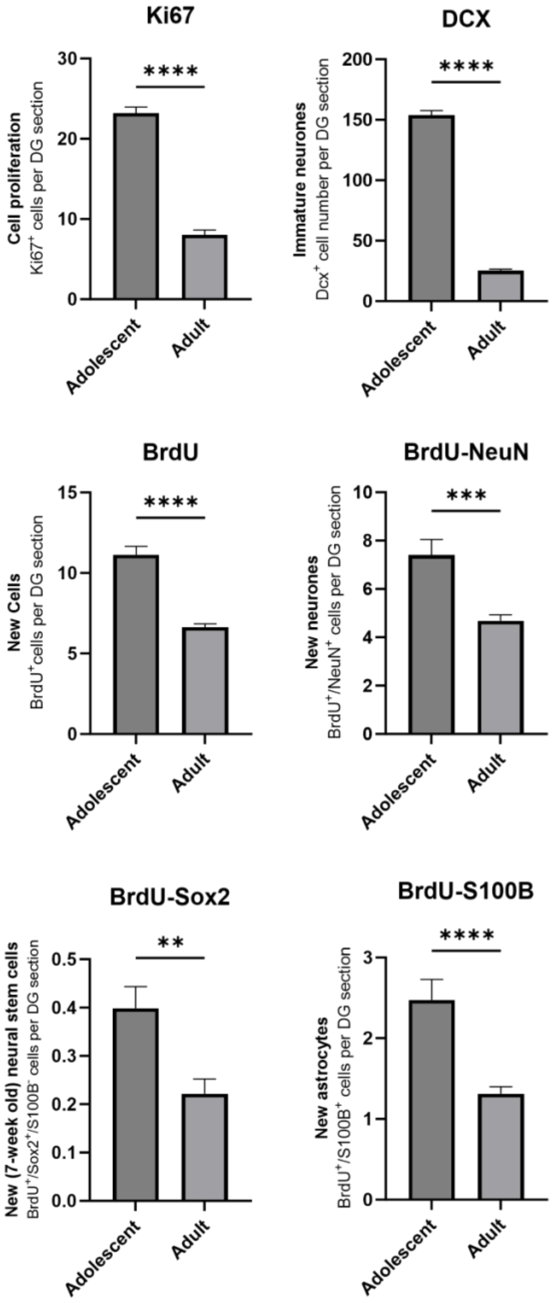

Appendix Figure S1. Comparison of age effects of cellular markers of AHN. Data from all sexes, genotypes and diets were pooled for each age group under study. The effect of age on each marker was assessed using unpaired student t-tests. ** P = < 0.01; *** P = < 0.001; **** P = < 0.0001;
