# Supplementary material for: The 5:2 diet does not increase adult hippocampal neurogenesis or enhance spatial memory in mice
Source: EMBO Rep. 2023 Nov 21;24(12):e57269. doi: 10.15252/embr.202357269 (PMC10702912; doi:10.15252/embr.202357269)
Supplement: Supplementary file 2 — Expanded View Figures PDF [file EMBR-24-e57269-s008.pdf]

Expanded View Figures

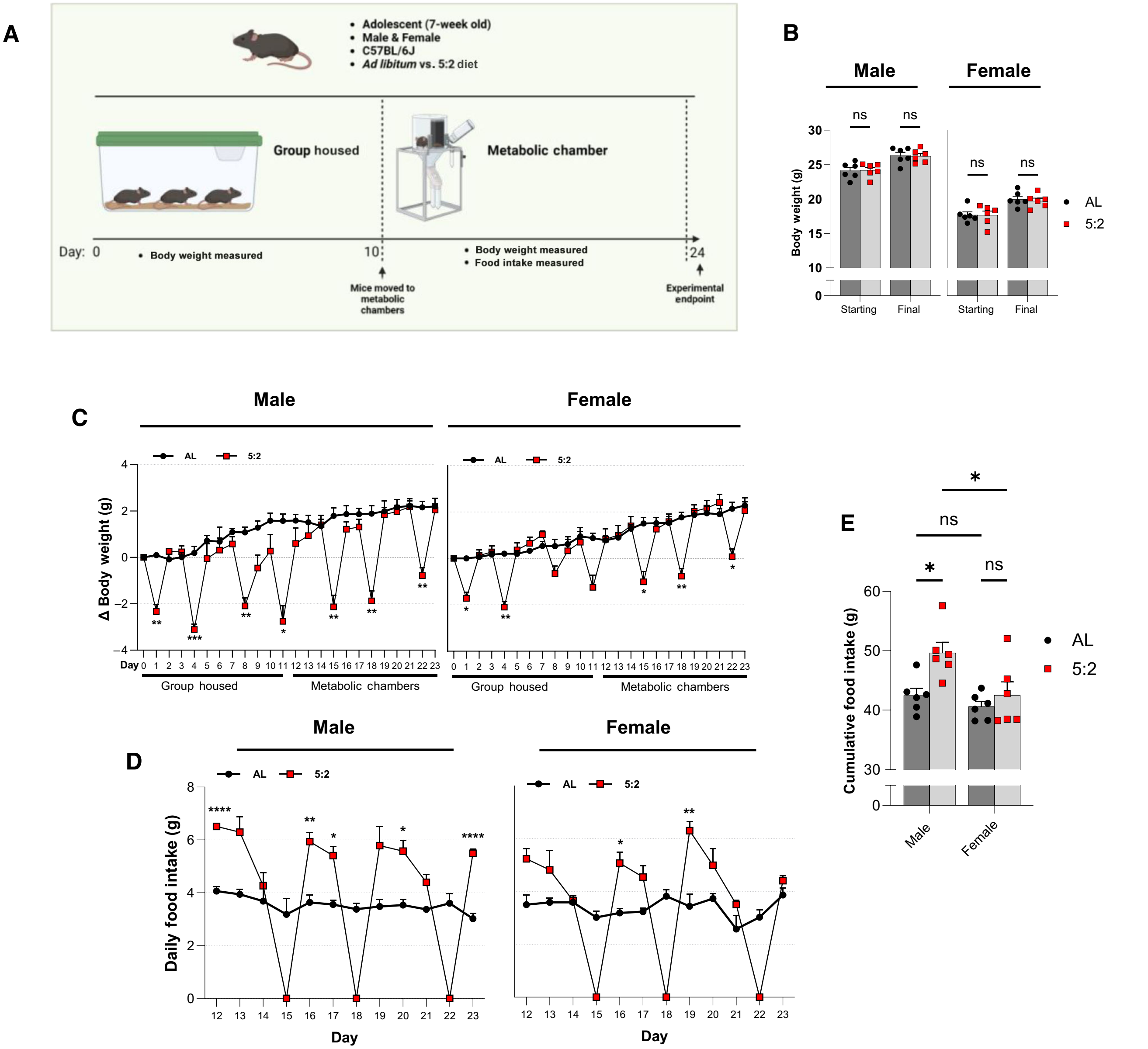

Figure EV1. Association between body weight change and food intake during 5:2 dietary regimen (related to Fig 1).

A Schematic overview of the experimental design. 7-week-old male and female C57BL/6J mice were fed either *ad libitum* or on the 5:2 diet for 24 days. Mice were group housed for the first 10 days of the intervention before being moved to individual metabolic chambers to measure daily food intake in addition to body weight.

B Starting (day 0) and final (day 23) body weights in male (left) and female (right) mice.

C Daily body weight changes (Δ Body Weight) over the experimental time course.

D Daily food intake between days 12 and 23.

E Cumulative food intake at the end of the recording period.

Data Information: For (B) and (E), symbols represent individual mice, bars represent mean values, error bars represent SEM. For (C) and (D), symbols represent mean values, error bars represent SEM. Statistical comparisons made using RM 2-way ANOVA for (B–D) and ordinary 2-way ANOVA for (E). Šídák's multiple comparisons used for post hoc assessments. ns $P \geq 0.05$; *$P \leq 0.05$; **$P \leq 0.01$; ***$P \leq 0.001$; ****$P \leq 0.0001$. $n = 6$ mice per group.
Source data are available online for this figure.

© 2023 The Authors

Figure EV2. Further characterisation of BrdU$^+$/NeuN$^+$ cell counts (Related to Fig 4).

A Representative multichannel images of BrdU (488 nm) and NeuN (568 nm) immunofluorescence in the DG of adolescent and adult male C57BL/6J mice. Images were acquired with an LSM980-Airyscan2 confocal system (Zeiss), using the SR-4Y airyscan mode. Scale bars represent 20 μm. It should be noted that the top row images (*ad libitum* fed animals) depict the same cells shown in the maximum intensity projections of Fig 4A.

B Annotated microscopy images of selected rostral and caudal hippocampal sections from Fig 4D. Images have been re-used to clarify the anatomical labelling used for the rostral and caudal dentate gyrus (DG) in this study. The three layers of the DG, the molecular layer (ML), granule cell layer (GCL) and polymorphic layer (PML), as well as the suprapyramidal (Sup.bld) and infrapyramidal (Inf.bld) blades of the GCL are highlighted in both images. The DG is entirely dorsal in the rostral image, whereas both dorsal and ventral portions of the DG are evident in the caudal image. In addition to the DG, both CA1 and CA3 hippocampal subfields are present in the rostral image, but only the CA1 is present in the representative caudal image. Scale bars represent 500 μm.

C Quantification of BrdU$^+$/NeuN$^+$ cells in the suprapyramidal and infrapyramidal blades of the DG in adolescent mice.

D Quantification of BrdU$^+$/NeuN$^+$ cells in the suprapyramidal and infrapyramidal blades of the DG in adult mice.

Data Information: Symbols represent individual mice, bars represent mean values, error bars represent SEM. Statistical comparisons made using RM 2-way ANOVA with Šídák's multiple comparisons *post hoc* assessments. ns $P \geq 0.05$; *$P \leq 0.05$; **$P \leq 0.01$. n = 5–9 mice per group.
Source data are available online for this figure.

 © 2023 The Authors

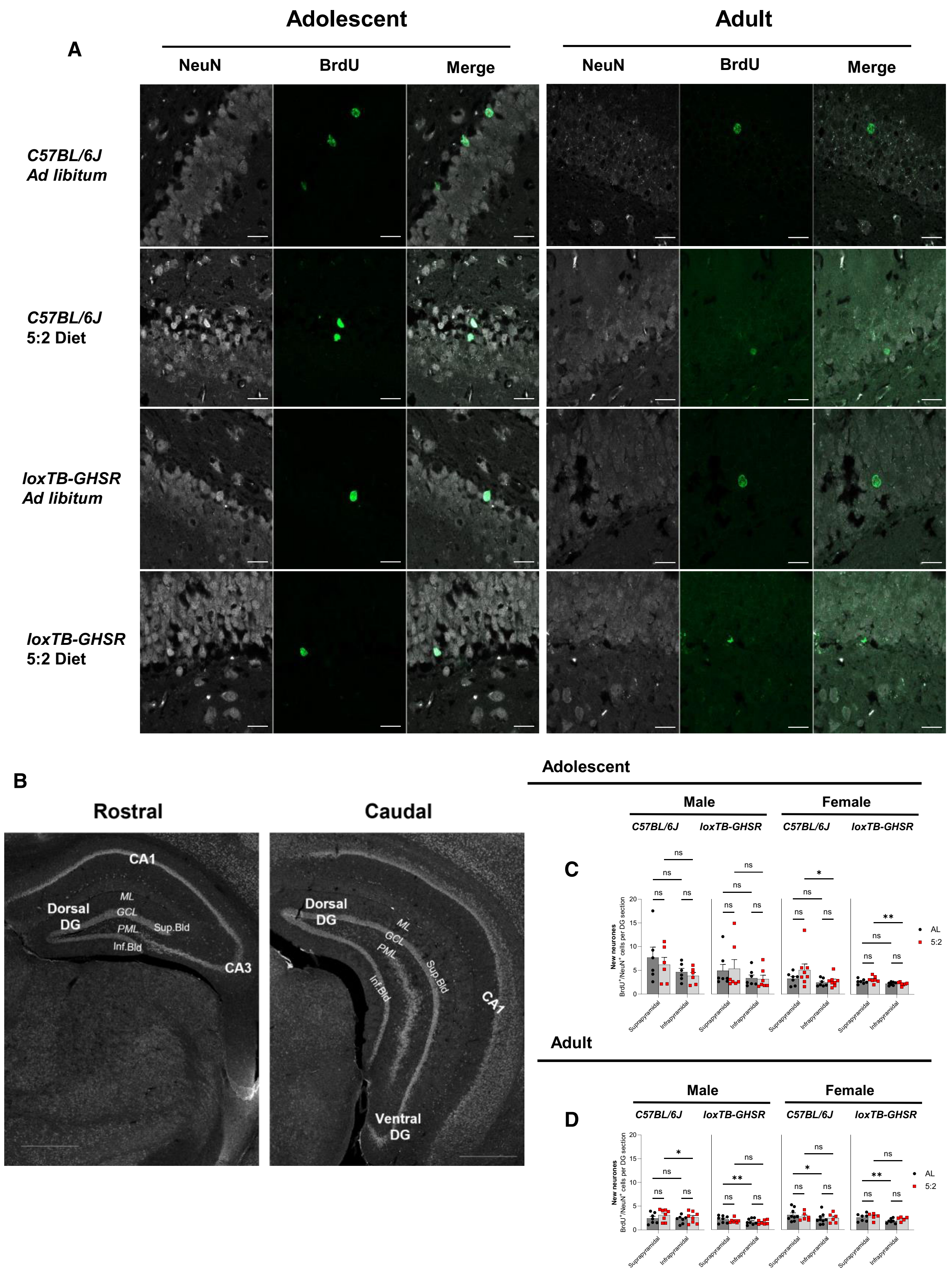

Adolescent
Adult
A
NeuN
BrdU
Merge
C57BL/6J Ad libitum
C57BL/6J 5:2 Diet
loxTB-GHSR Ad libitum
loxTB-GHSR 5:2 Diet
B
Rostral
Caudal
CA1
Dorsal DG
ML
GCL
PML
Sup.Bld
Inf.Bld
CA3
Ventral DG
Male
Female
C57BL/6J
loxTB-GHSR
C
D
New neurones BrdU+/NeuN+ cells per DG section
Suprapyramidal
Infrapyramidal
ns
*
**
AL
5:2

Figure EV2.

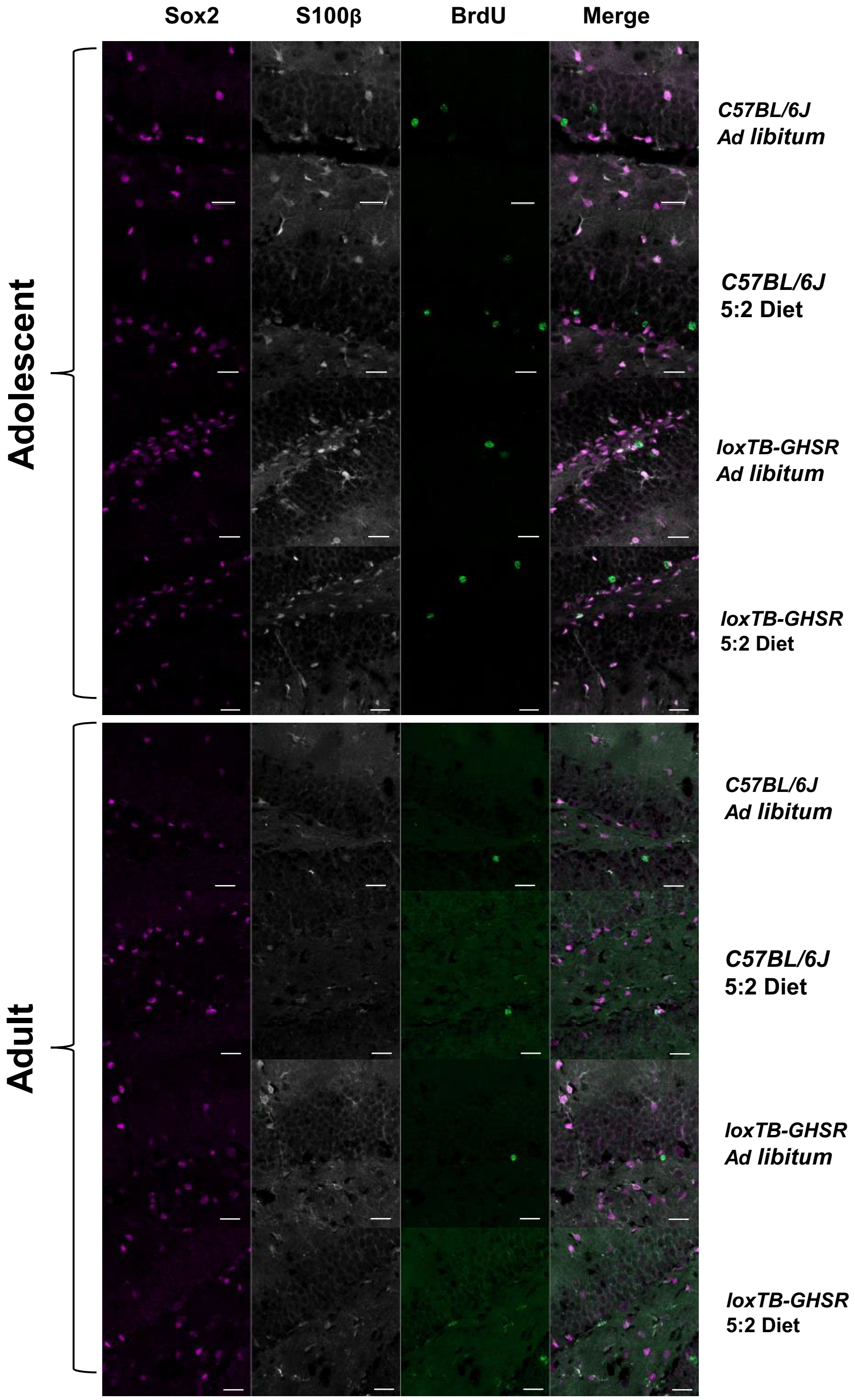

Figure EV3. Multichannel microscopy images of BrdU-S100β-Sox2 immunofluorescence (related to Fig 5).

Images were acquired with an LSM980-Airyscan2 confocal system (Zeiss), using the SR-4Y airyscan mode. Scale bars represent 20 μm. BrdU = 488 nm; S100β = 568 nm; Sox2 = 647 nm.

Source data are available online for this figure.

 © 2023 The Authors

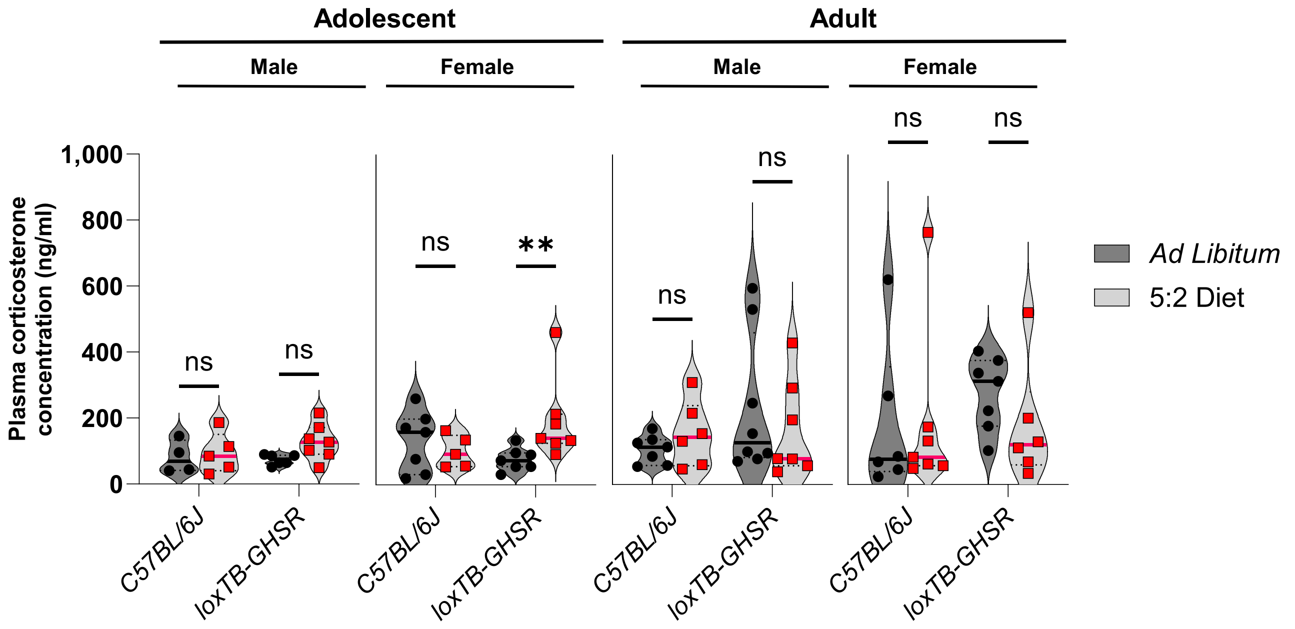

Figure EV4. Assessment of plasma corticosterone levels at experimental endpoint.

Plasma corticosterone concentration was quantified using a colorimetric competitive ELISA (Enzo Lifesciences ADI-900-097). Plasma samples were pre-incubated 1:1 with steroid displacement reagent and diluted to a final dilution of 1:40 with assay buffer, in line with the manufacturers protocol. Data presented as violin plots, with a solid line drawn at the median, dotted lines drawn at the quartiles, and overlaying symbols of individual mice. Non-parametric comparison of mean ranks was performed using multiple Mann–Whitney tests, with Holm–Š4́5dák correction method to account for multiple comparisons. ns $P \leq 0.05$; ** $P \leq 0.01$.

Source data are available online for this figure.
