# Supplementary figures and images for: The 5:2 diet does not increase adult hippocampal neurogenesis or enhance spatial memory in mice
Source: EMBO Rep. 2023 Nov 21;24(12):e57269. doi: 10.15252/embr.202357269 (PMC10702912; doi:10.15252/embr.202357269)

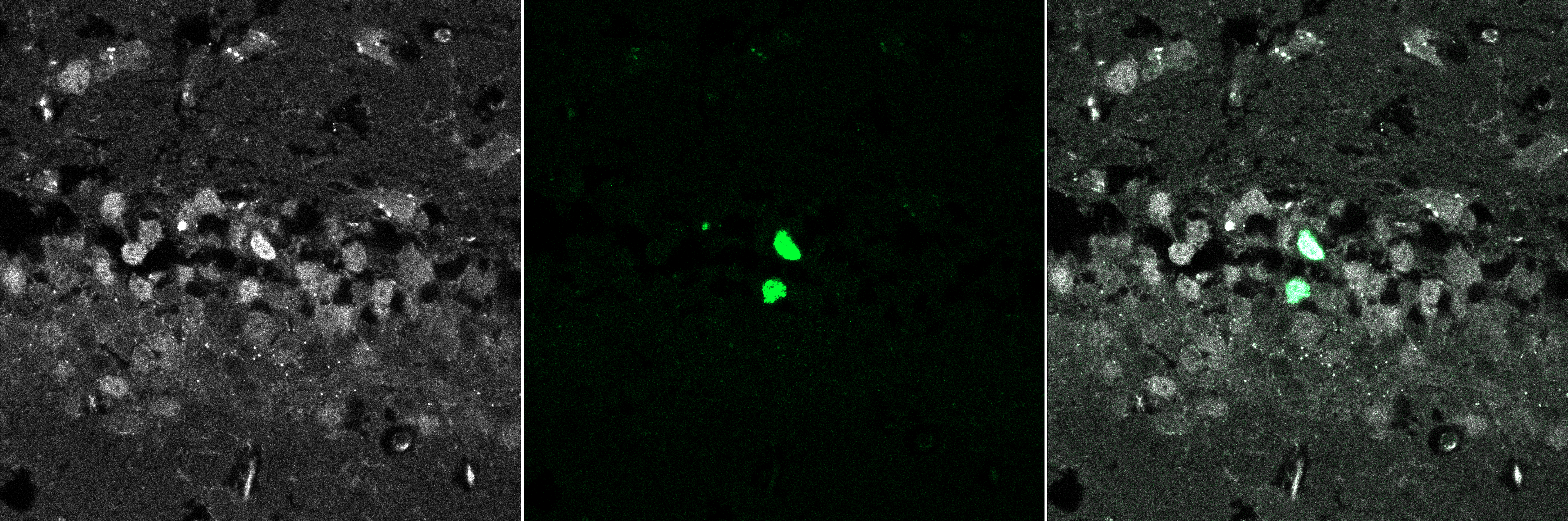

Supplement: Supplementary file 4 — Source Data for Expanded View and Appendix [file EMBR-24-e57269-s001.zip › Figure EV2/Figure EV2/EV2 A/Adolescent. C57BL6J. 5.2 Diet.tif]

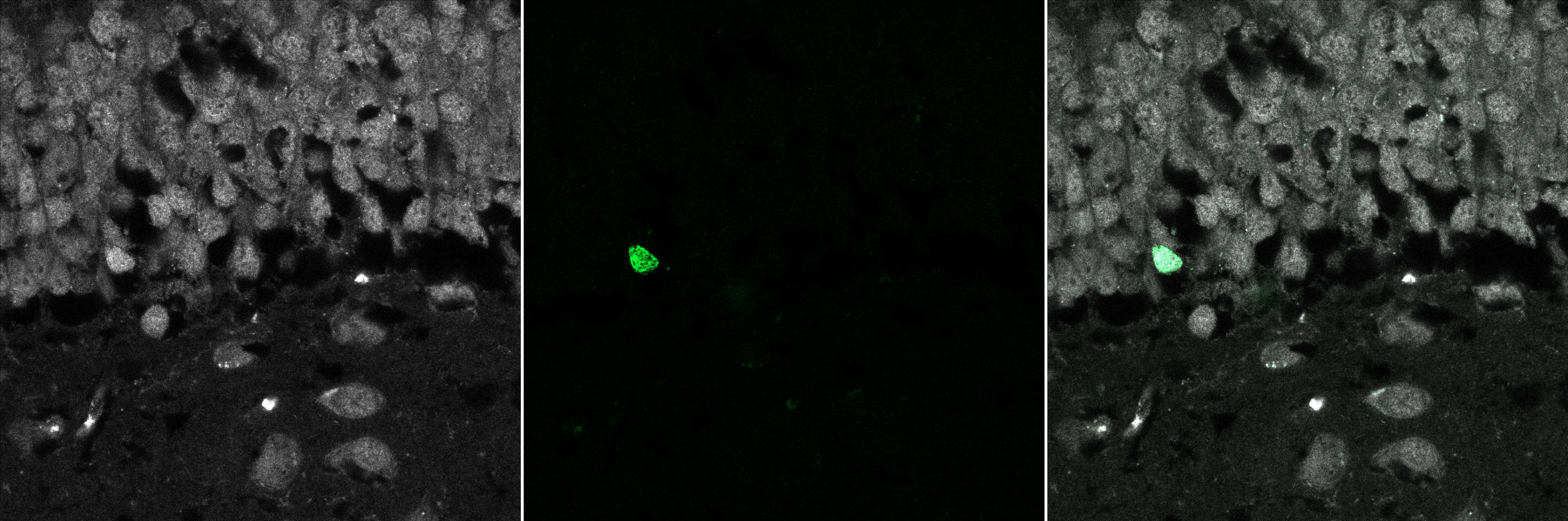

Supplement: Supplementary file 4 — Source Data for Expanded View and Appendix [file EMBR-24-e57269-s001.zip › Figure EV2/Figure EV2/EV2 A/Adolescent. loxTB-GHSR. 5.2 Diet.tif]

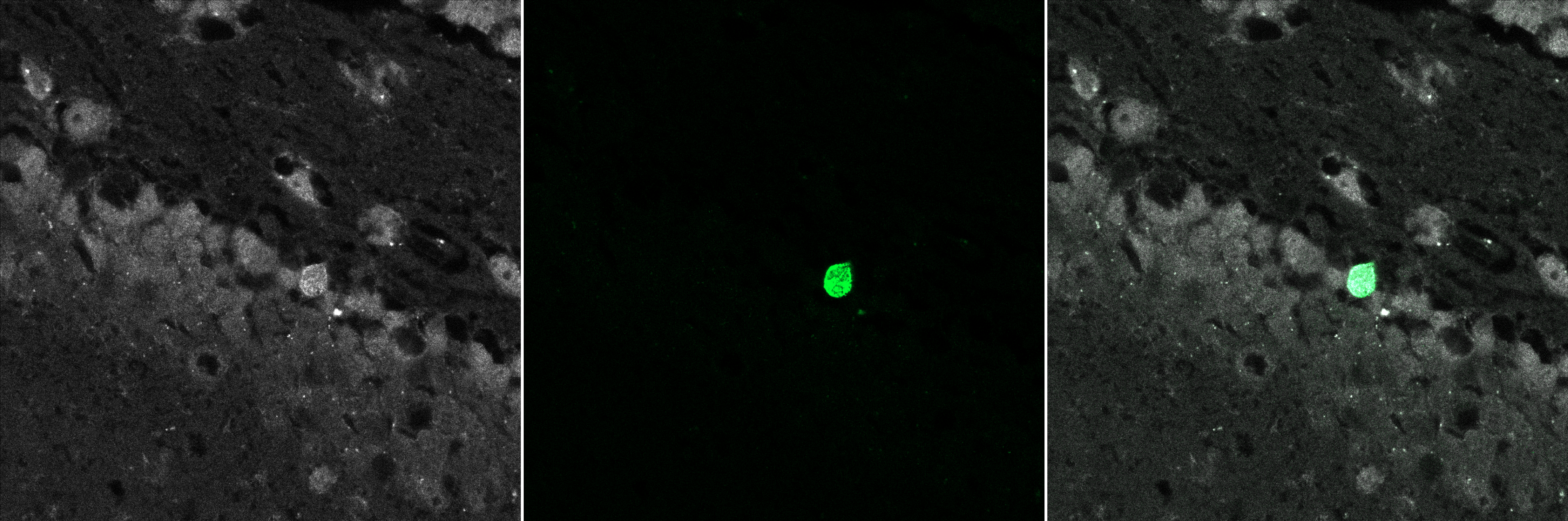

Supplement: Supplementary file 4 — Source Data for Expanded View and Appendix [file EMBR-24-e57269-s001.zip › Figure EV2/Figure EV2/EV2 A/Adolescent. loxTB-GHSR. Ad libitum.tif]

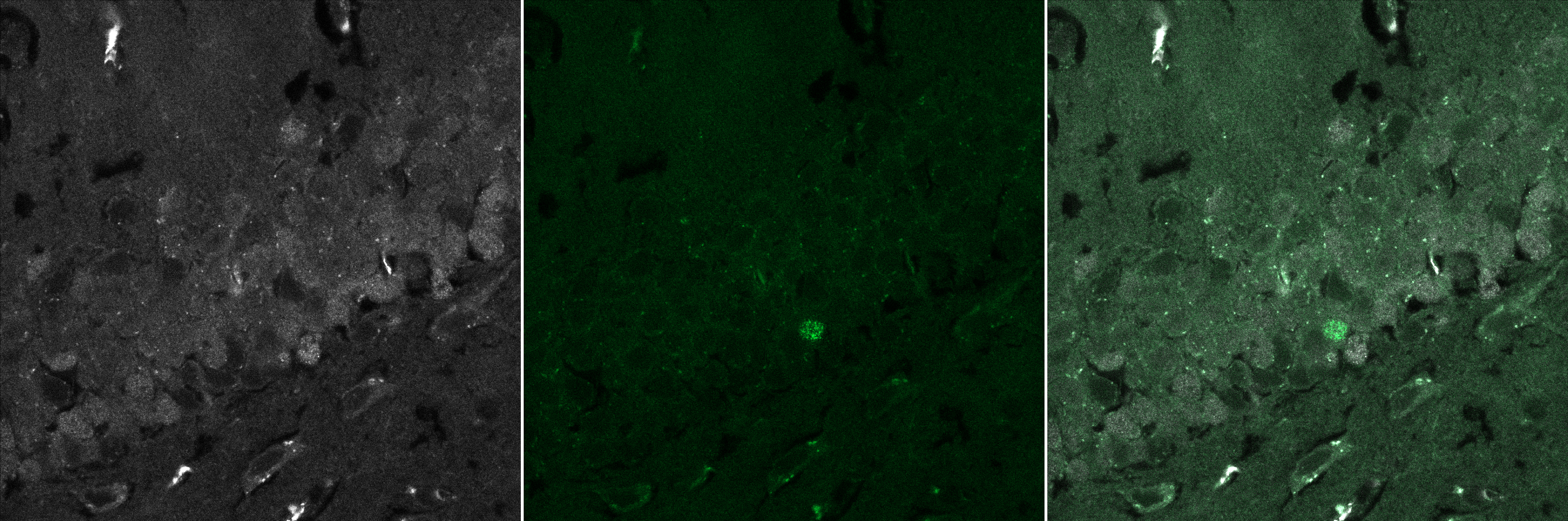

Supplement: Supplementary file 4 — Source Data for Expanded View and Appendix [file EMBR-24-e57269-s001.zip › Figure EV2/Figure EV2/EV2 A/Adult. C57BL6J. 5.2 Diet.tif]

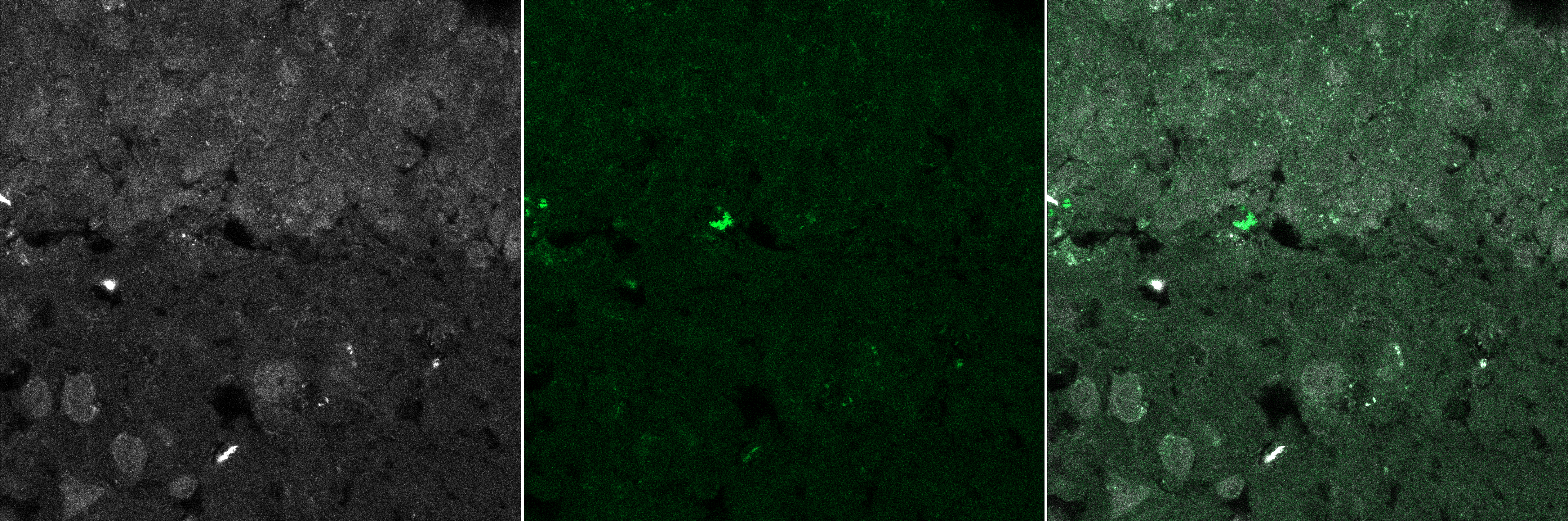

Supplement: Supplementary file 4 — Source Data for Expanded View and Appendix [file EMBR-24-e57269-s001.zip › Figure EV2/Figure EV2/EV2 A/Adult. loxTB-GHSR. 5.2 Diet.tif]

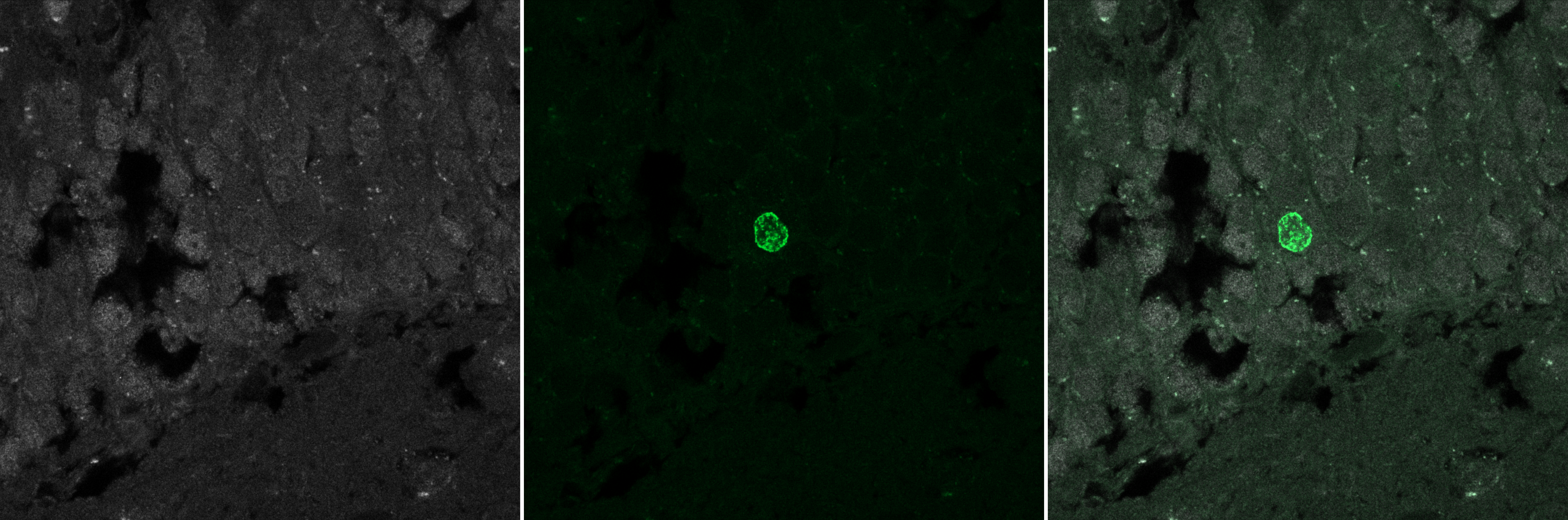

Supplement: Supplementary file 4 — Source Data for Expanded View and Appendix [file EMBR-24-e57269-s001.zip › Figure EV2/Figure EV2/EV2 A/Adult. loxTB-GHSR. Ad libitum.tif]

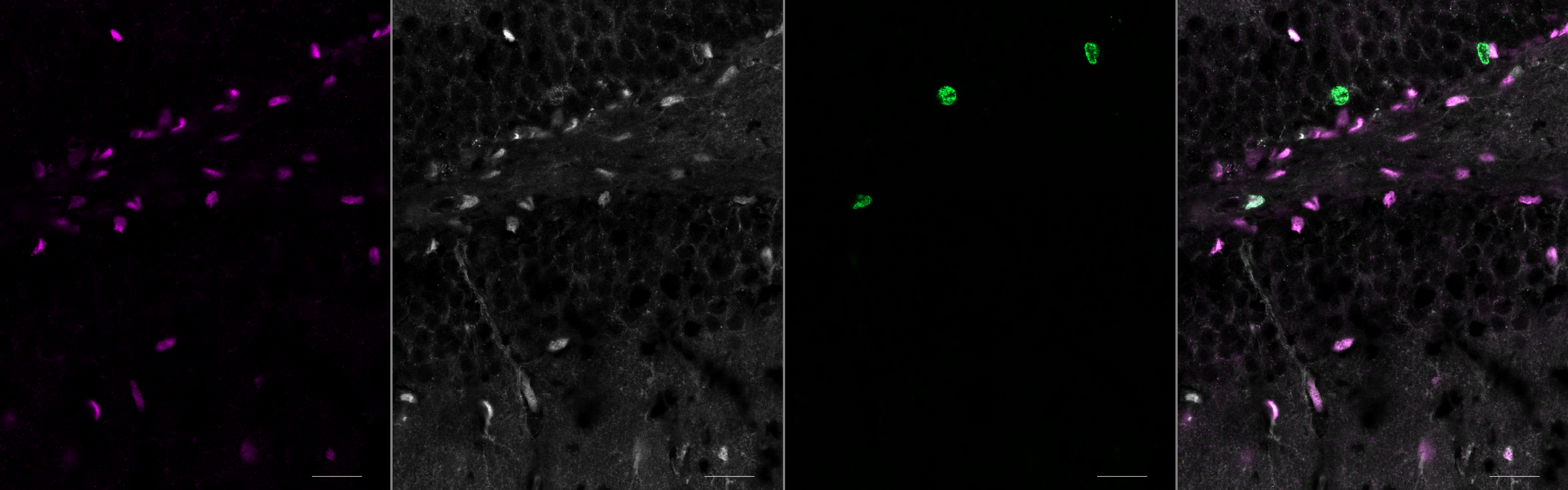

Supplement: Supplementary file 4 — Source Data for Expanded View and Appendix [file EMBR-24-e57269-s001.zip › Figure EV3/Figure EV3/Adolescent loxTB-GHSR. 5.2 diet.tif]

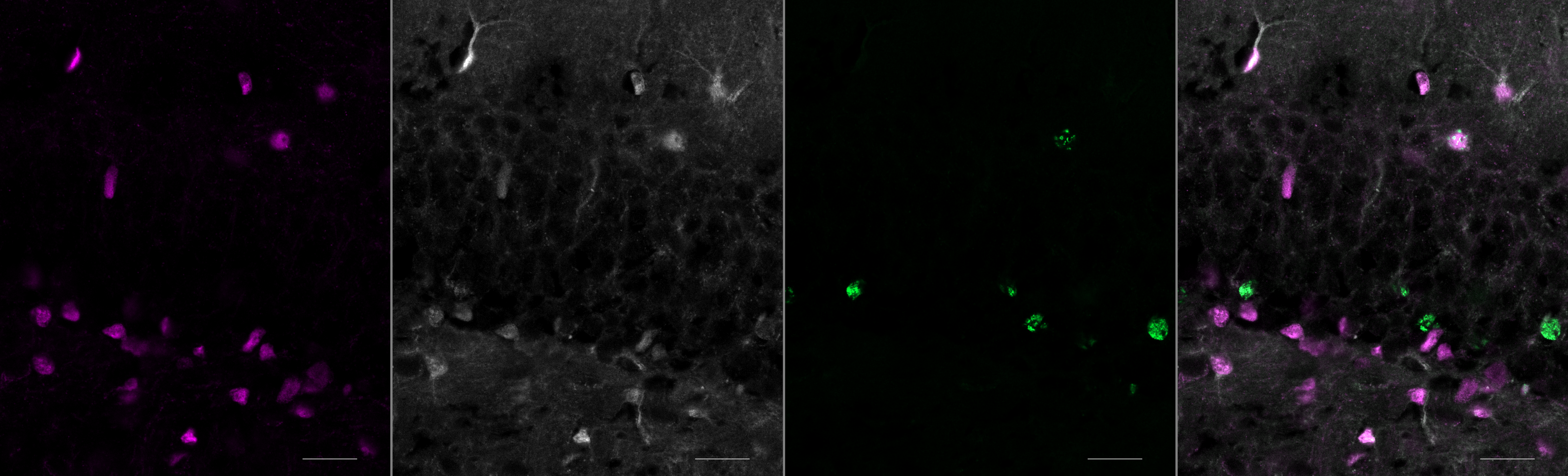

Supplement: Supplementary file 4 — Source Data for Expanded View and Appendix [file EMBR-24-e57269-s001.zip › Figure EV3/Figure EV3/Adolescent. C57BL6J. 5.2 Diet.tif]

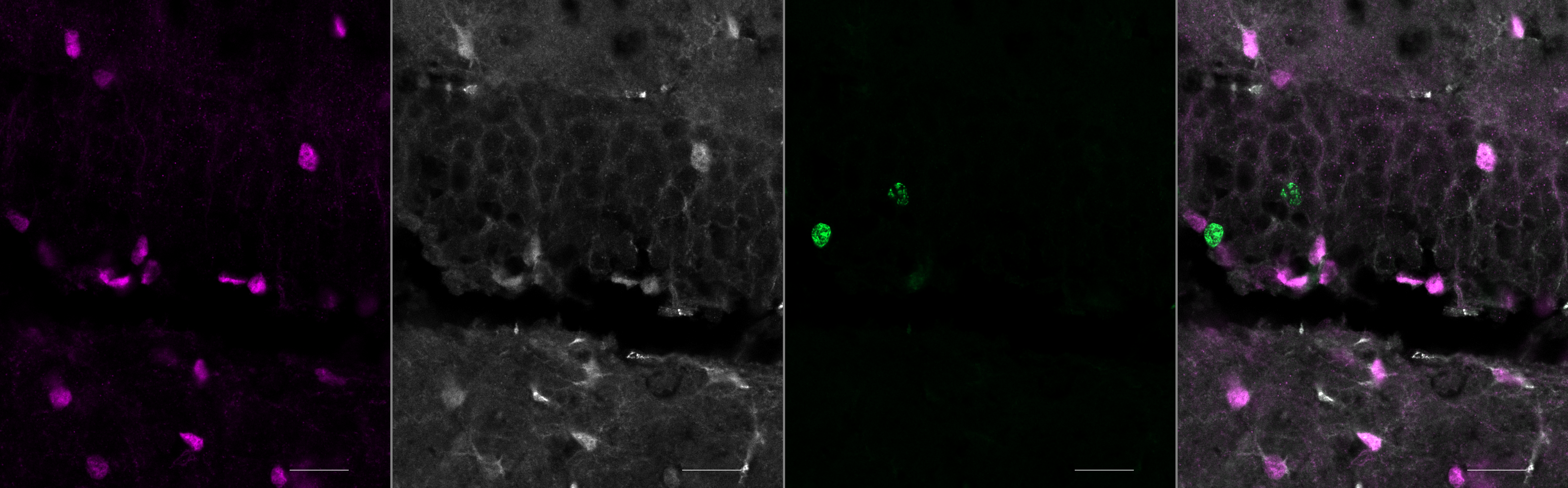

Supplement: Supplementary file 4 — Source Data for Expanded View and Appendix [file EMBR-24-e57269-s001.zip › Figure EV3/Figure EV3/Adolescent. C57BL6J. Ad libitum.tif]

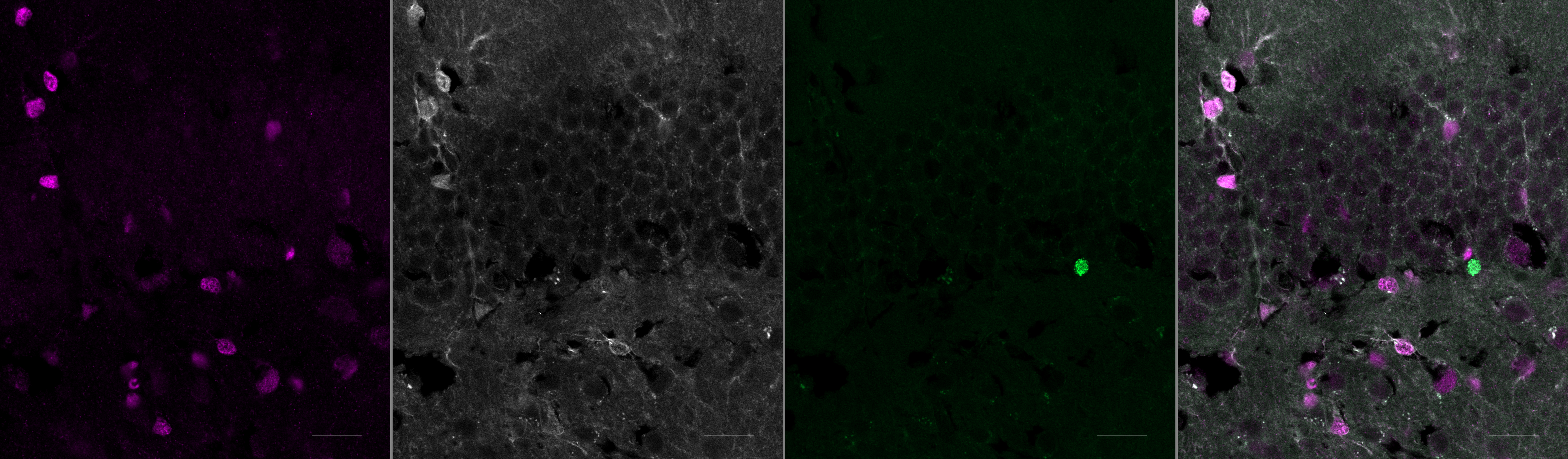

Supplement: Supplementary file 4 — Source Data for Expanded View and Appendix [file EMBR-24-e57269-s001.zip › Figure EV3/Figure EV3/Adult. loxTB-GHSR. Ad libitum.tif]

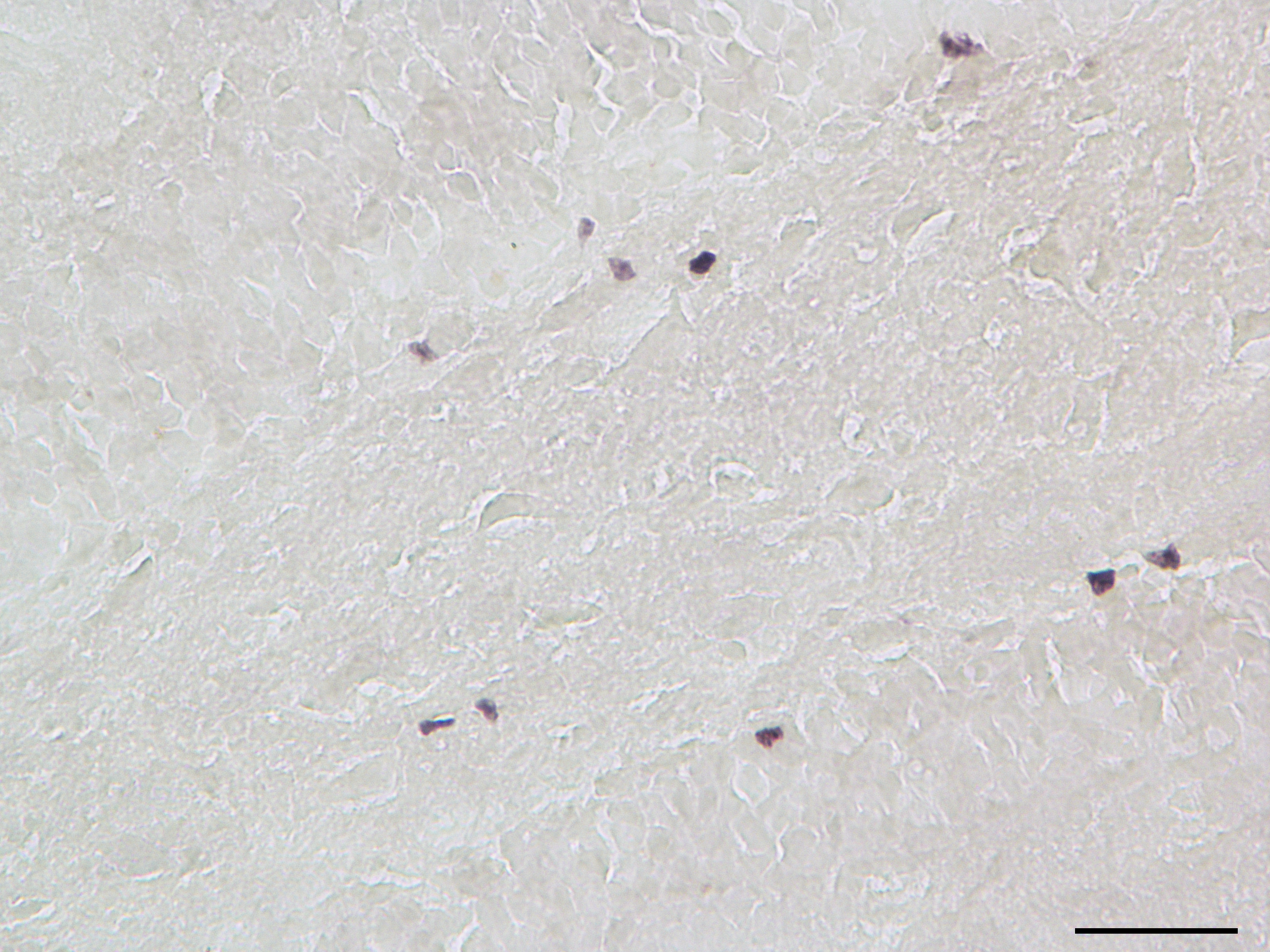

Supplement: Supplementary file 6 — Source Data for Figure 2 [file EMBR-24-e57269-s002.zip › Figure 2/2A/loxTB-GHSR. 5.2 Diet.tif]

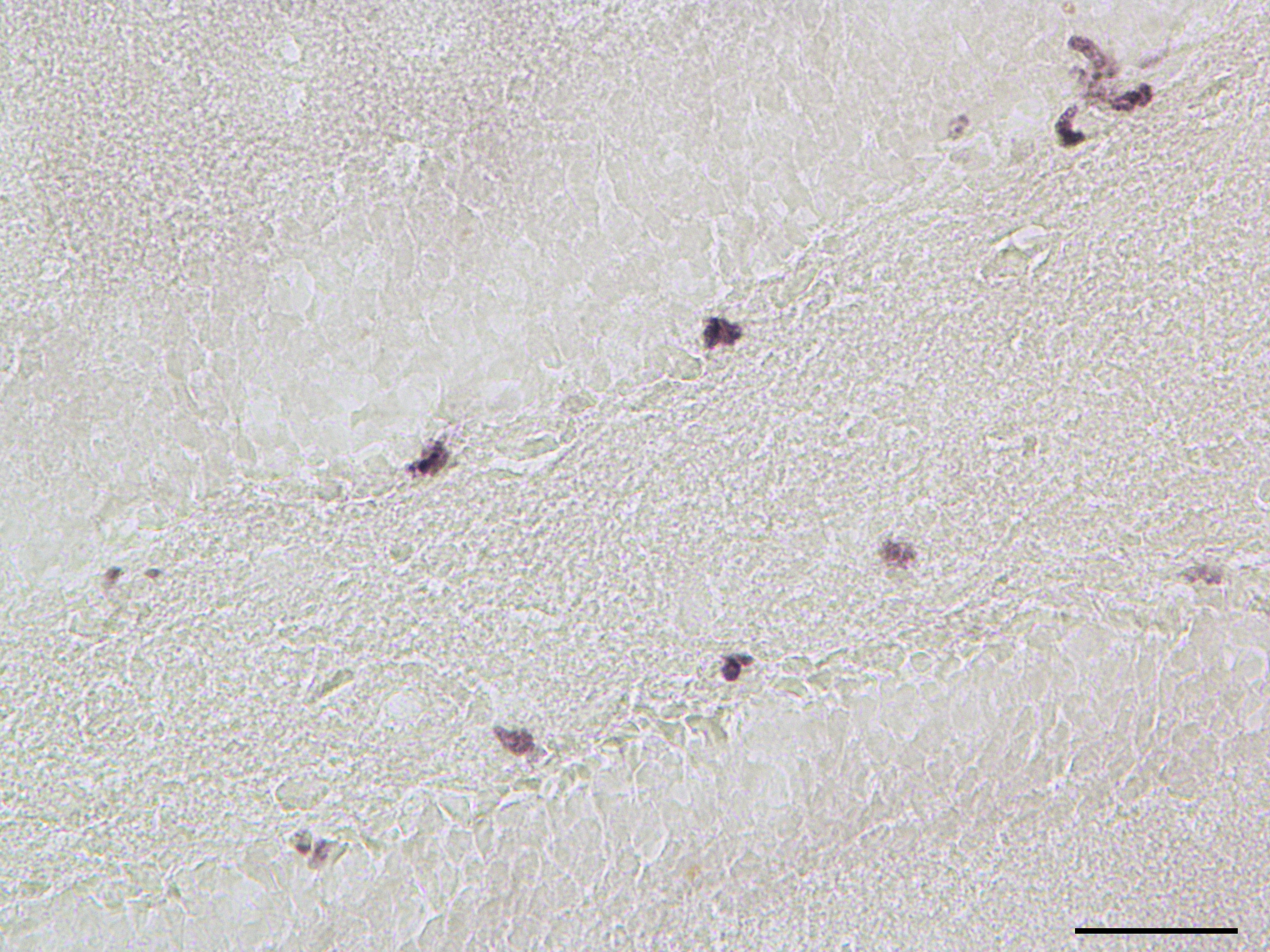

Supplement: Supplementary file 6 — Source Data for Figure 2 [file EMBR-24-e57269-s002.zip › Figure 2/2A/C57BL6J. Ad libitum.tif]

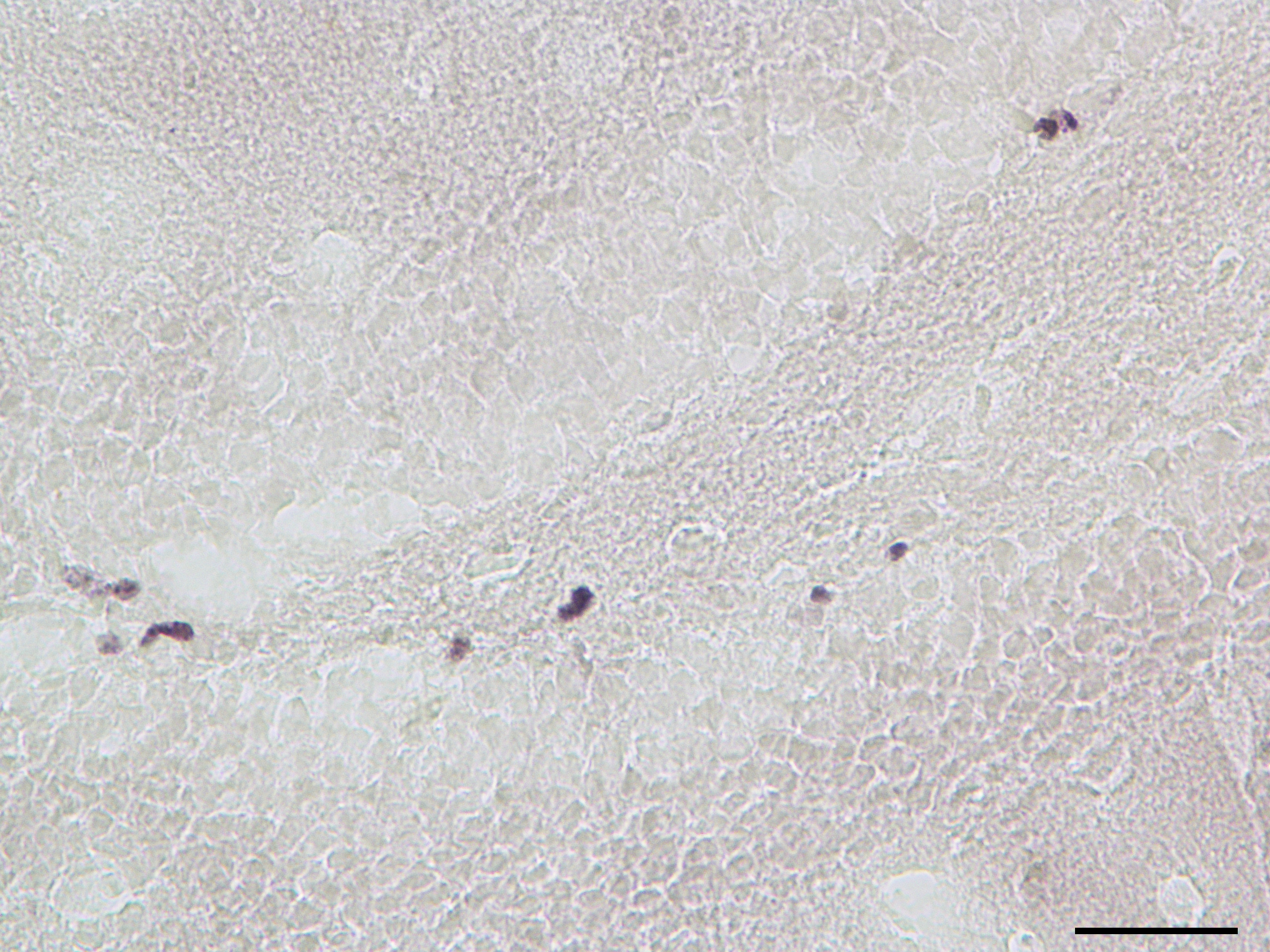

Supplement: Supplementary file 6 — Source Data for Figure 2 [file EMBR-24-e57269-s002.zip › Figure 2/2A/C57BL6J. 5.2 diet.tif]

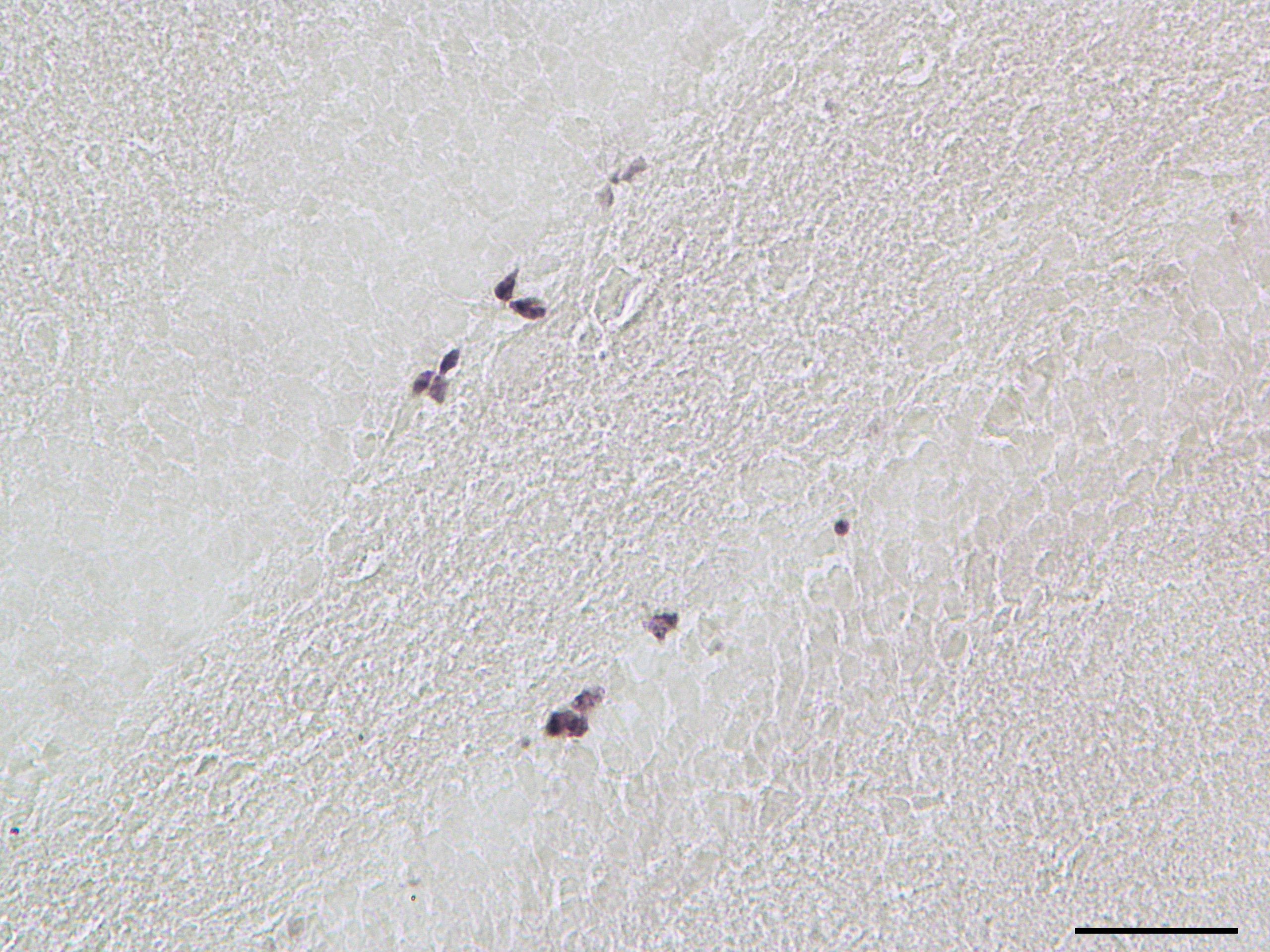

Supplement: Supplementary file 6 — Source Data for Figure 2 [file EMBR-24-e57269-s002.zip › Figure 2/2A/loxTB-GHSR. Ad libitum.tif]

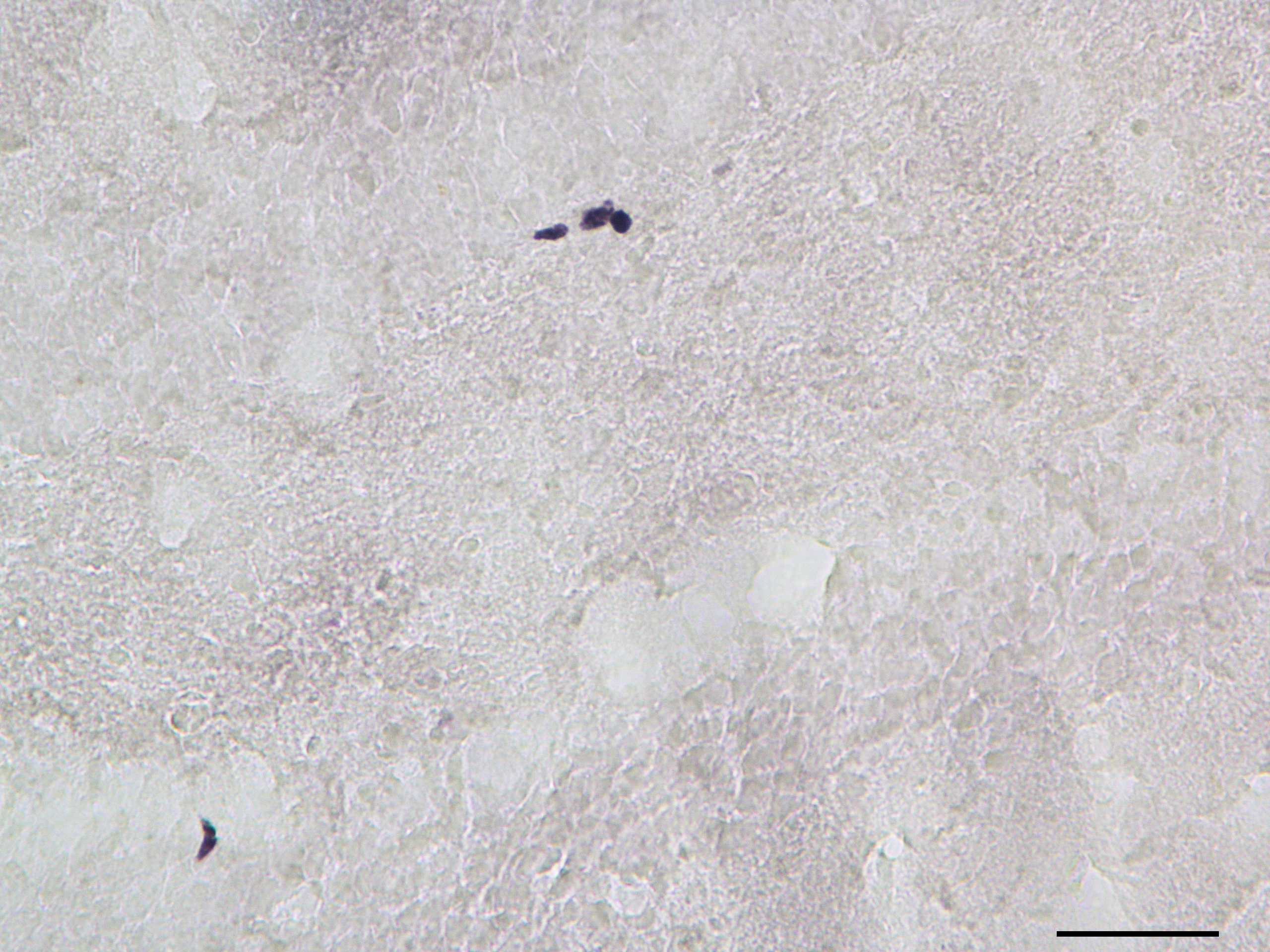

Supplement: Supplementary file 6 — Source Data for Figure 2 [file EMBR-24-e57269-s002.zip › Figure 2/2B/loxTB-GHSR. 5.2 Diet.tif]

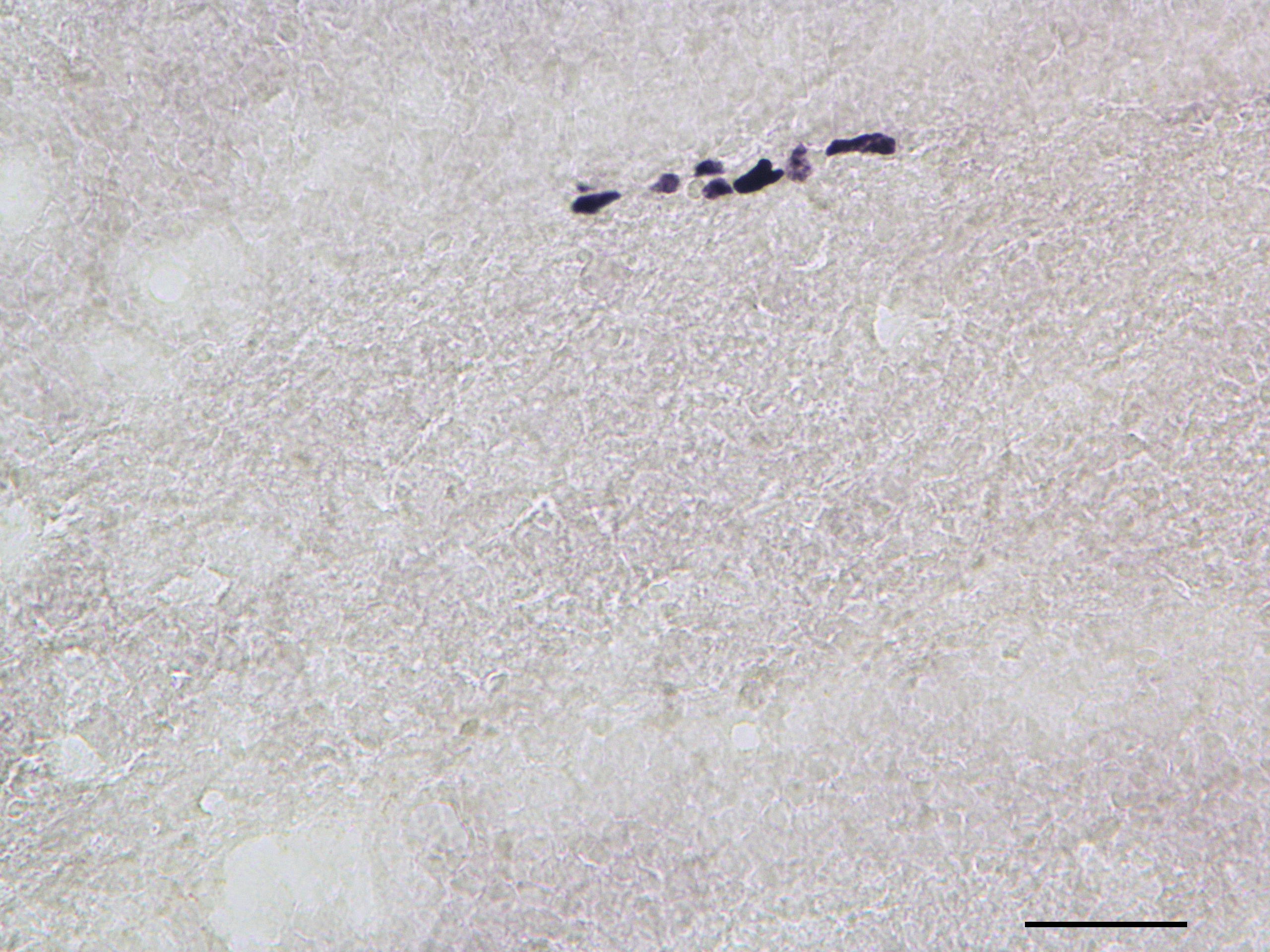

Supplement: Supplementary file 6 — Source Data for Figure 2 [file EMBR-24-e57269-s002.zip › Figure 2/2B/C57BL6J. Ad libitum.tif]

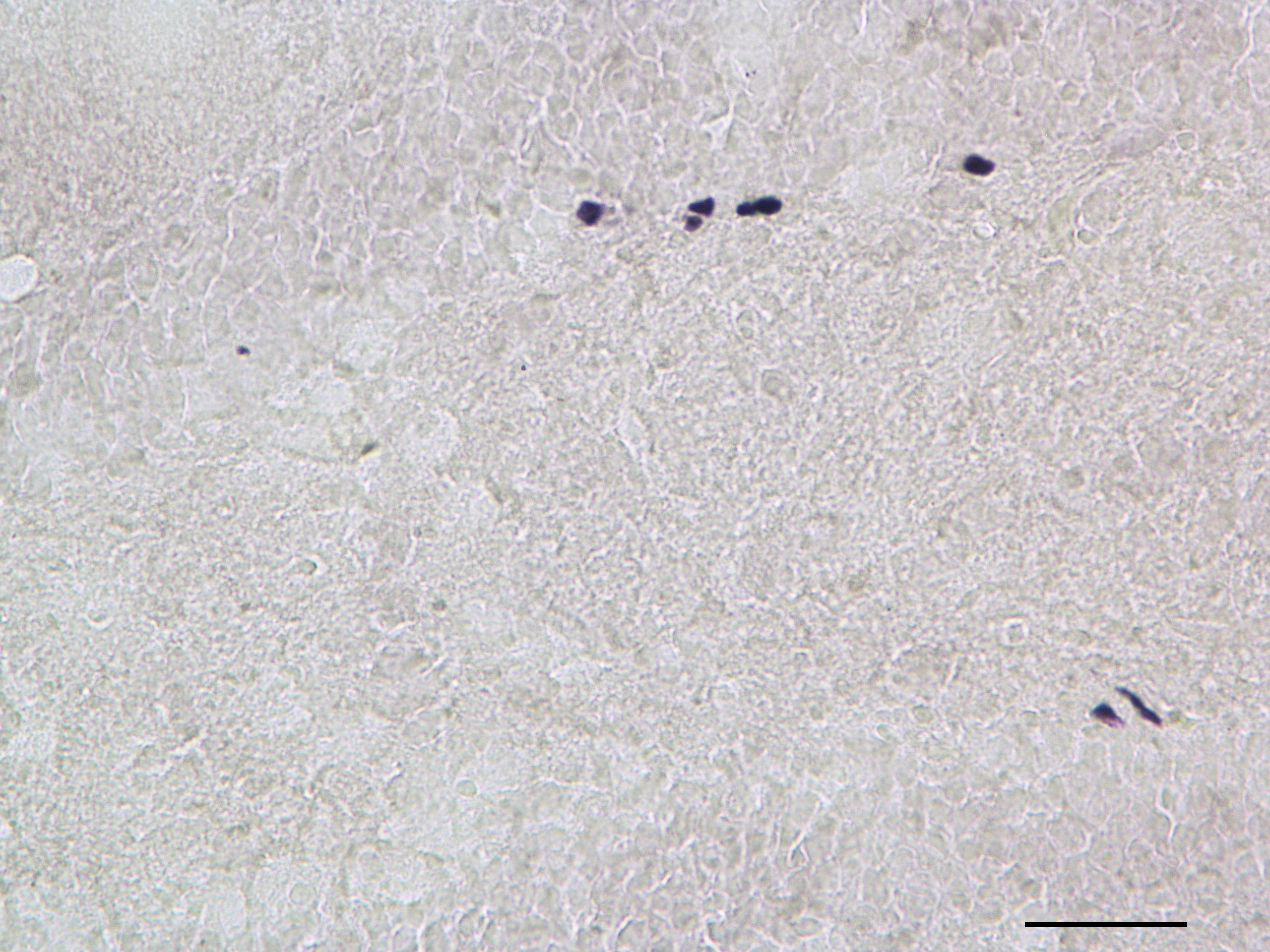

Supplement: Supplementary file 6 — Source Data for Figure 2 [file EMBR-24-e57269-s002.zip › Figure 2/2B/C57BL6J. 5.2 Diet.tif]

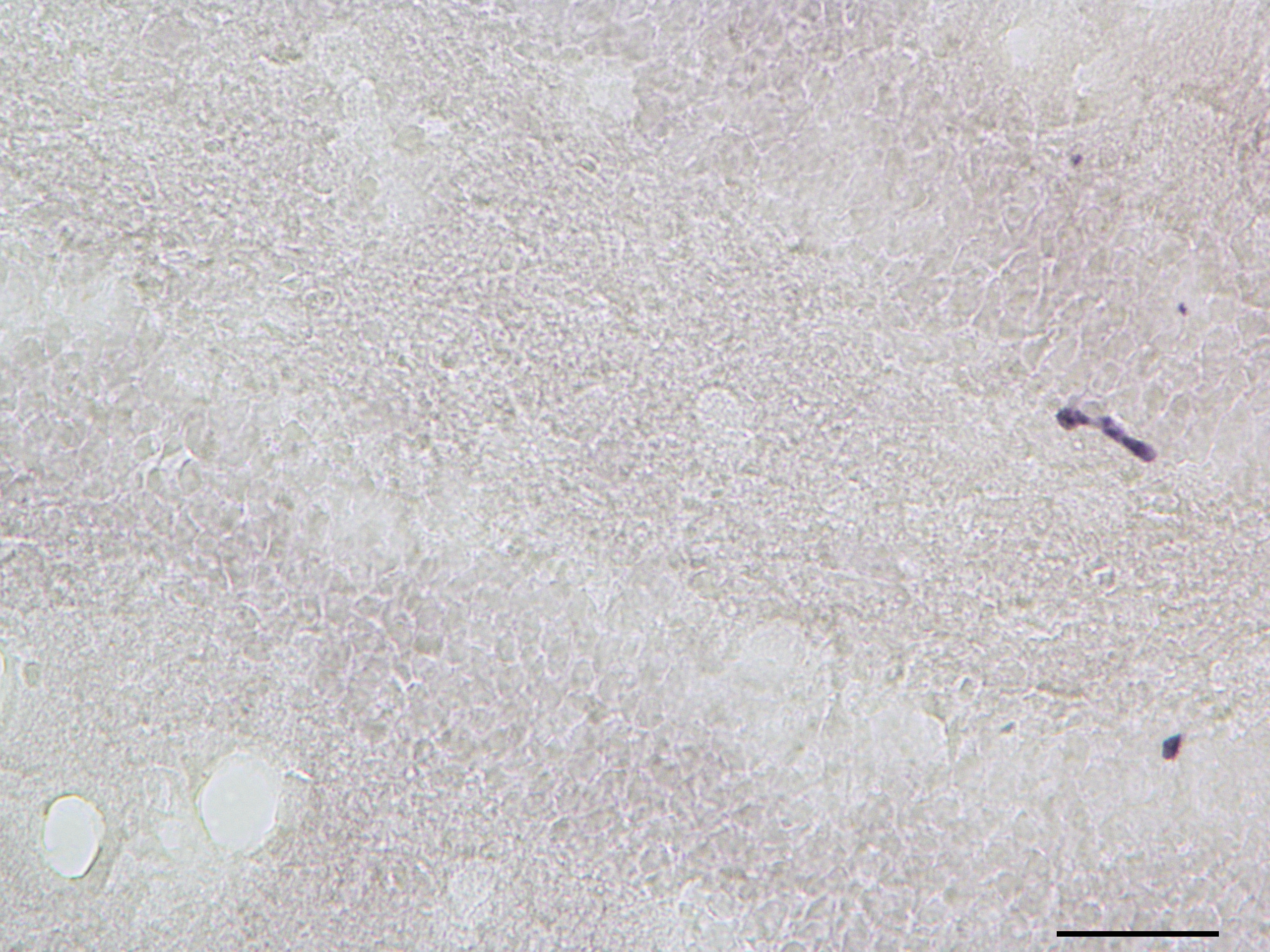

Supplement: Supplementary file 6 — Source Data for Figure 2 [file EMBR-24-e57269-s002.zip › Figure 2/2B/loxTB-GHSR. Ad libitum.tif]

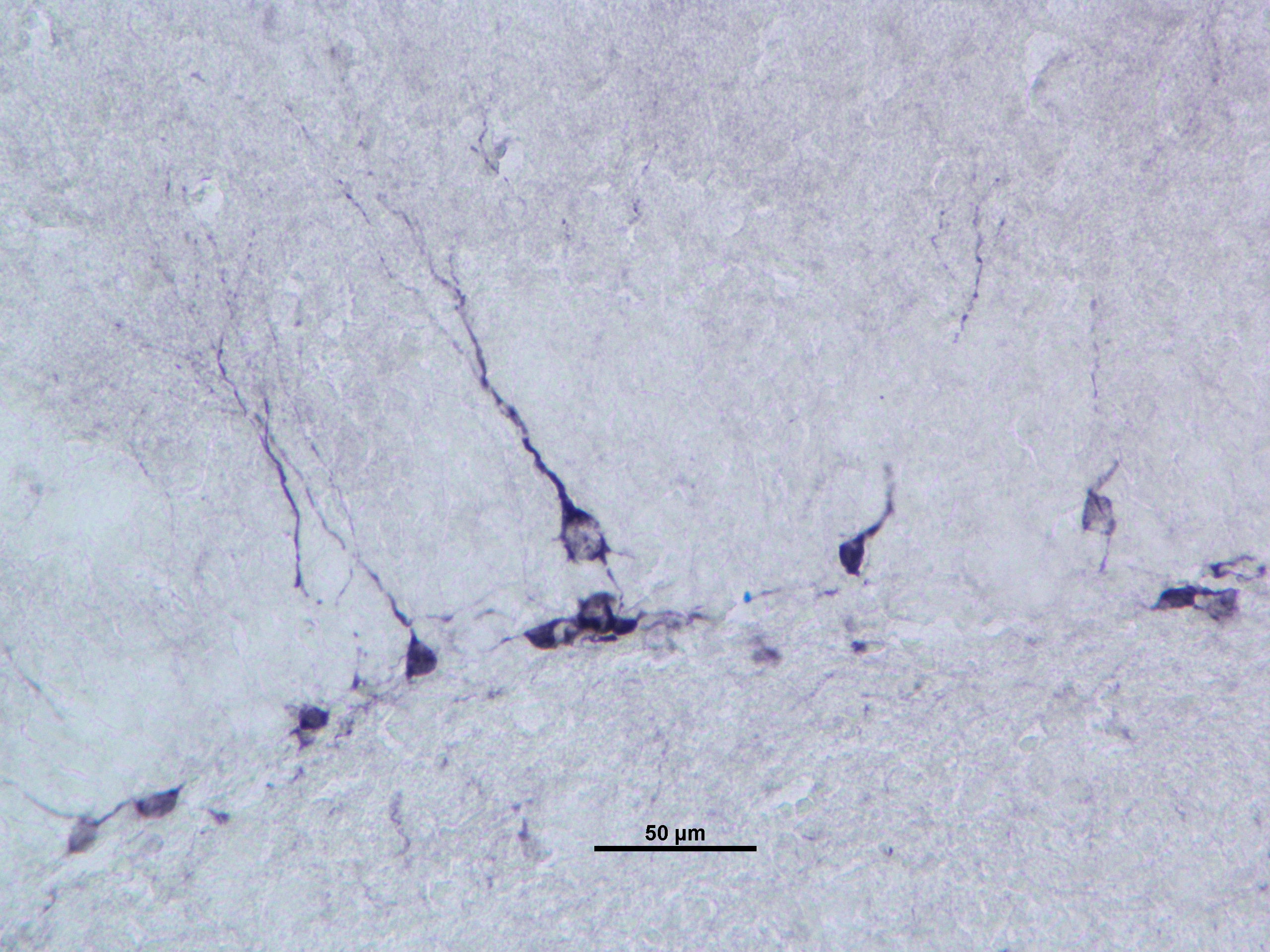

Supplement: Supplementary file 7 — Source Data for Figure 3 [file EMBR-24-e57269-s006.zip › Figure 3/3B/C57BL6J. Ad libitum. 40x objective.tif]

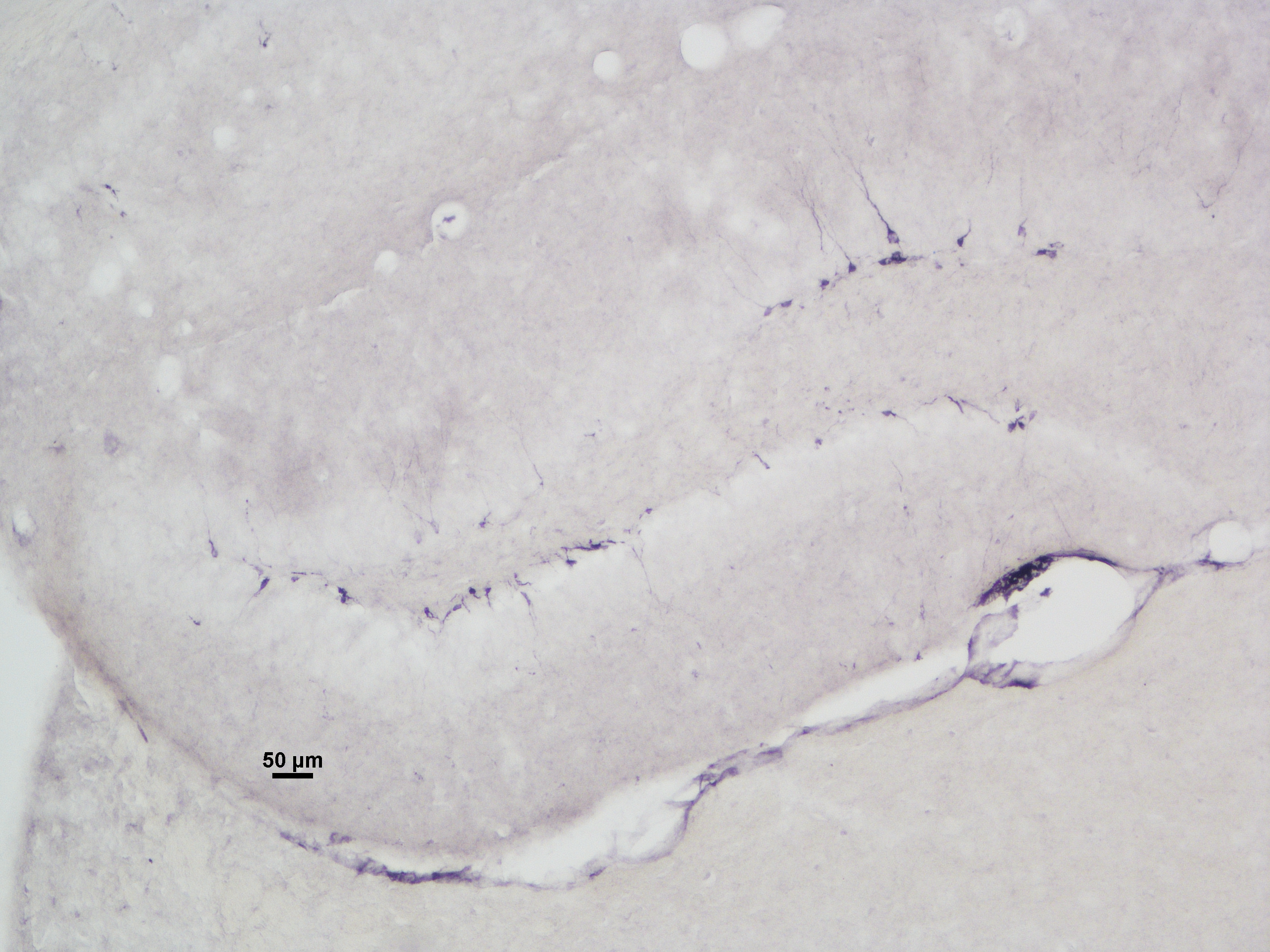

Supplement: Supplementary file 7 — Source Data for Figure 3 [file EMBR-24-e57269-s006.zip › Figure 3/3B/C57BL6J. Ad libitum. 10x objecitve.tif]

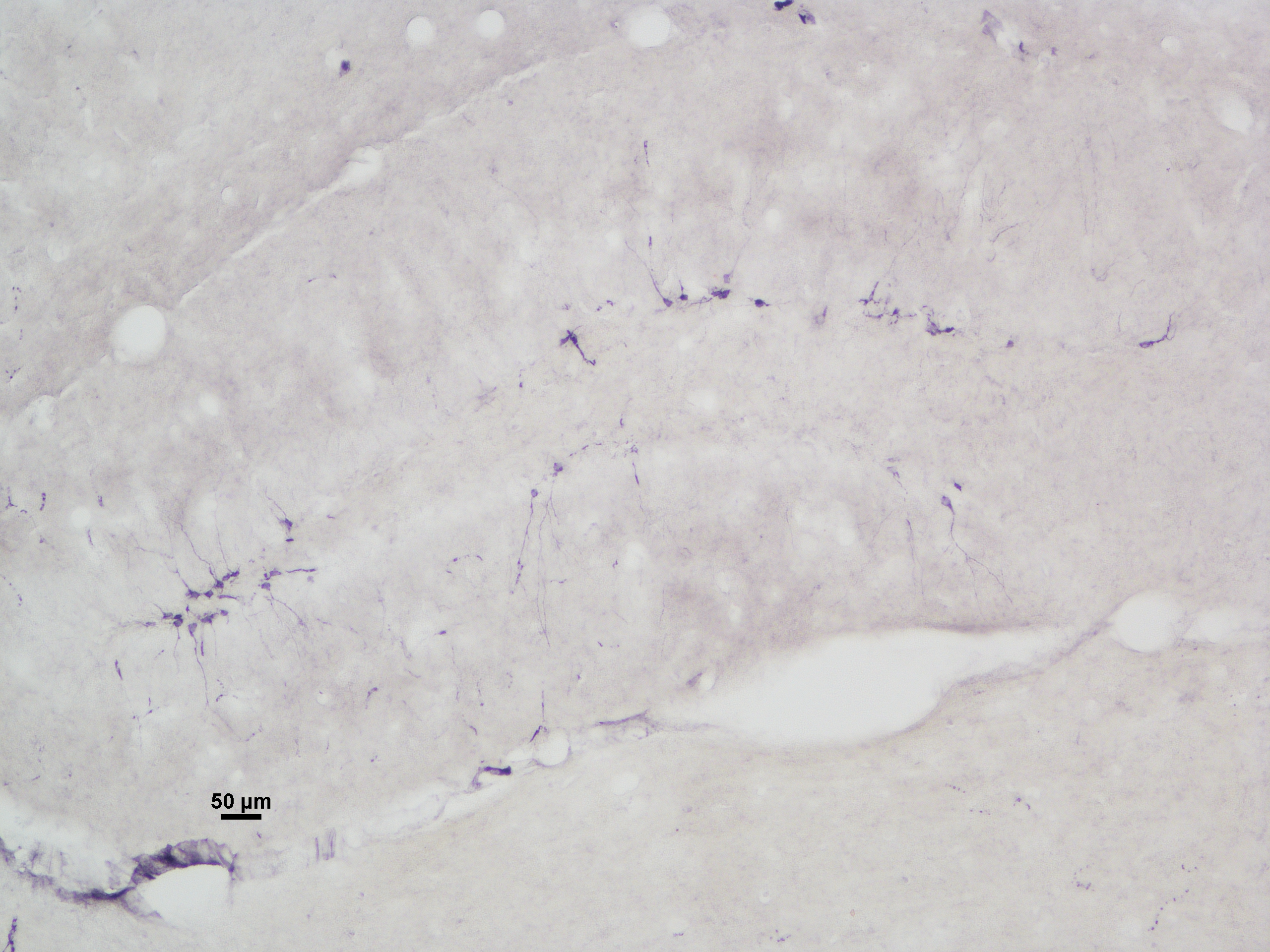

Supplement: Supplementary file 7 — Source Data for Figure 3 [file EMBR-24-e57269-s006.zip › Figure 3/3B/C57BL6J. 5.2 Diet. 10x objective.tif]

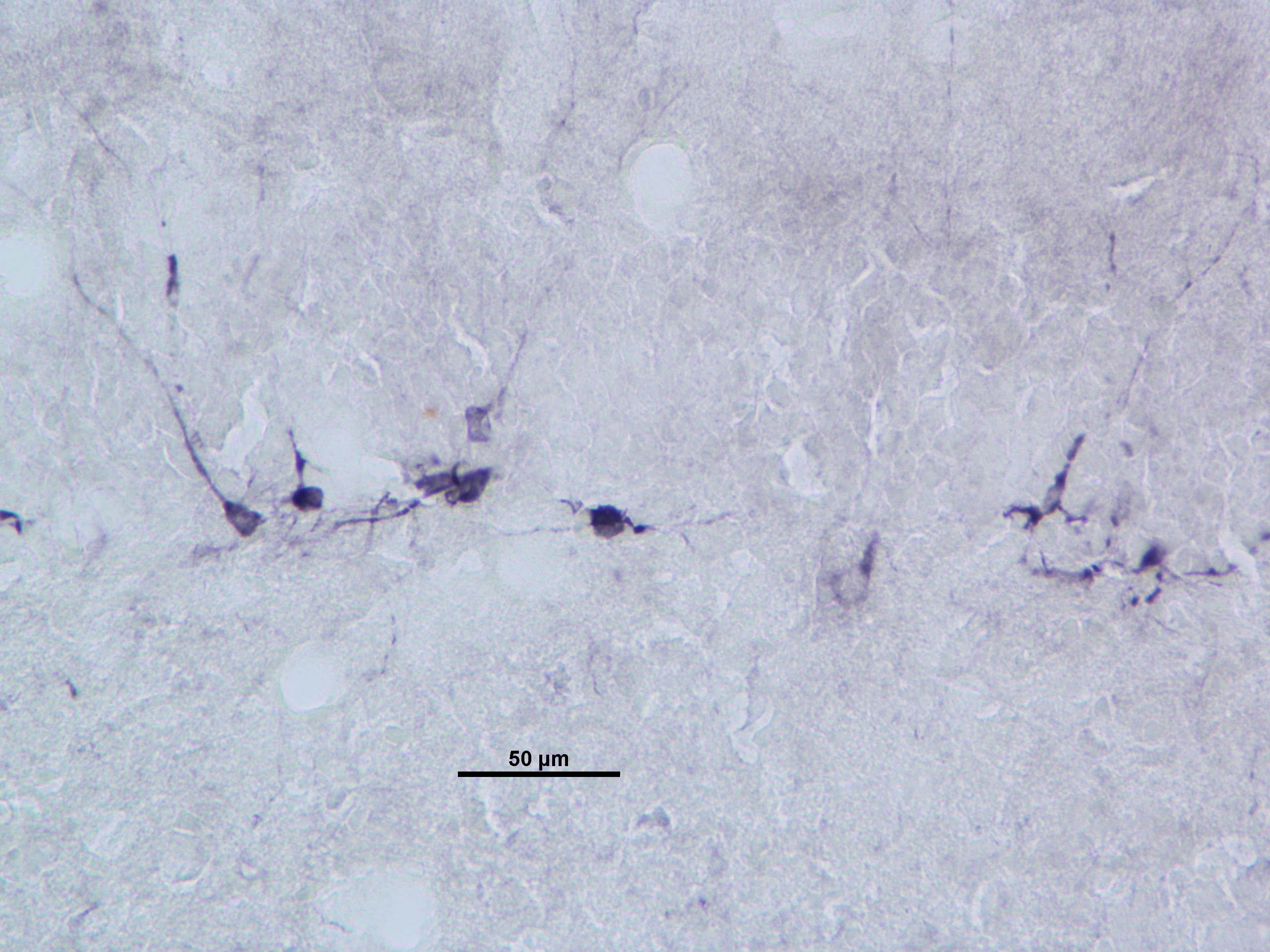

Supplement: Supplementary file 7 — Source Data for Figure 3 [file EMBR-24-e57269-s006.zip › Figure 3/3B/C57BL6J. 5.2 Diet 40x objective.tif]

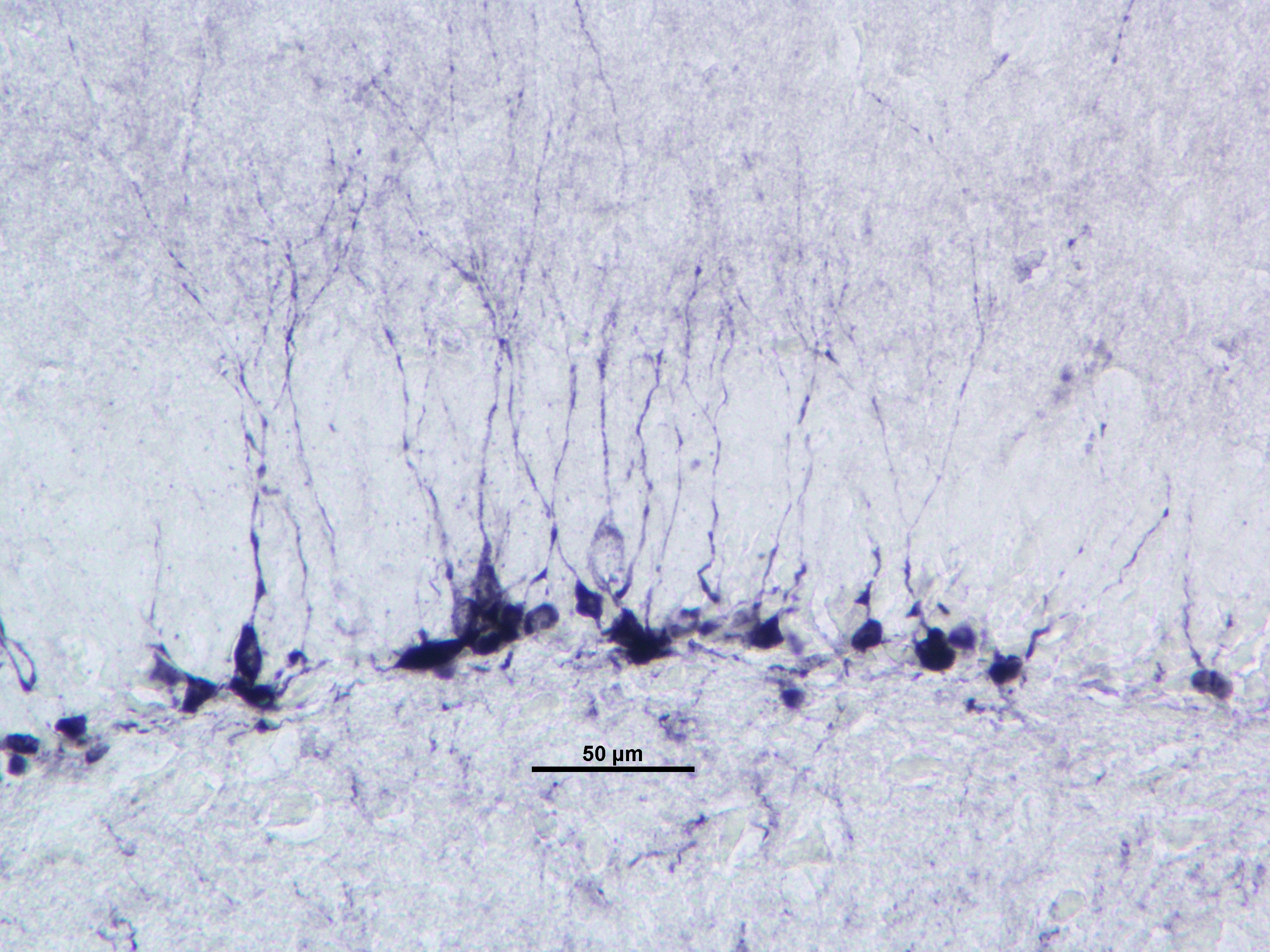

Supplement: Supplementary file 7 — Source Data for Figure 3 [file EMBR-24-e57269-s006.zip › Figure 3/3A/C57BL6J. Ad libitum. 40x objective.tif]

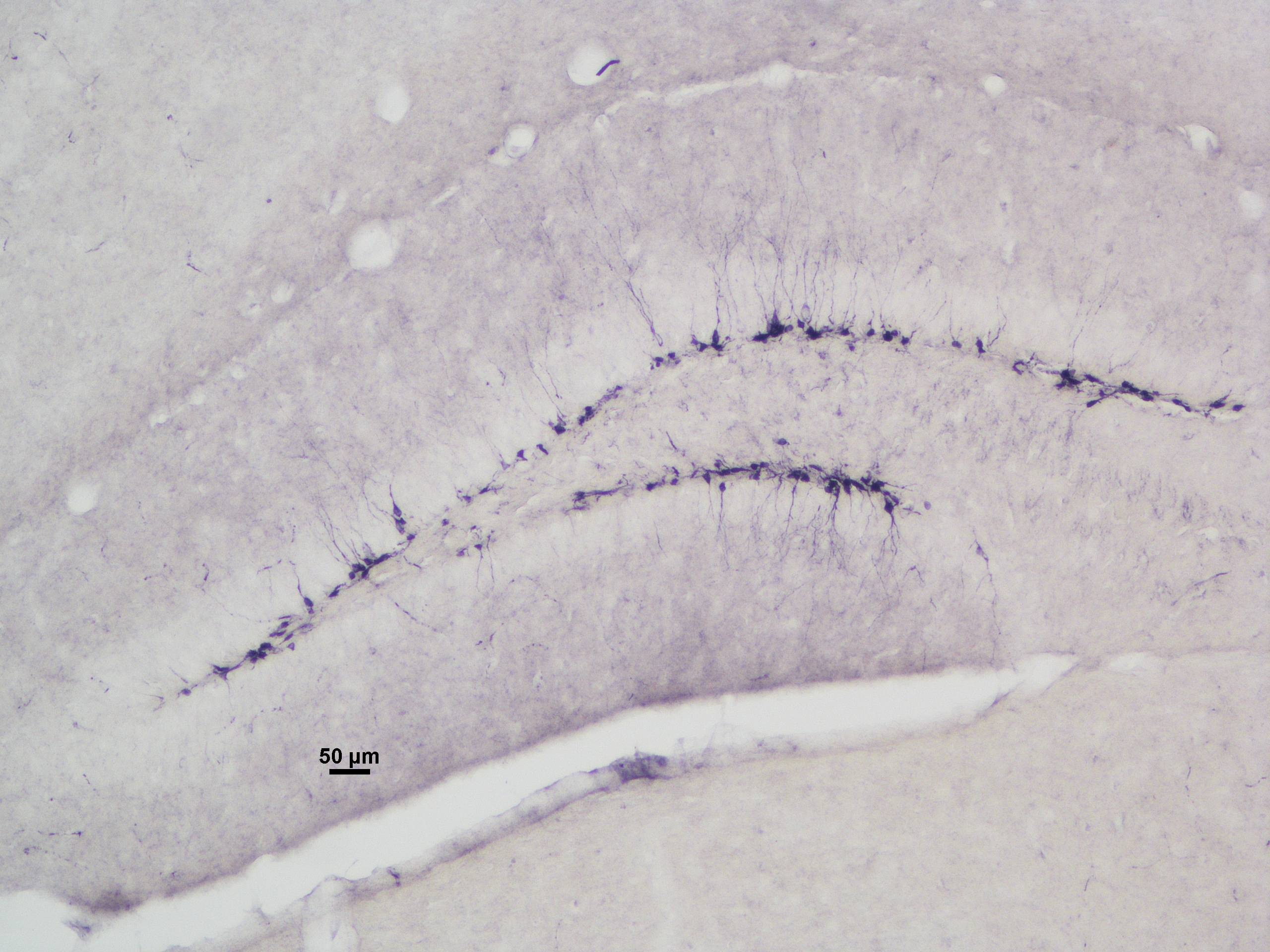

Supplement: Supplementary file 7 — Source Data for Figure 3 [file EMBR-24-e57269-s006.zip › Figure 3/3A/C57BL6J. Ad libitum. 10x objecitve.tif]

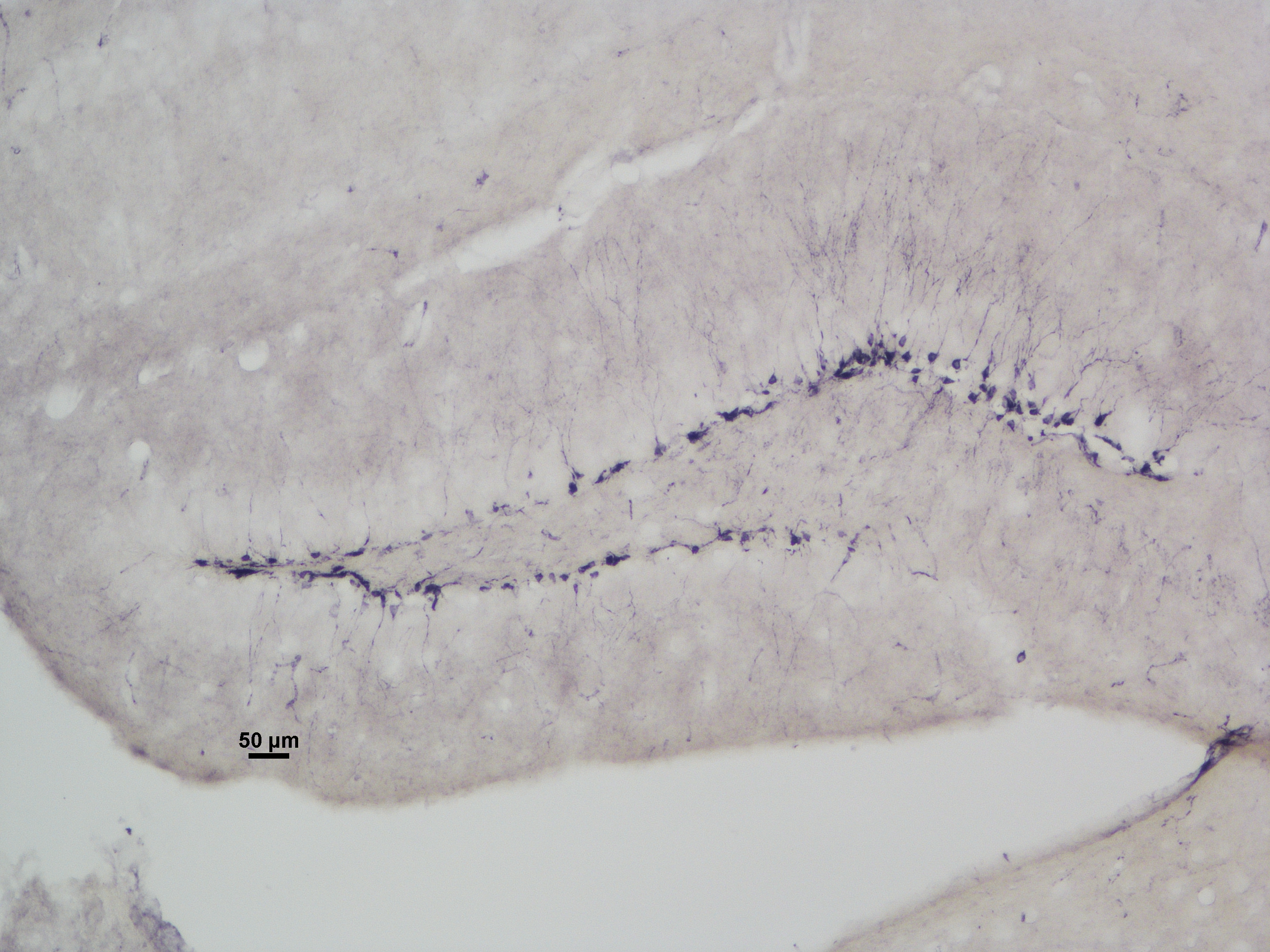

Supplement: Supplementary file 7 — Source Data for Figure 3 [file EMBR-24-e57269-s006.zip › Figure 3/3A/C57BL6J. 5.2 Diet. 10x objecitve.tif]

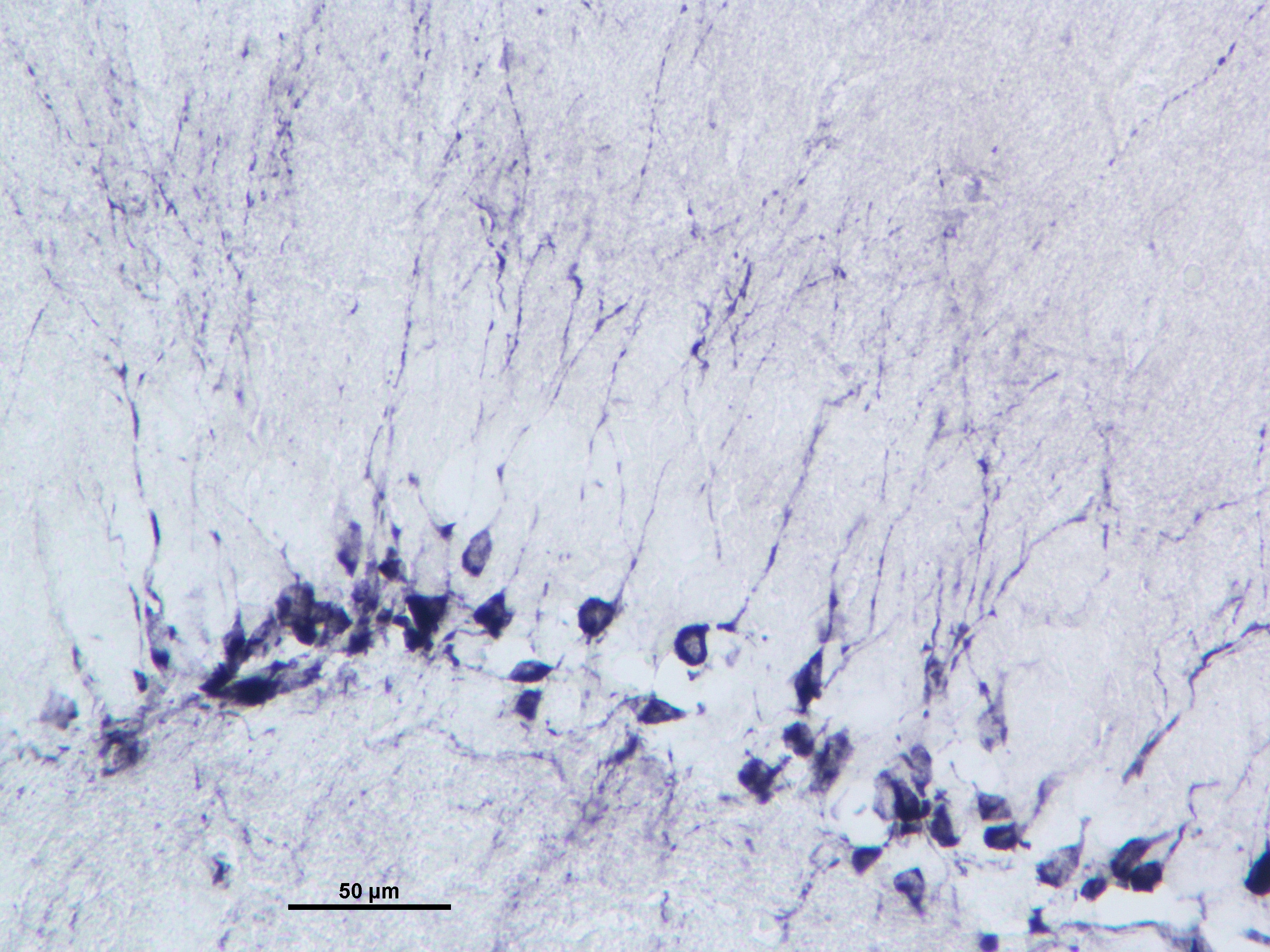

Supplement: Supplementary file 7 — Source Data for Figure 3 [file EMBR-24-e57269-s006.zip › Figure 3/3A/C57BL6J. 5.2 Diet. 40x objective.tif]

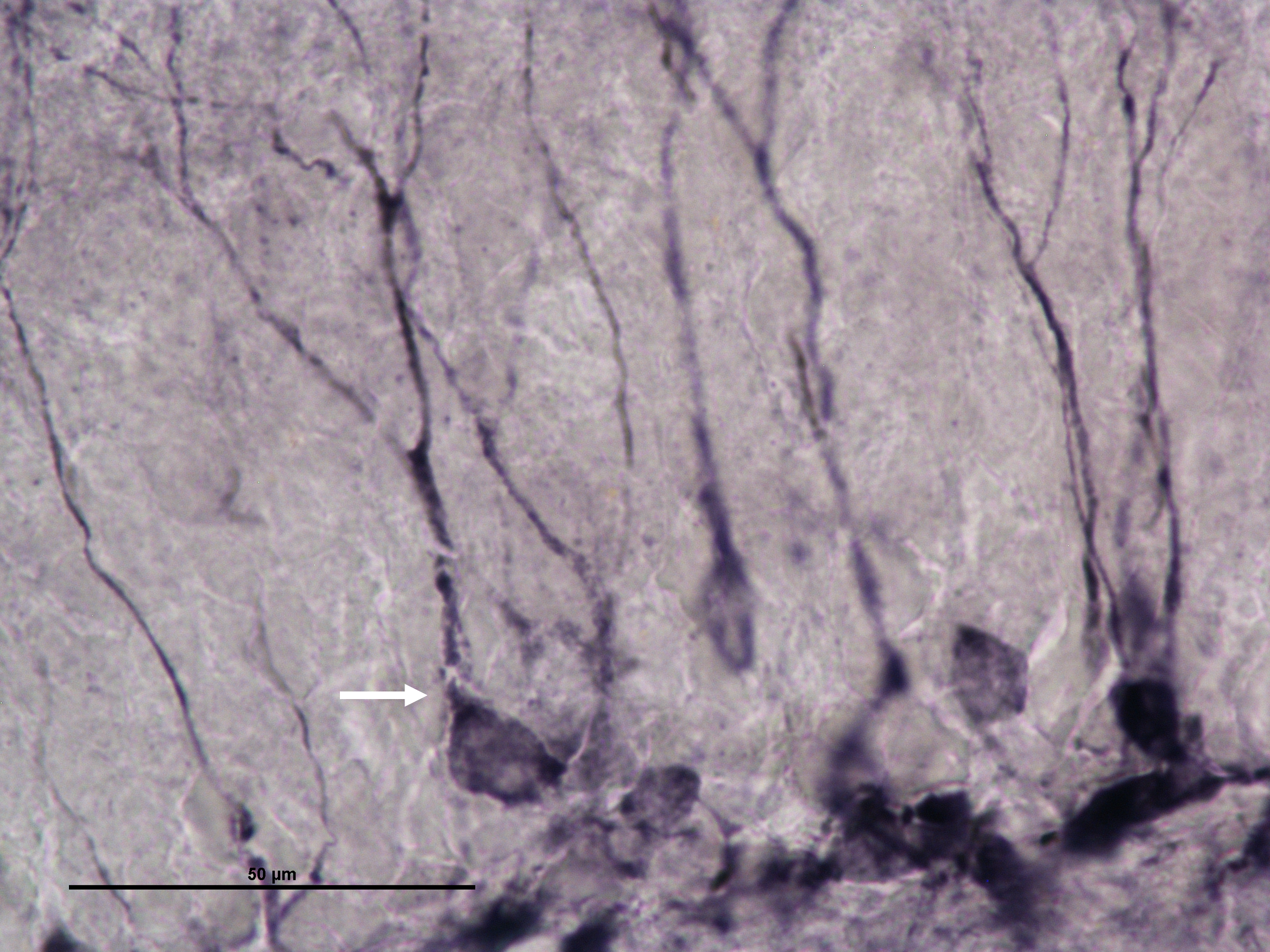

Supplement: Supplementary file 7 — Source Data for Figure 3 [file EMBR-24-e57269-s006.zip › Figure 3/3F/Morphology F.tif]

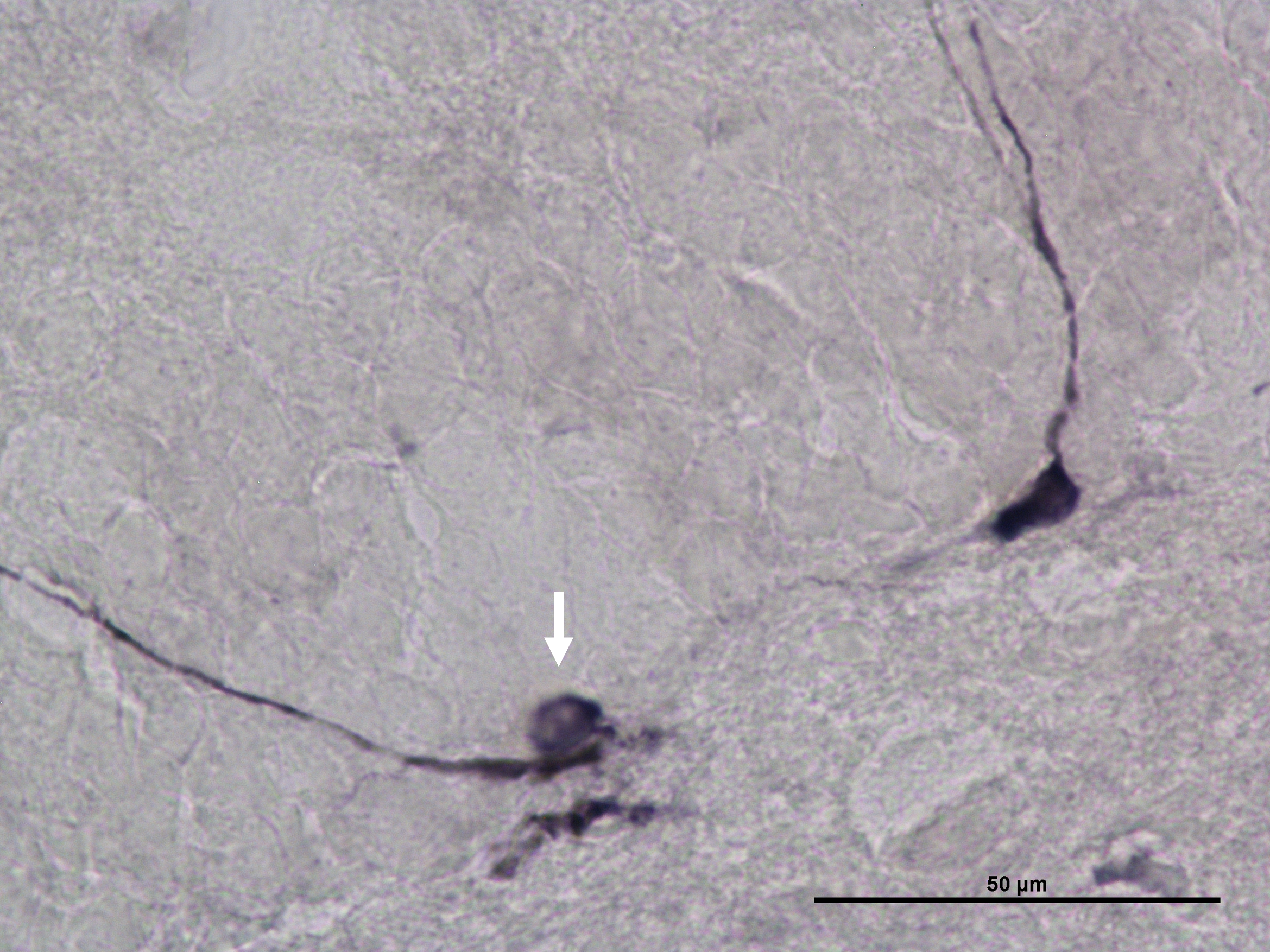

Supplement: Supplementary file 7 — Source Data for Figure 3 [file EMBR-24-e57269-s006.zip › Figure 3/3F/Morphology D.tif]

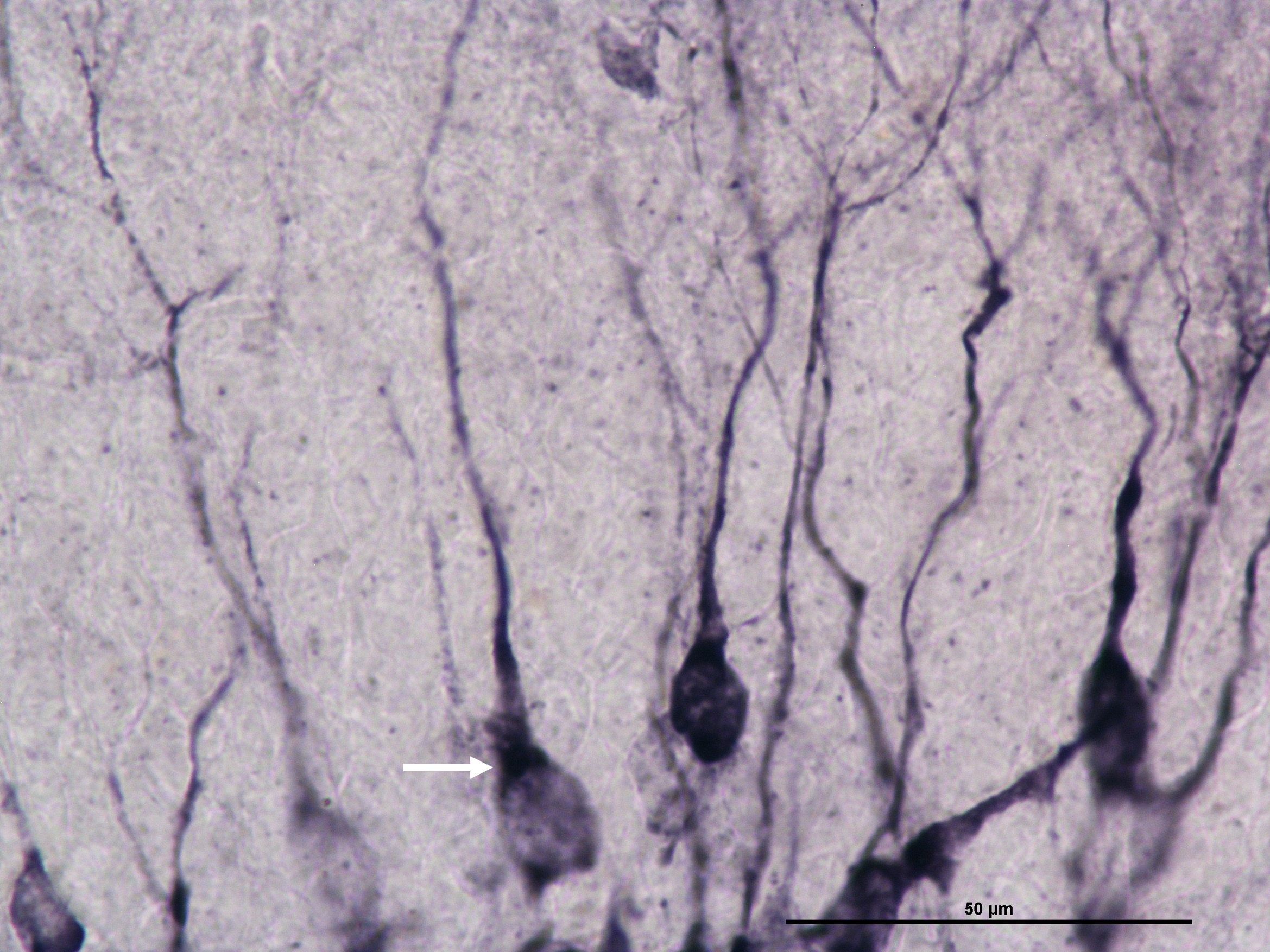

Supplement: Supplementary file 7 — Source Data for Figure 3 [file EMBR-24-e57269-s006.zip › Figure 3/3F/Morphology E.tif]

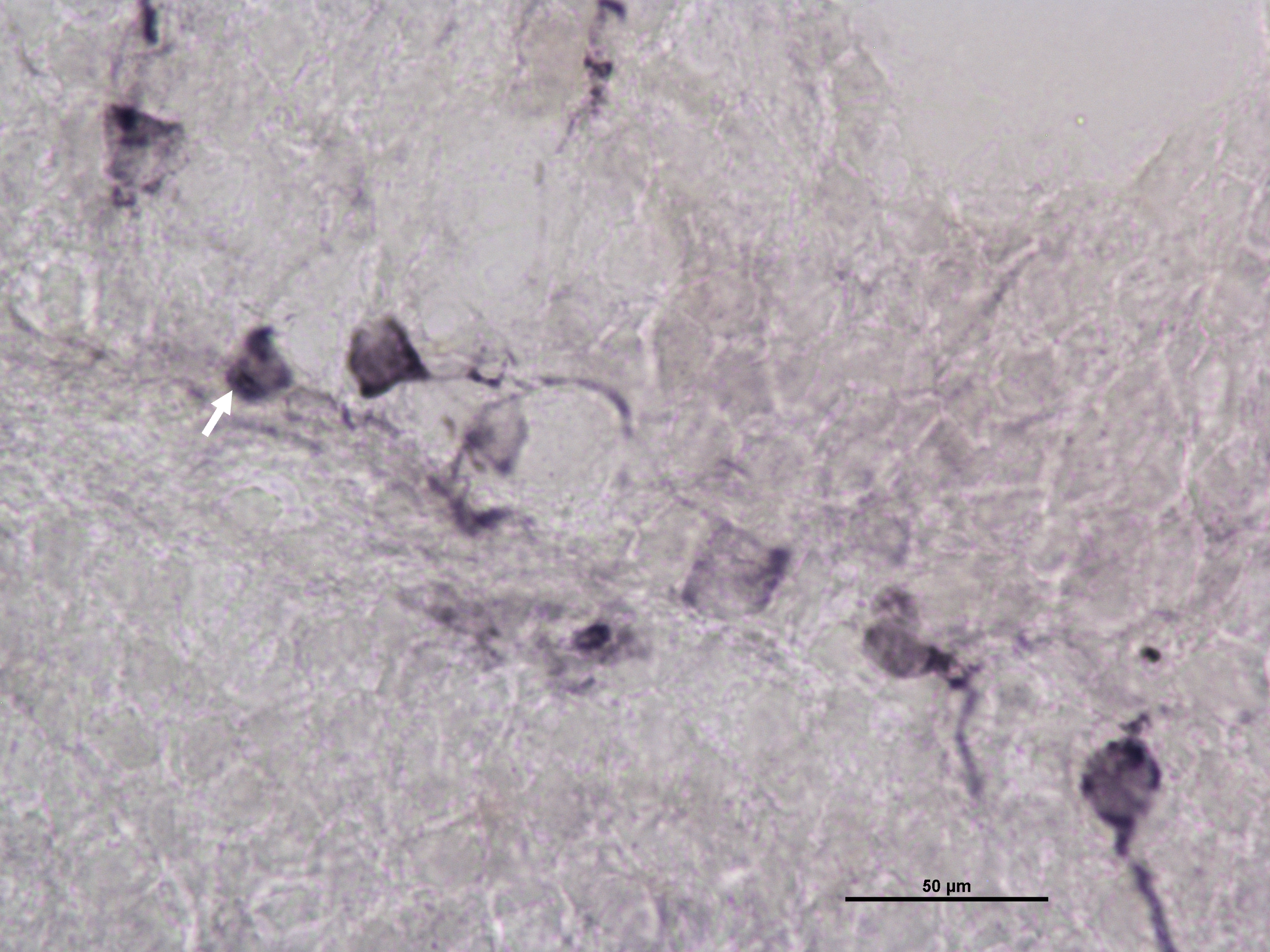

Supplement: Supplementary file 7 — Source Data for Figure 3 [file EMBR-24-e57269-s006.zip › Figure 3/3F/Morphology A.tif]

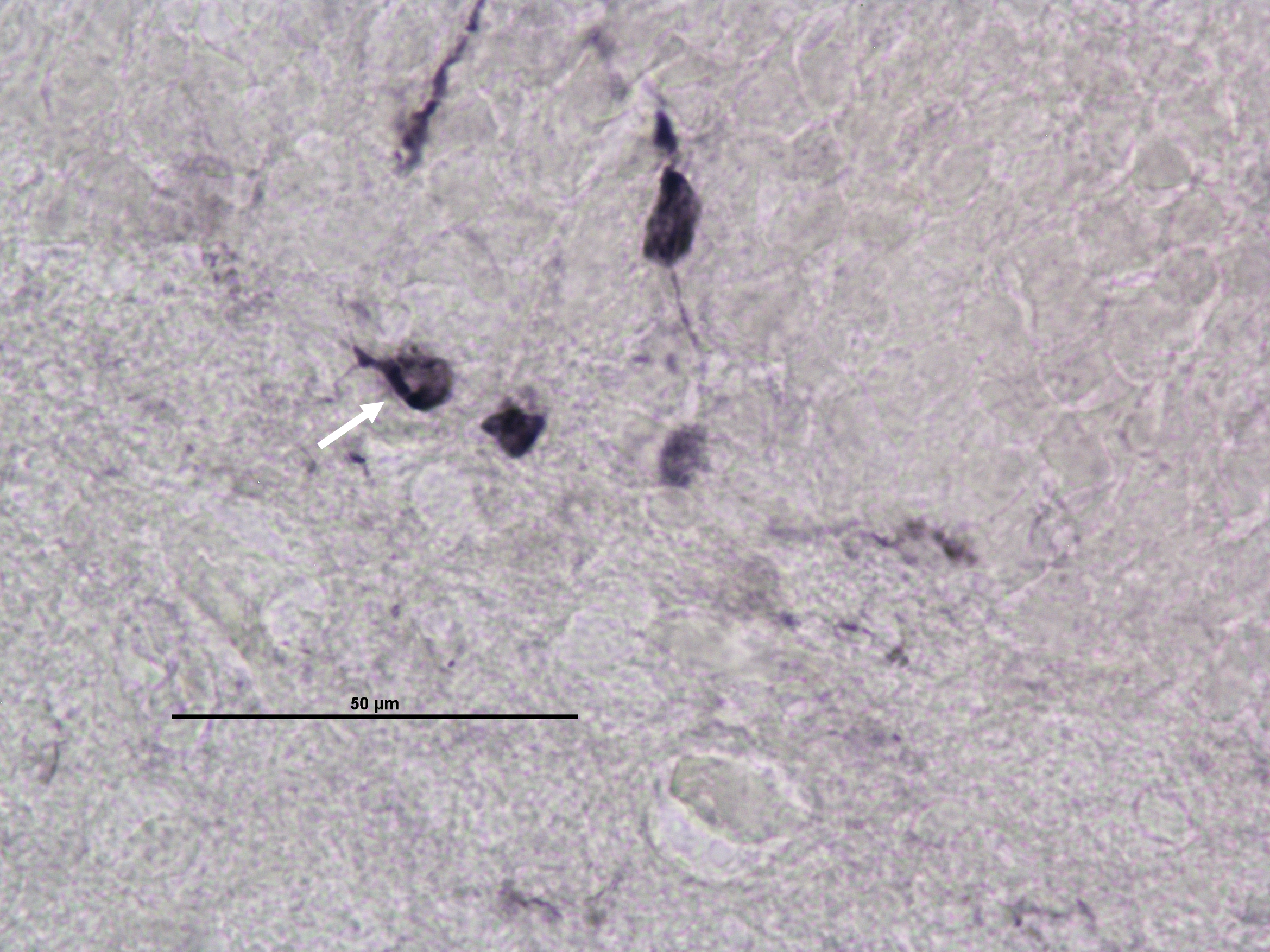

Supplement: Supplementary file 7 — Source Data for Figure 3 [file EMBR-24-e57269-s006.zip › Figure 3/3F/Morphology B.tif]

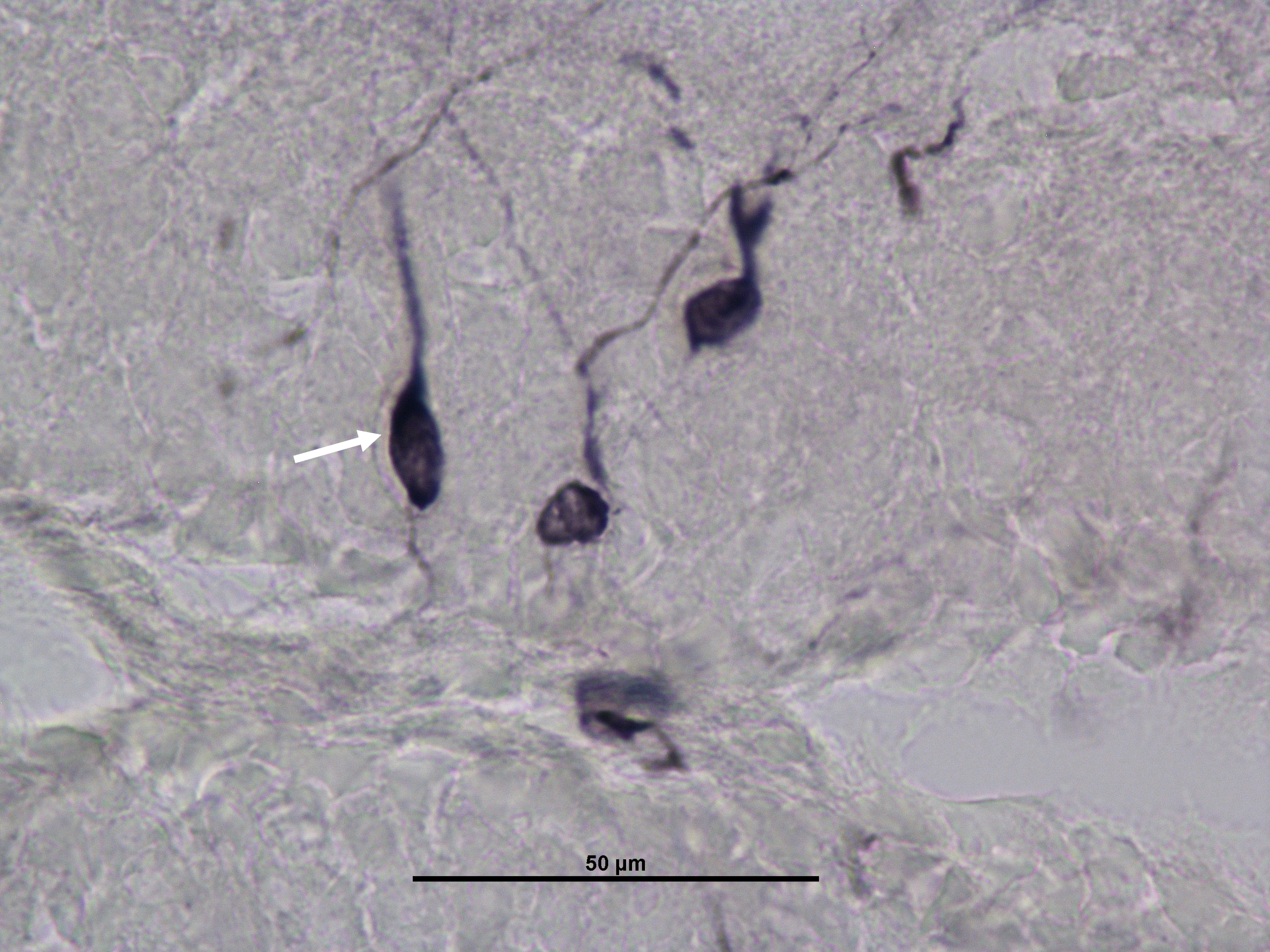

Supplement: Supplementary file 7 — Source Data for Figure 3 [file EMBR-24-e57269-s006.zip › Figure 3/3F/Morphology C.tif]

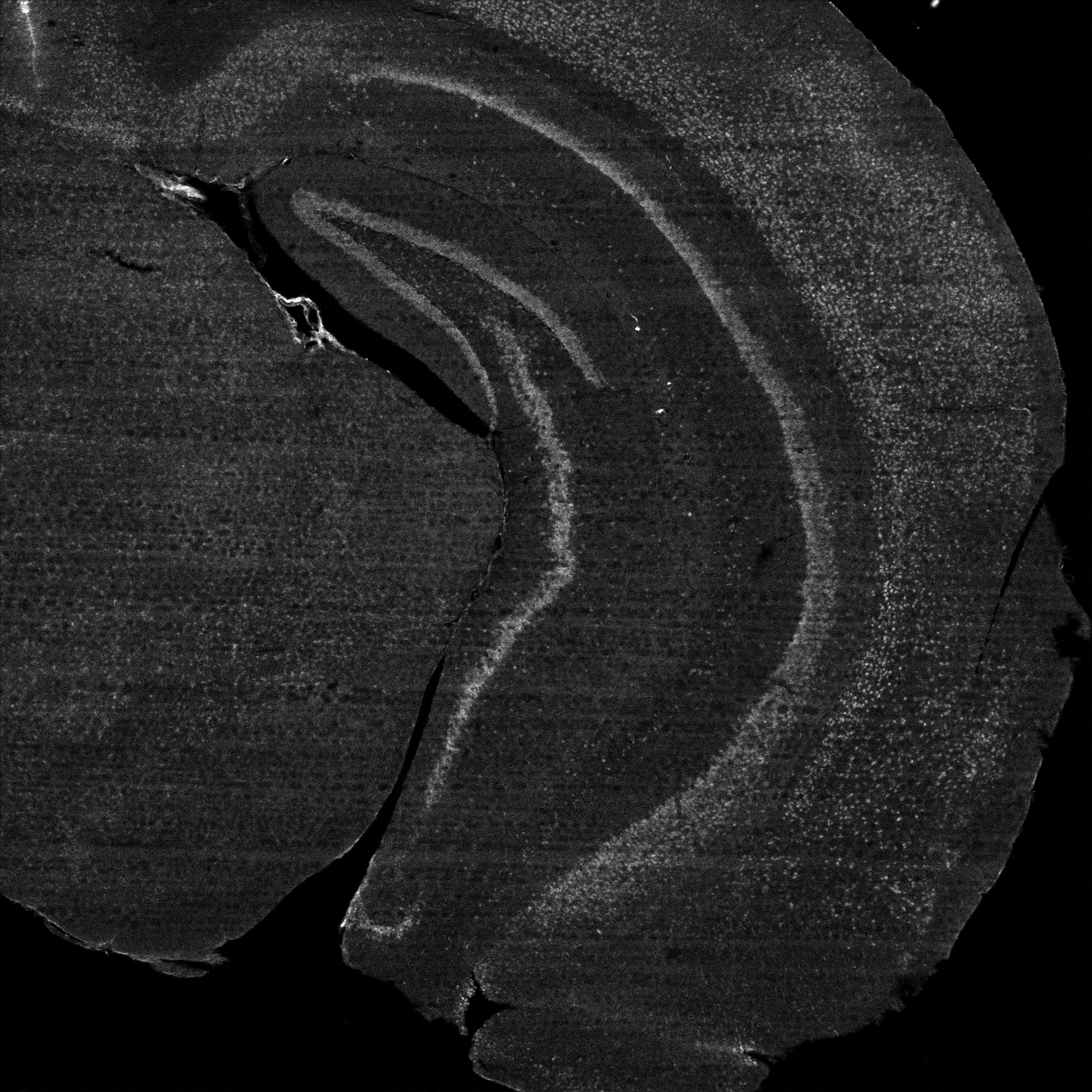

Supplement: Supplementary file 8 — Source Data for Figure 4 [file EMBR-24-e57269-s010.zip › Figure 4/4D/Caudal.2. (Bregma -3.08 mm).tif]

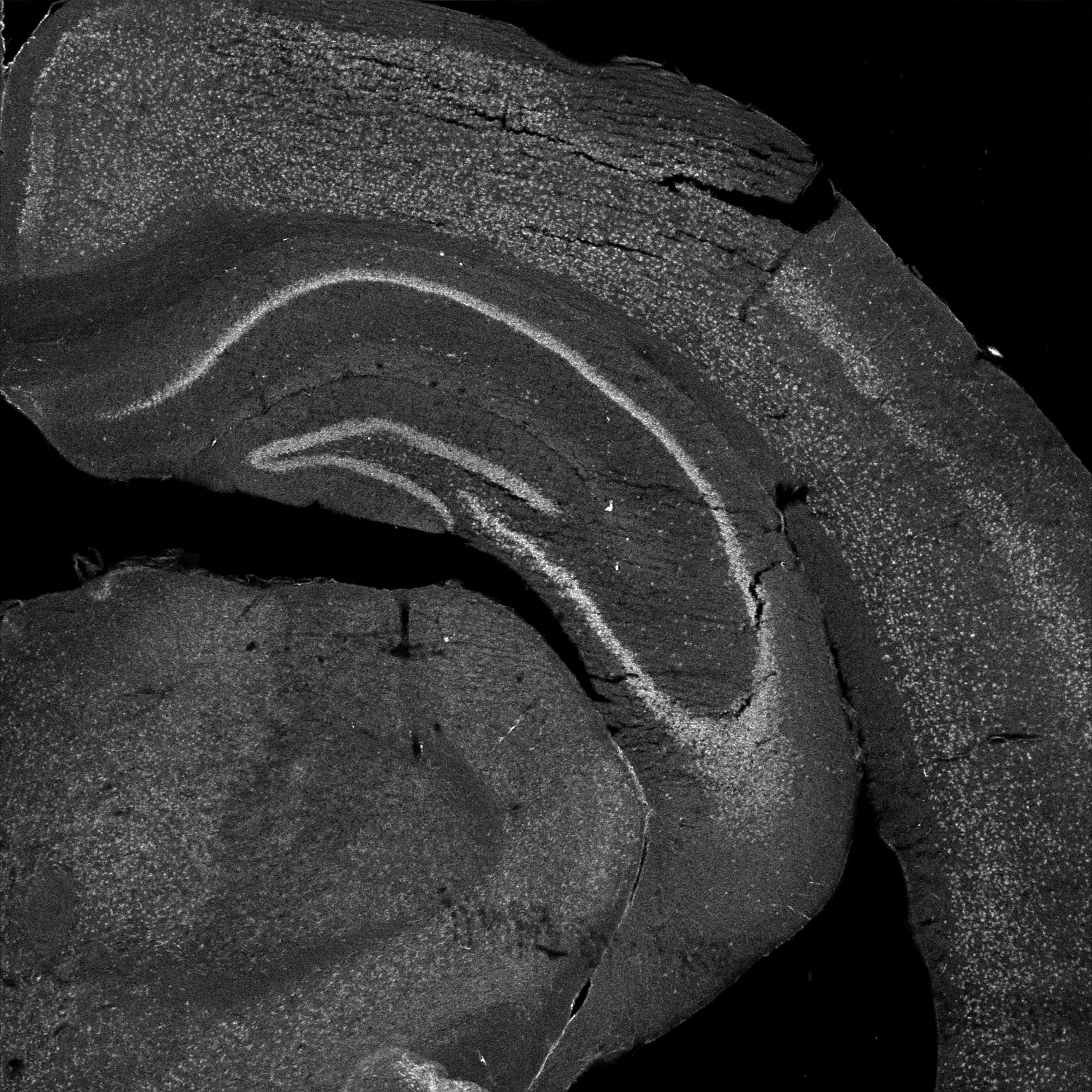

Supplement: Supplementary file 8 — Source Data for Figure 4 [file EMBR-24-e57269-s010.zip › Figure 4/4D/Caudal.1 (Bregma -2.80 mm).tif]

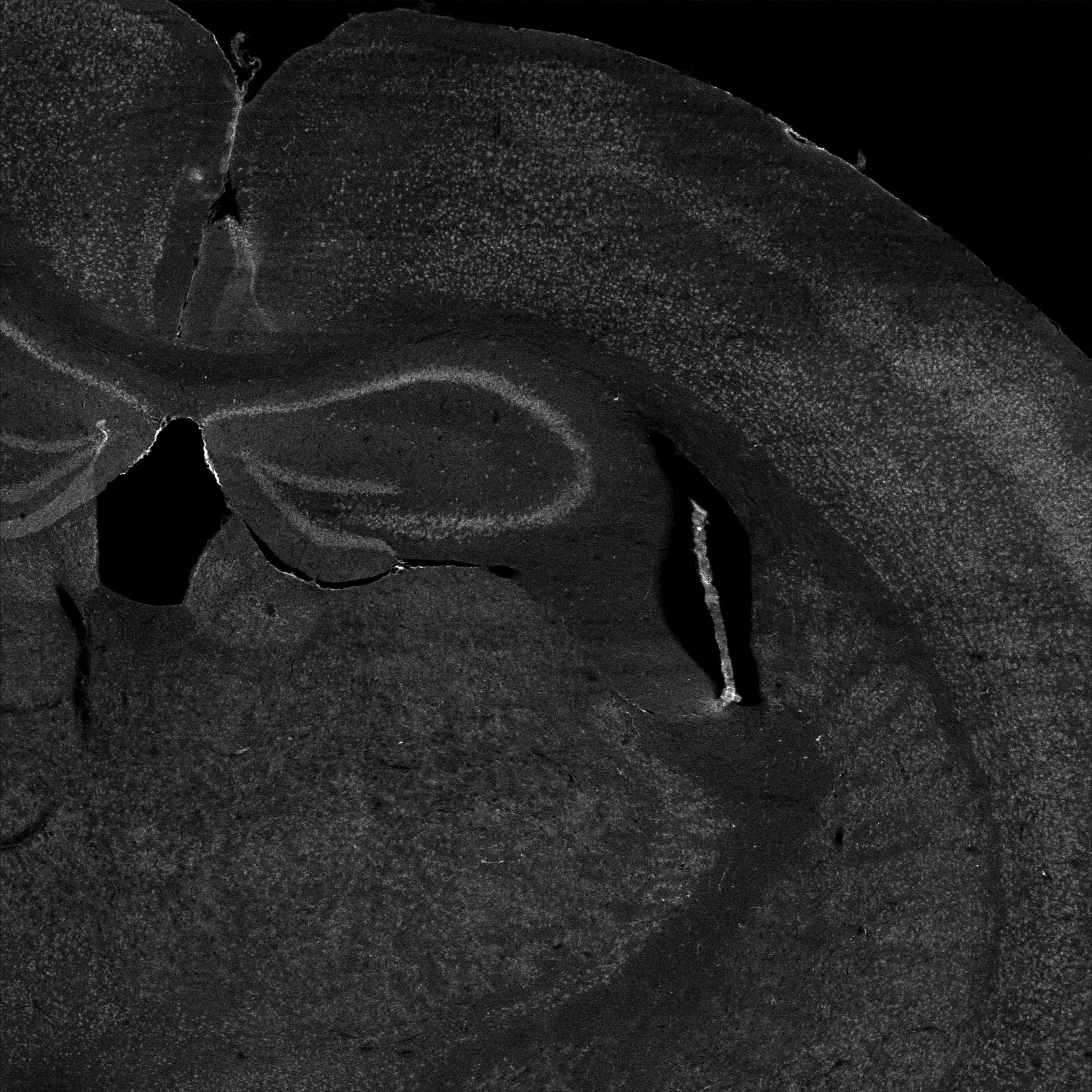

Supplement: Supplementary file 8 — Source Data for Figure 4 [file EMBR-24-e57269-s010.zip › Figure 4/4D/Rostral.1 (Bregma -1.34 mm).tif]

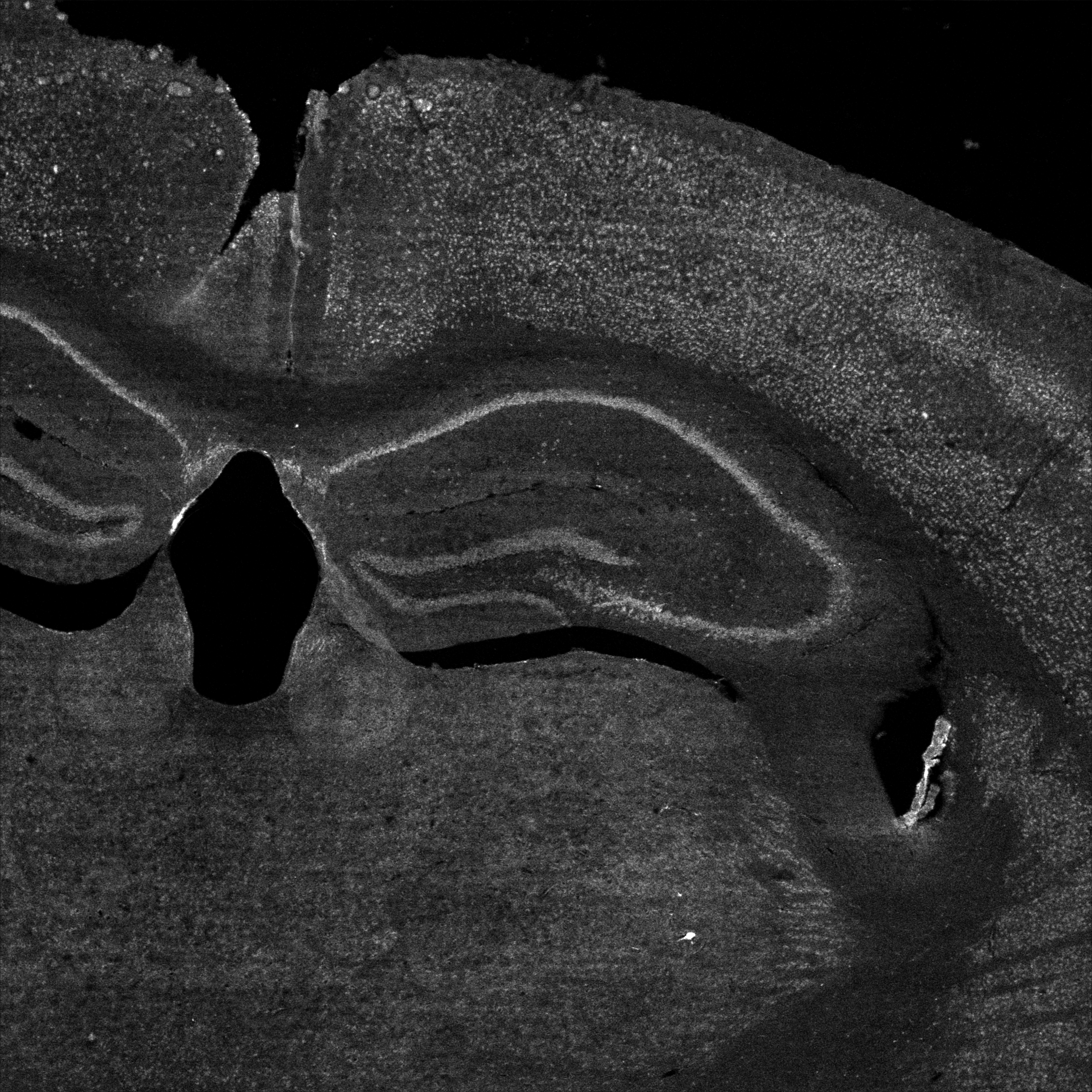

Supplement: Supplementary file 8 — Source Data for Figure 4 [file EMBR-24-e57269-s010.zip › Figure 4/4D/Rostral.2 (Bregma -1.82 mm).tif]

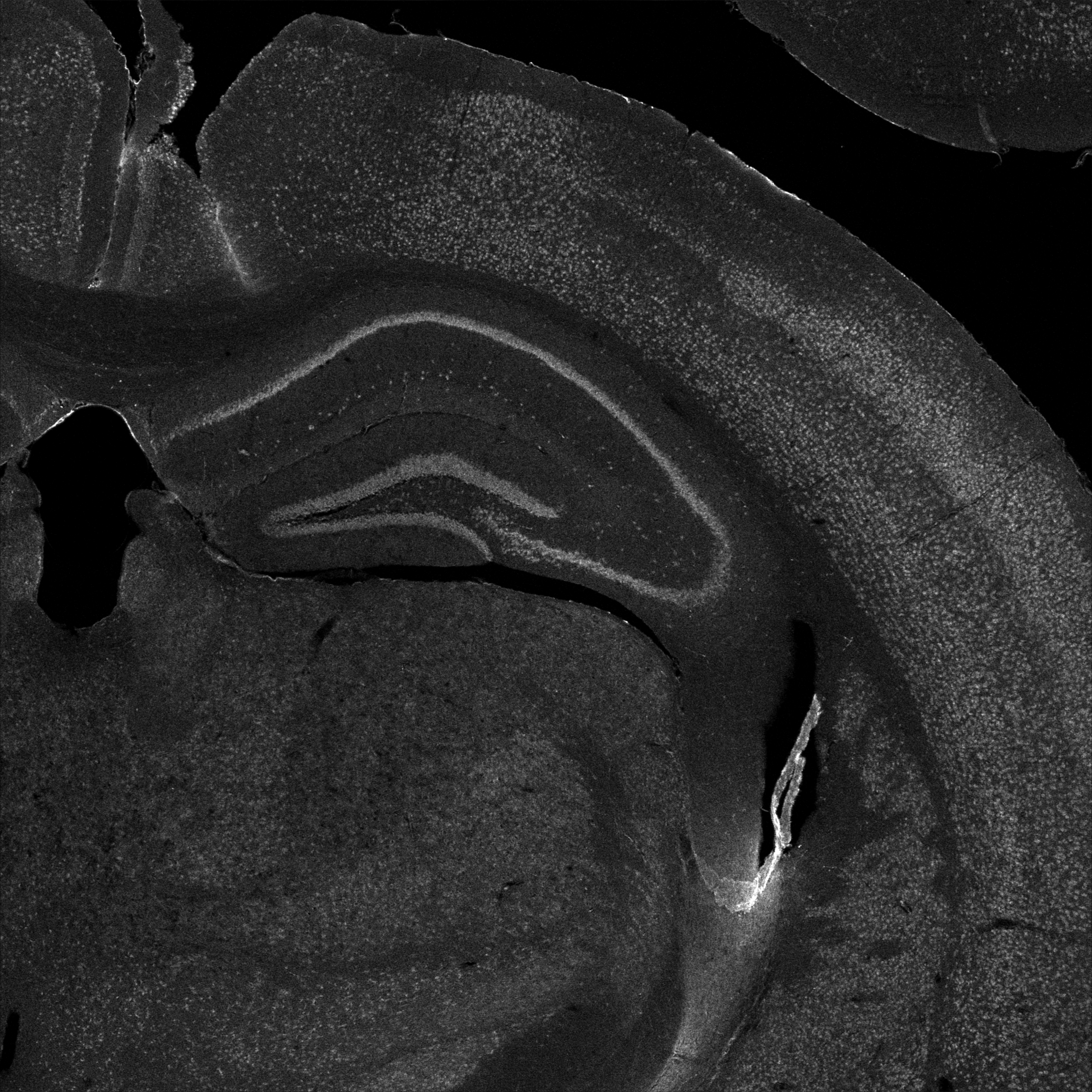

Supplement: Supplementary file 8 — Source Data for Figure 4 [file EMBR-24-e57269-s010.zip › Figure 4/4D/Rostral.3 (Bregma -2.06 mm).tif]

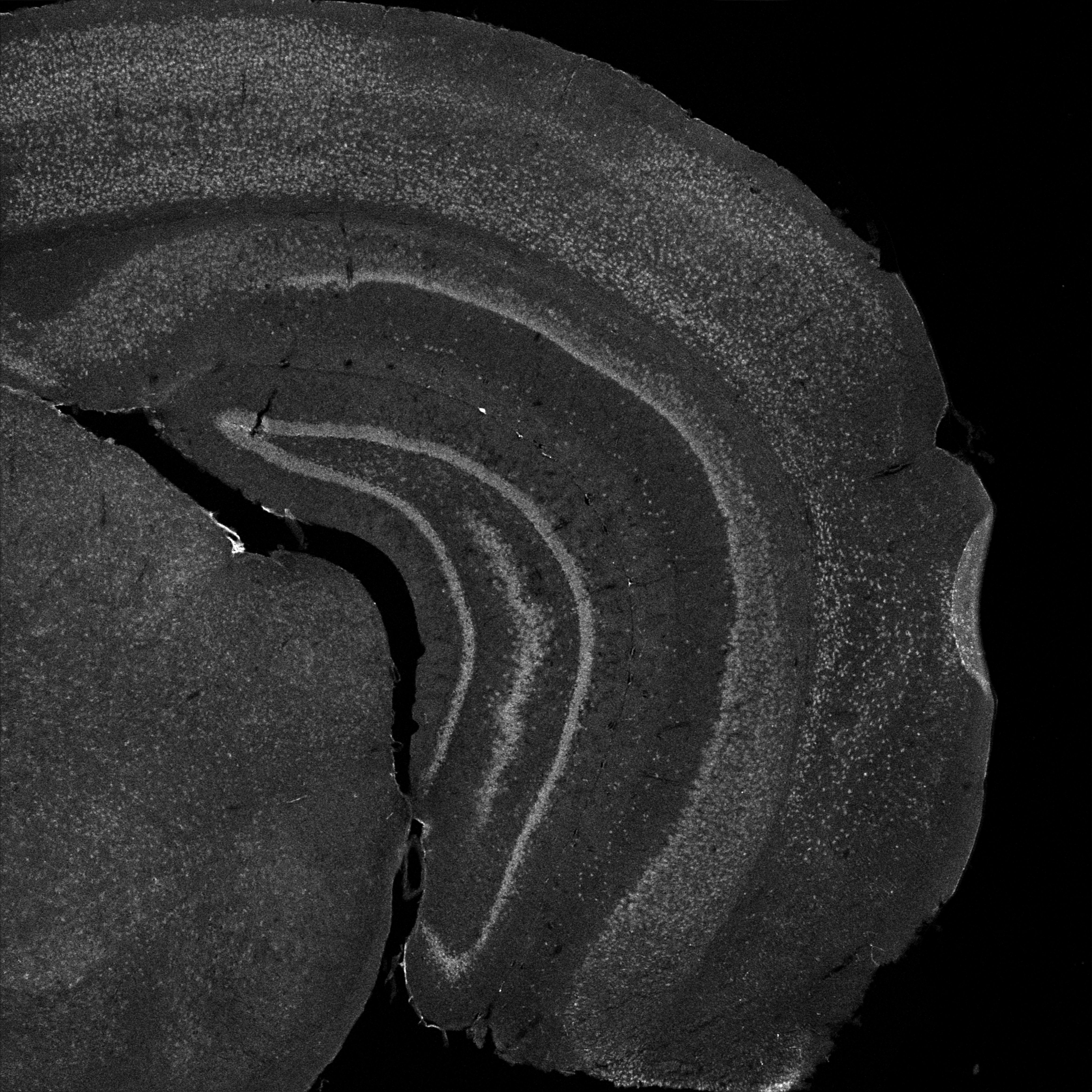

Supplement: Supplementary file 8 — Source Data for Figure 4 [file EMBR-24-e57269-s010.zip › Figure 4/4D/Caudal.3 (Bregma -3.52 mm).tif]

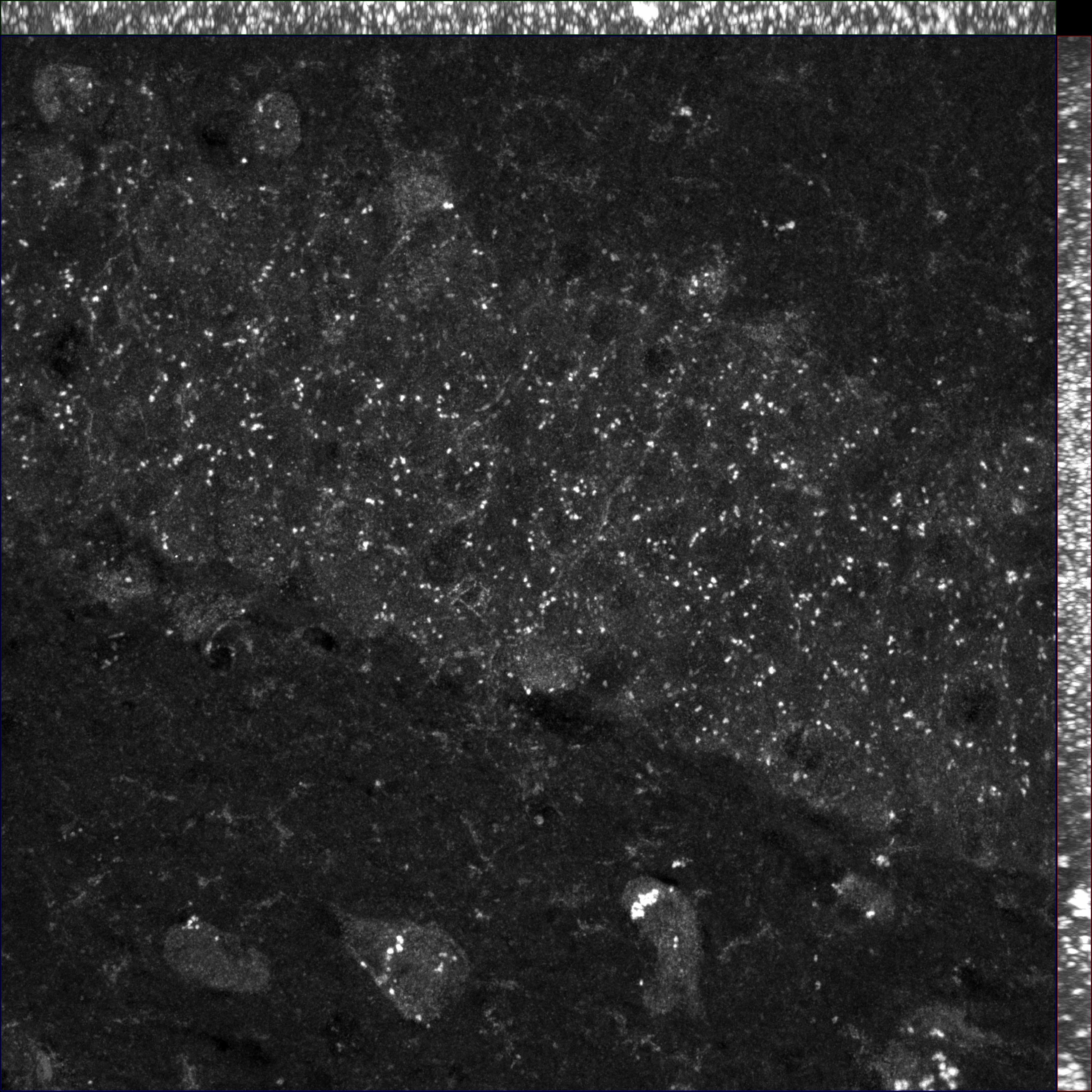

Supplement: Supplementary file 8 — Source Data for Figure 4 [file EMBR-24-e57269-s010.zip › Figure 4/4A/Adult 3D MPLX Airyscan Processed (MIP)_NeuN.tif]

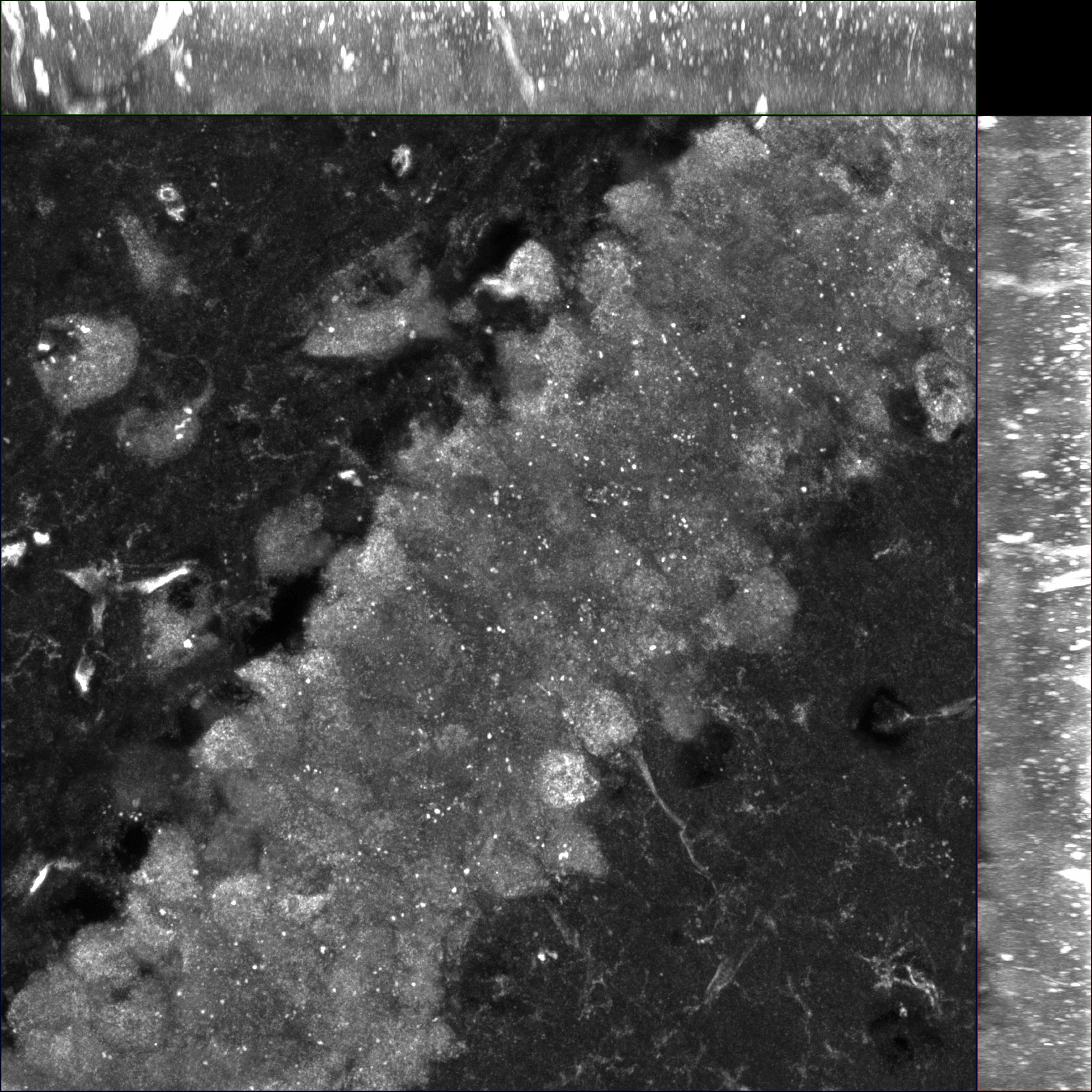

Supplement: Supplementary file 8 — Source Data for Figure 4 [file EMBR-24-e57269-s010.zip › Figure 4/4A/Adolescent 3D MPLX Airyscan Processed (MIP)_NeuN.tif]

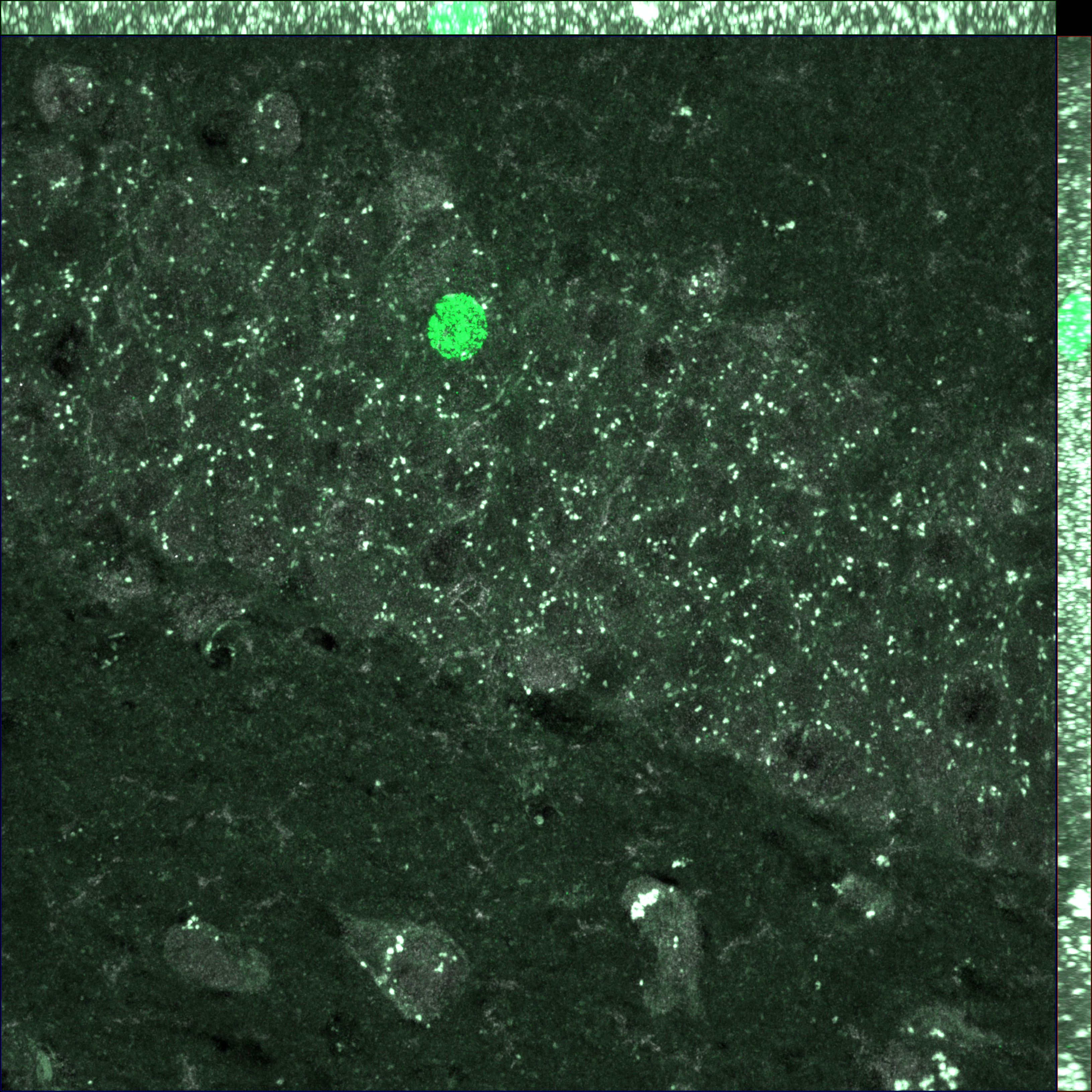

Supplement: Supplementary file 8 — Source Data for Figure 4 [file EMBR-24-e57269-s010.zip › Figure 4/4A/Adult 3D MPLX Airyscan Processed (MIP)_Merge.tif]

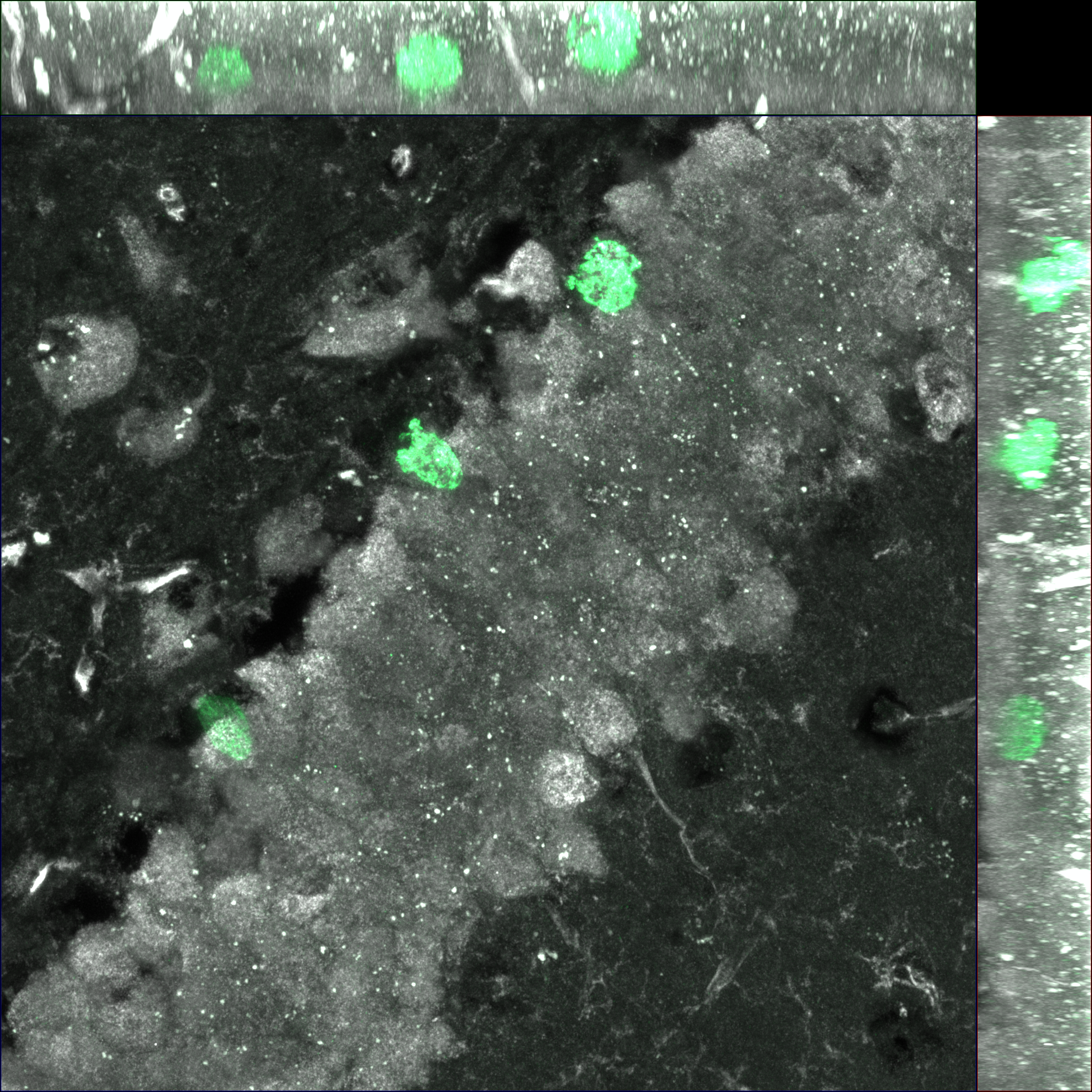

Supplement: Supplementary file 8 — Source Data for Figure 4 [file EMBR-24-e57269-s010.zip › Figure 4/4A/Adolescent 3D MPLX Airyscan Processed (MIP)_ Merge.tif]

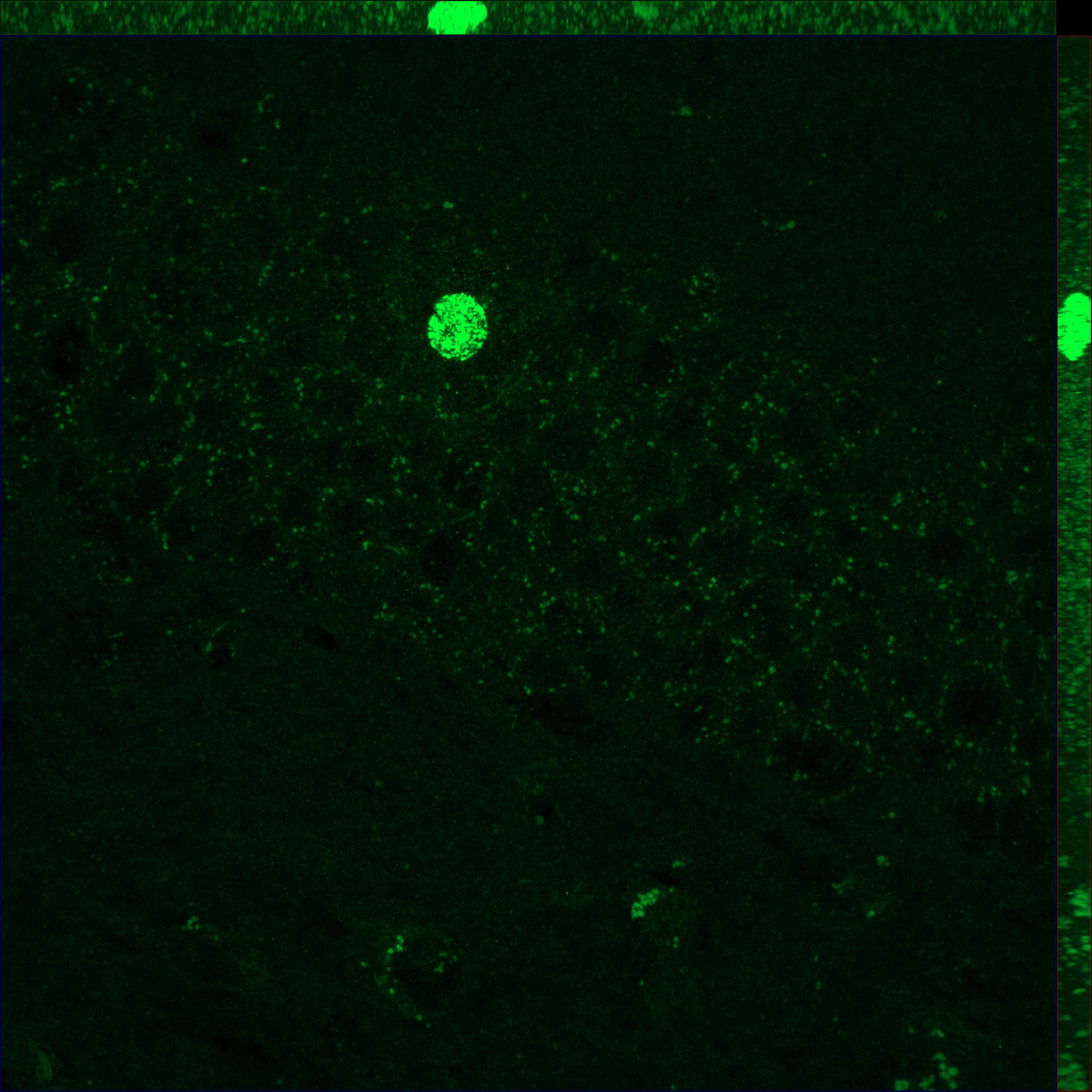

Supplement: Supplementary file 8 — Source Data for Figure 4 [file EMBR-24-e57269-s010.zip › Figure 4/4A/Adult 3D MPLX Airyscan Processed (MIP)_BrdU.tif]

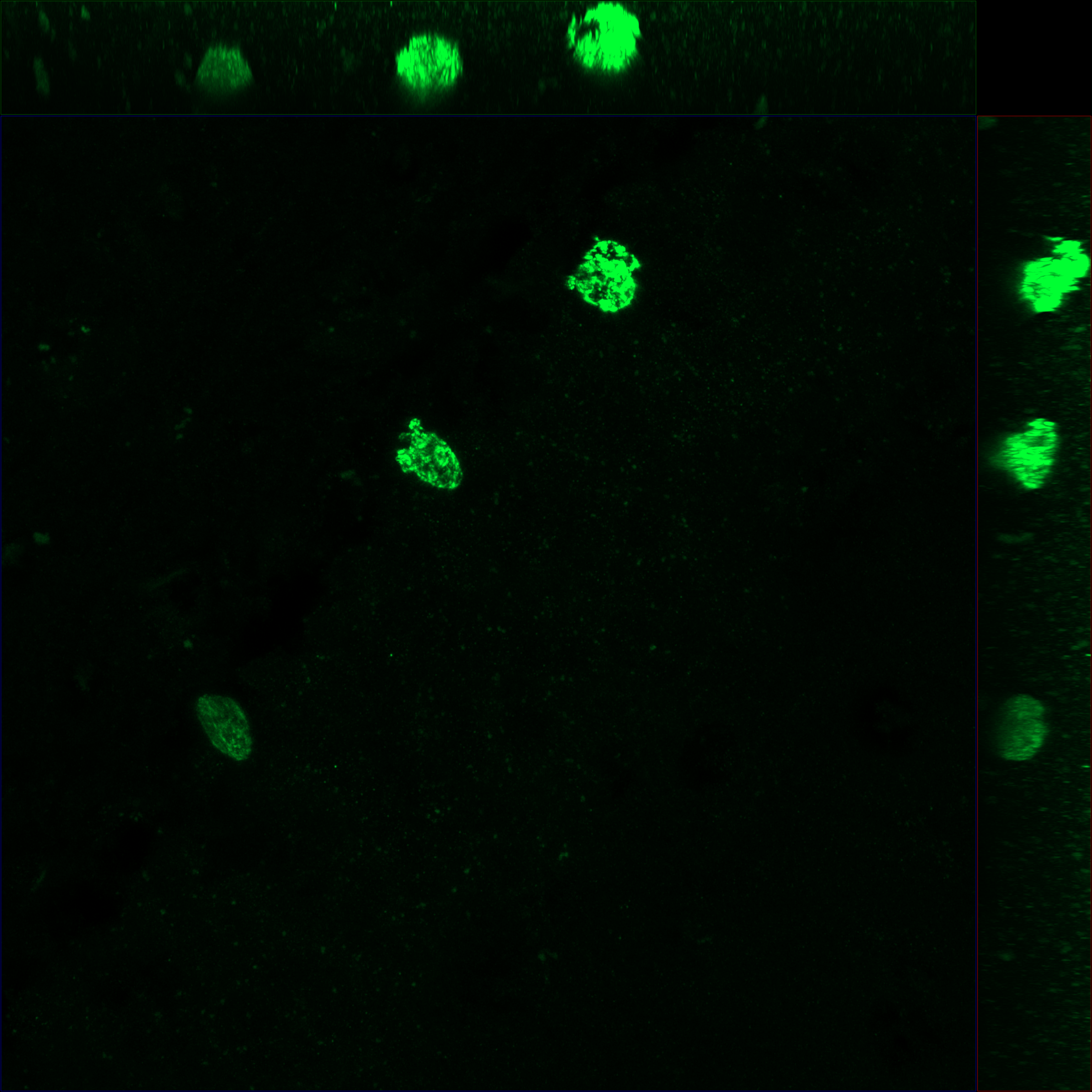

Supplement: Supplementary file 8 — Source Data for Figure 4 [file EMBR-24-e57269-s010.zip › Figure 4/4A/Adolescent 3D MPLX Airyscan Processed (MIP)_BrdU.tif]

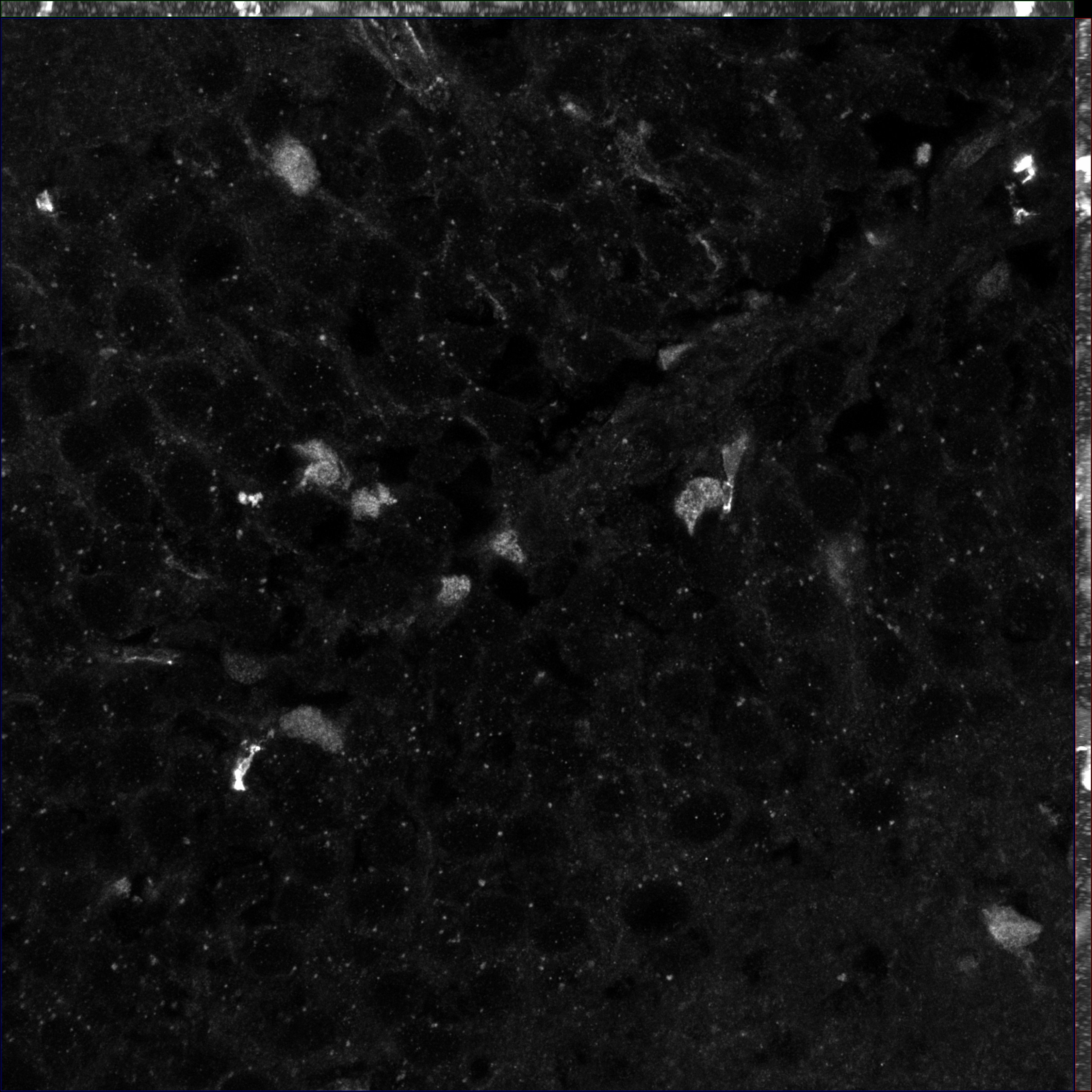

Supplement: Supplementary file 9 — Source Data for Figure 5 [file EMBR-24-e57269-s005.zip › Figure 5/5A/Adult. 3D MPLX Airyscan Processed (MIP)_S100B.tif]

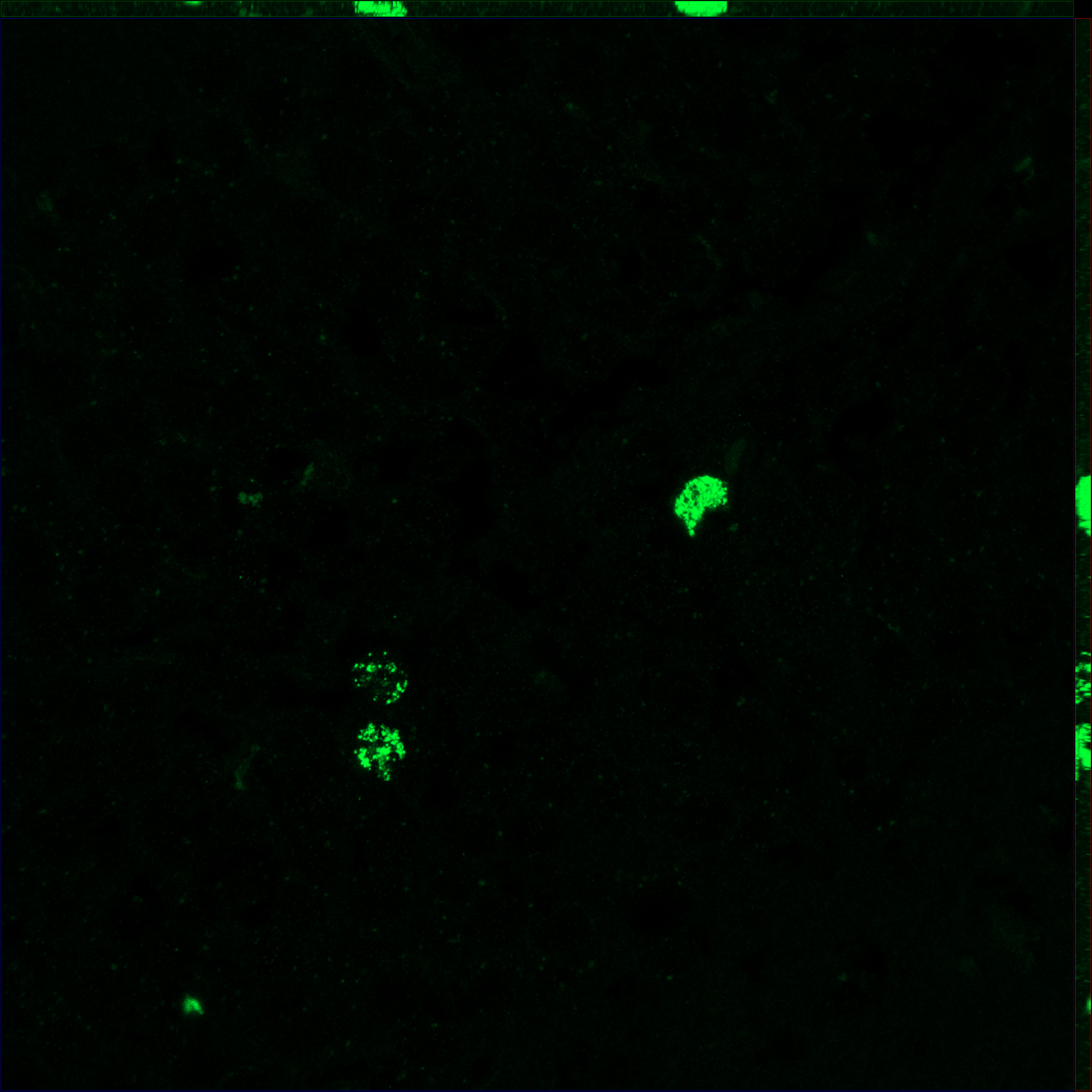

Supplement: Supplementary file 9 — Source Data for Figure 5 [file EMBR-24-e57269-s005.zip › Figure 5/5A/Adult. 3D MPLX Airyscan Processed (MIP)_BrdU.tif]

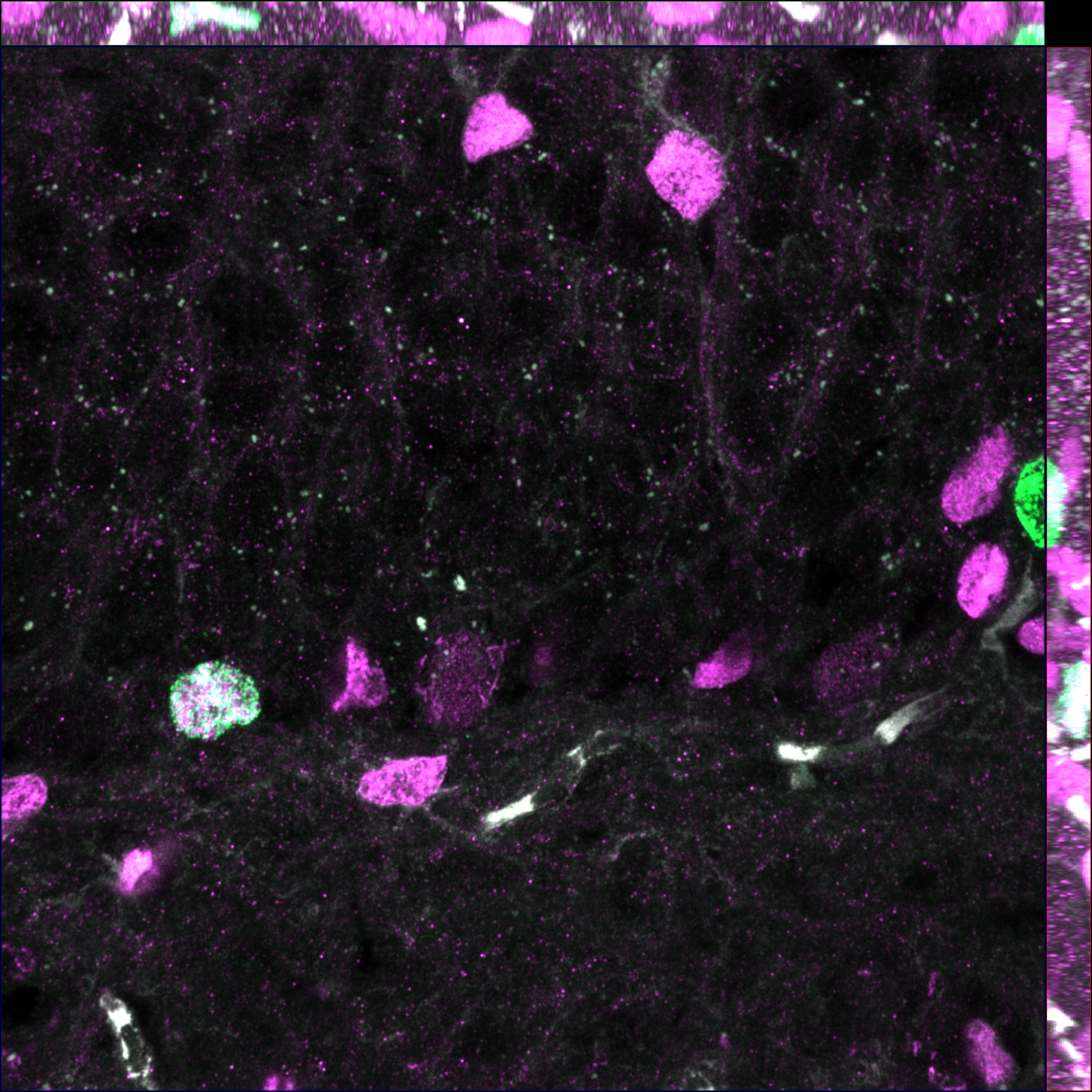

Supplement: Supplementary file 9 — Source Data for Figure 5 [file EMBR-24-e57269-s005.zip › Figure 5/5A/Adolescent. 3D MPLX Airyscan Processed (MIP)_Merge.tif]

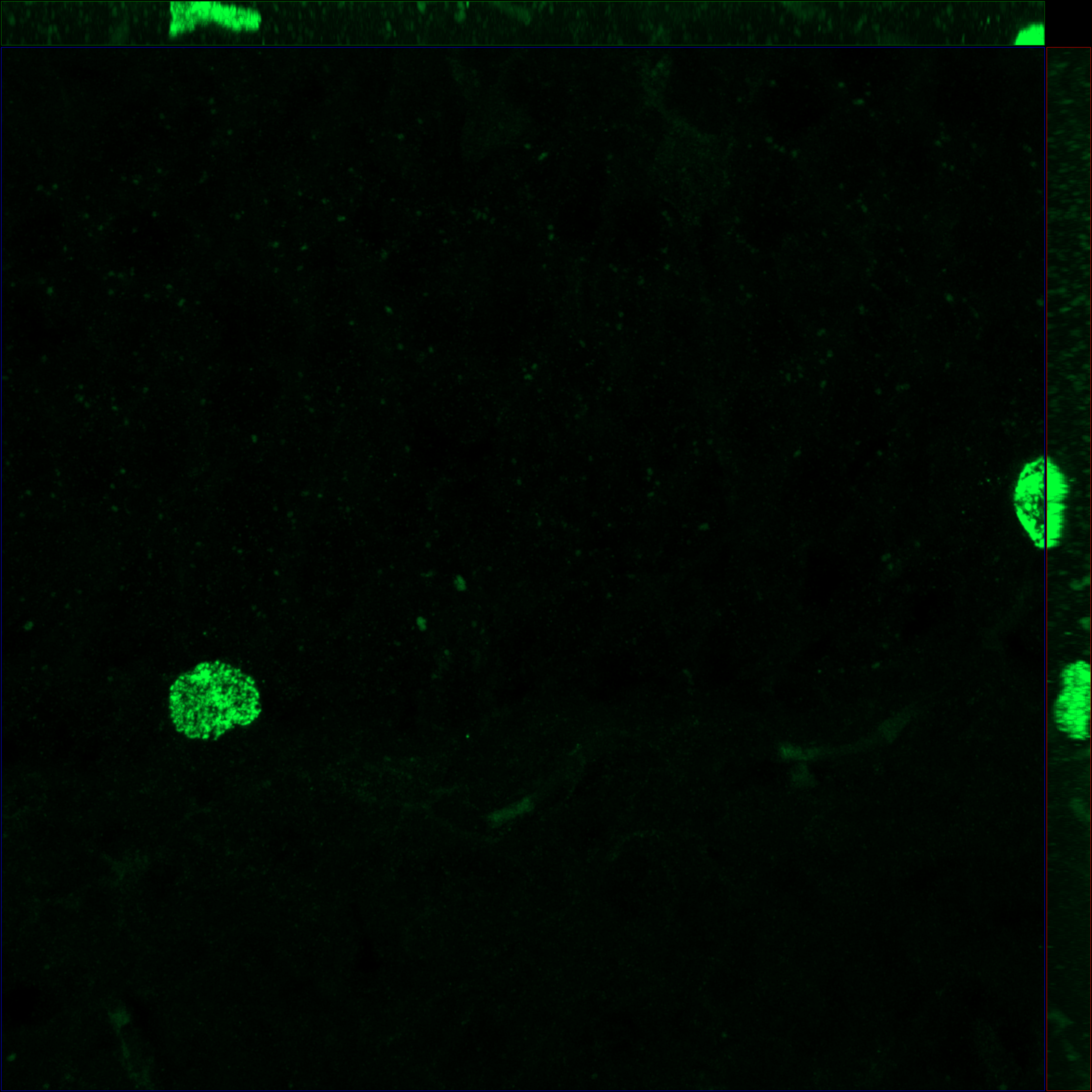

Supplement: Supplementary file 9 — Source Data for Figure 5 [file EMBR-24-e57269-s005.zip › Figure 5/5A/Adolescent. 3D MPLX Airyscan Processed (MIP)_BrdU.tif]

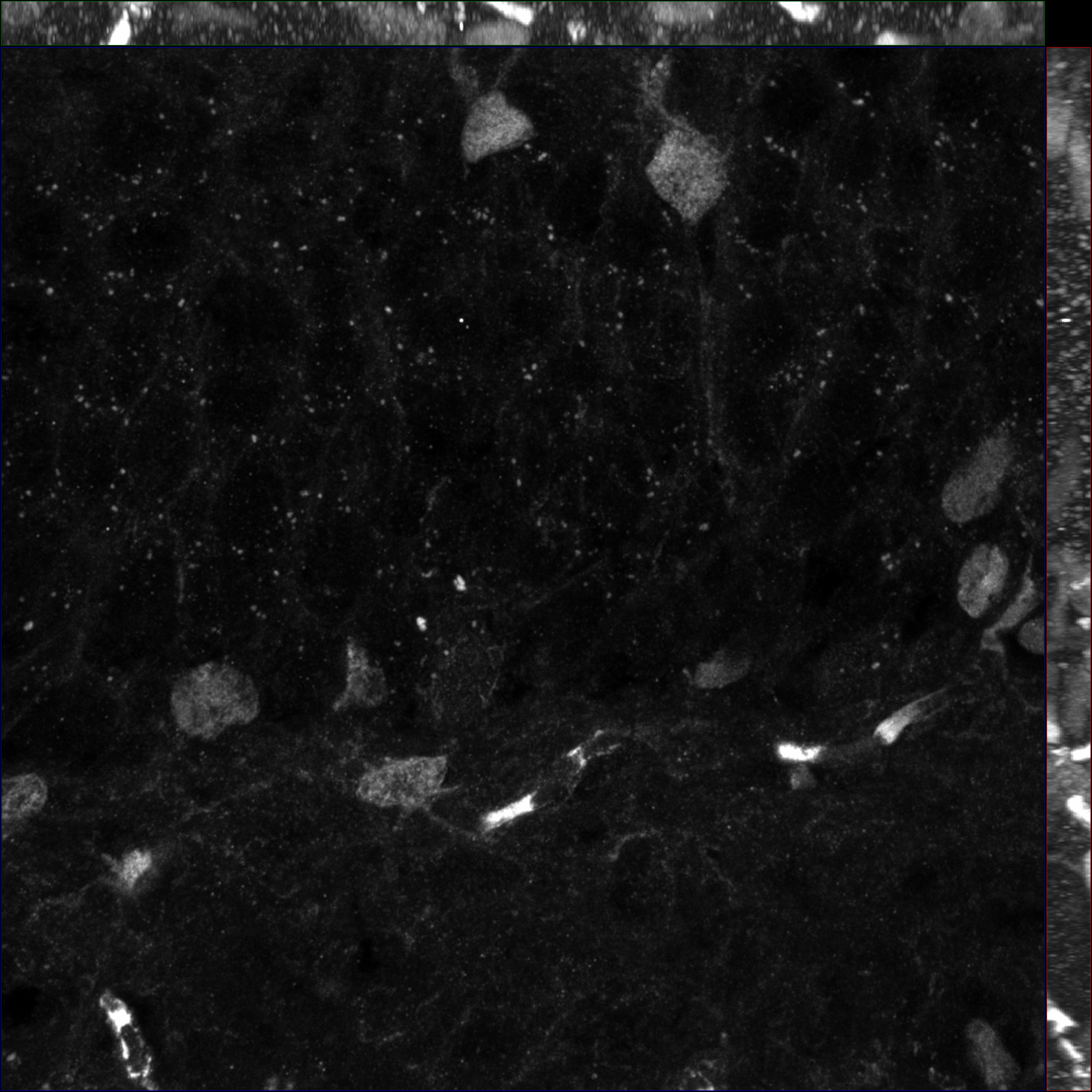

Supplement: Supplementary file 9 — Source Data for Figure 5 [file EMBR-24-e57269-s005.zip › Figure 5/5A/Adolescent. 3D MPLX Airyscan Processed (MIP)_S100B.tif]

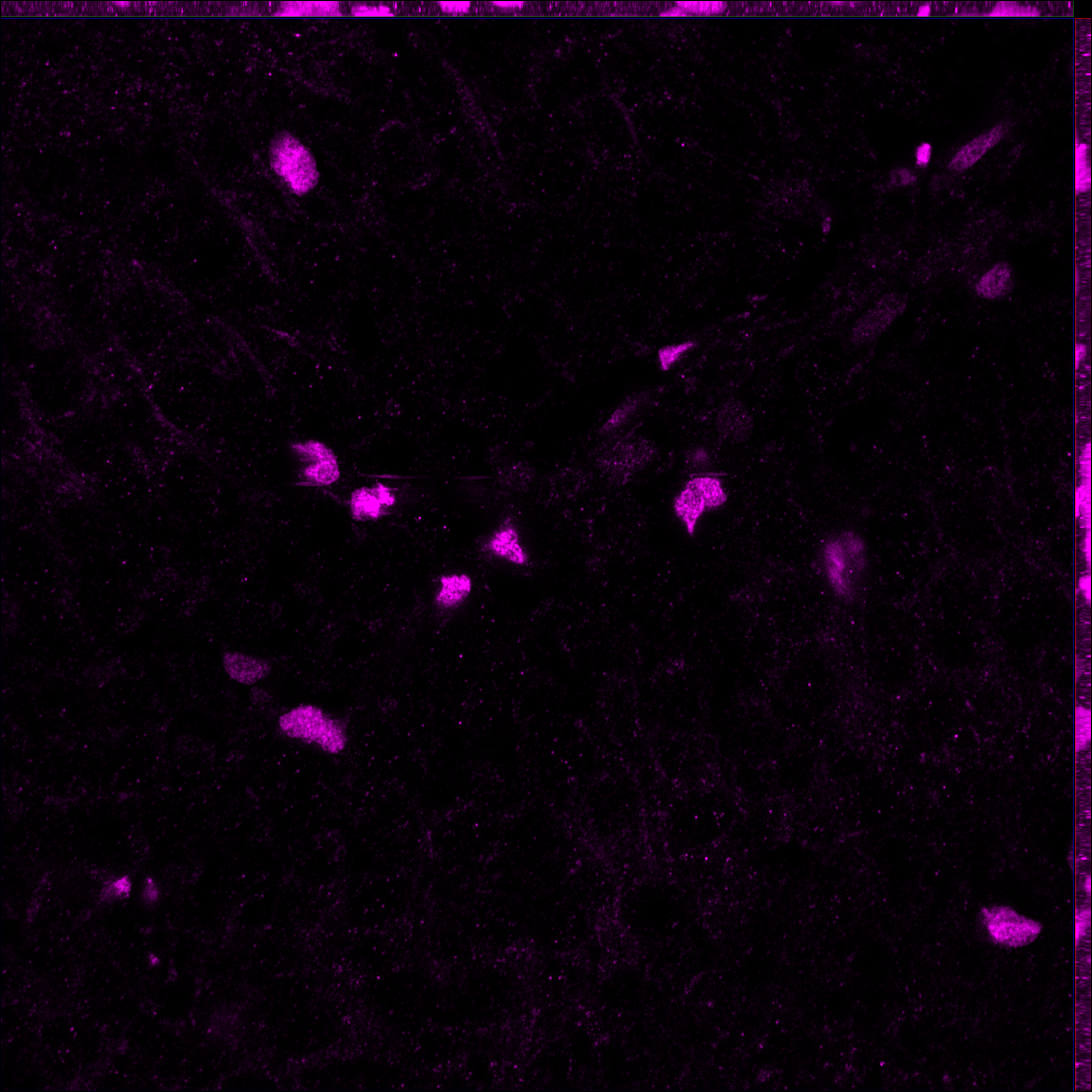

Supplement: Supplementary file 9 — Source Data for Figure 5 [file EMBR-24-e57269-s005.zip › Figure 5/5A/Adult. 3D MPLX Airyscan Processed (MIP)_Sox2.tif]

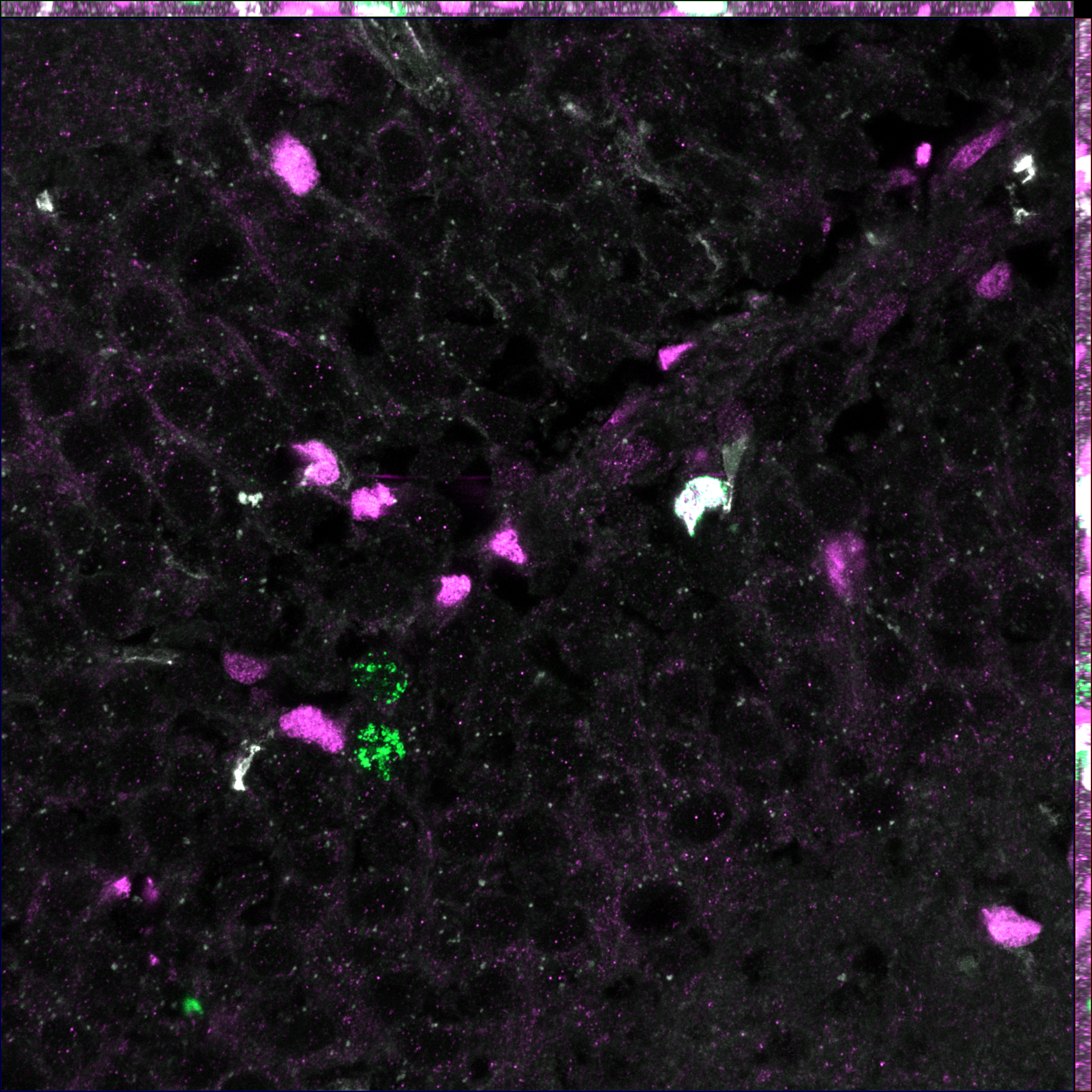

Supplement: Supplementary file 9 — Source Data for Figure 5 [file EMBR-24-e57269-s005.zip › Figure 5/5A/Adult. 3D MPLX Airyscan. Processed (MIP)_Merge.tif]

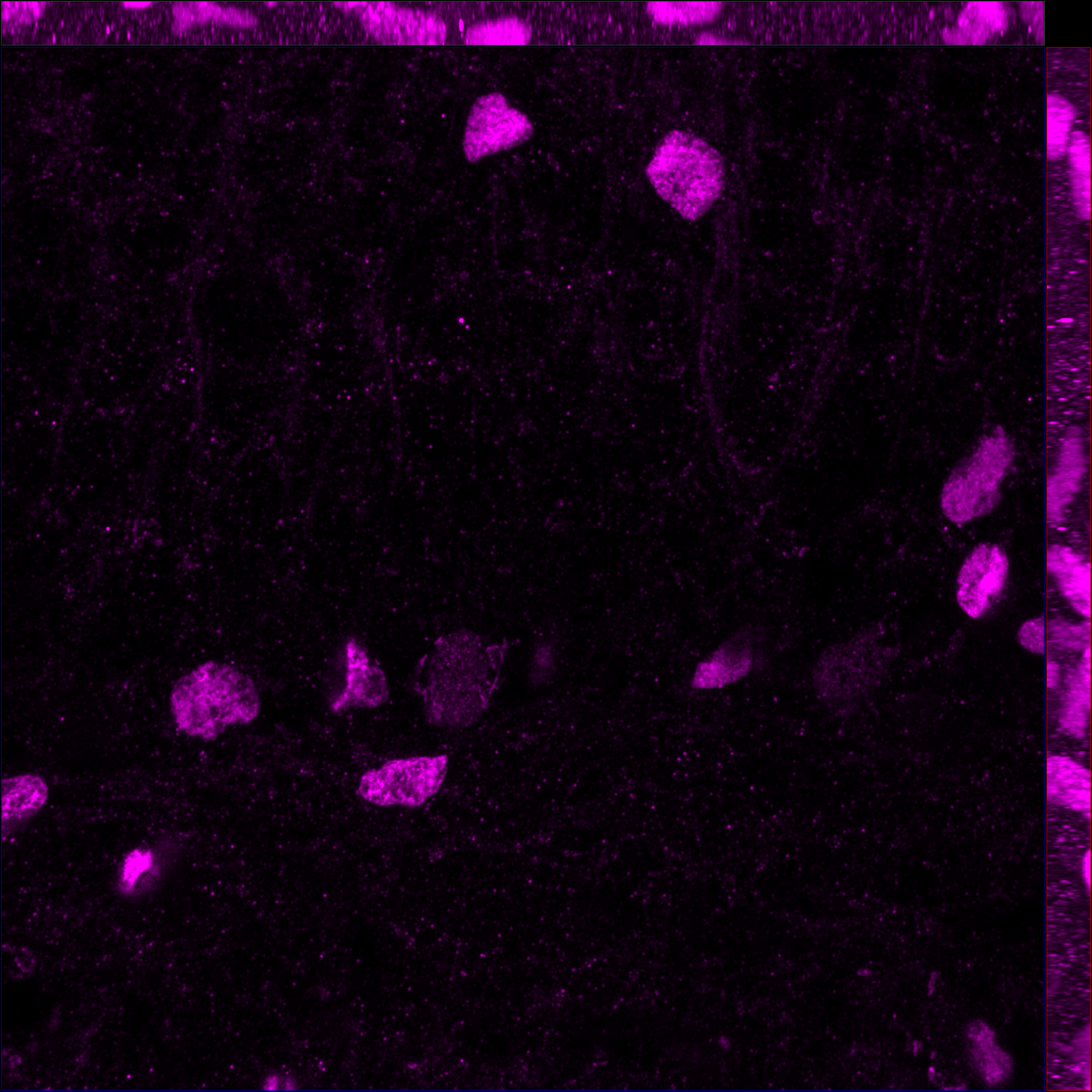

Supplement: Supplementary file 9 — Source Data for Figure 5 [file EMBR-24-e57269-s005.zip › Figure 5/5A/Adolescent. 3D MPLX Airyscan Processed (MIP)_Sox2.tif]

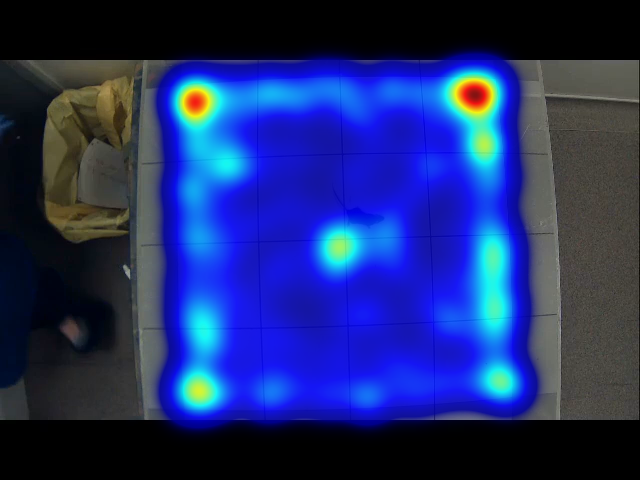

Supplement: Supplementary file 10 — Source Data for Figure 6 [file EMBR-24-e57269-s011.zip › Figure 6/6D/C57BL6J. 5.2 Diet. OF Heatmap..png]

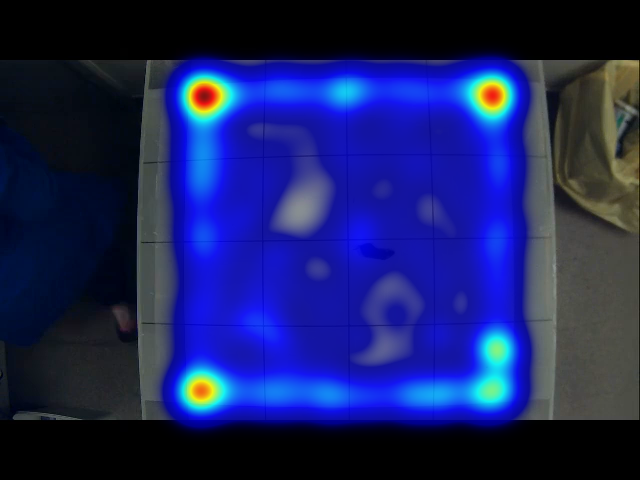

Supplement: Supplementary file 10 — Source Data for Figure 6 [file EMBR-24-e57269-s011.zip › Figure 6/6D/loxTB-GHSR. 5.2 Diet. OF Heatmap..png]

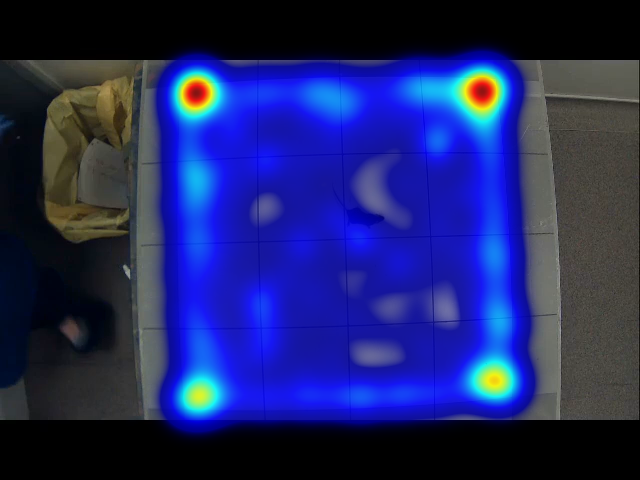

Supplement: Supplementary file 10 — Source Data for Figure 6 [file EMBR-24-e57269-s011.zip › Figure 6/6D/loxTB-GHSR. Ad libitum. OF Heatmap..png]

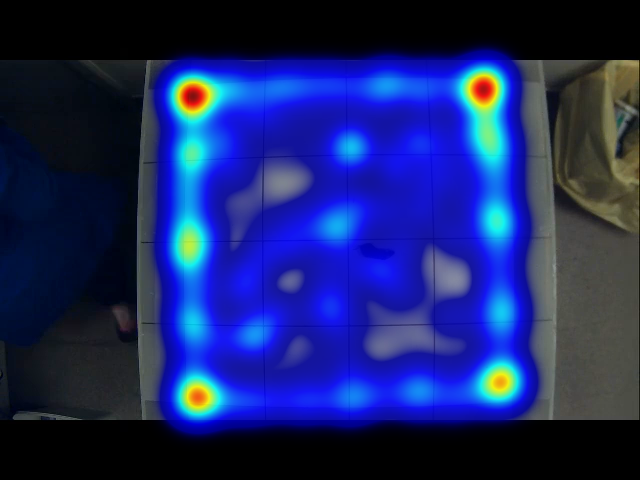

Supplement: Supplementary file 10 — Source Data for Figure 6 [file EMBR-24-e57269-s011.zip › Figure 6/6D/C57BL6J. Ad libitum. OF Heatmap..png]
