# Supplementary material for: The 5:2 diet does not increase adult hippocampal neurogenesis or enhance spatial memory in mice
Source: EMBO Rep. 2023 Nov 21;24(12):e57269. doi: 10.15252/embr.202357269 (PMC10702912; doi:10.15252/embr.202357269)
Supplement: Supplementary file 11 [file EMBR-24-e57269-s004.pdf]

EMBO reports

# The 5:2 diet does not increase adult hippocampal neurogenesis or enhance spatial memory in mice

Luke Roberts, Amanda Hornsby, Alanna Thomas, Martina Sassi, Aimee Kinzett, Nathan Hsiao, Bethan David, Mark Good, Timothy Wells, and Jeffrey Davies
**DOI: 10.15252/embr.202357269**

*Corresponding author(s): Jeffrey Davies (jeff.s.davies@swansea.ac.uk) , Timothy Wells (wellst@cardiff.ac.uk)*


## Review Timeline:

| | |
|---|---|
| Transfer from Review Commons: | 30th Mar 23 |
| Editorial Decision: | 11th Apr 23 |
| Revision Received: | 1st Sep 23 |
| Editorial Decision: | 6th Oct 23 |
| Revision Received: | 23rd Oct 23 |
| Accepted: | 30th Oct 23 |

*Editor: Ioannis Papaioannou / Deniz Senyilmaz Tiebe*


**Transaction Report: This manuscript was transferred to EMBO reports following peer review at Review Commons.**

# Review #1

## 1. Evidence, reproducibility and clarity:

### Evidence, reproducibility and clarity (Required)

In this manuscript, Roberts et al. hypothesised that the 5:2 diet (a popular form of IF, a dietary strategy within the Intermittent fasting that is thought to increase adult hippocampal neurogenesis - AHN) would enhance AHN in a ghrelin-dependent manner.
To do this, the Authors used immunohistochemistry to quantify new adult-born neurons and new neural stem cells in the hippocampal dentate gyrus of adolescent and adult wild-type mice and mice lacking the ghrelin receptor, following six weeks on a 5:2 diet. They report an age-related decline in neurogenic processes and identify a novel role for ghrelin-receptor in regulating the formation of new adult born neural stem cells in an age-dependent manner.
However, the 5:2 diet did not affect new neuron or neural stem cell formation in the dentate gyrus, nor did alter performance on a spatial learning and memory task. They conclude that the 5:2 diet used in their study does not increase AHN or improve associated spatial memory function.

**Major comments:**

I think that the key conclusions are convincing and no further experiments are required. Maybe some parts of the text should be rewritten (see below).
The methods are presented in such a way that they can be reproduced, and the experiments adequately replicated with proper statistical analysis.

- One criticism might be the fact that many aspects are addressed at the same time. For instance it is not fully clear the role of ghrelin with respect to testing the DR effects on AHN. Although the link between ghrelin, CR and AHN is explained by citing several previous studies, it is difficult to identify the main focus of the study. Maybe this is due to the fact that the Authors analyse and comment throughout the paper the different experimental approaches used by different Authors to study effect of DR to AHN. This is not bad in principle, since I think the Authors have a deep knowledge of this complex matter, but all this results in a difficulty to follow the flow of the rationale in the manuscript.
- Another major point: the Discussioni is too long. The Authors analyse all the possible reasons why different studies obtained different results concerning the effectiveness of DR in stimulating adult neurogenesis. Thus, the Discussion seems

more as a review article dealing with different methods/experimental approaches to evaluate DR effects. We know that sometimes different results are due to different experimental approaches, yet, when an effect is strong and clear, it occurs in different situations.
Thus, I think that the Authors must be less shy in expressing their conclusions, also reducing the methodological considerations. It is also well known that sometimes different results can be due to a study not well performed, or to biases from the Authors.

**Minor comments:**

- This sentence: "There is an age-related decline in adult hippocampal neurogenesis" cannot be put in the HIGHLIGHTS, since is a well known aspect of adult hippocampal neurogenesis
- Images in Figure 5 are not good quality.
- In general, there are not a lot of images refferring to microscopic/confocal photographs across the entire manuscript.
- The last sentence of the Discussion "These findings suggest that distinct DR regimens differentially regulate neurogenesis in the adult hippocampus and that further studies are required to identify optimal protocols to support cognition during ageing" is meaningless in the context of the study, and in contrast with the main results.

Honestly, my impression is that the Authors do not want to disappoint the conclusions of the previous studies; an alternative is that other Reviewers asked for this previously.

## 2. Significance:

**Significance (Required)**

The significance of this study relies on the fact that adult neurogenesis field (AN) has been often damaged by the search of "positive" results, aiming at showing that AN does occur "always and everywhere" and that most internal/external stimuli do increase it. This attitude created a bias in the field, persuading many scientists that a result in AN is worthy of publication (or of high impact factor publication) only when a positive result is found.

The Author cite in the Discussion the work by Gabarro-Solanas et al. (preprint), which share the same conclusion although analysing different aspect of neurogenesis. Both seems sound studies, substantially balanced in their conclusions.

## 3. How much time do you estimate the authors will need to complete the suggested revisions:

**Estimated time to Complete Revisions (Required)**

**(Decision Recommendation)**

Less than 1 month

## 4. *Review Commons* values the work of reviewers and encourages them to get credit for their work. Select 'Yes' below to register your reviewing activity at Web of Science Reviewer Recognition Service (formerly Publons); note that the content of your review will not be visible on Web of Science.

**Web of Science Reviewer Recognition**

No

# Review #2

## 1. Evidence, reproducibility and clarity:

**Evidence, reproducibility and clarity (Required)**

In this manuscript, Roberts et al. investigate the effect of the 5:2 diet on adult hippocampal neurogenesis (AHN) in mice via the ghrelin receptor. Many studies have reported benefits of dietary restriction (DR) on the brain that include increasing neurogenesis and enhancing cognitive function. However, neither the mechanisms underlying the effects of the 5:2 diet, nor potential benefits on the brain, are well understood. The authors hypothesize that the 5:2 diet enhances AHN and cognitive function via ghrelin-receptor signaling. To test this, they placed adolescent and adult ghrelin receptor knockout or wild type mice on either the 5:2 or ad libitum (AL) diet for 6 weeks, followed by spatial memory testing using an object in place (OIP) task. The authors also assessed changes in AHN via IHC using multiple markers for cell proliferation and neural stem cells. The authors observed a decrease in AHN due to

age (from adolescent to adult), but not due to diet or ghrelin-receptor signaling. While loss of the ghrelin-receptor impaired spatial memory, the 5:2 diet did not affect cognitive function. The authors conclude that the 5:2 diet does not enhance AHN or spatial memory.

**Major Points**

1. The authors use a 5:2 diet but fail to provide a basic characterization of this dietary intervention. For example, was the food intake assessed? In addition to the time restriction of the feeding, does this intervention also represent an overall caloric restriction or not? According to the provided results, the 5:2 diet does not appear to regulate adult hippocampal neurogenesis contrary to the authors' original hypothesis. Did the authors measure the effects of the 5:2 diet on any other organ system? Do they have any evidence that the intervention itself resulted in any well documented benefits in other cell types? Such data would provide a critical positive control for their intervention.
2. Based on the effects of grehlin in other dietary interventions, the authors speculate that the effect of the 5:2 diet is similarly mediated through grehlin. However, the authors do not provide any basic characterization of grehlin signaling to warrant this strong focus on the GSH-R mice. While the GSH-R mice display changes in NSC homeostasis and neurogenesis, none of these effects appear to be modified by the 5:2 diet. Thus, the inclusion of the GSH-R mice does not seem warranted and detracts from the main 5:2 diet focus of the manuscript.
3. Neurogenesis is highly sensitive to stress. The 5:2 diet may be associated with stress which could counteract any benefits on neurogenesis in this experimental paradigm. Did the authors assess any measures of stress in their cohorts? Were the mice group housed or single housed?
4. The authors state that the 5:2 diet led to a greater reduction in body weight (31%) in adolescent males compared to other groups. However, it appears that the cohorts were not evenly balanced and the adolescent 5:2 male mice started out with a significantly higher starting weight (Supplementary Figure 1). The difference in starting weight at such a young age is significantly confounding the conclusion that the 5:2 diet is more effective at limiting weight gain specifically in this group.
5. The authors count NSCs as Sox2+S100b- cells. However, the representative S100b staining does not look very convincing. Instead, it would be more appropriate to count Sox2+GFAP+ cells with a single vertical GFAP+ projection. Alternatively, the authors could also count Nestin-positive cells. Additionally, the authors label BrdU+ Sox2+ S100B- cells as "new NSCs". However, it appears that the BrdU labeling was performed approximately 6 weeks before the tissue was collected (Figure 1A). Thus, these BrdU-positive NSCs most likely represent label-retaining/quiescent NSCs that divided during the labeling 6 weeks prior but have not proliferated since. As such, the

term "new NSC" is misleading and would suggest an NSC that was actively dividing at the time of tissue collection.
6. Overall, this manuscript lacks a clear focus and narrative. Due to a lack of an affect by the 5:2 diet on hippocampal neurogenesis, the authors mostly highlight already well-known effects of aging and Grehlin/GSH-R on neurogenesis. Moreover, the authors repeatedly use age-related decline and morbidities as a rational for their study. However, they assess the effects of the 5:2 diet on neurogenesis only in adolescent and young mature but not aged mice.

**Minor Points**

1. The authors combine the data from both male and female mice for most bar graphs. While this does not appear to matter for neurogenesis or behavioral read-outs, there are very significant sexually dimorphic differences with respect to body size and weight. As such, male and female mice in Figure 1D,F should not be plotted in the same bar graph.
2. The Figure legends are very brief and should be expanded to include basic information of the experimental design, statistical analyses etc.
3. Many figures include a representative image. However, it is often unclear if that is a representative image of a WT or mutant mouse, or a 5:2 or control group (Figure 2A, 3A, 4A, 5A).
4. It would be helpful to provide representative images of DCX-positive cells in Figure 3A-F. Additionally, the authors should include a more extensive description of how this quantification was performed in the method section.
5. The authors state "the hippocampal rostro-caudal axis (also known as the dorso-ventral [] axis". However, the rostral-caudal and dorsal-central axis are usually considered perpendicular to one another.

# 2. Significance:

### Significance (Required)

Understanding the mechanisms of a popular form of intermittent fasting (5:2 diet) that is not well understood is an interesting topic. Moreover, examining the effect of this form of intermittent fasting on the brain is timely. Notwithstanding, while the authors use multiple markers to validate the effect of the 5:2 diet on adult hippocampal neurogenesis, concerns regarding experimental design, validation, and data analysis weaken the conclusions being drawn.

### 3. How much time do you estimate the authors will need to complete the suggested revisions:

**Estimated time to Complete Revisions (Required)**

**(Decision Recommendation)**

Between 3 and 6 months

### 4. *Review Commons* values the work of reviewers and encourages them to get credit for their work. Select 'Yes' below to register your reviewing activity at Web of Science Reviewer Recognition Service (formerly Publons); note that the content of your review will not be visible on Web of Science.

**Web of Science Reviewer Recognition**

Yes

# Review #3

## 1. Evidence, reproducibility and clarity:

**Evidence, reproducibility and clarity (Required)**

In this study, Roberts and colleagues used a specific paradigm of intermitted fasting, the 5:2 diet, meaning 5 days ad libitum food and 2 non-consecutive days of fasting. They exposed adolescent and adult wildtype mice and ghrelin receptor knockout mice (GHS-R-/-) for 6 weeks to this paradigm, followed by 1 week ad libitum food. They further used the "object in place task" (OIP) to assess spatial memory performance. At the end of the dietary regime, the authors quantified newborn neurons and neural stem cells (NSCs) by immunohistochemistry. Roberts et al. show that the 5:2 diet does not change the proliferation of cells in the hippocampus, but report an increased number of immature neurons (based on DCX) in all the mice exposed to the 5:2 diet. This change however did not result in an increased number of mature adult-born neurons, as assessed by a BrdU-birthdating paradigm. The authors further show diet-

independent effects of the ghrelin receptor knockout, leading to less adult born neurons, but more NSCs in the adolescent mice and a lower performance in the OIP task.

**Major comments:**

The main conclusion of this study is that a specific type of intermitted fasting (5:2 diet) has no effects on NSC proliferation and neurogenesis. As there are several studies showing beneficial effects of intermitted fasting on adult neurogenesis, while other studies found no effects, it is important to better understand the effects of such a dietary paradigm.

The experimental approaches used in this manuscript are mostly well explained, but it is overall rather difficult to follow the results part, as the authors always show the 4 experimental groups together (adolescent vs adult and wt vs GHS-R-/-). They highlight the main effects comparing all the groups, which most of the time is the factor "age". Age is a well-known and thus not surprising negative influencer of adult neurogenesis. Instead of focusing on the main tested factor, namely the difference in diet, the authors show example images of the two age classes (adolescent vs adult), which does not underly the major point they are making. Most of the time, they do not provide a post hoc analysis, so it is difficult to judge if the results with a significant main effect would be significant in a direct 1 to 1 comparison of the corresponding groups. The authors point out themselves that previous rodent studies did not use such a 5:2 feeding pattern, so having diet, age and genotype as factors at the same time makes the assessment of the diet effect more difficult.
The manuscript would improve if the authors restructure their data to compare first the diet groups (adolescent wt AL vs 5:2 and in a separate comparison adult wt AL vs 5:2) and only in a later part of the results check if the Ghrelin receptor plays a role or not in this paradigm.

As this 5:2 is a very specific paradigm, it is furthermore difficult to compare these results to other studies and the conclusions are only valid for this specific pattern and timing of the intervention (6 weeks). It remains unclear why the authors have not first tried to establish a study with wildtype mice and a similar duration as in previous studies observing beneficial effects of intermitted fasting on neurogenesis. Like this, it would have been possible to make a statement if the 5:2 per se does not increase neurogenesis or if the 6 weeks exposure were just too short.

The graphical representation of the data could also be improved. Below are a few examples listed:

1. Figure 1 B and C, the same symbol and colours are used for the adolescent and adult animals, which makes the graphs hard to read. One colour and symbol per group throughout the manuscript would be better.
2. The authors found no differences in the total number of Ki67 positive cells in the DG. However, Ki67 staining does not allow to conclude the type of cell which is proliferating. It would thus strengthen the findings if this analysis was combined with different markers, such as Sox2, GFAP and DCX.
3. In Figure 3, the authors say that the diet increases the number of DCX in adolescent and adult mice, which is not clear when looking at the graph in 3B. Are there any significant differences when directly comparing the corresponding groups, for instance the WT AL vs the WT 5:2? It is further not clear how the authors distinguished the different types of DCX morphology-wise. The quantification in C and D would need to be illustrated by example images. Furthermore, the colour-code used in these graphs is not explained and remains unclear.
4. In Figure 5, the authors show that the number of new NSCs is significantly increased in the adolescent GHS-R-/- mice, independent of the diet, but this increase does not persist in the adult mice. They conclude that "the removal of GHS-R has a detrimental effect on the regulation of new NSC number..." this claim is not substantiated and needs to be reformulated. As the GHS-R-/- mice have a transcriptional blockage of Ghrs since start of its expression, would such an effect on NSC regulation not result in an overall difference in brain development, as ghrelin is also important during embryonic development?
5. In Figure 6, the authors asses spatial memory performance with a single behavioral test, the OIP. As these kind of tests are influenced by the animal's motivation to explore, it's anxiety levels, physical parameters (movement) etc., the interpretation of such a test without any additional measured parameters can be problematic. The authors claim that the loss of GHS-R expression impairs spatial memory performance. As the discrimination ratio was calculated, it is not possible to see if there is an overall difference in exploration time between genotypes. This would be a good additional information to display.

Besides these points listed above, the methods are presented in such a way that they can be reproduced. The experiments contained 10-15 mice per group, which is a large enough group to perform statistical analyses. As mentioned above, the statistical analysis over all 4 groups with p-values for the main effects should be followed by post hoc multiple comparison tests to allow the direct comparison of the corresponding groups.

**Minor comments:**

The authors should provide more information in the figure legends and always show

representative images of the parameters analyzed. Some of the images are also of low resolution and should be replaced with higher resolution images (for instance Fig. 5A). The significant P values of the multiple comparison between groups should be added into the figures.

## 2. Significance:

**Significance (Required)**

In the last years, growing evidences suggested that IF might have positive effect on health in general and also for neurogenesis. However, a few recent studies report no effects on neurogenesis, using different IF paradigms. This study adds another proof that not all IF paradigms influence neurogenesis and shows that more work needs to be done to better understand when and how IF can have beneficial effects. This is an important finding for the neurogenesis field, but the results are only valid for this specific paradigm used here, which limits its significance. The reporting of such negative findings is however still important, as it shows that IF is not just a universal way to increase neurogenesis. In the end, such findings might have the potential to bring the field together to come up with a more standardized dietary intervention paradigm, which would be robust enough to give similar results across laboratories and mouse strains, and would allow to test the effect of genetic mutations on dietary influences of neurogenesis.

## 3. How much time do you estimate the authors will need to complete the suggested revisions:

**Estimated time to Complete Revisions (Required)**

**(Decision Recommendation)**

Between 1 and 3 months

## 4. *Review Commons* values the work of reviewers and encourages them to get credit for their work. Select 'Yes' below to register your reviewing activity at Web of Science Reviewer Recognition Service (formerly Publons); note that the content of your review will not be visible on Web of Science.

**Web of Science Reviewer Recognition**

No

# Revision Plan

**Manuscript number:** RC-2022-01714
**Corresponding author(s):** Jeffrey, Davies

## 1. General Statements [optional]

*This section is optional. Insert here any general statements you wish to make about the goal of the study or about the reviews.*

## 2. Description of the planned revisions

We provide here a point-by-point response to the reviewer's comments, with our answers and comments in blue.

**Reviewer #1** (Evidence, reproducibility and clarity (Required)):

**Summary:**
In this manuscript, Roberts et al. hypothesised that the 5:2 diet (a popular form of IF, a dietary strategy within the Intermittent fasting that is thought to increase adult hippocampal neurogenesis - AHN) would enhance AHN in a ghrelin-dependent manner. To do this, the Authors used immunohistochemistry to quantify new adult-born neurons and new neural stem cells in the hippocampal dentate gyrus of adolescent and adult wild-type mice and mice lacking the ghrelin receptor, following six weeks on a 5:2 diet. They report an age-related decline in neurogenic processes and identify a novel role for ghrelin-receptor in regulating the formation of new adult born neural stem cells in an age-dependent manner. However, the 5:2 diet did not affect new neuron or neural stem cell formation in the dentate gyrus, nor did alter performance on a spatial learning and memory task. They conclude that the 5:2 diet used in their study does not increase AHN or improve associated spatial memory function.

**Major comments:**
One criticism might be the fact that many aspects are addressed at the same time. For instance it is not fully clear the role of ghrelin with respect to testing the DR effects on AHN. Although the link between ghrelin, CR and AHN is explained by citing several previous studies, it is difficult to identify the main focus of the study. Maybe this is due to the fact that the Authors analyse and comment throughout the paper the different experimental approaches used by different Authors to study effect of DR to AHN. This is not bad in principle, since I think the Authors have a deep knowledge of this complex matter, but all this results in a difficulty to follow the flow of the rationale in the manuscript.

We appreciate the reviewer's critique regarding the rationale of the studies presented in the manuscript.

The role of ghrelin in the regulation of AHN by dietary interventions such as CR and IF is a major interest of our lab and is the main focus of the study. We, and others, have shown that ghrelin mediates the beneficial effects of CR on AHN. It is often assumed that ghrelin will elicit similar effects in other DR paradigms. We selected the 5:2 diet since it is widely practiced by humans, but it has not been well tested experimentally.
We sought to empirically test how the neurogenic response to 5:2 differed between mice with functional and impaired ghrelin signaling.
Given that plasma ghrelin levels and AHN are reduced during ageing, we also wanted to determine if 5:2 diet could slow or even prevent neurogenic decline in ageing mice.
We will re-write the manuscript to ensure that our primary aim is clearly presented. We will also reanalyze the data, with genotype and 5:2 diet as key variables. To help maintain focus, the variable of age will be analyzed separately. This amendment will, we hope, help the reader follow the narrative of our manuscript.

Another major point: the Discussion is too long. The Authors analyse all the possible reasons why different studies obtained different results concerning the effectiveness of DR in stimulating adult neurogenesis. Thus, the Discussion seems more as a review article dealing with different methods/experimental approaches to evaluate DR effects. We know that sometimes different results are due to different experimental approaches, yet, when an effect is strong and clear, it occurs in different situations. Thus, I think that the Authors must be less shy in expressing their conclusions, also reducing the methodological considerations. It is also well known that sometimes different results can be due to a study not well performed, or to biases from the Authors.

In our discussion, we felt that it was particularly important to be as rigorous as possible in contextualizing our findings with other published data, whilst highlighting methodological differences. Our aim was to be as precise as possible when comparing findings across studies, however, this resulted in the narrative drifting from the key objectives of our study – namely, to determine the effect of 5:2 diet on neurogenesis and whether or not ghrelin-signalling regulated the process. We will amend the text of the discussion to ensure that the key points of our study are only compared and contrasted with relevant studies in the field. We thank the reviewer for their candid comment.

**Minor comments:**

- This sentence: "There is an age-related decline in adult hippocampal neurogenesis" cannot be put in the HIGHLIGHTS, since is a well known aspect of adult hippocampal neurogenesis

The reviewer is correct to state this. Our study replicates this interesting age-related phenomenon. However, we will remove it from the 'Highlights' section.

- Images in Figure 5 are not good quality.

# Revision Plan

We apologise for this oversight. We will review each figure and panel to ensure that high-resolution images, that are appropriately annotated, are used throughout the manuscript.

- In general, there are not a lot of images referring to microscopic/confocal photographs across the entire manuscript.

We structured the manuscript with a limited number of figures and associated microscope captured panels, with the aim of presenting representative images to illustrate the nature and quality of the IHC protocols. However, we will amend the figures for the revised manuscript to provide representative microscopy images, with each group included and clearly annotated.

- The last sentence of the Discussion "These findings suggest that distinct DR regimens differentially regulate neurogenesis in the adult hippocampus and that further studies are required to identify optimal protocols to support cognition during ageing" is meaningless in the context of the study, and in contrast with the main results. Honestly, my impression is that the Authors do not want to disappoint the conclusions of the previous studies; an alternative is that other Reviewers asked for this previously.

We do not believe that this statement is contradictory to our findings, as distinct DR paradigms do appear to regulate AHN in different ways. However, we agree that we can be more explicit with regards to our own study findings and will prioritize the conclusions of our study over those of the entire field during revision.

**Reviewer #1 (Significance (Required)):**

The significance of this study relies on the fact that adult neurogenesis field (AN) has been often damaged by the search of "positive" results, aiming at showing that AN does occur "always and everywhere" and that most internal/external stimuli do increase it. This attitude created a bias in the field, persuading many scientists that a result in AN is worthy of publication (or of high impact factor publication) only when a positive result is found.
The Author cite in the Discussion the work by Gabarro-Solanas et al. (preprint), which share the same conclusion although analysing different aspect of neurogenesis.
Both seems sound studies, substantially balanced in their conclusions.

We appreciate the reviewer's comment on publication bias and that they value the significance of publishing studies that will advance the field.

**Reviewer #2 (Evidence, reproducibility and clarity (Required)):**

In this manuscript, Roberts et al. investigate the effect of the 5:2 diet on adult hippocampal neurogenesis (AHN) in mice via the ghrelin receptor. Many studies have reported benefits of dietary restriction (DR) on the brain that include increasing neurogenesis and enhancing cognitive function. However, neither the mechanisms underlying the effects of the 5:2 diet, nor potential benefits on the brain, are well understood. The authors hypothesize that the 5:2 diet enhances AHN and cognitive function via ghrelin-receptor signaling. To test this, they placed adolescent and adult ghrelin receptor knockout or wild type mice on either the 5:2 or ad libitum (AL) diet for 6 weeks, followed by spatial memory testing using an object in place (OIP) task.

The authors also assessed changes in AHN via IHC using multiple markers for cell proliferation and neural stem cells. The authors observed a decrease in AHN due to age (from adolescent to adult), but not due to diet or ghrelin-receptor signaling. While loss of the ghrelin-receptor impaired spatial memory, the 5:2 diet did not affect cognitive function. The authors conclude that the 5:2 diet does not enhance AHN or spatial memory.

We thank the reviewer for this summary. We note that there was a significant reduction in new neurones ($BrdU^{+}/NeuN^{+}$) cells in GHS-R null animals, regardless of age or diet (3 way ANOVA of age, genotype and diet (sexes pooled): Genotype P = 0.0290). These data suggest that the loss of ghrelin receptor signalling does impair AHN. However, we will re-analyse our data in light of reviewer 1 comments to remove ‘age’ as a variable. The new analyses and associated discussion will be presented in our revised manuscript.

1. The authors use a 5:2 diet but fail to provide a basic characterization of this dietary intervention. For example, was the food intake assessed? In addition to the time restriction of the feeding, does this intervention also represent an overall caloric restriction or not? According to the provided results, the 5:2 diet does not appear to regulate adult hippocampal neurogenesis contrary to the authors' original hypothesis. Did the authors measure the effects of the 5:2 diet on any other organ system? Do they have any evidence that the intervention itself resulted in any well documented benefits in other cell types? Such data would provide a critical positive control for their intervention.

This is an important point raised by the reviewer. Currently, we carefully quantified weight change across the duration of the study. However, we do not know whether the 5:2 diet reduced overall food intake or whether it impacted the timing of feeding events. To overcome this limitation, we will now test what impact the 5:2 dietary regime has on food intake and the timing of feeding. This study will allow us to correlate any changes with 5:2 diet. In addition, we have collected tibiae to quantify skeletal growth and have collected both liver and plasma (end point) samples which will be used to assess changes in the GH-IGF-1 axis. These additional studies will allow us to characterise the effects of the 5:2 paradigm on key indicators of physiological growth. These new data will be incorporated into the revised manuscript.

2. Based on the effects of ghrelin in other dietary interventions, the authors speculate that the effect of the 5:2 diet is similarly mediated through ghrelin. However, the authors do not provide any basic characterization of ghrelin signaling to warrant this strong focus on the GSH-R mice. While the GSH-R mice display changes in NSC homeostasis and neurogenesis, none of these effects appear to be modified by the 5:2 diet. Thus, the inclusion of the GSH-R mice does not seem warranted and detracts from the main 5:2 diet focus of the manuscript.

The role of ghrelin signalling via its receptor, GHSR, is a central tenet of our hypothesis. The loxTB-GHS-R null mouse is a well validated model of impaired ghrelin signalling, in which insertion of a transcriptional blocking cassette prevents expression of the ghrelin receptor (ZIgman et al.2005 JCI). We have previously shown that this mouse model is insensitive to calorie restriction (CR) mediated stimulation of AHN, in contrast to WT mice (Hornsby et al. 2016), justifying its suitability as a model for assessing the role of ghrelin signalling in response to DR interventions, such as the 5:2 paradigm. Whilst our findings do not support a role for ghrelin signalling in the context of the 5:2 diet studied, we did follow the scientific method to empirically test the stated hypothesis. While critiques of experimental design are welcome, the

removal of these data may perpetuate publication bias in favour of positive outcomes and is something we wish to avoid.

Neurogenesis is highly sensitive to stress. The 5:2 diet may be associated with stress which could counteract any benefits on neurogenesis in this experimental paradigm. Did the authors assess any measures of stress in their cohorts? Were the mice group housed or single housed?

We thank the reviewer for raising this point. We have open-field recordings that will now be analysed to assess general locomotor activity, anxiety and exploration behaviour. Additionally, we will assess levels of the stress hormone, ACTH, in end point plasma samples. These datasets will be incorporated into the revised manuscript.

4. The authors state that the 5:2 diet led to a greater reduction in body weight (31%) in adolescent males compared to other groups. However, it appears that the cohorts were not evenly balanced and the adolescent 5:2 male mice started out with a significantly higher starting weight (Supplementary Figure 1). The difference in starting weight at such a young age is significantly confounding the conclusion that the 5:2 diet is more effective at limiting weight gain specifically in this group.

We thank the reviewer for highlighting this limitation. In the revision we will re-focus our discussion around the Δ Body weight repeated measures data, which compares the daily body weight of each group to its baseline value - thereby normalising any intergroup differences in starting weight. Furthermore, we will restructure figures 1 and S1 so that figure 1 presents only the repeated measure Δ Body weight data, while data for body weight both at baseline and on the final day of the study will be presented in figure S1.

5. The authors count NSCs as Sox2+S100b- cells. However, the representative S100b staining does not look very convincing. Instead, it would be more appropriate to count Sox2+GFAP+ cells with a single vertical GFAP+ projection. Alternatively, the authors could also count Nestin-positive cells. Additionally, the authors label BrdU+ Sox2+ S100B- cells as "new NSCs". However, it appears that the BrdU labeling was performed approximately 6 weeks before the tissue was collected (Figure 1A). Thus, these BrdU-positive NSCs most likely represent label retaining/quiescent NSCs that divided during the labeling 6 weeks prior but have not proliferated since. As such, the term "new NSC" is misleading and would suggest an NSC that was actively dividing at the time of tissue collection.

We apologise for presenting low-resolution images – these will be replaced by high-resolution images in the revised manuscript. In this study we have quantified the actively dividing $BrdU^+$/$Sox2^+$/$S100B^-$ cells that represent type II NSCs (rather than $GFAP^+$ or $Nestin^+$ type I NSCs) that have incorporated BrdU within the time period of the 6-week intervention. We appreciate the reviewer's comments concerning the "new NSCs" terminology. We agree that we should be more specific in clarifying that the NSCs identified are those labelled during the 1st week of the 6-week intervention. We will amend this throughout the revised manuscript by re-naming these cells as 6-week old NSCs.

6. Overall, this manuscript lacks a clear focus and narrative. Due to a lack of an affect by the 5:2 diet on hippocampal neurogenesis, the authors mostly highlight already well-known effects of aging and Grehlin/GSH-R on neurogenesis. Moreover, the authors repeatedly use age-related

decline and morbidities as a rational for their study. However, they assess the effects of the 5:2 diet on neurogenesis only in adolescent and young mature but not aged mice.

To provide greater clarity, and in accordance with reviewer 1's comments, we will amend the text throughout to provide a focus on the data obtained. The objective of the changes will be to re-enforce the original study narrative. In relation to the use of the term 'age-related decline' or 'age-related changes', we think that these are appropriate to our study. Physiological ageing doesn't begin at a specific point of chronological time, but is a process that is continuously ongoing. Indeed, our data is in agreement with previous studies reporting an age-related reduction in AHN at 6 months of age (e.g Kuhn et al.196).

Minor Points

1. The authors combine the data from both male and female mice for most bar graphs. While this does not appear to matter for neurogenesis or behavioral readouts, there are very significant sexually dimorphic differences with respect to body size and weight. As such, male and female mice in Figure 1D,F should not be plotted in the same bar graph.

We agree that sexual dimorphism exists with respect to body size and weight. We used distinct male and female symbols for each individual animal on these bar graphs, but do agree with the reviewer that sexual dimorphic differences should be emphasized. To achieve this, we will include additional supplementary graphs presenting the sex differences in starting weight, final weight, and weight change versus starting weight.

2. The Figure legends are very brief and should be expanded to include basic information of the experimental design, statistical analyses etc.

We thank the reviewer for this comment. We will provide specific experimental detaisl in the revised figure legends.

3. Many figures include a representative image. However, it is often unclear if that is a representative image of a WT or mutant mouse, or a 5:2 or control group (Figure 2A, 3A, 4A, 5A).

We structured the manuscript with a limited number of figures and associated microscope captured panels, with the aim of presenting representative images to illustrate the nature and quality of the IHC protocols. However, we will amend the figures for the revised manuscript to provide representative microscopy images, with each group included and clearly annotated.

It would be helpful to provide representative images of DCX-positive cells in Figure 3A-F. Additionally, the authors should include a more extensive description of how this quantification was performed in the method section.

We will revise the manuscript to provide representative high-resolution $Dcx^+$ images displaying cells of each category. The method will also be revised to include a detailed description of how the quantification and classification was performed.

5. The authors state "the hippocampal rostro-caudal axis (also known as the dorsoventral[] axis". However, the rostral-caudal and dorsal-central axis are usually considered perpendicular to one another.

We agree that the dorso-ventral and rostral-caudal axes are anatomically distinct. The terms are often used interchangeably in the literature, which can lead misinterpretations (e.g the caudal portion of dorsal hippocampus is often mislabelled as ventral hippocampus). To avoid ambiguity, mislabelling or misidentification, we will include a supplementary figure detailing our anatomical definitions of the rostral and caudal poles of the hippocampus, alongside representative images and the bregma coordinates.

**Reviewer #2 (Significance (Required)):**

Understanding the mechanisms of a popular form of intermittent fasting (5:2 diet) that is not well understood is an interesting topic. Moreover, examining the effect of this form of intermittent fasting on the brain is timely. Notwithstanding, while the authors use multiple markers to validate the effect of the 5:2 diet on adult hippocampal neurogenesis, concerns regarding experimental design, validation, and data analysis weaken the conclusions being drawn.

We thank reviewer 2 for this significance statement. We will revise the manuscript, as mentioned above, to clarify the experimental design, improve presentation of the data, and re-focus the narrative of the primary aims of the study.

**Reviewer #3 (Evidence, reproducibility and clarity (Required)):**

**Summary**

In this study, Roberts and colleagues used a specific paradigm of intermitted fasting, the 5:2 diet, meaning 5 days ad libitum food and 2 non-consecutive days of fasting. They exposed adolescent and adult wild-type mice and ghrelin receptor knockout mice (GHS-R-/-) for 6 weeks to this paradigm, followed by 1 week ad libitum food. They further used the "object in place task" (OIP) to assess spatial memory performance. At the end of the dietary regime, the authors quantified newborn neurons and neural stem cells (NSCs) by immunohistochemistry. Roberts et al. show that the 5:2 diet does not change the proliferation of cells in the hippocampus, but report an increased number of immature neurons (based on DCX) in all the mice exposed to the 5:2 diet. This change however did not result in an increased number of mature adult-born neurons, as assessed by a BrdU birthdating paradigm. The authors further show diet-independent effects of the ghrelin receptor knockout, leading to less adult born neurons, but more NSCs in the adolescent mice and a lower performance in the OIP task.

**Major comments:**

The main conclusion of this study is that a specific type of intermitted fasting (5:2 diet) has no effects on NSC proliferation and neurogenesis. As there are several studies showing beneficial effects of intermitted fasting on adult neurogenesis, while other studies found no effects, it is important to better understand the effects of such a dietary paradigm.

# Revision Plan

The experimental approaches used in this manuscript are mostly well explained, but it is overall rather difficult to follow the results part, as the authors always show the 4 experimental groups together (adolescent vs adult and wt vs GHS-R-/-). They highlight the main effects comparing all the groups, which most of the time is the factor "age". Age is a well-known and thus not surprising negative influencer of adult neurogenesis. Instead of focusing on the main tested factor, namely the difference in diet, the authors show example images of the two age classes (adolescent vs adult), which does not underly the major point they are making. Most of the time, they do not provide a post hoc analysis, so it is difficult to judge if the results with a significant main effect would be significant in a direct 1 to 1 comparison of the corresponding groups. The authors point out themselves that previous rodent studies did not use such a 5:2 feeding pattern, so having diet, age and genotype as factors at the same time makes the assessment of the diet effect more difficult.

The manuscript would improve if the authors restructure their data to compare first the diet groups (adolescent wt AL vs 5:2 and in a separate comparison adult wt AL vs 5:2) and only in a later part of the results check if the Ghrelin receptor plays a role or not in this paradigm.

We thank the reviewer for these comments. In line with comments from the other reviewers we will re-formulate the presentation of our datasets. We will remove 'age' as a key variable as age related changes are to be expected. For the revision, we will separate the adolescent and adult mouse data sets, plotting individual graphs for both. This should provide a clearer focus on 5:2 responses in both assessed genotypes.

This re-configuration will impact the data being analysed and, therefore, the statistical analysis presented. In our original manuscript *post hoc* analyses were performed, however, only significant *post hoc* comparisons were highlighted (e.g figure 5). Non-significant post hoc comparisons have not been presented. In the method section of the revised manuscript, we will clarify that we'll report *post hoc* differences when they are observed.

During our study design, we decided to assess diet and genotype in parallel - as part of the same analysis. This seemed to us to be the most appropriate statistical method, so that we assessed dietary responses in both WT and GHS-R null mice.

As this 5:2 is a very specific paradigm, it is furthermore difficult to compare these results to other studies and the conclusions are only valid for this specific pattern and timing of the intervention (6 weeks). It remains unclear why the authors have not first tried to establish a study with wildtype mice and a similar duration as in previous studies observing beneficial effects of intermitted fasting on neurogenesis. Like this, it would have been possible to make a statement if the 5:2 per se does not increase neurogenesis or if the 6 weeks exposure were just too short.

The reviewer raises this relevant point which we considered during the study design period. Given that we had previously reported significant modulation of AHN with a relatively short period of 30% CR (14 days followed by 14 days AL refeeding (Hornsby *et al*.2016)), we predicted that a 6 week course on the 5:2 paradigm (totalling 12 days of complete food restriction over the 6 week period) would provide a similar dietary challenge. The fact that we did not observe similar changes in AHN with this 5:2 paradigm is notable.

# Revision Plan

The graphical representation of the data could also be improved. Below are a few examples listed:

1.) Figure 1 B and C, the same symbol and colours are used for the adolescent and adult animals, which makes the graphs hard to read. One colour and symbol per group throughout the manuscript would be better.

We thank the reviewer for this comment. We will amend the presentation of the graphs throughout the manuscript to ensure that they are easier to interpret.

2.) The authors found no differences in the total number of Ki67 positive cells in the DG. However, Ki67 staining does not allow to conclude the type of cell which is proliferating. It would thus strengthen the findings if this analysis was combined with different markers, such as Sox2, GFAP and DCX.

Double labelling of Ki67 positive cells would allow for further insight into the identity of distinct proliferating cell populations. However, quantifying Ki67 immunopositive cells within the sub-granular zone of the GCL, as a single marker, is commonly used in studies of AHN. Given that studies of intermittent fasting, calorie restriction and treatment with exogenous acyl-ghrelin report no effect on NPC cell division, we decided not to pursue this line of inquiry.

3.) In Figure 3, the authors say that the diet increases the number of DCX in adolescent and adult mice, which is not clear when looking at the graph in 3B. Are there any significant differences when directly comparing the corresponding groups, for instance the WT AL vs the WT 5:2? It is further not clear how the authors distinguished the different types of DCX morphology-wise. The quantification in C and D would need to be illustrated by example images. Furthermore, the colour-code used in these graphs is not explained and remains Unclear

While the 3 way ANOVA does yield a significant overall effect for diet, we agree that it is indeed difficult to see a difference on the graph, although the mean values of the adolescent 5:2 animals are more prominent than the AL counterparts. Mean +/- SEM will be provided in the supplementary section of the revised manuscript. Furthermore, we will clarify the method used to identify distinct $DCX^+$ morphologies, include representative high-resolution images of each $DCX^+$ cell category, and amend the colour coding to avoid misinterpretation.

4) In Figure 5, the authors show that the number of new NSCs is significantly increased in the adolescent GHS-R-/- mice, independent of the diet, but this increase does not persist in the adult mice. They conclude that "the removal of GHS-R has a detrimental effect on the regulation of new NSC number..." this claim is not substantiated and needs to be reformulated. As the GHS-R-/- mice have a transcriptional blockage of Ghrs since start of its expression, would such an effect on NSC regulation not result in an overall difference in brain development, as ghrelin is also important during embryonic development?

This is an interesting point. However, we disagree that the statement "*the removal of GHS-R has a detrimental effect on the regulation of new NSC number...*" is unsubstantiated, since it does not exclude any developmental deficits in these mice that may account for the differences observed. Nonetheless, we will rephrase the sentence to clarify our intended point and remove any ambiguity.

5.) In Figure 6, the authors asses spatial memory performance with a single behavioral test, the OIP. As these kind of tests are influenced by the animal's motivation to explore, it's anxiety levels, physical parameters (movement) etc., the interpretation of such a test without any additional measured parameters can be problematic. The authors claim that the loss of GHS-R expression impairs spatial memory performance. As the discrimination ratio was calculated, it is not possible to see if there is an overall difference in exploration time between genotypes. This would be a good additional information to display.

We thank the reviewer for this insight. We have open-field recordings that will now be analysed to assess general locomotor activity, anxiety and exploration behaviour. These data, alongside exploratory time of the mice during the OIP task will be incorporated into the revised manuscript.

Besides these points listed above, the methods are presented in such a way that they can be reproduced. The experiments contained 10-15 mice per group, which is a large enough group to perform statistical analyses. As mentioned above, the statistical analysis over all 4 groups with p-values for the main effects should be followed by post hoc multiple comparison tests to allow the direct comparison of the corresponding groups.

**Reviewer #3 (Significance (Required)):**

In the last years, growing evidences suggested that IF might have positive effect on health in general and also for neurogenesis. However, a few recent studies report no effects on neurogenesis, using different IF paradigms. This study adds another proof that not all IF paradigms influence neurogenesis and shows that more work needs to be done to better understand when and how IF can have beneficial effects. This is an important finding for the neurogenesis field, but the results are only valid for this specific paradigm used here, which limits its significance. The reporting of such negative findings is however still important, as it shows that IF is not just a universal way to increase neurogenesis. In the end, such findings might have the potential to bring the field together to come up with a more standardized dietary intervention paradigm, which would be robust enough to give similar results across laboratories and mouse strains, and would allow to test the effect of genetic mutations on dietary influences of neurogenesis.

We thank the reviewer for their insightful and thorough feedback.

## 3. Description of the revisions that have already been incorporated in the transferred manuscript

*Please insert a point-by-point reply describing the revisions that were already carried out and included in the transferred manuscript. If no revisions have been carried out yet, please leave this section empty.*

The manuscript has not been revised at this stage.

4. Description of analyses that authors prefer not to carry out

*Please include a point-by-point response explaining why some of the requested data or additional analyses might not be necessary or cannot be provided within the scope of a revision. This can be due to time or resource limitations or in case of disagreement about the necessity of such additional data given the scope of the study. Please leave empty if not applicable.*

We have included in our replies to the reviewers a description of the amendments that we will make to our manuscript. Two requested revisions stand out as being unnecessary or cannot be provided within the scope of a revision.

The first was the request to perform the 5:2 study in older mice. This an interesting suggestion, however, the expense and time needed to maintain mice into old age (e.g >18 months) cannot be provided within the scope of our revision. In addition, given that we report no effect of the 5:2 paradigm on AHN in adolescent (7 week old) and adult (7 month old) mice, there is less justification for such a study in old mice.

The second request, that we disagree with, was to remove data relating to the GHS-R null mice (see reviewer 2, point 2). The role of ghrelin signalling via its receptor, GHS-R, is a central tenet of our hypothesis. Whilst our findings do not support a role for ghrelin signalling in the context of the 5:2 diet studied, we followed the scientific method to empirically test the stated hypothesis. While critiques of experimental design are welcome, the removal of such data may perpetuate publication bias in favour of positive outcomes and is something we wish to avoid.

**1st Editorial Decision** **11th Apr 2023**

Dear Prof. Davies,

Thank you for transferring your research manuscript from Review Commons to EMBO reports. I apologize for the delayed response, but I have now read and discussed your manuscript with the other members of our editorial board, along with the reports of the referees who evaluated it via Review Commons, as well as your revision plan.

The referees are positive about the significance of the work and acknowledge that the findings are potentially interesting. However, they also identify a number of limitations in your study and provide suggestions for its improvement. Given their constructive comments, we would like to invite you to revise your manuscript with the understanding that the referee concerns (as detailed in their reports) must be fully addressed and their suggestions taken on board. Please address all referee concerns in a complete point-by-point response. Acceptance of the manuscript will depend on a positive outcome of a second round of review. It is EMBO reports policy to allow a single round of revision only and acceptance or rejection of the manuscript will therefore depend on the completeness of your responses included in the next, final version of the manuscript. If you have any questions or comments, we can also discuss the revisions in a video chat, if you like.

We realize that it is difficult to revise to a specific deadline. In the interest of protecting the conceptual advance provided by the work, we usually recommend a revision within 3 months (July 10th). Please discuss with me the revision progress ahead of this time if you require more time to complete the revisions.

*****
IMPORTANT NOTE:
We perform an initial quality control of all revised manuscripts before re-review. Your manuscript will FAIL this control and the handling will be DELAYED if the following APPLIES:

1) If a data availability section providing access to data deposited in public databases is missing. If you have not deposited any data, please add a sentence to the data availability section that explains that (see below for more information).

2) If your manuscript contains statistics and error bars based on n=2. Please use scatter plots in these cases. No statistics should be calculated if n=2.

When submitting your revised manuscript, please carefully review the instructions that follow below. Failure to include requested items will delay the evaluation of your revision.
*****

When submitting your revised manuscript, we will require:

1) A .docx formatted version of the manuscript text (including legends for main figures, EV figures and tables). Please make sure that the changes are highlighted to be clearly visible.

2) Individual production quality figure files as .eps, .tif, .jpg (one file per figure).
Please download our Figure Preparation Guidelines (figure preparation pdf) from our Author Guidelines pages https://www.embopress.org/page/journal/14693178/authorguide for more info on how to prepare your figures.

3) A .docx formatted letter INCLUDING the reviewers' reports and your detailed point-by-point responses to their comments. As part of the EMBO Press transparent editorial process, the point-by-point response is part of the Review Process File (RPF), which will be published alongside your paper unless you opt out of this (please see below for further information).

4) A complete author checklist, which you can download from our author guidelines (). Please insert information in the checklist that is also reflected in the manuscript. The completed author checklist will also be part of the RPF (please see below for more information).

5) Please note that all corresponding authors are required to supply an ORCID ID for their name upon submission of a revised manuscript (). Please find instructions on how to link your ORCID ID to your account in our manuscript tracking system in our Author guidelines
()

6) We replaced Supplementary Information with Expanded View (EV) Figures and Tables that are collapsible/expandable online. A maximum of 5 EV Figures can be typeset. EV Figures should be cited as 'Figure EV1, Figure EV2" etc... in the text and their respective legends should be included in the main text after the legends of regular figures.

- For the figures that you do NOT wish to display as Expanded View figures, they should be bundled together with their legends in a single PDF file called *Appendix*, which should start with a short Table of Content. Appendix figures should be referred to in the main text as: "Appendix Figure S1, Appendix Figure S2" etc. See detailed instructions regarding expanded view here:

- Additional Tables/Datasets should be labeled and referred to as Table EV1, Dataset EV1, etc. Legends have to be provided in a separate tab in case of .xls files. Alternatively, the legend can be supplied as a separate text file (README) and zipped together with the Table/Dataset file.

7) Please note that a "Data availability" section at the end of Materials and Methods is now mandatory. In case you have no data that require deposition in a public database, please state so instead of refereeing to the database: "Our study includes no data deposited in public repositories." under the heading "Data availability".
See also ). Please note that the Data availability statement is restricted to new primary data that are part of this study.

8) We request authors to consider both actual and perceived competing interests. Please review the new policy () and update your competing interests statement if necessary. Please name this section 'Disclosure and competing interests statement' and place it after the Acknowledgements section.

9) Figure legends and data quantification:
The following points must be specified in each figure legend:

- the name of the statistical test used to generate error bars and P values,
- the number (n) of independent experiments (please specify technical or biological replicates) underlying each data point,
- the nature of the bars and error bars (s.d., s.e.m.)
- If the data are obtained from n {less than or equal to} 2, use scatter plots showing the individual data points.

Discussion of statistical methodology can be reported in the Materials and Methods section, but figure legends should contain a basic description of n, P and the test applied.

See also the guidelines for figure legend preparation:
https://www.embopress.org/page/journal/14693178/authorguide#figureformat

- Please also include scale bars in all microscopy images.

10) We now request publication of original source data with the aim of making primary data more accessible and transparent to the reader. Our source data coordinator will contact you to discuss which figure panels we would need source data for and will also provide you with helpful tips on how to upload and organize the files.

11) Our journal encourages inclusion of *data citations in the reference list* to directly cite datasets that were re-used and obtained from public databases. Data citations in the article text are distinct from normal bibliographical citations and should directly link to the database records from which the data can be accessed. In the main text, data citations are formatted as follows: "Data ref: Smith et al, 2001" or "Data ref: NCBI Sequence Read Archive PRJNA342805, 2017". In the Reference list, data citations must be labeled with "[DATASET]". A data reference must provide the database name, accession number/identifiers and a resolvable link to the landing page from which the data can be accessed at the end of the reference. Further instructions are available at .

12) Please also note our reference format:
.

13) We now use CRediT to specify the contributions of each author in the journal submission system. CRediT replaces the author contribution section, which should be removed from the manuscript. Please use the free text box to provide more detailed descriptions. See also guide to authors:
.

14) As part of the EMBO publications' Transparent Editorial Process, EMBO reports publishes online a Review Process File to accompany accepted manuscripts. This File will be published in conjunction with your paper and will include the referee reports, your point-by-point response and all pertinent correspondence relating to the manuscript.

You can opt out of this by letting the editorial office know (emboreports@embo.org). If you do opt out, the Review Process File link will point to the following statement: "No Review Process File is available with this article, as the authors have chosen not to make the review process public in this case."

We would also welcome the submission of cover suggestions, or motifs to be used by our Graphics Illustrator in designing a

cover.

I look forward to seeing a revised version of your manuscript when it is ready. Please let me know if you have any questions or comments regarding the revision.

Yours sincerely,

Ioannis Papaioannou, PhD
Editor
EMBO reports

# Authors' Response

Review COMMONS

**Manuscript number:** RC-2022-01714
**Corresponding author(s):** Jeffrey, Davies

## 1. General Statements [optional]

*This section is optional. Insert here any general statements you wish to make about the goal of the study or about the reviews.*

## 2. Description of the planned revisions

We provide here a point-by-point response to the reviewer's comments, with our answers and comments in blue.
>> We provide a point-by-point response confirming the changes made to our revised manuscript in response to the questions raised by the reviewers (green).

**Reviewer #1** (Evidence, reproducibility and clarity (Required)):

**Summary:**
In this manuscript, Roberts et al. hypothesised that the 5:2 diet (a popular form of IF, a dietary strategy within the Intermittent fasting that is thought to increase adult hippocampal neurogenesis - AHN) would enhance AHN in a ghrelin-dependent manner. To do this, the Authors used immunohistochemistry to quantify new adult-born neurons and new neural stem cells in the hippocampal dentate gyrus of adolescent and adult wild-type mice and mice lacking the ghrelin receptor, following six weeks on a 5:2 diet. They report an age-related decline in neurogenic processes and identify a novel role for ghrelin-receptor in regulating the formation of new adult
born neural stem cells in an age-dependent manner. However, the 5:2 diet did not affect new neuron or neural stem cell formation in the dentate gyrus, nor did alter performance on a spatial learning and memory task. They conclude that the 5:2 diet used in their study does not increase AHN or improve associated spatial memory function.

**Major comments:**
One criticism might be the fact that many aspects are addressed at the same time. For instance it is not fully clear the role of ghrelin with respect to testing the DR effects on AHN. Although the link between ghrelin, CR and AHN is explained by citing several previous studies, it is difficult to identify the main focus of the study. Maybe this is due to the fact that the Authors analyse and comment throughout the paper the different experimental approaches used by different Authors to study effect of DR to AHN. This is not bad in principle, since I think the Authors have a deep knowledge of this complex matter, but all this results in a difficulty to follow the flow of the rationale in the manuscript.

We appreciate the reviewer's critique regarding the rationale of the studies presented in the manuscript.
The role of ghrelin in the regulation of AHN by dietary interventions such as CR and IF is a major interest of our lab and is the main focus of the study. We, and others, have shown that ghrelin mediates the beneficial effects of CR on AHN. It is often assumed that ghrelin will elicit similar effects in other DR paradigms. We selected the 5:2 diet since it is widely practiced by humans, but it has not been well tested experimentally.
We sought to empirically test how the neurogenic response to 5:2 differed between mice with functional and impaired ghrelin signaling.
Given that plasma ghrelin levels and AHN are reduced during ageing, we also wanted to determine if 5:2 diet could slow or even prevent neurogenic decline in ageing mice.
We will re-write the manuscript to ensure that our primary aim is clearly presented. We will also reanalyze the data, with genotype and 5:2 diet as key variables. To help maintain focus, the variable of age will be analyzed separately. This amendment will, we hope, help the reader follow the narrative of our manuscript.
>> In line with the comments above, we have made considerable changes to the manuscript. These include amending the text to emphasize the study's central aim of assessing the effect of 5:2 diet on neurogenesis and learning, re-analyzing the data without including age as a key variable. The narrative is improved by these changes.

Another major point: the Discussion is too long. The Authors analyse all the possible reasons why different studies obtained different results concerning the effectiveness of DR in stimulating adult neurogenesis. Thus, the Discussion seems more as a review article dealing with different methods/experimental approaches to evaluate DR effects. We know that sometimes different results are due to different experimental approaches, yet, when an effect is strong and clear, it occurs in different situations. Thus, I think that the Authors must be less shy in expressing their conclusions, also reducing the methodological considerations. It is also well known that sometimes different results can be due to a study not well performed, or to biases from the Authors.

In our discussion, we felt that it was particularly important to be as rigorous as possible in contextualizing our findings with other published data, whilst highlighting methodological differences. Our aim was to be as precise as possible when comparing findings across studies, however, this resulted in the narrative drifting from the key objectives of our study – namely, to determine the effect of 5:2 diet on neurogenesis and whether or not ghrelin-signalling regulated the process. We will amend the text of the discussion to ensure that the key points of our study are only compared and contrasted with relevant studies in the field. We thank the reviewer for their candid comment.
>> The discussion has been extensively edited, with the removal of unnecessary text that is not related to our study.

**Minor comments:**

- This sentence: "There is an age-related decline in adult hippocampal neurogenesis" cannot be put in the HIGHLIGHTS, since is a well known aspect of adult hippocampal neurogenesis

The reviewer is correct to state this. Our study replicates this interesting age-related phenomenon. However, we will remove it from the 'Highlights' section.
>> Completed.

- Images in Figure 5 are not good quality.

We apologise for this oversight. We will review each figure and panel to ensure that high-resolution images, that are appropriately annotated, are used throughout the manuscript.
>> Completed.

- In general, there are not a lot of images referring to microscopic/confocal photographs across the entire manuscript.

We structured the manuscript with a limited number of figures and associated microscope captured panels, with the aim of presenting representative images to illustrate the nature and quality of the IHC protocols. However, we will amend the figures for the revised manuscript to provide representative microscopy images, with each group included and clearly annotated.
>> Completed.

- The last sentence of the Discussion "These findings suggest that distinct DR regimens differentially regulate neurogenesis in the adult hippocampus and that further studies are required to identify optimal protocols to support cognition during ageing" is meaningless in the context of the study, and in contrast with the main results. Honestly, my impression is that the Authors do not want to disappoint the conclusions of the previous studies; an alternative is that other Reviewers asked for this previously.

We do not believe that this statement is contradictory to our findings, as distinct DR paradigms do appear to regulate AHN in different ways. However, we agree that we can be more explicit with regards to our own study findings and will prioritize the conclusions of our study over those of the entire field during revision.
>> Completed.

**Reviewer #1 (Significance (Required)):**

The significance of this study relies on the fact that adult neurogenesis field (AN) has been often damaged by the search of "positive" results, aiming at showing that AN does occur "always and everywhere" and that most internal/external stimuli do increase it. This attitude created a bias in the field, persuading many scientists that a result in AN is worthy of publication (or of high impact factor publication) only when a positive result is found.
The Author cite in the Discussion the work by Gabarro-Solanas et al. (preprint), which share the same conclusion although analysing different aspect of neurogenesis.
Both seems sound studies, substantially balanced in their conclusions.

We appreciate the reviewer's comment on publication bias and that they value the significance of publishing studies that will advance the field.

**Reviewer #2 (Evidence, reproducibility and clarity (Required)):**

In this manuscript, Roberts et al. investigate the effect of the 5:2 diet on adult hippocampal neurogenesis (AHN) in mice via the ghrelin receptor. Many studies have reported benefits of dietary restriction (DR) on the brain that include increasing neurogenesis and enhancing cognitive function. However, neither the mechanisms underlying the effects of the 5:2 diet, nor potential benefits on the brain, are well understood. The authors hypothesize that the 5:2 diet enhances AHN and cognitive function via ghrelin-receptor signaling. To test this, they placed adolescent and adult ghrelin receptor knockout or wild type mice on either the 5:2 or ad libitum (AL) diet for 6 weeks, followed by spatial memory testing using an object in place (OIP) task. The authors also assessed changes in AHN via IHC using multiple markers for cell proliferation and neural stem cells. The authors observed a decrease in AHN due to age (from adolescent to adult), but not due to diet or ghrelin-receptor signaling. While loss of the ghrelin-receptor impaired spatial memory, the 5:2 diet did not affect cognitive function. The authors conclude that the 5:2 diet does not enhance AHN or spatial memory.

We thank the reviewer for this summary. We note that there was a significant reduction in new neurones ($BrdU^+/NeuN^+$) cells in GHS-R null animals, regardless of age or diet (3 way ANOVA of age, genotype and diet (sexes pooled): Genotype P = 0.0290). These data suggest that the loss of ghrelin receptor signalling does impair AHN. However, we will re-analyse our data in light of reviewer 1 comments to remove 'age' as a variable. The new analyses and associated discussion will be presented in our revised manuscript.
>> Completed. The data has been re-analysed accordingly and presented in the manuscript.

1. The authors use a 5:2 diet but fail to provide a basic characterization of this dietary intervention. For example, was the food intake assessed? In addition to the time restriction of the feeding, does this intervention also represent an overall caloric restriction or not? According to the provided results, the 5:2 diet does not appear to regulate adult hippocampal neurogenesis contrary to the authors' original hypothesis. Did the authors measure the effects of the 5:2 diet on any other organ system? Do they have any evidence that the intervention itself resulted in any well documented benefits in other cell types? Such data would provide a critical positive control for their intervention.

This is an important point raised by the reviewer. Currently, we carefully quantified weight change across the duration of the study. However, we do not know whether the 5:2 diet reduced overall food intake or whether it impacted the timing of feeding events. To overcome this limitation, we will now test what impact the 5:2 dietary regime has on food intake and the timing of feeding. This study will allow us to correlate any changes with 5:2 diet. In addition, we have collected tibiae to quantify skeletal growth and have collected both liver and plasma (end point) samples which will be used to assess changes in the GH-IGF-1 axis. These additional studies will allow us to characterise the effects of the 5:2 paradigm on key indicators of physiological growth. These new data will be incorporated into the revised manuscript.
>> We have performed an additional study (presented as EV1) demonstrating the effect of 5:2 diet on food intake and body weight change. We have also quantified nose-anus length and tibiae length (presented in Table S1) to provide further insights into the physiological effects of our 5:2 dietary regime. We have not performed the liver and plasma IGF-1 analyses, however,

these analyses will be performed and presented in a future manuscript focussing on the metabolic consequences of intermittent fasting related diets.

2. Based on the effects of ghrelin in other dietary interventions, the authors speculate that the effect of the 5:2 diet is similarly mediated through ghrelin. However, the authors do not provide any basic characterization of ghrelin signaling to warrant this strong focus on the GSH-R mice. While the GSH-R mice display changes in NSC homeostasis and neurogenesis, none of these effects appear to be modified by the 5:2 diet. Thus, the inclusion of the GSH-R mice does not seem warranted and detracts from the main 5:2 diet focus of the manuscript.

The role of ghrelin signalling via its receptor, GHSR, is a central tenet of our hypothesis. The loxTB-GHS-R null mouse is a well validated model of impaired ghrelin signalling, in which insertion of a transcriptional blocking cassette prevents expression of the ghrelin receptor (ZIgman et al.2005 JCI). We have previously shown that this mouse model is insensitive to calorie restriction (CR) mediated stimulation of AHN, in contrast to WT mice (Hornsby et al. 2016), justifying its suitability as a model for assessing the role of ghrelin signalling in response to DR interventions, such as the 5:2 paradigm. Whilst our findings do not support a role for ghrelin signalling in the context of the 5:2 diet studied, we did follow the scientific method to empirically test the stated hypothesis. While critiques of experimental design are welcome, the removal of these data may perpetuate publication bias in favour of positive outcomes and is something we wish to avoid.
>> Completed.

Neurogenesis is highly sensitive to stress. The 5:2 diet may be associated with stress which could counteract any benefits on neurogenesis in this experimental paradigm. Did the authors assess any measures of stress in their cohorts? Were the mice group housed or single housed?

We thank the reviewer for raising this point. We have open-field recordings that will now be analysed to assess general locomotor activity, anxiety and exploration behaviour. Additionally, we will assess levels of the stress hormone, ACTH, in end point plasma samples. These datasets will be incorporated into the revised manuscript.
>> Completed. Our revised manuscript demonstrates that the 5:2 diet does not alter anxiety like behaviour in the Open Field test, and that circulating corticosterone is largely unaltered. These data suggest that any stress imparted by the 5:2 diet is negligible and unlikely to counteract any pro-neurogenic effects of 5:2 diet.

4. The authors state that the 5:2 diet led to a greater reduction in body weight (31%) in adolescent males compared to other groups. However, it appears that the cohorts were not evenly balanced and the adolescent 5:2 male mice started out with a significantly higher starting weight (Supplementary Figure 1). The difference in starting weight at such a young age is significantly confounding the conclusion that the 5:2 diet is more effective at limiting weight gain specifically in this group.

We thank the reviewer for highlighting this limitation. In the revision we will re-focus our discussion around the Δ Body weight repeated measures data, which compares the daily body weight of each group to its baseline value - thereby normalising any intergroup differences in starting weight. Furthermore, we will restructure figures 1 and S1 so that figure 1 presents only

the repeated measure Δ Body weight data, while data for body weight both at baseline and on the final day of the study will be presented in figure S1.
>> Completed. Δ Body weight data across the duration of the study are presented in figure 1 for each sex, genotype and age.

5. The authors count NSCs as Sox2+S100b- cells. However, the representative S100b staining does not look very convincing. Instead, it would be more appropriate to count Sox2+GFAP+ cells with a single vertical GFAP+ projection. Alternatively, the authors could also count Nestin-positive cells. Additionally, the authors label BrdU+ Sox2+ S100B- cells as "new NSCs". However, it appears that the BrdU labeling was performed approximately 6 weeks before the tissue was collected (Figure 1A). Thus, these BrdU-positive NSCs most likely represent label retaining/quiescent NSCs that divided during the labeling 6 weeks prior but have not proliferated since. As such, the term "new NSC" is misleading and would suggest an NSC that was actively dividing at the time of tissue collection.

We apologise for presenting low-resolution images – these will be replaced by high-resolution images in the revised manuscript. In this study we have quantified the actively dividing $BrdU^+/Sox2^+/S100B^-$ cells that represent type II NSCs (rather than $GFAP^+$ or $Nestin^+$ type I NSCs) that have incorporated BrdU within the time period of the 6-week intervention. We appreciate the reviewer's comments concerning the "new NSCs" terminology. We agree that we should be more specific in clarifying that the NSCs identified are those labelled during the 1$^{st}$ week of the 6-week intervention. We will amend this throughout the revised manuscript by re-naming these cells as 6-week old NSCs.
>> Completed.

6. Overall, this manuscript lacks a clear focus and narrative. Due to a lack of an affect by the 5:2 diet on hippocampal neurogenesis, the authors mostly highlight already well-known effects of aging and Grehlin/GSH-R on neurogenesis. <u>Moreover, the authors repeatedly use age-related decline and morbidities as a rational for their study. However, they assess the effects of the 5:2 diet on neurogenesis only in adolescent and young mature but not aged mice.</u>

To provide greater clarity, and in accordance with reviewer 1's comments, we will amend the text throughout to provide a focus on the data obtained. The objective of the changes will be to re-enforce the original study narrative. In relation to the use of the term 'age-related decline' or 'age-related changes', we think that these are appropriate to our study. Physiological ageing doesn't begin at a specific point of chronological time, but is a process that is continuously ongoing. Indeed, our data is in agreement with previous studies reporting an age-related reduction in AHN at 6 months of age (e.g Kuhn et al.1996).
>> Completed.

Minor Points

1. The authors combine the data from both male and female mice for most bar graphs. While this does not appear to matter for neurogenesis or behavioral readouts, there are very significant sexually dimorphic differences with respect to body size and weight. As such, male and female mice in Figure 1D,F should not be plotted in the same bar graph.

We agree that sexual dimorphism exists with respect to body size and weight. We used distinct male and female symbols for each individual animal on these bar graphs, but do agree with the reviewer that sexual dimorphic differences should be emphasized. To achieve this, we will

include additional supplementary graphs presenting the sex differences in starting weight, final weight, and weight change versus starting weight.
>> Completed. We now clearly present the data for sex, genotype and age.

2. The Figure legends are very brief and should be expanded to include basic information of the experimental design, statistical analyses etc.

We thank the reviewer for this comment. We will provide specific experimental detaisl in the revised figure legends.
>> Completed.

3. Many figures include a representative image. However, it is often unclear if that is a representative image of a WT or mutant mouse, or a 5:2 or control group (Figure 2A, 3A, 4A, 5A).

We structured the manuscript with a limited number of figures and associated microscope captured panels, with the aim of presenting representative images to illustrate the nature and quality of the IHC protocols. However, we will amend the figures for the revised manuscript to provide representative microscopy images, with each group included and clearly annotated.
>> Completed.

It would be helpful to provide representative images of DCX-positive cells in Figure 3A-F. Additionally, the authors should include a more extensive description of how this quantification was performed in the method section.

We will revise the manuscript to provide representative high-resolution $Dcx^+$ images displaying cells of each category. The method will also be revised to include a detailed description of how the quantification and classification was performed.
>> Completed.

5. The authors state "the hippocampal rostro-caudal axis (also known as the dorsoventral[] axis". However, the rostral-caudal and dorsal-central axis are usually considered perpendicular to one another.

We agree that the dorso-ventral and rostral-caudal axes are anatomically distinct. The terms are often used interchangeably in the literature, which can lead misinterpretations (e.g the caudal portion of dorsal hippocampus is often mislabelled as ventral hippocampus). To avoid ambiguity, mislabelling or misidentification, we will include a supplementary figure detailing our anatomical definitions of the rostral and caudal poles of the hippocampus, alongside representative images and the bregma coordinates.
>> Completed. Revised figure 4 and figure EV2 (related to figure 4) include details of the dorso-ventral and rostral-caudal axes analysed in the study.

**Reviewer #2 (Significance (Required)):**

Understanding the mechanisms of a popular form of intermittent fasting (5:2 diet) that is not well understood is an interesting topic. Moreover, examining the effect of this form of intermittent fasting on the brain is timely. Notwithstanding, while the authors use multiple markers to validate the effect of the 5:2 diet on adult hippocampal neurogenesis, concerns regarding experimental design, validation, and data analysis weaken the conclusions being drawn.

We thank reviewer 2 for this significance statement. We will revise the manuscript, as mentioned above, to clarify the experimental design, improve presentation of the data, and re-focus the narrative of the primary aims of the study.

**Reviewer #3 (Evidence, reproducibility and clarity (Required)):**

**Summary**

In this study, Roberts and colleagues used a specific paradigm of intermitted fasting, the 5:2 diet, meaning 5 days ad libitum food and 2 non-consecutive days of fasting. They exposed adolescent and adult wild-type mice and ghrelin receptor knockout mice (GHS-R-/-) for 6 weeks to this paradigm, followed by 1 week ad libitum food. They further used the "object in place task" (OIP) to assess spatial memory performance. At the end of the dietary regime, the authors quantified newborn neurons and neural stem cells (NSCs) by immunohistochemistry. Roberts et al. show that the 5:2 diet does not change the proliferation of cells in the hippocampus, but report an increased number of immature neurons (based on DCX) in all the mice exposed to the 5:2 diet. This change however did not result in an increased number of mature adult-born neurons, as assessed by a BrdU birthdating paradigm. The authors further show diet-independent effects of the ghrelin receptor knockout, leading to less adult born neurons, but more NSCs in the adolescent mice and a lower performance in the OIP task.

**Major comments:**

The main conclusion of this study is that a specific type of intermitted fasting (5:2 diet) has no effects on NSC proliferation and neurogenesis. As there are several studies showing beneficial effects of intermitted fasting on adult neurogenesis, while other studies found no effects, it is important to better understand the effects of such a dietary paradigm.

The experimental approaches used in this manuscript are mostly well explained, but it is overall rather difficult to follow the results part, as the authors always show the 4 experimental groups together (adolescent vs adult and wt vs GHS-R-/-). They highlight the main effects comparing all the groups, which most of the time is the factor "age". Age is a well-known and thus not surprising negative influencer of adult neurogenesis. Instead of focusing on the main tested factor, namely the difference in diet, the authors show example images of the two age classes (adolescent vs adult), which does not underly the major point they are making. Most of the time, they do not provide a post hoc analysis, so it is difficult to judge if the results with a significant main effect would be significant in a direct 1 to 1 comparison of the corresponding groups. The authors point out themselves that previous rodent studies did not use such a 5:2 feeding pattern, so having diet, age and genotype as factors at the same time makes the assessment of the diet effect more difficult.

The manuscript would improve if the authors restructure their data to compare first the diet groups (adolescent wt AL vs 5:2 and in a separate comparison adult wt AL vs 5:2) and only in a later part of the results check if the Ghrelin receptor plays a role or not in this paradigm.

We thank the reviewer for these comments. In line with comments from the other reviewers we will re-formulate the presentation of our datasets. We will remove 'age' as a key variable as age related changes are to be expected. For the revision, we will separate the adolescent and adult

mouse data sets, plotting individual graphs for both. This should provide a clearer focus on 5:2 responses in both assessed genotypes.

This re-configuration will impact the data being analysed and, therefore, the statistical analysis presented. In our original manuscript *post hoc* analyses were performed, however, only significant *post hoc* comparisons were highlighted (e.g figure 5). Non-significant post hoc comparisons have not been presented. In the method section of the revised manuscript, we will clarify that we'll report *post hoc* differences when they are observed.

During our study design, we decided to assess diet and genotype in parallel - as part of the same analysis. This seemed to us to be the most appropriate statistical method, so that we assessed dietary responses in both WT and GHS-R null mice.
>> Completed.

As this 5:2 is a very specific paradigm, it is furthermore difficult to compare these results to other studies and the conclusions are only valid for this specific pattern and timing of the intervention (6 weeks). It remains unclear why the authors have not first tried to establish a study with wildtype mice and a similar duration as in previous studies observing beneficial effects of intermitted fasting on neurogenesis. Like this, it would have been possible to make a statement if the 5:2 per se does not increase neurogenesis or if the 6 weeks exposure were just too short.

The reviewer raises this relevant point which we considered during the study design period. Given that we had previously reported significant modulation of AHN with a relatively short period of 30% CR (14 days followed by 14 days AL refeeding (Hornsby *et al*.2016)), we predicted that a 6 week course on the 5:2 paradigm (totalling 12 days of complete food restriction over the 6 week period) would provide a similar dietary challenge. The fact that we did not observe similar changes in AHN with this 5:2 paradigm is notable.
>> Completed.

The graphical representation of the data could also be improved. Below are a few examples listed:

1.) Figure 1 B and C, the same symbol and colours are used for the adolescent and adult animals, which makes the graphs hard to read. One colour and symbol per group throughout the manuscript would be better.

We thank the reviewer for this comment. We will amend the presentation of the graphs throughout the manuscript to ensure that they are easier to interpret.
>> Completed.

2.) The authors found no differences in the total number of Ki67 positive cells in the DG. However, Ki67 staining does not allow to conclude the type of cell which is proliferating. It would thus strengthen the findings if this analysis was combined with different markers, such as Sox2, GFAP and DCX.

Double labelling of Ki67 positive cells would allow for further insight into the identity of distinct proliferating cell populations. However, quantifying Ki67 immunopositive cells within the sub-granular zone of the GCL, as a single marker, is commonly used in studies of AHN. Given that

studies of intermittent fasting, calorie restriction and treatment with exogenous acyl-ghrelin report no effect on NPC cell division, we decided not to pursue this line of inquiry.
>> Completed.

3.) In Figure 3, the authors say that the diet increases the number of DCX in adolescent and adult mice, which is not clear when looking at the graph in 3B. Are there any significant differences when directly comparing the corresponding groups, for instance the WT AL vs the WT 5:2? It is further not clear how the authors distinguished the different types of DCX morphology-wise. The quantification in C and D would need to be illustrated by example images. Furthermore, the colour-code used in these graphs is not explained and remains Unclear

While the 3 way ANOVA does yield a significant overall effect for diet, we agree that it is indeed difficult to see a difference on the graph, although the mean values of the adolescent 5:2 animals are more prominent than the AL counterparts. Mean +/- SEM will be provided in the supplementary section of the revised manuscript. Furthermore, we will clarify the method used to identify distinct $DCX^+$ morphologies, include representative high-resolution images of each $DCX^+$ cell category, and amend the colour coding to avoid misinterpretation.
>> Completed. These data are presented in figure 3.

4) In Figure 5, the authors show that the number of new NSCs is significantly increased in the adolescent GHS-R-/- mice, independent of the diet, but this increase does not persist in the adult mice. They conclude that "the removal of GHS-R has a detrimental effect on the regulation of new NSC number..." this claim is not substantiated and needs to be reformulated. As the GHS-R-/- mice have a transcriptional blockage of Ghrs since start of its expression, would such an effect on NSC regulation not result in an overall difference in brain development, as ghrelin is also important during embryonic development?

This is an interesting point. However, we disagree that the statement "*the removal of GHS-R has a detrimental effect on the regulation of new NSC number*..." is unsubstantiated, since it does not exclude any developmental deficits in these mice that may account for the differences observed. Nonetheless, we will rephrase the sentence to clarify our intended point and remove any ambiguity.
>> Completed.

5.) In Figure 6, the authors asses spatial memory performance with a single behavioral test, the OIP. As these kind of tests are influenced by the animal's motivation to explore, it's anxiety levels, physical parameters (movement) etc., the interpretation of such a test without any additional measured parameters can be problematic. The authors claim that the loss of GHS-R expression impairs spatial memory performance. As the discrimination ratio was calculated, it is not possible to see if there is an overall difference in exploration time between genotypes. This would be a good additional information to display.

We thank the reviewer for this insight. We have open-field recordings that will now be analysed to assess general locomotor activity, anxiety and exploration behaviour. These data, alongside exploratory time of the mice during the OIP task will be incorporated into the revised manuscript.
>> Completed. These data are presented in figure 6.

Besides these points listed above, the methods are presented in such a way that they can be reproduced. The experiments contained 10-15 mice per group, which is a large enough group to

perform statistical analyses. As mentioned above, the statistical analysis over all 4 groups with p-values for the main effects should be followed by post hoc multiple comparison tests to allow the direct comparison of the corresponding groups.

**Reviewer #3 (Significance (Required)):**

In the last years, growing evidences suggested that IF might have positive effect on health in general and also for neurogenesis. However, a few recent studies report no effects on neurogenesis, using different IF paradigms. This study adds another proof that not all IF paradigms influence neurogenesis and shows that more work needs to be done to better understand when and how IF can have beneficial effects. This is an important finding for the neurogenesis field, but the results are only valid for this specific paradigm used here, which limits its significance. The reporting of such negative findings is however still important, as it shows that IF is not just a universal way to increase neurogenesis. In the end, such findings might have the potential to bring the field together to come up with a more standardized dietary intervention paradigm, which would be robust enough to give similar results across laboratories and mouse strains, and would allow to test the effect of genetic mutations on dietary influences of neurogenesis.

We thank the reviewer for their insightful and thorough feedback.

## 3. Description of the revisions that have already been incorporated in the transferred manuscript

*Please insert a point-by-point reply describing the revisions that were already carried out and included in the transferred manuscript. If no revisions have been carried out yet, please leave this section empty.*

The manuscript has not been revised at this stage.

## 4. Description of analyses that authors prefer not to carry out

*Please include a point-by-point response explaining why some of the requested data or additional analyses might not be necessary or cannot be provided within the scope of a revision. This can be due to time or resource limitations or in case of disagreement about the necessity of such additional data given the scope of the study. Please leave empty if not applicable.*

We have included in our replies to the reviewers a description of the amendments that we will make to our manuscript. Two requested revisions stand out as being unnecessary or cannot be provided within the scope of a revision.
The first was the request to perform the 5:2 study in older mice. This an interesting suggestion, however, the expense and time needed to maintain mice into old age (e.g >18 months) cannot be provided within the scope of our revision. In addition, given that we report no effect of the 5:2 paradigm on AHN in adolescent (7 week old) and adult (7 month old) mice, there is less justification for such a study in old mice.

# Authors' Response

The second request, that we disagree with, was to remove data relating to the GHS-R null mice (see reviewer 2, point 2). The role of ghrelin signalling via its receptor, GHS-R, is a central tenet of our hypothesis. Whilst our findings do not support a role for ghrelin signalling in the context of the 5:2 diet studied, we followed the scientific method to empirically test the stated hypothesis. While critiques of experimental design are welcome, the removal of such data may perpetuate publication bias in favour of positive outcomes and is something we wish to avoid.

## 1st Revision - Editorial Decision 6th Oct 2023

Dear Prof. Davies,

Thank you for submitting your revised manuscript. It has now been seen by all of the original referees.

As you can see, the referees find that the study is significantly improved during revision. However, referees #2 and #3 also point out some minor outstanding issues, which need to be addressed. In particular, referee #3 requests further revising the statements regarding the role of ghrelin receptor in regulation of AHN. Please address the remaining concerns and provide a point-by-point response. While we appreciate the comments of referee #3 on the significance of the findings, we still find the study of interest.

Moreover, I need you to address the editorial points below before I can accept the manuscript.

• Please provide 3-5 keywords for your study. These will be visible in the html version of the paper and on PubMed and will help increase the discoverability of your work.
• Please add a 'Disclosure Statement & Competing Interests' section after the Acknowledgements (https://www.embopress.org/page/journal/14693178/authorguide#conflictsofinterest)
• As per our format requirements, in the reference list, citations should be listed in alphabetical order and then chronologically, with the authors' surnames and initials inverted; where there are more than 10 authors on a paper, 10 will be listed, followed by 'et al.'. Moreover, we need DOIs only for preprints and datasets that have not been published yet. Please see https://www.embopress.org/page/journal/14693178/authorguide#referencesformat
• We note that the ORDCID ID of the co-corresponding author Dr. Timothy Wells is currently not linked. EMBO Press policy asks for all corresponding authors to link to their ORCID iDs. You can read about the change under "Authorship Guidelines" in the Guide to Authors here: https://www.embopress.org/page/journal/14693178/authorguide#authorshipguidelines

In order to link your ORCID iD to your account in our manuscript tracking system, please do the following:

1. Click the 'Modify Profile' link at the bottom of your homepage in our system.
2. On the next page you will see a box halfway down the page titled ORCID*. Below this box is red text reading 'To Register/Link to ORCID, click here'. Please follow that link: you will be taken to ORCID where you can log in to your account (or create an account if you don't have one)

3. You will then be asked to authorise Wiley to access your ORCID information. Once you have approved the linking, you will be brought back to our manuscript system.

We regret that we cannot do this linking on your behalf for security reasons.
• Please make sure that the funding information is complete in the manuscript submission system. We note that Cardiff University information is currently missing.
• We note the following regarding the figure callouts: Callouts for Fig 5C, Fig 6BCE-G are missing. The callouts for Appendix Table 2 , 3 and 6 need to be corrected as "Appendix Table S1" instead of "Table S1", etc.
• We realize that videos are mentioned in the manuscript text, but no files are provided.
• During our routine figure checks we noted the potential re-use of some panels in Figure 4D and Figure EV2 B, which is only allowed if the figures are derived from the same experiment, in which case it should be clearly stated in the figure legends along with the reason for double presentation.
• We realize that scale bar and its definition are missing for figure 3f. Also, the scale bar needs to be defined for figure EV2b.
• Papers published in EMBO Reports include a 'synopsis' and 'bullet points' to further enhance discoverability. Both are displayed on the html version of the paper and are freely accessible to all readers. The synopsis includes a short standfirst summarizing the study in 1 or 2 sentences (max 35 words) that summarize the paper and are provided by the authors and streamlined by the handling editor. I would therefore ask you to include your synopsis blurb and 3-5 bullet points listing the key experimental findings.
• In addition, please provide an image for the synopsis. This image should provide a rapid overview of the question addressed in the study but still needs to be kept fairly modest since the image size cannot exceed 550 x 300-600 pixels.

Thank you again for giving us to consider your manuscript for EMBO Reports, I look forward to your minor revision.

Kind regards,

Deniz Senyilmaz Tiebe

--
Deniz Senyilmaz Tiebe, PhD
Scientific Editor
EMBO Reports

Referee #1:

My report was already positive in the previous round of revision. The Authors provide solid evidence for their conclusion, the experiments are convincing and well explained. The quality of Figures has been improved. The Authors extensively answered to the points raised by all Reviewers and made substantial changes in several sections of the manuscript. Particularly, the corticosterone ELISA analysis was added.
On these bases, I think that the work is suitable for publication in EMBO reports. The novelty does not reside in some new positive results, but in assessing the absence of effect of the diet used in this study on adult hippocampal neurogenesis, thus leading to re-think the results obtained in previous (in my view, more superficial) studies. Last, but not least, the fact that the study confirms, and takes into account, the remarkable age-related reduction of adult neurogenesis in mice, an information that is often missed, thus leading to biases.

Referee #2:

In this revised manuscript, Gabarró-Solanas et al. investigate the effect of the 5:2 diet, an intermittent fasting-related paradigm, on adult hippocampal neurogenesis and cognition in adolescent and mature mice. Unlike other dietary interventions, the authors do not observe any robust effect on neuronal stem cell number, proliferation, differentiation, or survival. Overall, this is an improved manuscript in which data and manuscript text have been streamlined and edited for clarity. The authors provide additional experiments and analyses, including an independent cohort of adult WT mice on the 5:2 diet that was used to assess food intake using metabolic chambers.

The authors have adequately addressed most of the previously raised concerns. Below are a few minor points for the authors to consider:

1) The authors state that "Endpoint body weight analyses (Fig 1B, C; Table S1) revealed that 6-weeks of feeding on the 5:2 diet paradigm did not lead to significant weight loss compared to ad libitum controls in any of the genotypes, sexes or age groups under study ". However, there appears to be a clear visual separation of the weight for both adult and adolescent C57Bl6J mice (Figure 1B/C) indicated that male WT mice on the 5:2 diet gain less weight than the ad libitum counterparts.
2) Since there is no counterstaining for the Ki67 DAB staining, it may be helpful to draw the outline of the dentate gyrus in Figure 2AB. Similar to the previous version of the manuscript, this would facilitate the regional identification of the dentate gyrus.
3) The new metabolic chamber experiment indicates that male mice cumulatively consumed more food than their ad libitum counterparts (Figure EV1D,E). This is an important observation that could, in part, explain the lack of effect of the 5:2 paradigm. This finding should be incorporated into the discussion section.
4) The new graphical representation of the data largely facilitates the interpretation of the results. Some of the comparisons from the two-way ANOVA are only discussed in the manuscript text and it may be useful for significant effects to be somehow incorporated into the main figures.

Referee #3:

EMBOR-2023-57269V2 Robert et al., 2023

In this manuscript, Roberts and colleagues demonstrate that the 5:2 diet has no effect on NSC proliferation, adult hippocampal neurogenesis and spatial memory performance in adolescent and adult wildtype and ghrelin knockout mice. In the revised version, the authors have re-analyse and represent the data differently, and have also included additional data regarding the impact of the 5:2 regime on food intake and body weight, using metabolic chambers. In addition, the authors further characterized that the diet regime did not alter exploratory and anxiety behavior by analyzing data from the open field test.

The major criticism about the original version was that the authors always showed the 4 experimental groups together (adolescent vs adult and wt vs GHS-R-/-, with AL or 5:2 diet). This made it difficult to judge the effect of the main tested factor, namely the difference in diet. Following the suggestion to re-analyse and represent the data differently and also following the other reviewers suggestions to show males and females separately, the authors have now revisited all the data and improved the graphical presentation throughout the manuscript, which makes it much easier to follow. The main message of the study that the 5:2 diet does not affect AHN has not changed with the novel data analysis and shows that not all IF paradigms influence neurogenesis. While I'm supporting the publication of negative results in general, the results shown here are only valid for this very specific 5:2 feeding paradigm used, which limits its significance. They seem thus not to be within the scope of EMBO reports, asking for a "broad biological significance" of its published papers.

In the original version of the manuscript, the authors also observed statistical significances between wildtype and ghrelin knockout mice (main effect genotype), leading to quite strong statements in the highlight section ("Ghrelin-receptor regulates new neural stem cell and new neurone number in an age-related manner"; "Ghrelin-receptor is required for intact spatial memory"). With the new data analysis, the overall effects of the GHS-R knockout on AHN have become less clear, as almost

none of the comparisons show an effect, or if so only in specific subgroups and not consistent over different aspects analysed. This makes it difficult to interpret the role of ghrelin on AHN in this setting. As a consequence, the authors have removed the two highlights in the highlight section regarding the findings with the ghrelin knockout mice, but still keep some of these statements in the headers of the results part (e.g. "The 5:2 diet did not increase the number of new neural stem cells (NSCs) in the hippocampal DG, but GHS-R was needed for regulating new NSC number"; "Spatial memory performance was not enhanced by 5:2 diet, but GHSR was needed for intact memory function in adult males"). Looking at the graphs, these statements do not seem to be substantiated, as all the direct comparisons are non-significant. Thus, these statements should be carefully revised and the very small effect of GHS-R knockout on AHN should be better discussed in the discussion session.

Overall, with the very specific paradigm tested and the lack of a clear connection of the ghrelin knockout and AHN, this study seems to be better suited for another journal than EMBO reports.

Minor comment:
1. The representative images displayed in figure 4 and 5 should match the style of the previous figures, showing an example of each of the four groups, even if no differences are observed.

**Rebuttal**: we include below a point-by point response to reviewer comments.

Referee 1.
We thank referee 1 for constructive comments and queries regarding our manuscript.

Referee 2.
The manuscript has been amended in an attempt to improve interpretation of the data. We thank referee 2 for stating that these changes have improved the manuscript.
Referee 2's 'minor points' are addressed below;

1. We present data in fig1B/C relating to the change in body weight across the course of the study. Whilst there are clear and statistically significant reductions in this measure on days corresponding to food withdrawal in the 5:2 group, as mentioned by the referee, our statement refers to the lack of a change at the endpoint of the study.
2. We agree that annotating fig2A/B to highlight the dentate gyrus will be helpful. We have amended this figure.
3. The referee notes that male mice consume more food (cumulative) than their A/L counterparts, and that this could partially explain the lack of an effect of the 5:2 paradigm. We have amended the discussion to include, *'It is also possible that the 5:2 diet led to increased food intake, as observed in male mice in our pilot study, which may counter beneficial effects on neurogenesis'*.
4. We have included the key post-hoc statistical comparisons in the figures presented. In addition, we have also provided all the main statistical effects of each comparison in table format in the Appendix. We believe that this format provides clarity for the reader to accurately interpret the data.

Referee 3.
We thank the referee for highlighting that our amendments have improved the graphical presentation throughout the manuscript, making it easier to follow. The comments of the referee are addressed below.

1. Re-analysis of the data (e.g separating analysis of adolescent & adult mice, and male & female mice), in line with prior referee suggestions has altered the statistical significance of some comparisons, including the effect of genotype. Where the effect of genotype is statistically significant, we have included this main effect in the text. However, we have – in agreement with Referee 3 - removed these highlights from the sub-section headings.
2. Expanded view figures 2 & 3, respectively, include representative group images for new neurones (BrdU+/NeuN+), new stem cells (BrdU+/Sox2+/S100B-) and new astrocytes (BrdU+/Sox2+/S100B+).

## 2nd Revision - Editorial Decision 30th Oct 2023

Dear Jeff,

Thank you for submitting your revised manuscript and providing the additional information. I have now looked at everything and all is fine. Therefore, I am very pleased to accept your manuscript for publication in EMBO Reports.

Congratulations on a nice work!

Kind regards,

Deniz
--
Deniz Senyilmaz Tiebe, PhD
Editor
EMBO Reports
--

At the end of this email I include important information about how to proceed. Please ensure that you take the time to read the information and complete and return the necessary forms to allow us to publish your manuscript as quickly as possible.

As part of the EMBO publication's Transparent Editorial Process, EMBO reports publishes online a Review Process File to accompany accepted manuscripts. As you are aware, this File will be published in conjunction with your paper and will include the referee reports, your point-by-point response and all pertinent correspondence relating to the manuscript.

If you do NOT want this File to be published, please inform the editorial office within 2 days, if you have not done so already, otherwise the File will be published by default [contact: emboreports@embo.org]. If you do opt out, the Review Process File link will point to the following statement: "No Review Process File is available with this article, as the authors have chosen not to make the review process public in this case."

Should you be planning a Press Release on your article, please get in contact with emboreports@wiley.com as early as possible, in order to coordinate publication and release dates.

Thank you again for your contribution to EMBO reports and congratulations on a successful publication. Please consider us again in the future for your most exciting work.

Yours sincerely,

Deniz Senyilmaz Tiebe, PhD
Editor
EMBO Reports

*********************************************************************************

THINGS TO DO NOW:

You will receive proofs by e-mail approximately 2-3 weeks after all relevant files have been sent to our Production Office; you should return your corrections within 2 days of receiving the proofs.

EMBO Press participates in many Publish and Read agreements that allow authors to publish Open Access with reduced/no publication charges. Check your eligibility: https://authorservices.wiley.com/author-resources/Journal-Authors/open-access/affiliation-policies-payments/index.html

Please inform us if there is likely to be any difficulty in reaching you at the above address at that time. Failure to meet our deadlines may result in a delay of publication, or publication without your corrections.

All further communications concerning your paper should quote reference number EMBOR-2023-57269V3 and be addressed to emboreports@wiley.com.

Should you be planning a Press Release on your article, please get in contact with emboreports@wiley.com as early as possible, in order to coordinate publication and release dates.

***
Rev_Com_number: RC-2022-01714
New_manu_number: EMBOR-2023-57269V3
Corr_author: Davies
Title: The 5:2 diet does not increase adult hippocampal neurogenesis or enhance spatial memory in mice

## EMBO Press Author Checklist

| | |
|---|---|
| Corresponding Author Name: Jeff Davies | |
| Journal Submitted to: EMBO Reports | |
| Manuscript Number: EMBOR-2023-57269V1 | |

**USEFUL LINKS FOR COMPLETING THIS FORM**
The EMBO Journal - Author Guidelines
EMBO Reports - Author Guidelines
Molecular Systems Biology - Author Guidelines
EMBO Molecular Medicine - Author Guidelines

**Reporting Checklist for Life Science Articles (updated January**
This checklist is adapted from Materials Design Analysis Reporting (MDAR) Checklist for Authors. MDAR establishes a minimum set of requirements in transparent reporting in the life sciences (see Statement of Task: 10.31222/osf.io/9sm4x). Please follow the journal's guidelines in preparing your
**Please note that a copy of this checklist will be published alongside your article.**

### Abridged guidelines for figures
**1. Data**
The data shown in figures should satisfy the following conditions:
- the data were obtained and processed according to the field's best practice and are presented to reflect the results of the experiments in an accurate and unbiased manner.
- ideally, figure panels should include only measurements that are directly comparable to each other and obtained with the same assay.
- plots include clearly labeled error bars for independent experiments and sample sizes. Unless justified, error bars should not be shown for technical
- if n<5, the individual data points from each experiment should be plotted. Any statistical test employed should be justified.
- Source Data should be included to report the data underlying figures according to the guidelines set out in the authorship guidelines on Data

**2. Captions**
Each figure caption should contain the following information, for each panel where they are relevant:
- a specification of the experimental system investigated (eg cell line, species name).
- the assay(s) and method(s) used to carry out the reported observations and measurements.
- an explicit mention of the biological and chemical entity(ies) that are being measured.
- an explicit mention of the biological and chemical entity(ies) that are altered/varied/perturbed in a controlled manner.
- the exact sample size (n) for each experimental group/condition, given as a number, not a range;
- a description of the sample collection allowing the reader to understand whether the samples represent technical or biological replicates (including how many animals, litters, cultures, etc.).
- a statement of how many times the experiment shown was independently replicated in the laboratory.
- definitions of statistical methods and measures:
  - common tests, such as t-test (please specify whether paired vs. unpaired), simple χ2 tests, Wilcoxon and Mann-Whitney tests, can be unambiguously identified by name only, but more complex techniques should be described in the methods section;
  - are tests one-sided or two-sided?
  - are there adjustments for multiple comparisons?
  - exact statistical test results, e.g., P values = x but not P values < x;
  - definition of 'center values' as median or average;
  - definition of error bars as s.d. or s.e.m.

**Please complete ALL of the questions below.**
**Select "Not Applicable" only when the requested information is not relevant for your study.**

### Materials

| Newly Created Materials | Information included in the manuscript? | In which section is the information available? (Reagents and Tools Table, Materials and Methods, Figures, Data Availability Section) |
|---|---|---|
| New materials and reagents need to be available; do any restrictions apply? | Not Applicable | |

| Antibodies | Information included in the manuscript? | In which section is the information available? (Reagents and Tools Table, Materials and Methods, Figures, Data Availability Section) |
|---|---|---|
| For **antibodies** provide the following information:<br>- Commercial antibodies: RRID (if possible) or supplier name, catalogue number and or/clone number<br>- Non-commercial: RRID or citation | Yes | Methods |

| DNA and RNA sequences | Information included in the manuscript? | In which section is the information available? (Reagents and Tools Table, Materials and Methods, Figures, Data Availability Section) |
|---|---|---|
| **Short novel DNA or RNA including primers, probes**: provide the sequences. | Not Applicable | |

| Cell materials | Information included in the manuscript? | In which section is the information available? (Reagents and Tools Table, Materials and Methods, Figures, Data Availability Section) |
|---|---|---|
| **Cell lines:** Provide species information, strain. Provide accession number in repository **OR** supplier name, catalog number, clone number, and/**OR** RRID. | Not Applicable | |
| **Primary cultures:** Provide species, strain, sex of origin, genetic modification status. | Not Applicable | |
| Report if the cell lines were recently **authenticated** (e.g., by STR profiling) and tested for mycoplasma contamination. | Not Applicable | |

| Experimental animals | Information included in the manuscript? | In which section is the information available? (Reagents and Tools Table, Materials and Methods, Figures, Data Availability Section) |
|---|---|---|
| **Laboratory animals or Model organisms:** Provide species, strain, sex, age, genetic modification status. Provide accession number in repository **OR** supplier name, catalog number, clone number, **OR** RRID. | Yes | Methods |
| **Animal observed in or captured from the field:** Provide species, sex, and age where possible. | Not Applicable | |
| Please detail **housing and husbandry conditions**. | Yes | Methods |

| Plants and microbes | Information included in the manuscript? | In which section is the information available? (Reagents and Tools Table, Materials and Methods, Figures, Data Availability Section) |
|---|---|---|
| **Plants:** provide species and strain, ecotype and cultivar where relevant, unique accession number if available, and source (including location for collected wild specimens). | Not Applicable | |
| **Microbes:** provide species and strain, unique accession number if available, and source. | Not Applicable | |

| Human research participants | Information included in the manuscript? | In which section is the information available? (Reagents and Tools Table, Materials and Methods, Figures, Data Availability Section) |
|---|---|---|
| If collected and within the bounds of privacy constraints report on age, sex and gender or ethnicity for all study participants. | Not Applicable | |

| Core facilities | Information included in the manuscript? | In which section is the information available? (Reagents and Tools Table, Materials and Methods, Figures, Data Availability Section) |
|---|---|---|
| If your work benefited from core facilities, was their service mentioned in the acknowledgments section? | Not Applicable | |

### Design

| Study protocol | Information included in the manuscript? | In which section is the information available? (Reagents and Tools Table, Materials and Methods, Figures, Data Availability Section) |
|---|---|---|

| | | |
|---|---|---|
| If study protocol has been **pre-registered, provide DOI in the manuscript**. For clinical trials, provide the trial registration number **OR** cite DOI. | Not Applicable | |
| Report the **clinical trial registration number** (at ClinicalTrials.gov or equivalent), where applicable. | Not Applicable | |

| Laboratory protocol | Information included in the manuscript? | In which section is the information available? (Reagents and Tools Table, Materials and Methods, Figures, Data Availability Section) |
|---|---|---|
| Provide DOI OR other citation details if **external detailed step-by-step protocols** are available. | Not Applicable | |

| Experimental study design and statistics | Information included in the manuscript? | In which section is the information available? (Reagents and Tools Table, Materials and Methods, Figures, Data Availability Section) |
|---|---|---|
| Include a statement about **sample size** estimate even if no statistical methods were used. | Not Applicable | |
| Were any steps taken to minimize the effects of subjective bias when allocating animals/samples to treatment (e.g. **randomization procedure**)? If yes, have they been described? | Yes | Methods |
| Include a statement about **blinding** even if no blinding was done. | Yes | Methods |
| Describe **inclusion/exclusion criteria** if samples or animals were excluded from the analysis. Were the criteria pre-established?<br><br>If sample or data points were omitted from analysis, report if this was due to attrition or intentional exclusion and provide justification. | Yes | Methods |
| For every figure, are **statistical tests** justified as appropriate? Do the data meet the assumptions of the tests (e.g., normal distribution)? Describe any methods used to assess it. Is there an estimate of variation within each group of data? Is the variance similar between the groups that are being statistically compared? | Yes | Methods |

| Sample definition and in-laboratory replication | Information included in the manuscript? | In which section is the information available? (Reagents and Tools Table, Materials and Methods, Figures, Data Availability Section) |
|---|---|---|
| In the figure legends: state number of times the experiment was **replicated** in laboratory. | Not Applicable | |
| In the figure legends: define whether data describe **technical or biological replicates**. | Not Applicable | |

## Ethics

| Ethics | Information included in the manuscript? | In which section is the information available? (Reagents and Tools Table, Materials and Methods, Figures, Data Availability Section) |
|---|---|---|
| Studies involving **human participants**: State details of **authority granting ethics approval** (IRB or equivalent committee(s), provide reference number for approval. | Not Applicable | |
| Studies involving **human participants**: Include a statement confirming that **informed consent** was obtained from all subjects and that the experiments conformed to the principles set out in the WMA Declaration of Helsinki and the Department of Health and Human Services Belmont Report. | Not Applicable | |
| Studies involving **human participants:** For publication of **patient photos**, include a statement confirming that consent to publish was obtained. | Not Applicable | |
| Studies involving experimental **animals**: State details of **authority granting ethics approval** (IRB or equivalent committee(s), provide reference number for approval. Include a statement of compliance with ethical regulations. | Yes | Methods |
| Studies involving **specimen and field samples:** State if relevant **permits** obtained, provide details of authority approving study; if none were required, explain why. | Not Applicable | |

| Dual Use Research of Concern (DURC) | Information included in the manuscript? | In which section is the information available? (Reagents and Tools Table, Materials and Methods, Figures, Data Availability Section) |
|---|---|---|
| Could your study fall under dual use research restrictions? Please check biosecurity documents and list of **select agents and toxins** (CDC): https://www.selectagents.gov/sat/list.htm | Not Applicable | |
| If you used a select agent, is the security level of the lab appropriate and reported in the manuscript? | Not Applicable | |
| If a study is subject to dual use research of concern regulations, is the name of the **authority granting approval and reference number** for the regulatory approval provided in the manuscript? | Not Applicable | |

## Reporting

The MDAR framework recommends adoption of discipline-specific guidelines, established and endorsed through community initiatives. Journals have their own policy about requiring specific guidelines and recommendations to complement MDAR.

| Adherence to community standards | Information included in the manuscript? | In which section is the information available? (Reagents and Tools Table, Materials and Methods, Figures, Data Availability Section) |
|---|---|---|
| State if relevant guidelines or checklists (e.g., **ICMJE, MIBBI, ARRIVE, PRISMA**) have been followed or provided. | Not Applicable | |
| For **tumor marker prognostic studies,** we recommend that you follow the **REMARK** reporting guidelines (see link list at top right). See author guidelines, under 'Reporting Guidelines'. Please confirm you have followed these guidelines. | Not Applicable | |
| For **phase II and III randomized controlled trials**, please refer to the **CONSORT** flow diagram (see link list at top right) and submit the CONSORT checklist (see link list at top right) with your submission. See author guidelines, under 'Reporting Guidelines'. Please confirm you have submitted this list. | Not Applicable | |

## Data Availability

| Data availability | Information included in the manuscript? | In which section is the information available? (Reagents and Tools Table, Materials and Methods, Figures, Data Availability Section) |
|---|---|---|
| Have **primary datasets** been deposited according to the journal's guidelines (see 'Data Deposition' section) and the respective accession numbers provided in the Data Availability Section? | Yes | Data availability section (BioStudies accession number S-BSST1218) |
| Were **human clinical and genomic datasets** deposited in a public access-controlled repository in accordance to ethical obligations to the patients and to the applicable consent agreement? | Not Applicable | |
| Are **computational models** that are central and integral to a study available without restrictions in a machine-readable form? Were the relevant accession numbers or links provided? | Not Applicable | |
| If publicly available data were reused, provide the respective **data citations in the reference list**. | Not Applicable | |
